# CoMOGrad and PHOG: From Computer Vision to Fast and Accurate Protein Tertiary Structure Retrieval

**DOI:** 10.1038/srep13275

**Published:** 2015-08-21

**Authors:** Rezaul Karim, Mohd. Momin Al Aziz, Swakkhar Shatabda, M. Sohel Rahman, Md. Abul Kashem Mia, Farhana Zaman, Salman Rakin

**Affiliations:** 1 A*l*EDA Group, Department of Computer Science and Engineering, Bangladesh University of Engineering and Technology, Bangladesh; 2 Department of Computer Science and Engineering, United International University, Bangladesh

## Abstract

The number of entries in a structural database of proteins is increasing day by day. Methods for retrieving protein tertiary structures from such a large database have turn out to be the key to comparative analysis of structures that plays an important role to understand proteins and their functions. In this paper, we present fast and accurate methods for the retrieval of proteins having tertiary structures similar to a query protein from a large database. Our proposed methods borrow ideas from the field of computer vision. The speed and accuracy of our methods come from the two newly introduced features- the co-occurrence matrix of the oriented gradient and pyramid histogram of oriented gradient- and the use of Euclidean distance as the distance measure. Experimental results clearly indicate the superiority of our approach in both running time and accuracy. Our method is readily available for use from this website: http://research.buet.ac.bd:8080/Comograd/.

Proteins perform a large array of functions within a living organism. Proteins are polymers of amino-acid monomers. Linear sequence of amino acids is called the primary structure of a protein. There are three other levels of structural complexity: secondary, tertiary and quaternary. Secondary structure is the local spatial arrangement of the backbone atoms formed by the intramolecular and intermolecular hydrogen bonding of amide groups. Tertiary structure refers to the three-dimensional structure of an entire polypeptide chain and quaternary structure is the spatial arrangement of two or more polypeptide chains known as sub-units.

Every protein has a unique, stable and kinetically accessible^1^ three dimensional structure or tertiary structure also known as the native structure. The functionalities of a protein are principally correlated to its tertiary structure. That is why a protein ceases to function when its tertiary structure is broken by denaturation at a high temperature despite that its primary structure may still remain intact^2^. Proteins with similar tertiary structures have similar ligand binding sites and pockets^3^. Moreover, tertiary structures are more conserved than amino acid sequences during evolution^4^. Misfolded proteins are the cause of many critical diseases like Alzheimer’s disease^5^. Therefore, analysis of similar tertiary structures is of great importance in the function prediction of novel proteins, study of evolution, disease diagnosis, drug discovery, antibody design and in many other fields.

Following the determination of the first tertiary structure in 1958 using crystallography^6^, biologists have successfully determined a large number of structures by now. Due to recent developments in X-ray crystallography and nuclear magnetic resonance imaging, there has been a rapid increase in the number of experimentally determined structures stored in the world-wide repository, Protein Data Bank (PDB)^7^ (http://wwpdb.org/). RCSB PDB is the primary repository for known protein structures. As of February 11, 2014, the number of protein structures stored in PDB has been more than 97,789. With this increase in the number of known protein structures, the need for fast and accurate protein tertiary structure retrieval algorithm is now greater than ever. In the protein tertiary structure retrieval problem, the task is to retrieve proteins having the most similar structure to the query protein from the known structure database. The results are ranked based on similarity or distance measures.

There exist numerous approaches in the literature focusing on fast and accurate retrieval of protein tertiary structures. The traditional way to compare structures is to treat each one as a rigid three-dimensional object and superimpose one on the other. Differences are then calculated using different distance metrics, e.g., least-squares method. In the pattern recognition literature, the structures are often represented by feature vectors and similarity or dissimilarity is measured by comparing the feature vectors with one another. As a feature to represent the tertiary structure of a protein chain, the *α* carbon distance matrix is widely used in the literature^8^,^9^. Here, the 3D coordinate data is mapped to a two dimensional feature matrix. The *α* carbon distance matrix gives the intra-molecular distances of *α* carbons in a protein chain. This *α* carbon distance matrix resembles the backbone of the tertiary structure of a protein and the secondary structure elements conserved in it. Notably, *α* carbon distance matrix based exact algorithms run in *O*(*N*!) time assuming that the input matrix is of size *N* × *N*.

*DALI*^8^ compares structures by aligning the *α* carbon distance matrices. Each distance matrix is decomposed into sub-matrices of fixed size that are called the elementary contact patterns. It compares those contact patterns (pair-wise), and store the matching pairs in a list with a matching score. Then, it assembles pairs in the correct order using Monte Carlo optimization to yield the overall alignment and the final matching score. In a later version, it has used branch and bound method to assemble the pairs^10^. The *CE* method^11^ also takes the *α* carbon distance matrix as the feature vector and uses combinatorial extension and Monte Carlo optimization to compare protein structures in a way that is quite similar to *DALI*. Both *DALI* and *CE* require lots of computation and hence the corresponding web servers respond to the query requests via email only after a certain period that is required to complete the costly processing. A faster approach based on the *α* carbon distance matrix as a feature is *MatAlign*^12^. It provides an *O*(*N*^4^) dynamic programming solution where *N* is the dimension of the distance matrix. *MatAlign* compares distance matrix of the query protein and the target protein row by row and builds up a dynamic programming table based on a row by row matching score. Then, it aligns rows of the query protein with the rows of the target protein to maximize the corresponding matching score. So, higher matching score indicates higher similarity between the protein structures.

A celebrated web server named ProteinDBS for protein structure retrieval algorithm has been reported in^13^ which is based on the algorithm presented in^14^. The technique used in^14^ has some similarities with our work which will be discussed in a later section. Subsequently, a second and enhanced version of the system called ProteinDBS v2.0 has been developed which is reported in^15^. ProteinDBS v2.0 incorporates the global comparison method using the knowledge-based feature extraction and online database indexing method of^16^ and the local comparison method of^17^. Another interesting method called ‘3D Surfer’ has been presented in^18^ which compares surface shape similarity rather than backbone structure similarity. 3D Surfer uses 3D zernike descriptor^19^ as the representative feature of the surface shape.

Marsolo *et al*.^20^ have introduced a wavelet based approach that resizes the distance matrices of the protein structures before the actual comparison is done. Later on Mirceva *et al*. have introduced *MASASW*^21^ that uses wavelet coefficients of distance matrices as the feature vector. It has been shown in^21^ that *Daubechies-2 wavelets* can provide improved accuracy over others. *MASASW* transforms all the *α* carbon distance matrices into a 32 × 32 matrix by interpolation and wavelet transformation. It then compares them like *MatAlign* but with a sliding window to reduce the number of comparisons. The time complexity of *MASASW* is *O*(*wWN*^2^) where *w* and *W* are window sizes and *N* is the dimension of the distance matrices. As has been mentioned above, *MASASW* assumes *N* = 32.

Although several methods are found in the literature for protein structure retrieval, the quest for even faster and more accurate methods still continues as the number of known protein structures grows at a rapid pace. In this paper, we present an extremely fast and highly accurate method of retrieving proteins from a large database. In particular, here we present an ultra fast algorithm based on two novel feature vectors. These are the **Co**-occurrence **M**atrix of the **O**riented **Gra**dient of **D**istance Matrices (CoMOGrad) and the **P**yramid **H**istogram of **O**riented **G**radient (PHOG). Additionally, as will be reported later, our proposed algorithm gives more accurate results than the state of the art methods. Very briefly, the speed and accuracy of our method can be attributed to the novel features from the field of computer vision and pattern recognition. Our aim has been to introduce a feature which does not require any complex algorithm to compare the tertiary structures. And indeed, a simple distance measure to calculate the distance between the two vector quantities has been used in our approach.

At this point a brief discussion on our rationale to use CoMOGrad and PHOG as our feature vectors is in order. As has already been mentioned above, some previous methods in the literature have used the *α* carbon distance matrix as their feature vector. Upon analyzing the tertiary structure and *α* carbon distance matrix represented as a gray-scale image, we have observed that not all data in the matrix are equally important. We further have realized that the co-occurrence matrix of the oriented gradient of the distance matrix is the most important feature with respect to the comparison of tertiary structures. These observations and findings motivate us to use these as our feature vectors. Finally, we have anticipated that the Euclidean distance or 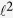 norm of our novel features as the distance measure would outperform the widely used costly alignment distance/similarity measure of *α* carbon distance matrices. In the sequel, the combination of the above ideas has given us an extremely fast method without sacrificing the accuracy.

Note that the idea of borrowing features from the field of computer vision and applying it in protein structure retrieval is not new. In particular, Chi *et al*. also used a similar approach in^14^ which was later implemented in the ProteinDBS server^13^. In fact both our method and the method of Chi *et al*. start from generating distance matrix images. In particular, Chi *et al*. extracted some texture features of the images from the original size images and diagonally partitioned images. Subsequently, they used well known features of image processing. The key difference between the two methods lies in that, unlike their method, we have taken gradients of the image first and then have used the co-occurrence matrix and histogram. There exist differences in the implementation level as well: Chi *et al*. used multiple CPU clusters to improve speed and their search space is also distributed, whereas, our method gives a single CPU solution.

Another interesting technique from computer vision called geometric hashing^22^,^23^,^24^ has also been successfully applied in structural comparison of proteins in the literature^25^,^26^,^27^. Broadly speaking, the methods based on geometric hashing take either 3 atoms or 3 alpha carbon atom triplets from the protein chains. From *n* atoms, among 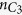 triplets, they take triplets with some restrictions. At this point they have a set of triplets. Then they prepare a hash table with sides of the triangle as keys to the hash table. Then they selectively align atoms of the two chains using the hash table. Actually, they use this hash table approach to compare the coordinates of the two chains with translating coordinate systems to the selected bases formed by the triplets. This makes their approach robust against translation. What makes geometric hashing different from our method is the fact that to make the comparison translation invariant, we have followed alpha carbon distance matrix based approach. This distance matrix transforms a 3D structure to a 2D matrix which can also be considered as an image. And these combined together achieve translation, rotation and scale invariance.

## Methods

**Figure 1:**
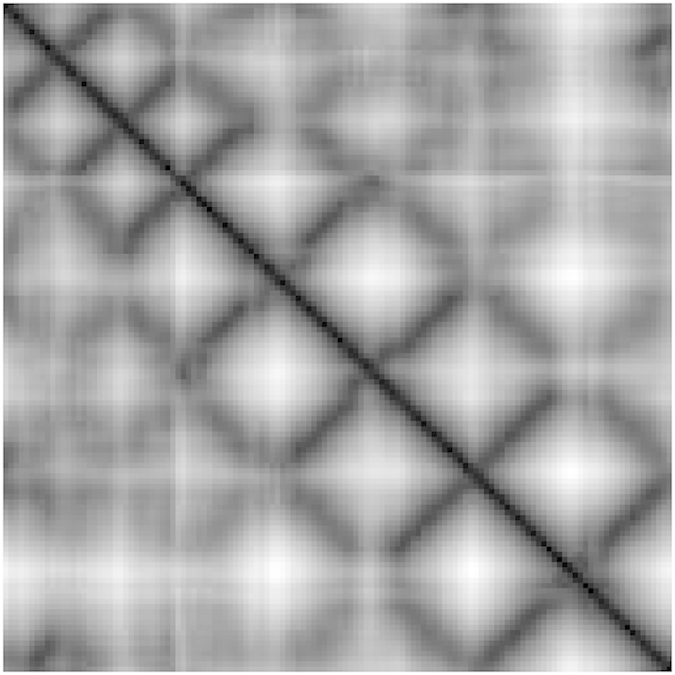
Representation of *β* sheets of domain d1n4ja^36^ in *α* carbon distance matrix gray-scale image.

**Figure 2:**
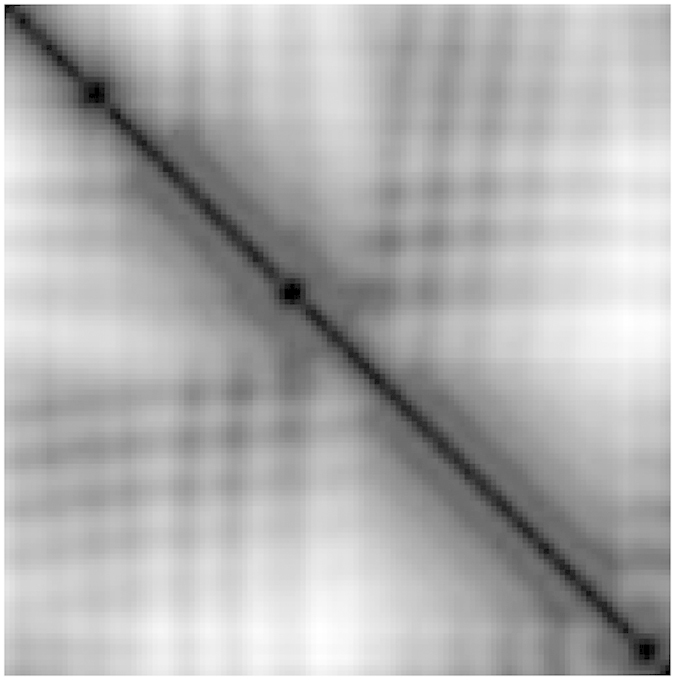
Representation of *α* helices of domain d1irqa^37^ in *α* carbon distance matrix gray-scale image.

We have carefully analyzed the gray-scale images from the *α* carbon distance matrices and the tertiary structures. We have observed that the *α* helices and the anti-parallel beta sheets appear as dark lines parallel to the diagonal dark line and parallel beta sheets appear as dark lines normal to the diagonal dark line. Beta sheets of two strips appear as one dark line normal to the diagonal; beta sheets of three strips appear as two dark lines normal to the diagonal and one dark line parallel to the diagonal. In general, for a standard beta sheet, the number of points of co-occurrence of parallel and anti-parallel diagonal lines depends on the number of strips in the beta sheets. Again, the number of single parallel lines depends on the number of standard *α* helices. Moreover, lengths of those lines near the diagonal region are proportional to the lengths of the *α* helices. The distance of the parallel lines from the diagonal dark line is proportional to the radius of the *α* helix. Figure 1 depicts the corresponding *α* carbon distance matrix of a tertiary structure of a protein with beta sheets as a gray scale image. In the gray scale image, the 7 anti-parallel dark lines near the diagonal dark line correspond to the presence of 8 beta sheets in the corresponding protein structure and the lengths of those dark lines are proportional to the lengths of the beta sheets. Figure 2 represents the gray scale image of the corresponding *α* carbon distance matrix of a protein tertiary structure with two alpha helices. Here in the image, the two parallel dark lines near the diagonal dark line correspond to the presence of two *α* helices in the protein structure and the lengths of the dark lines are proportional to the lengths of the *α* helices.

In the contemporary literature of computer vision and digital image processing^28^ the lines in digital images are usually recognized from the gradient of the images. This leads us to believe that, the co-occurrence of gradient angles represents the secondary structure elements more precisely than just the distance matrix image. The tertiary structure corresponds to the presence of the secondary structure elements (SSEs), their size and position in the chain and their orientation. From the images of protein structures and their corresponding *α* carbon distance matrix gray-scale image, it is clear that the SSEs are represented by the orientation of the dark lines at the near diagonal region of the *α* carbon distance matrix image. The positions of the SSEs in a protein chain are represented by the positions of the dark lines at the near diagonal region of the image. Here, *near diagonal region* refers to the region nearby the diagonal dark line. The sizes of the SSEs are represented by the lengths of the dark lines at the near diagonal region. The orientation of the SSEs are represented by the presence of the dark lines at the far diagonal regions and the darkness and orientation of those dark lines. Here, *far diagonal region* is the region in the image that is distant from the main diagonal dark line. Therefore, we need to incorporate the gradient orientation angle along with the gradient magnitude and gradient spatial orientation to incorporate the orientation of the SSEs in the feature vector. We also need co-occurrence of gradient angles. Based on our study and analysis, we introduce **Co**-occurrence **M**atrix of the **O**riented **Grad**ient (CoMOGrad) as our first feature vector which incorporates gradient orientation angles and the co-occurrence of the gradient orientation angles. This feature enables us to devise an algorithm that can facilitate extremely fast comparison. However, this speed is achieved at the cost of a slight decrease in the accuracy. Subsequently, we introduce another feature vector, namely, the **P**yramid **H**istogram of **O**riented **G**radient (PHOG) by incorporating the spatial information and gradient magnitude. The combination of both CoMOGrad and PHOG features results in an algorithm that is not only faster but also highly accurate.

### Feature vectors and feature extraction

#### Mapping 3D coordinates to 2D function

Distance matrix of the *α* carbons in residues is a good candidate to transform the 3D structure to the corresponding 2D vector representation as shown in *MASASW*^21^ and the wavelet based approach by Marsolo *et al*.^20^. This distance matrix gives the pairwise distance between all pair s of *α* carbons in the polypeptide chain. Proteins with similar tertiary structures will have similar distance matrices and vice versa. As stated earlier, if we consider the matrix as a monochromatic image, *α*-helices and parallel *β*-sheets will appear as dark lines parallel to the main diagonal and antiparallel beta sheets will appear as dark lines normal to the main diagonal. This distance matrix feature also has a very appealing property: this is translation, scaling and rotation invariant.

Interestingly, since it is a two dimensional matrix like a digital image we can easily apply image processing and computer vision algorithms on it. Similarly, we can use this matrix as an adjacency matrix and interpret it as a graph. Subsequently, graph theory techniques may also be applied to solve the tertiary structure retrieval problem with this feature. In this paper, we apply ideas from the field of image processing and computer vision. Most recently these ideas have got their niche in pedestrians and car detection^29^,^30^.

### Scaling C*α*-C*α* distance matrix images to the same dimension

#### Bi-cubic interpolation

As different protein chains have different number of *α* carbons, the dimensions of their *α* carbon distance matrices vary. Therefore, a natural approach is to scale the distance matrices to the same dimension. For scaling the distance matrices, we use the methods of digital image processing used for image resizing. At first, we scale all the images to the dimension that is a power of 2 and the nearest to their original dimension. As an example, if the image dimension is 80 × 80 we scale it to 64 × 64 and if the original image dimension is 100 × 100 we scale to 128 × 128. For this step, we use bi-cubic interpolation.

Note that similar scaling approaches have also been adopted by other researchers in the literature (e.g.,^21^). However, we do realize that once scaled, the magnitudes of gradients could mean different things in different size structures. This is why with respect to the scaling we are faced with a dilemma: should we scale or should we not. In the sequel we end up implementing both versions (i.e., with and without scaling) of our final algorithm.

#### Wavelet transform

After scaling the images as mentioned above, we apply wavelet transform to transform all the images to the same dimension. Notably, wavelet transform is the most widely used technique for image scaling or resizing in digital image processing. Using wavelet transform, we scale all images to 128 × 128 dimension as, during our study, most of the images in the previous step have been found to be of that dimension. For wavelet filter we use Daubechies-2 wavelet^31^ as this filter has been shown to have outperformed other traditional wavelets for protein structure feature representation in *MASASW*^21^. Wavelet transform of an image gives four images, namely, the *approximate detail, horizontal detail, diagonal detail* and *vertical detail*. Each of these images is half of the original image in dimension. We take the approximate detail to scale a large image to a smaller size since this is the approximate sub sampled image. For the images with dimension greater than 128 × 128, we perform wavelet transform on each image multiple times to get the approximate coefficient of size 128 × 128. For an image with dimension less than 128 × 128, we first perform wavelet transform on it. Then using bi-cubic interpolation, we scale all four coefficients to twice their initial size. After that, applying inverse wavelet transform on the four scaled wavelet coefficients, we get the original image scaled up to twice its initial size. With repeated application of scaling up (down) the images that are smaller (higher) than dimension 128 × 128, we finally get all of them in the desired dimension of 128 × 128.

### Novel features from scaled C*α*-C*α* distance matrix images

#### Co-occurrence matrix of oriented gradient (CoMOGrad)

After having all the images at dimension 128 × 128 we extract our CoMOGrad feature. First we take the gradient of each of the images and compute the gradient angle and magnitude. As the angle values are continuous quantities, we have to quantize those values. For quantization, we have tuned the number of quantization bins as a parameter. With experiments using various bin sizes (9, 16, 32 etc), we have found that using 16 bins with a bin size of 22.5 degree gives excellent results. After quantization to 16 bins, we compute co-occurrence matrix which is a 16 × 16 matrix. We convert this 16 × 16 matrix to a vector of size 256. This is our CoMOGrad feature vector. With this feature, we can simply take Euclidean distance to compare structures rather than using the alignment technique of *α* carbon distance matrices used by *MASASW* and *MatAlign*. As anticipated, introduction of this feature makes the comparison method much simpler and faster.

#### Pyramid histogram of oriented gradient (PHOG)

**Figure 3:**
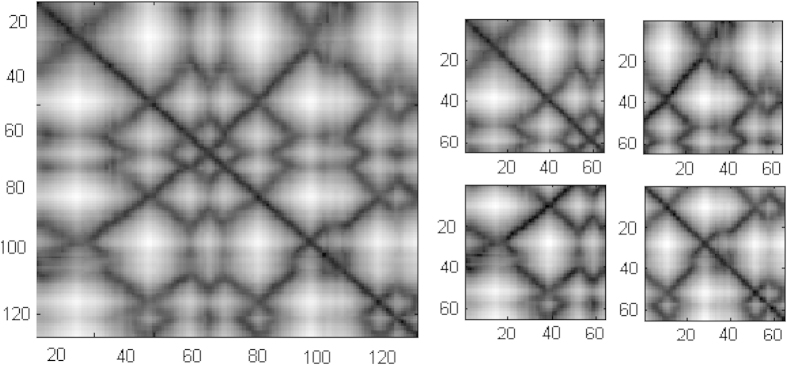
Level 1 quad tree of *α* carbon distance matrix image.

The use of CoMOGrad gives us an ultra fast structure retrieval algorithm. However it achieves this speed at the cost of some reduction in accuracy. From the discussion in the previous sections, it is clear that we have to incorporate the gradient magnitude and spatial orientation of gradient along with the angular orientation of gradient to accurately capture the tertiary structure of a protein. The CoMOGrad feature only includes the angular orientation of gradient and the co-occurrence of angular orientation of gradient. To incorporate the gradient magnitude and the spatial orientation of gradient along with the angular orientation of gradient, we extract another feature named pyramid histogram of oriented gradient (PHOG) together with our CoMOGrad feature to improve the accuracy. PHOG was first proposed and successfully used in object classification and pattern recognition by Bosch *et al*. in^32^. We create a quad tree of the original image with the original image at the root as follows. Each node of the quad tree has four children, namely, *top-left*, *top-right*, *bottom-left* and *bottom right*. Each of these images is of size one fourth of the original image. In Fig. 3, we have shown a quad tree up to level 1. In our experiments, we have taken the quad tree up to level 3 and have achieved excellent results. For quad tree up to level 3, there are 1 + 4 + 4 × 4 + 4 × 4 × 4=85 nodes. For each node, we create gradient orientation histogram with 9 bins with a bin size of 40 degree. Now, we have 85 × 9 = 765 features. We incorporate these 765 features to a vector of size 765. Then, we normalize the vector by dividing it with the sum of its 765 components. This is our PHOG feature vector. Now, PHOG combined with CoMOGrad gives a total of 256 + 765 = 1021 features.

### Distance Measure

We use the Euclidean distance or 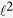 norm as the distance measure for our new features. PHOG combined with CoMOGrad can be seen as a vector of length 1021. Note that, we do not apply any weighting scheme on our two features. Suppose, *f*_*q*_ and *f*_*i*_ denote the feature vectors of the query protein *q* and a protein *i* in the database, respectively. Then the distance score 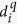 of protein *q* and *i* would be calculated according to Eqn. (1) below.


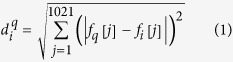


Clearly, the above distance measure can be calculated in *O*(*N*) time, where *N* = 1021 is the size of our feature vector. Also, recall that for CoMOGrad alone, *N* = 256 only. Our algorithm needs to compute 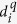 for each protein *i* in the database and then sort the results to rank them.

## Results and Discussion

We have implemented our algorithm in Java (jdk 1.6) with Netbeans IDE and MySQL database. The feature extraction has been done Using *MATLAB* R2012. The experiments have been conducted on GNU/Linux debian ubuntu i686 operating system. We have used a machine having *Intel* (R) Core(TM) i5 3470 CPU 3.20 GHz with 4 GB RAM. We have compared our methods with the tertiary structure retrieval method, *MASASW*^21^, which is shown to be the best performer in the literature to date. We have implemented two versions of our method, one using CoMOGrad and the other combining CoMOGrad with PHOG. We have also implemented the latter version without scaling the alpha carbon distance matrices. Our methods are readily available for use at: http://research.buet.ac.bd:8080/Comograd/. The source code of our implementation can be found at: https://github.com/rezaulnkarim/protein_tertiary_structure_retrieval-.

### Benchmark Datasets

For our experiments, we have used *SCOP*^33^,^34^ domains classification. SCOP is well accepted as the benchmark in the literature. We have taken 152,487 chains from *SCOP* domain as the search space and extracted features from them. As the query proteins, we have used 4965 protein chains from 417 *SCOP* family, 330 superfamily, 234 folds and 11 classes. We have run all the queries over the search space using our methods and sorted the results in ascending order based on our distance measure. We also have run MASASW^21^ on the same settings and sorted the results in descending order based on its similarity measure. Then we compare the results considering the top four *SCOP* labels, namely, *class, fold, superfamily* and *family*. Lists of the query sequences as well as the search space sequences are provided as supplementary files.

### Accuracy Comparison

When a query protein is searched, the system (usually) returns a number of top ranked results. Suppose, we are searching Protein 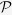. Let us denote 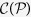, 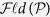, 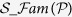 and 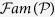 to denote the class, fold, superfamily and family of 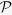 respectively. And suppose that the set of top ranked results returned by the system is 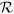. For each protein 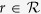 we can check its SCOP label. The accuracy of a system is measured by checking how many of the proteins in 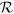 (i.e., the returned top-ranked results) match the SCOP label of the query protein 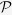. And the accuracy is measured in percentage. More formally, we have Equations (2, 3, 4, 5) below for measuring accuracy.


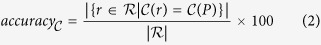



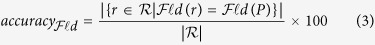



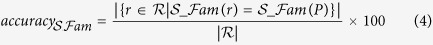



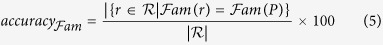


The accuracy defined here is similar to the term precision used in statistics and machine learning. However, we prefer to use the term accuracy. In our experiments we measure average accuracy by taking the average of the numbers of a SCOP label match from multiple queries (in fact, 4965 queries) and using it in the numerator of Equations (2, 3, 4, 5). As an example, suppose, we have run a total of three queries and considered the top 50 results of each query. Assume that, for the three queries the numbers of match for the label *family* are 40, 42 and 48 respectively. Then the average number of family match is (40 + 42 + 48)/3 = 45 and the percentage of family match is (45 × 100)/(50) = 90 percent.

**Figure 4:**
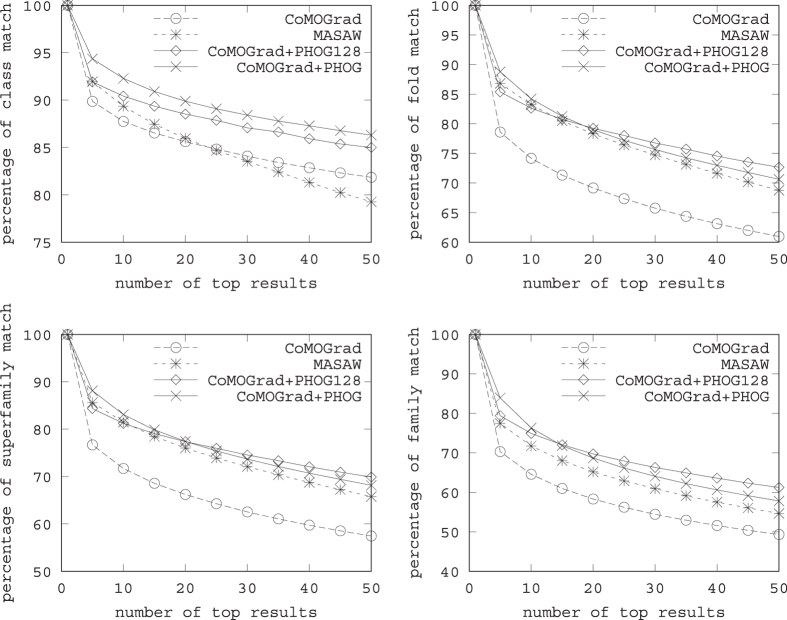
Percentage of matches of Class, Fold, Superfamily and Family for up to top 50 retrieval results.

The comparison of the accuracy of the query results is provided in Fig. 4. In particular, Fig. 4 presents four line graphs one each for each SCOP label. The horizontal axis entitled “*number of top results*” in each line graph, gives the number of top ranked query results considered whereas the vertical axis entitled “*% of ‘label’ match”* gives the average number of query results that have matched the *SCOP* ‘label’ with the corresponding query protein. So, each point in a graph reports the average number of query results having the same *SCOP* ‘label’ as the corresponding query protein for a specific number of top results. Each line graph reports results for 4 methods: the method of 21 (MASASW), our method with the CoMOGrad feature only (CoMOG), and both the scaled and non-scaled version of our method combining CoMOGrad with PHOG (CoMOG + PHOG 128 and CoMOG + PHOG). We report the results for all the *SCOP* labels, i.e., *class, fold, family* and *superfamily* in the line graphs presented in Fig. 3 starting from the leftmost one and going in clockwise direction respectively and each graph considers top 5 to top 50 retrieval results.

### Runtime Comparison

The time required to retrieve the results for all three methods are reported in Table 1. The run time is recorded by executing 100 queries for each of the methods. In the table, *Loading Time* is the time needed to load the feature vectors from disk to memory which is done once when the system starts. The *Time Per Query* in the table is the time needed to compare the feature vector of a query protein with that of all the 152,487 proteins in our protein database and sort the results based on the distance/similarity measure and to return the sorted top ranked results. Note that the query time reported excludes the loading time. The *Query Time* in the table indicates the total time needed for 100 query structures. The results indicate that the query time for the variant with only the CoMOGrad feature is ultra fast albeit at the cost of some reduction in the accuracy as is evident from Fig. 4. However, the variant using both CoMOGrad and PHOG as features is both super fast and more accurate than *MASASW*.

### Quality measure

To measure the quality of our distance measure, we have conducted a separate set of experiment as follows. We have examined the performance of a binary classifier designed with our feature sets. In particular, we have evaluated the Matthews correlation coefficient (MCC) values with the binary classifier partition at various distances. The Matthews correlation coefficient is a measure of the quality of binary classifications^35^. It takes true and false positives and negatives into account and is generally regarded as a balanced measure which can be used even if the classes are of very different sizes. The MCC is in essence a correlation coefficient between the observed and predicted binary classifications; it returns a value between −1 and +1. A coefficient of +1 indicates a perfect prediction, 0 indicates no better than random prediction and −1 indicates total disagreement between prediction and observation.

**Figure 5:**
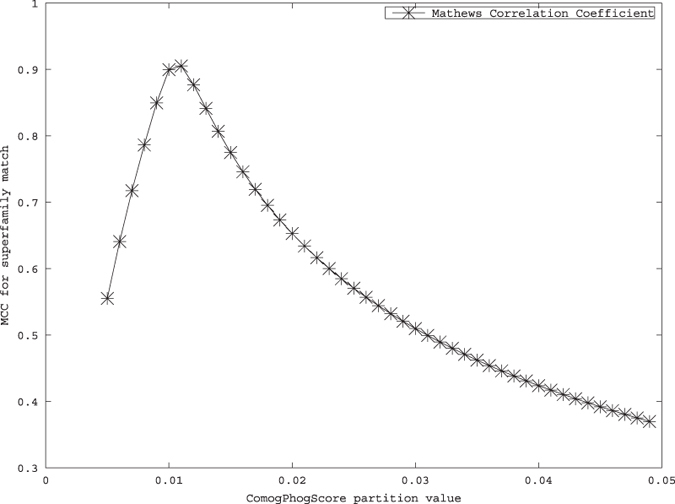
MCC values for binary classification.

Plotting MCC for binary classification with discrimination at various distance values shows that the MCC graph is convex which indicates that this measure is good for binary classifiers. And MCC have highest value at distance 0.011 which indicates that binary classification at distance 0.011 will give the best classification. Also MCC value there is nearly 0.9 which is very good. From this observation it is clearly evident that the distance measure is an effective one (See Fig. 5).

## Discussion

The exact algorithm for the matching of tertiary structures with *α* carbon distance matrix runs in *O*(*N*!) time assuming that the input matrix is of size *N* × *N*. The time complexity of *MASASW* for comparing two features is *O*(*wWN*^2^), which assumes *N* = 32 as the dimension of the distance matrix. Here, *W* and *w* are the size of the sliding windows to align matrices and to align rows, respectively. The authors of MASASW have empirically obtained the most reasonable and suitable values for *W* and *w* which are 5 and 8 respectively. Our CoMOGrad feature is a vector of size 256 and the combination of CoMOGrad and PHOG gives us a feature vector of size of 1021. For both of our methods, the run time for comparing two features is just *O*(*N*) where N is the length of feature vector. Therefore, when only the CoMOGrad feature is used, the time to compare two features is approximately (32 × 32 × 5 × 8)/(256) = 160 times faster than *MASASW*. And, the combination of CoMOGrad and PHOG is approximately (32 × 32 × 5 × 8)/(1021) = 40 times faster than *MASASW* in this respect. The feature extraction of the query protein as and when it is submitted as a coordinate file in *PDB* format does not have noticeable effect on the running time as this operation is done for one, i.e., the query protein only; the features of all the proteins in the target search space (i.e., in the database) are made available beforehand as they have already been preprocessed.

As can be see from Table 1, compared to *MASASW*, the query time of the variant with CoMOGrad is almost 30 times and the variant with CoMOGrad and PHOG is more than 7 times faster. As mentioned in our earlier discussion, theoretically the variant with CoMOGrad is 160 times and the variant with CoMOGrad and PHOG is 40 times faster than MASASW in comparing two features. The apparent discrepancy between the theory and practice with respect to running time can be attributed to the fact that, in addition to the feature comparison, the actual retrieval algorithm needs to perform a sorting operation on the results of the distance/similarity values of all the proteins in the database against the query protein. Note that both MASASW and our two methods need to use this sorting algorithm. MASASW however sorts in descending order because it uses a similarity measure. As a result the actual improvement in the query time achieved by our methods does not completely match with the theoretical deduction.

In terms of accuracy, the performance of our method using only the CoMOGrad feature is almost similar to that of *MASASW*. On the other hand, the combination of CoMOGrad and PHOG, both with or without scaling the alpha carbon distance matrix images, achieves a higher degree of accuracy. We notice that for the class label, the non-scaled version shows better performance than the scaled version. However, for the other three SCOP labels, the non-scaled version performs better up to a cutoff point (around 20) after which the scaled version seems to be superior. Note that in most practical scenarios, only the top few results of the returned result set are of interest. Hence, from the results reported in Fig. 4, it seems that the non-scaled version should be used for those scenarios. The experimental dataset used comprises proteins taken from 417 various families and the number is significant. We believe our most significant achievement is the substantial reduction of the query processing time which gives us the ability to offer a real-time web experience and that too without compromising the accuracy at all.

### Comparison with other methods

In our experiments, we have only compared our methods with MASASW which to the best of our knowledge is the best algorithm in the literature to date. Another candidate for comparison perhaps is ProteinDBS v2.0, the successor of the celebrated web server ProteinDBS. However, as it turns out ProteinDBS v2.0 (and also its predecessor) is a complex system and hence it is not possible to simply package the codes for comparison under the current implementation.In the absence of a running interface or any implementation we are not able to include ProteinDBS v2.0 in our experiments. Since direct comparison through experiments are not possible, we here make an effort to do an indirect comparison as follows. While presenting ProteinDBS in^15^ the authors have claimed that it is 10.87 times faster than CE. Now, as has been reported in^21^, MASASW is more accurate and much more faster than CE. On the other hand our experimental results show that the query time of CoMOGrad is 30.58 times and the combination of CoMOGrad and PHOG is 7.64 times faster than that of *MASASW*. This suggests that our methods are faster than ProteinDBS v2.0. Nevertheless, it would be interesting to conduct a direct comparison with ProteinDBS v2.0 or a later version on a level-playing ground. This seems possible in near future when the implementation of a parallelized version of ProteinDBS series in Hadoop is expected to be released.

Another candidate for comparison is the geometric hashing method used in^25^,^26^,^27^. However as will be clear after the discussion below, this method doesn’t seem to scale well for large search spaces. In what follows, we will denote the number of atoms by *n*, that of alpha carbons by *m* and that of chains in the database by *N*. First of all, geometric hashing technique first requires to examine all the *O*(*m*^3^) triplets for a protein with *m* alpha carbons, which incurs huge computational cost for large proteins. Then, before the actual comparison is done, a hash table is prepared to contain all the selected triplets with the sides of the corresponding triangles as keys to the hash table. Then, triplets are selected from the hash table one after another using appropriate filtering constraints. For each selected triplet, an orthogonal system represented by the 3 atoms as basis is computed. Then the coordinates of other *m* − 3 atoms are transformed to that orthogonal system and the new coordinates are compared to the alpha carbon coordinates of the target structure to compute the score. This process is repeated for all the triplets to optimize the score. These iterations also incur huge computational cost. Clearly, our methods require less computing in preprocessing/learning as well as in the comparison step. Additionally, our methods also do not require any complex data structure like nonlinear hash table required by the geometric hashing technique. Informatively, the matching and comparison stage of geometric hashing is also computation and memory intensive procedure as it employs a voting technique requiring a lot of memory for the voting entries.

## Conclusion

In this paper, we have presented two novel features, namely, CoMOGrad and PHOG, for faster and accurate retrieval of the protein tertiary structures. We have compared our results considering all the levels of *SCOP* classification hierarchy. We have reported average percentage of matching for class, fold, super family and family of our retrieval results with the query protein while most of the works in the literature have only shown similarity on only class and fold; very few in fact have worked on automated similarity match for the lower levels. Our results are in good compliance with the *SCOP* classification. CoMOGrad feature is ultra fast as compared to the state of the art methods but this extreme speed is achieved at the cost of a slight reduction in the accuracy. The combination of CoMOGrad with PHOG is also very fast and at the same time is superior in terms of accuracy as compared to state of the art methods. This creates the opportunity to implement a web based service for the protein tertiary structure retrieval with a truly online behaviour, i.e., a web-server that can provide the results in seconds, while the present web services usually provide query results via email only.

## Additional Information

**How to cite this article**: Karim, R. *et al*. CoMOGrad and PHOG: From Computer Vision to Fast and Accurate Protein Tertiary Structure Retrieval. *Sci. Rep*. **5**, 13275; doi: 10.1038/srep13275 (2015).

## Supplementary Material

Supplementary Information

Supplementary Information

## Figures and Tables

**Table 1:** Comparison of query time.

Method	Loading Time	Query Time	Time Per Query
MASASW	28 min 11 s	42 min 18 s	25.38 s.
CoMOGrad	18 m 31 s	1 m 23	0.83 s
CoMOGrad + PHOG	27 min 24 s	5 min 32 s	3.32 s.
