# Supplementary material for: CoMOGrad and PHOG: From Computer Vision to Fast and Accurate Protein Tertiary Structure Retrieval
Source: Sci Rep. 2015 Aug 21;5:13275. doi: 10.1038/srep13275 (PMC4543952; doi:10.1038/srep13275)
Supplement: Supplementary Information [file srep13275-s1.doc]

CoMOGrad and PHOG: From Computer Vision to Fast and Accurate Protein Tertiary Structure Retrieval

Rezaul Karim, Mohd. Momin Al Aziz, Swakkhar Shatabda, M. Sohel Rahman, Md. Abul Kashem Mia, Farhana Zaman and Salman Rakin

* Supplementary Table 1

Id of the structures used in the experiments as query structure:

d1cexa_

d1g66a_

d1agya_

d1cuaa_

d1cuba_

d3c70a_

d1dwoa_

d1dwob_

d1e89a_

d1e89b_

d3efza1

d3iqua_

d3efzc_

d2npma_

d2npmb_

d1f0la2

d1giqa1

d1ikpa2

d2a5db1

d2zitb1

d1m22a_

d2gi3a1

d1m21a_

d1o9na_

d1o9pa_

d2gl7a_

d2gl7d_

d4ev8a_

d4evaa_

d4evac_

d2bfda1

d2bfba1

d2bfca1

d2bfea1

d2bffa1

d1uzva_

d1uzvb_

d1uzvc_

d1uzvd_

d1oxca_

d2h12a_

d2ifca_

d1amza_

d1csia_

d1csra_

d2gcia_

d1p5ha_

d1pt7a_

d1pt8a_

d1q7ea_

d1b6aa2

d1chma2

d2v3xa2

d2v3ya2

d2v3za2

d1q3ka_

d1v7za_

d1v7zb_

d1v7zc_

d1v7zd_

d4gpha_

d4gphb_

d4gphc_

d4goha_

d4gpca_

d1u6ka_

d1u6kb_

d1u6kc_

d1qv9a_

d1qv9b_

d1a4ha_

d1ah8a_

d1ah8b_

d1byqa_

d1osfa_

d3gtda_

d1re5b_

d1re5c_

d1re5d_

d1dofb_

d2jnpa1

d2k2ga1

d2z2da1

d1ayka_

d1b8ya_

d1hr6a1

d1hr6a2

d1hr6b2

d2fgea1

d2fgea2

d1bo4a_

d1cjwa_

d1ghea_

d1i12a_

d1m4ia_

d1t6ub_

d1t6uc_

d1t6ue_

d1t6ug_

d1t6uh_

d1ii2a2

d1j3ba2

d1khba2

d2pc9c2

d2pxzx2

d1eysh2

d1l9bh2

d1psth2

d1rg5h2

d1rzhh2

d1hwma_

d1m2ta_

d1ahca_

d1apaa_

d1br6a_

d1vq821

d1vqp21

d1yhq21

d3cc221

d1s722_

d2qalm1

d2qbfm1

d2qbdm1

d2qoym1

d2qp0m1

d2gy9b1

d2vqeb1

d1fjgb_

d1i94b_

d1vi5a_

d1a34a_

d1stma_

d1stmb_

d1stmc_

d1stmd_

d1a7ta_

d1jjta_

d1k07a_

d1ko3a_

d1m2xa_

d1l6ra_

d2b30a1

d2fuea_

d2hf2a_

d1rlma_

d1io7a_

d1jfba_

d1bu7a_

d1bvya_

d1bvyb_

d1c3ca_

d1dofa_

d1aosa_

d1auwa_

d1dcna_

d1a9xa5

d1a9xa6

d1dv1a3

d1gsoa3

d2j9ga3

d1clca1

d1ia6a_

d1ks8a_

d1cema_

d1ksda_

d1k6da_

d2ahua2

d2g39a1

d2g39a2

d2nrba2

d2ab0a1

d2fexa1

d3l18a_

d1izya_

d1izza_

d1b77a1

d1b77a2

d1dmla1

d1dmla2

d1iz5a1

d1a8qa_

d1brta_

d1broa_

d1va4a_

d1va4b_

d1w6ja2

d2sqcb1

d2sqcb2

d1umpa2

d1umpb1

d1g4ia_

d1le6a_

d1mc2a_

d1a2aa_

d1a3da_

d1p80a1

d1sy7a1

d1p80b1

d1p80c1

d1p80d1

d2ahua1

d2nrca1

d1m3eb2

d1ooya1

d1ooyb1

d2f9wa1

d2f9wa2

d3bexa1

d3bexa2

d2f9tb1

d1ezia_

d1h7ea_

d1i52a_

d1jyka_

d1eyra_

d1fgja_

d1h21a_

d3bnja_

d2rf7d_

d3tora_

d1beda_

d3l9sa_

d1a23a_

d1a2la_

d1ac1a_

d1a1va1

d1a1va2

d2bmfa1

d2bmfa2

d1cu1a2

d1lo7a_

d2cyea1

d2fuja1

d2gf6a1

d2gvha1

d2a14a1

d3kqsa_

d1hnna_

d1hnnb_

d2g8na_

d1aw5a_

d1b4ea_

d1b4ka_

d1b4kb_

d1e51a_

d1llfa_

d1clea_

d1crla_

d1gt6a_

d1gz7a_

d1ispa_

d2dsna_

d1cvla_

d1ex9a_

d1i6wb_

d1gwua_

d1a2fa_

d1a2ga_

d1apxa_

d1b80a_

d1j30a_

d1jiga_

d1lb3a_

d1lkoa1

d2fjca1

d1aw9a1

d1duga1

d1e6ba1

d1f2ea1

d1gnwa1

d1bd0a2

d1hkva2

d1knwa2

d1d7ka2

d1f3ta2

d3bvua3

d2ow6a3

d1qx1a3

d3ejqa1

d2ow7a3

d3bvua1

d3dx1a2

d3ejra2

d3ejua2

d3ejpa2

d1ecsa_

d1klla_

d2i7ra1

d1byla_

d1ewja_

d1atia1

d1g5ha1

d1h4vb1

d1hc7a1

d1kmma1

d1e4cp_

d1k0wa_

d1dzup_

d1dzvp_

d1dzwp_

d4fyxa1

d4fyxa2

d4fyxc1

d4fyxc2

d4fyya1

d1cg2a1

d1fnoa4

d1lfwa1

d2fvga2

d2grea2

d1dv1a1

d1gsoa1

d2j9ga1

d1dv1b1

d1dv2a1

d1ddba_

d2ponb1

d1af3a_

d1bxla_

d1g5ja_

d2fy7a_

d1fgxa_

d1fgxb_

d1fr8a_

d1nf5b_

d1ddza2

d1ekja_

d1g5ca_

d1ddza1

d1ekjb_

d2bfdb2

d1ik6a2

d1umdb2

d2ozlb2

d1qs0b2

d2ingx2

d1l0ba1

d1l0ba2

d1n5ox2

d1oqaa_

d1buoa_

d1hv2a_

d1lm8c_

d1cs3a_

d1fs1b2

d1dr9a2

d1iama1

d2d9qb1

d2oz4a1

d1cdya2

d1edha2

d1l3wa3

d1l3wa4

d1l3wa5

d2omwb1

d2bgca1

d2gaua1

d1hw5a1

d1hw5b1

d1i5za1

d1dpba_

d3claa_

d1b5sa_

d1c4ta_

d1c4tb_

d2ccaa2

d2dv1a1

d3n3ra1

d3n3ra2

d3n3rb1

d2d4za3

d2ef7a1

d2j9la1

d3ddja1

d3ddja2

d1aoha_

d1g87a2

d3bwza_

d1anua_

d1aohb_

d1ndba1

d1ndba2

d1nm8a1

d1nm8a2

d1q6xa1

d1y1ki1

d1y33i1

d1y34i1

d1y3bi1

d1y3ci1

d2j7ja1

d2j7ja2

d2j7ja3

d1a1ga1

d1a1ha1

d1w63a_

d1w63b_

d1w63c_

d1w63d_

d1w63e_

d1jrob1

d3b9jc1

d1dgja3

d1ffvb1

d1n60b1

d1bkva_

d1bkvb_

d1bkvc_

d1q7da_

d1q7db_

d2qzfa1

d1c1za5

d1g40a1

d1g40a2

d1g40a3

d1bd7a_

d1h4ax1

d1h4ax2

d1hdfa_

d1a45a1

d3gaxa_

d1a90a_

d1cewi_

d1cyua_

d1g96a_

d1alna1

d1alna2

d2fr5a_

d1jtka_

d1mq0a_

d1c0pa2

d2gf3a2

d1kifa2

d1l9ca2

d1ng4a2

d1w79a1

d1w79b1

d2ex2a1

d2y4ad_

d2y4aa_

d2a8na1

d2g84a1

d2hxva2

d1ox7a_

d1p6oa_

d1jm0a_

d1jm0b_

d1jm0c_

d1jm0d_

d1jm0e_

d1eyea_

d1ad1a_

d1ad1b_

d1ad4a_

d1ajza_

d1ih7a2

d2hhva2

d2p5gb2

d2p5gc2

d2vwja2

d1ep0a_

d1dzra_

d1nxma_

d1oi6a_

d1oi6b_

d1d5ra2

d1fpza_

d1i9sa_

d3f81a_

d1fpzc_

d2akza1

d2ptza1

d1e9ic1

d1iyxa1

d1oepa1

d1ejda_

d1g6sa_

d1a2na_

d1dlga_

d1epsa_

d2ba0a3

d2bl5a1

d2ctea1

d2ctfa1

d2ctja1

d1d7ya3

d1feca3

d1h6va3

d1m6ia3

d3grsa3

d2h1va_

d3hcna_

d1ak1a_

d1c1ha_

d1l8xa_

d1fmta2

d1jkxa_

d1meoa_

d2blna2

d2bw0a2

d1krua_

d2ic7a_

d3dhoa_

d3eevc_

d1khra_

d1cf2o2

d2g17a2

d2g82a2

d2gz1a2

d2pkqo2

d1d2ga_

d1d2ha_

d1kiaa_

d1nbia_

d1r74a_

d1gpea2

d1ju2a2

d1kdga2

d2f5va2

d3b3ra2

d1a8ra_

d1a9ca_

d1fb1a_

d1gtpa_

d1n3re_

d1b6ga_

d1mj5a_

d1beza_

d1bn7a_

d1cqwa_

d1ehda1

d1ehda2

d1eisa1

d1eisa2

d1en2a1

d1czan1

d1czan2

d1ig8a1

d1ig8a2

d1bdga1

d1c3pa_

d3ew8a_

d1c3ra_

d1t64a_

d1vkga_

d1fita_

d3imia_

d1kpcd_

d1kpea_

d1kpfa_

d2gykb_

d2jazb1

d2jbgb1

d1emvb_

d1fr2b_

d1gmea_

d1gmeb_

d1gmed_

d1shsa_

d2h50a1

d1em7a_

d1fccc_

d1fcla_

d1fd6a_

d1gb4a_

d3sdya_

d4h32i_

d4h32g_

d4gxxe_

d4f23a1

d1i3oe_

d1jd5a_

d3d9ta1

d2raxa1

d3d9ua1

d1ka1a_

d1lbva_

d1bk4a_

d1cnqa_

d1d9qa_

d1c3za_

d3cz1a_

d1qwva_

d1twoa1

d1dqeb_

d2dtra1

d1g3sa1

d1bi1a1

d1bi0a1

d1p92a1

d2ev6a2

d2ev6b2

d2ev5a2

d2ev5b2

d2f5fb2

d3bxqb1

d2olzb1

d2olzd1

d2olzf1

d2olzh1

d1f52a1

d1f52b1

d1f52c1

d1f52d1

d1f52e1

d1a0pa1

d3mgva1

d3mgvb1

d3mgvc1

d3mgvd1

d1a7ia1

d1a7ia2

d1b8ta1

d1b8ta2

d1b8ta3

d1gq2a2

d1gz3a2

d1gz4a2

d1o0sa2

d1pj3a2

d1c3ma_

d2guda1

d1c3ka_

d1c3na_

d1j4ta_

d1iq6a_

d2bi0a1

d2bi0a2

d2c2ia1

d3k67a_

d1hsja1

d1jgsa_

d2a61a1

d2bv6a1

d2etha1

d1czya1

d1lb6a_

d3ivva_

d1d00a1

d1lb4a_

d1ivha1

d2c12a1

d2d29a1

d2c12b1

d2c12c1

d1di6a_

d1mkza_

d2g2ca1

d1eava_

d1mkzb_

d1o1a7_

d1m8qv_

d1o192_

d1o196_

d1o198_

d1dnu.1

d1d7w.1

d3kk6b2

d2oyep1

d3kk6a2

d1ct9a1

d1gpma1

d1jgta1

d1k92a1

d1kqpa_

d1d7aa_

d1o4va_

d1qcza_

d1u11a_

d1u11b_

d1imhc1

d2cxka1

d3brda1

d2i9tb1

d1zk9a_

d1mioa_

d1miob_

d1fp4a_

d1fp4c_

d1l5ha_

d2masa_

d3epwa_

d1hoza_

d1hozb_

d1kica_

d2a84a_

d1ihoa_

d1mopa_

d1mopb_

d1n2ga_

d1fmja_

d1j99a_

d1aqua_

d1aqub_

d1bo6a_

d1dcsa_

d1gp6a_

d1e5ha_

d1e5ia_

d1gp4a_

d1khba1

d2pc9a1

d1ii2a1

d1j3ba1

d1j3bb1

d2glga1

d2glha1

d1bkua_

d1bzba_

d1byva_

d3bwuc2

d1l4ia2

d1l4ib2

d1p5ua2

d1p5va2

d1cyxa_

d2cuaa_

d2dyrb1

d3dtub1

d3ehbb1

d1dqua_

d3lg3a_

d1f61a_

d1f8ia_

d1f8ma_

d1bf6a_

d1dpma_

d1eywa_

d1ez2a_

d1i0da_

d4b0mm1

d3dpba1

d3dsna1

d3dsnd1

d3dosa1

d1d5ra1

d1e7ua2

d1gmia_

d2cjta1

d2ep6a1

d1i17a_

d1ag2a_

d1b10a_

d1dwya_

d1e1ga_

d2cz4a1

d2ns1b1

d1gnka_

d1hwua_

d1hwub_

d1egaa2

d2qalc1

d1e3ha4

d1e3pa5

d1egab2

d3k40a_

d1pmma_

d1pmmc_

d1pmoa_

d1pmob_

d1iu8a_

d1a2za_

d1auga_

d1iofa_

d1ioia_

d1e7ua3

d1k8rb_

d1lfda_

d2byea1

d2c5lc1

d1a8pa2

d1fdra2

d2bmwa2

d1b2ra2

d1bjka2

d1a3qa2

d1bvoa_

d2o61b2

d1a02n2

d1a66a_

d1az3a_

d1az3b_

d1az4a_

d1az4b_

d1b96a_

d4flga_

d4flgb_

d3o9ba_

d3o9ea_

d3nlsa_

d4j93a_

d4e92a_

d4e92b_

d4inba_

d4lqwc_

d1bx4a_

d2absa1

d2afba_

d2ajra_

d2fv7a1

d1xbpg2

d2gycg2

d2qami2

d3cjqb2

d3cjrb2

d2pa2a1

d1ffkf_

d1jj2h_

d1s72h_

d1vqoh1

d1j3aa_

d2j01n1

d2gych1

d2hgqm1

d2zjrg1

d1vq8p1

d1vqpp1

d1yhqp1

d3cc2p1

d1s72p_

d2aarw1

d2hgq11

d2qamx1

d2zjrv1

d1r73a_

d1ffkv_

d1vqoy1

d1vq8y1

d1vqpy1

d1yhqy1

d1dmga_

d2j01f1

d2gycc1

d2hgqf1

d2qame1

d3bn0a1

d2gy9p1

d2qalp1

d2qanp1

d2vhpp1

d2qalr1

d2vqer1

d1g1xc_

d1i94r_

d2uubr1

d2gy9s1

d1fjgs_

d1qkha_

d2j00s1

d2uubs1

d2gy9t1

d2qalt1

d2vqet1

d2uubt1

d2qant1

d2qalc2

d2uubc2

d2qanc2

d2qbfc2

d2qbdc2

d2qang1

d2qbfg1

d2qbdg1

d2qoyg1

d2qp0g1

d2qale2

d1i94e2

d1pkpa2

d2uube2

d2qane2

d1voy7_

d1vp07_

d1ibmc_

d1iblc_

d1s1id_

d2d69a2

d2v69c2

d2v6ad2

d1aa1b2

d1ausl2

d2b1xb1

d2bmob1

d3e99a1

d3ebya1

d3gzyb_

d2dg1a_

d2ghsa1

d1e1aa_

d1pjxa_

d2dsoa1

d1hula_

d1jlia_

d2d48a_

d3ejja_

d3l5xa_

d1m2ka_

d1icia_

d1icib_

d1j8fa_

d1j8fc_

d1chua2

d1jnra2

d1kf6a2

d2bs2a2

d1d4ca2

d1eucb1

d2csua2

d2csua3

d2nu6a2

d2nu7a2

d4j7da_

d4j74a_

d4j7ea_

d4ipfa_

d4j3ea_

d3c14c2

d3c15c2

d3c16c2

d3ab3a2

d3ab3c2

d1ex0a2

d1ex0a3

d1g0da2

d1g0da3

d1l9na2

d1qloa_

d3mraa_

d1kdla_

d1xnla_

d1bhaa_

d1f86a_

d1bz8a_

d1bzda_

d1bzea_

d1dvua_

d2rieb2

d3ikna1

d3iknb1

d3iknc1

d3ikra1

d1iq8a1

d3blda_

d1efza_

d1j2ba2

d1ozqa_

d1dlwa_

d2bkma_

d2gkma_

d1dlya_

d1idra_

d2btoa2

d1z2ba2

d1z2bb2

d2vapa2

d2vawa2

d3e46a1

d1dv0a_

d1f4ia_

d1ifya_

d1oqya1

d1j2ga1

d1j2ga2

d2ibaa1

d2ibaa2

d2yzbe2

d1j5sa_

d1j5sb_

d2pnka1

d3hk9f_

d3hk9j_

d1erja_

d1gxra_

d1k8kc_

d1a0rb_

d1gg2b_

d2jmfa1

d1f8ab1

d1i5hw_

d1i8gb_

d1jmqa_

d3mkva2

d3mkvb2

d3mkvc2

d3mkvd2

d3mkve2

d1egia_

d1h8ua_

d1hq8a_

d1j34a_

d1jwib_

d1jzna_

d3c8ja1

d1afb11

d1bch11

d1bcj11

d1a9xb2

d1gpma2

d1i7qb_

d1jvna2

d1k9vf_

d1ka9h_

d1l9xa_

d2abwa1

d1c30b2

d1cs0b2

d12asa_

d1atia2

d1b8aa2

d1e1oa2

d1eova2

d1g5ha2

d1h4vb2

d1hc7a2

d1j5wa_

d1jjca_

d4klia2

d4klga2

d4klma2

d4klja2

d4klla2

d4kloa2

d4klfa2

d3rh4a2

d4klda2

d4klea2

d1xcka1

d1xckb1

d1xckc1

d1xckd1

d1xcke1

d1xckf1

d1xckg1

d1xckh1

d1xcki1

d1xckj1

d1m7va_

d1d0ca_

d1d0cb_

d1dd7a_

d1jwja_

d1jwka_

d1m7za_

d1m9kb_

d1m9ma_

d1mjta_

d1cn3a_

d1sida_

d1sidb_

d1sidc_

d1sidd_

d1sidf_

d1sva1_

d1sva3_

d1sva4_

d1sva5_

d2ieja_

d3e33a_

d3e34a_

d3ksla_

d3ksqa_

d4gtma_

d4gtoa_

d4gtra_

d3e30a_

d2zira_

d2d6ya2

d2fbqa2

d2fd5a2

d2fq4a2

d2fx0a2

d2g3ba2

d2g7ga2

d2g7la2

d2g7sa2

d2gena2

d1fcya_

d1g2na_

d2aclb1

d3d24a1

d1a52a_

d1a52b_

d1dkfa_

d1errb_

d1exaa_

d1fbya_

d1b0ua_

d1f2t.1

d1g6ha_

d1ji0a_

d2ixea_

d3dhwc1

d2r6ga2

d1e69a_

d1ewqa2

d1ewqb2

d2bcqa3

d2fmpa3

d2vana2

d3c5fa3

d3c5ga3

d1huza4

d1jn3a2

d1rpla2

d1xsna3

d1zjna3

d1jq4a_

d1kf6b2

d1krha3

d2bs2b2

d3c8ya2

d1dgja2

d1ffva2

d1ffvd2

d1fiqa2

d1n62a2

d1b9ra_

d1czpa_

d1doia_

d1l5pa_

d2bt6a_

d1a70a_

d1awda_

d1ayfa_

d1cjea_

d1cjeb_

d1isia_

d3f6ya_

d3i9la_

d2ef1a1

d1lbea_

d1lbeb_

d1r0sa_

d1r12a_

d2o3ra_

d3i9ma_

d3dssb_

d3dsub_

d3hxdb_

d3dsxb_

d1s64h_

d2f0yb_

d1n4ph_

d1n4sh_

d1tnoh_

d3e37b_

d2bgra2

d1orva2

d1u8ea2

d1u8eb2

d2g63a2

d2oncb2

d1orwb2

d1r9nd2

d1orvd2

d2iivb2

d1l7aa_

d1odsa_

d1odtc_

d3fyth_

d3fvtf_

d1vlqa_

d1vlqb_

d1vlqc_

d1vlqd_

d1vlqe_

d1bg6a2

d1dlja2

d1evya2

d1f0ya2

d1i36a2

d1jaya_

d1ks9a2

d1lj8a4

d2ahra2

d2b0ja2

d1c4oa1

d1c4oa2

d1fuka_

d1gm5a3

d1gm5a4

d1hv8a1

d1hv8a2

d2b2na1

d2eyqa2

d2eyqa3

d1auib_

d1exra_

d1j7qa_

d1m45a_

d1y0vh1

d2dfsb1

d2dfsc1

d2hf5a1

d2jt0a1

d2ju0a1

d1zbbd1

d2f8nk1

d2jssa1

d2jssa2

d3b6fa1

d3c1ch1

d1aoic_

d1aoie_

d1eqza_

d1eqzb_

d1ej2a_

d1k4ma_

d1lw7a1

d1mrza2

d2h29a_

d3mlaa_

d1f9aa_

d1gn8a_

d1gzua_

d1h1ta_

d3mk1a_

d1ajba_

d1alha_

d1alia_

d1alka_

d1ania_

d1anja_

d1elxa_

d1elya_

d1elza_

d1g8oa_

d1lzia_

d1lzja_

d1o7oa_

d1o7qa_

d1vzxa_

d1wsza_

d1wt0a_

d1wt1a_

d3v0qb_

d1bgva2

d1c1da2

d1gtma2

d1leha2

d1aupa2

d1b26a2

d1b3ba2

d1bvua2

d1bxga2

d1bxgb2

d2fzcb1

d2ipob1

d2qg9b1

d2qgfb1

d1nbeb1

d1pg5b1

d1skub1

d2airb1

d2atcb1

d2h3eb1

d1a9xa3

d1dv1a2

d1gsoa2

d1kjqa2

d2j9ga2

d3etja2

d1a9xa4

d1b6sa2

d1c30a3

d1dv2b2

d2f9yb1

d1od2a2

d1od2b1

d1od4a1

d1on3a1

d1on3a2

d1on3b1

d1on3d1

d1on3e1

d1on9e1

d4asua3

d4asub3

d4asuc3

d2v7qd3

d2v7qe3

d2v7qf3

d2jizd3

d2jize3

d2jizf3

d2jizk3

d1cx4a1

d1cx4a2

d1i5za2

d2gaua2

d3bpza_

d3e5ua2

d1g6na2

d1hw5a2

d1i5zb2

d1i6xa2

d1cvja2

d1fj7a_

d1fjca_

d1fxla1

d1fxla2

d1l3ka1

d2adca1

d2adca2

d2b0ga1

d2cpfa1

d1m72a_

d2j32a_

d1f1ja_

d1f1jb_

d1gqfa_

d1i4oa_

d1i4ob_

d1jxqa_

d1jxqb_

d1k86a_

d1goia3

d1jnda2

d2dpea2

d2o9oa2

d2pi6a2

d1edqa3

d1guva2

d1ll7a2

d1owqa2

d1syta2

d1f7ua2

d1ffya3

d1h3fa1

d1h3na3

d1i6la_

d1ilea3

d1iq0a2

d1irxa2

d1ivsa4

d1j09a2

d1e58a_

d1h2ea_

d1e59a_

d1ebba_

d1fzta_

d1qhfa_

d1riia_

d1riib_

d1riic_

d1riid_

d2vt0b1

d1qw9a1

d1uhva1

d1w91a1

d2bs9a1

d2c7fa1

d2c7fb1

d2c7fc1

d2c7ff1

d2nt0a1

d1dcia_

d1ef8a_

d1hzda_

d1mj3a_

d2a7ka1

d2f6qa1

d2gtra_

d1dubb_

d1ey3a_

d1hnua_

d1c7na_

d1cl1a_

d1cs1a_

d1d2fa_

d1eg5a_

d1elua_

d1ibja_

d1iuga_

d1m32a_

d2bkwa1

d4jeaa_

d4jeab_

d4jeac_

d4jead_

d3tola_

d3told_

d4je9a_

d4je9b_

d3toma_

d3tomb_

d1ec7a1

d1jpdx1

d1jpma1

d1kkoa1

d1muca1

d2gdqa1

d2gl5a1

d2mnra1

d1bqga1

d1dtna1

d1hqsa_

d1a05a_

d1ai3a_

d1cm7a_

d1cnza_

d1cw4a_

d1cw7a_

d1dpza_

d1dpzb_

d1dr0a_

d1jmsa1

d4klia1

d4klga1

d4klma1

d4klja1

d4klla1

d4kloa1

d4klfa1

d3rh4a1

d4klda1

d1euwa_

d1f7da_

d2bsya1

d2bsya2

d3hzaa_

d1duca_

d1duna_

d1f7db_

d1f7oa_

d1f7oc_

d1bf2a1

d1clca2

d1h3ga1

d1j0ha1

d1k3ia1

d2bvya1

d2bvtb1

d3vgba1

d2fgza1

d2fgza2

d3bpse1

d3bt1a1

d3bt2a1

d1a3pa_

d1adxa_

d1autl1

d1autl2

d1bf9a_

d1c5mf_

d1ccfa_

d1cjxa1

d1cjxa2

d1f1ua1

d1f1ua2

d1kw3b1

d1kw3b2

d1mpya1

d1mpya2

d1eiqa2

d1f1ub1

d3d04a_

d1u1za_

d1u1zb_

d1u1zc_

d1z6ba1

d1zhga1

d2glla_

d2gllb_

d2gllc_

d2glld_

d1e4mm_

d1bgga_

d1cbga_

d1dwam_

d1e1eb_

d1e1fa_

d1e4ia_

d1e4na_

d1e55a_

d1e55b_

d1uerb1

d1uerc1

d1uerd1

d1uesa1

d1uesb1

d1uesc1

d1uesd1

d1qnna1

d1qnnb1

d1qnnc1

d1gtea5

d1h0hb_

d1hfel2

d1jnrb_

d1kqfb1

d2c42a5

d2fug34

d2fug91

d3c8ya3

d1y4zb1

d3bb2a_

d3bb2b_

d3b9ua_

d3baoa_

d3baob_

d2hwma_

d2hwmb_

d3ba7a_

d3ba7b_

d3bava_

d1fd9a_

d1hxva_

d1jnsa_

d1jvwa_

d1m5ya3

d1bkfa_

d1bl4a_

d1c9ha_

d1eq3a_

d1eyma_

d1bvyf_

d1e5da1

d1f4pa_

d1ag9a_

d1ahna_

d1akqa_

d1akra_

d1akta_

d1akwa_

d1azla_

d1dxya1

d1gdha1

d1l7da1

d1li4a1

d1b3ra1

d1hkua1

d1j49a1

d1j4aa1

d1ky5a1

d1mx3a1

d1i0va_

d3agna_

d1bira_

d1ch0a_

d1fysa_

d1fzua_

d1g02a_

d1hyfa_

d1i0xd_

d1i2ga_

d1dgw.1

d1dgwa_

d1lrha_

d2et1a_

d1caua_

d1caub_

d1fxza1

d1fxza2

d1ipja1

d1ipja2

d1fxoa_

d1fxob_

d1h5ra_

d1h5rb_

d1h5sa_

d1h5sb_

d1h5sd_

d1iina_

d1lvwa_

d1lvwb_

d2ocda1

d1agxa_

d1hg0a_

d1ho3a_

d1jaza_

d1jsra_

d1nnsa_

d1o7ja_

d1wsaa_

d1zq1a2

d3ezwa1

d3ezwa2

d1bu6o1

d1bu6y1

d1glag1

d1glag2

d1glfo1

d1glfx1

d1gljo1

d1r59o1

d1dk7a_

d1fy9a_

d1fyaa_

d1grla2

d1ioka2

d1kida_

d1kp8a2

d1la1a_

d1oela2

d1sjpa2

d1kp8a3

d1ioka3

d1j4za3

d1sjpa3

d1we3a3

d1xcka2

d1xckb2

d1xckc2

d1xckd2

d1xcke2

d1gwea_

d1m7sa_

d1a4ea_

d1cf9a2

d1dgfa_

d1dghb_

d1e93a_

d1f4ja_

d1f4jb_

d1f4jc_

d1g4us2

d1lara1

d1lara2

d1lyva_

d2f71a_

d1a5ya_

d1bzca_

d1c86a_

d1c88a_

d1eena_

d1mija_

d2cufa1

d1ahdp_

d1akha_

d1aplc_

d1b72b_

d1du0a_

d1du0b_

d1du6a_

d1e3oc1

d1atza_

d1ijba_

d1mf7a_

d1mjna_

d1u0oc1

d1ao3a_

d1aoxa_

d1aoxb_

d1atzb_

d1auqa_

d2a15a1

d1buqa_

d1c7ha_

d1cqsa_

d1dmma_

d1dmna_

d1dmqa_

d1e3rb_

d1e3va_

d1e3vb_

d1b5qa2

d2dw4a3

d2iida2

d2ivda2

d2v5za2

d1o5wa2

d1reoa2

d1seza2

d2xfna2

d2xfnb2

d1byka_

d1dbqa_

d1dp4a_

d1guda_

d1jdpa_

d1jx6a_

d1jyea_

d2fvya_

d2gx6a_

d3ckma1

d2j01x1

d1vs6t1

d1vsar1

d2qamt1

d2zjrq1

d1ffkp_

d1n88a_

d1vqos1

d3cf5q1

d2aarr1

d1ejba_

d1c2ya_

d1c41a_

d1di0a_

d1di0c_

d1di0d_

d1di0e_

d1kyva_

d1kyvc_

d1kyve_

d1f9va_

d1kk8a2

d1lkxa_

d2zfja1

d2zfka1

d1b7ta4

d1bg2a_

d1br1a2

d1br2a2

d1cz7a_

d1i9ba_

d1uv6a_

d1uw6a_

d1uw6b_

d1ux2a_

d1yi5a1

d1uw6c_

d1uw6d_

d1uw6e_

d1uw6f_

d1be4a_

d1bhna_

d1ehwa_

d1ehwb_

d1f3fa_

d1f6tb_

d1hhqa_

d1hlwa_

d1jxva_

d1k44a_

d3d06a_

d1gzha_

d1gzhc_

d1hu8a_

d1kzya_

d1tsra_

d1uola_

d1ycsa_

d2feja1

d2p52a_

d1ixla_

d2f41a1

d2fs2a1

d2hboa1

d3f5oa_

d1j1yb_

d1psua_

d1psub_

d1q4ta_

d1q4tb_

d1h8la2

d1jqga1

d1m4la_

d1ayea1

d1bava_

d1cbxa_

d1cpxa_

d1dtda_

d1ee3p_

d1kwma1

d4gjba_

d4gjca_

d4gjbb_

d4lxmc_

d4gj7b_

d4j1ka_

d4gjab_

d4gjdb_

d3vf3a_

d4djxa_

d2axwa1

d2cnza_

d2j2zb1

d4av5a_

d1z9sb1

d3crea1

d1kiub1

d1p5ub_

d1p5uc_

d1p5vb_

d2g50a2

d3e0wa2

d1a3wa2

d1a3xa2

d1a49a2

d1e0ta2

d1e0ua2

d1pkla2

d1pkma2

d1pkna2

d1a3wa3

d1e0ta3

d2g50a3

d3e0wa3

d1a3xa3

d1a49a3

d1f3wa3

d1f3xa3

d1pkla3

d1pkma3

d2ez9a3

d2ihta3

d2ji7a3

d2v3wa3

d1jsca3

d1jscb3

d1ozfa3

d1ozha3

d1ozhb3

d1ozhd3

d2ez9a2

d2ihta2

d2ji7a2

d1jsca2

d1jscb2

d1ozfb2

d1ozga2

d1ozha2

d1ozhc2

d1pi3a2

d2ez9a1

d2ihta1

d2ji7a1

d1jsca1

d1jscb1

d1n0ha1

d1n0hb1

d1ozha1

d1ozhc1

d1poxa1

d1d4aa_

d1dxqa_

d1h69a_

d1kbqa_

d1kbqb_

d1qbga_

d1qrda_

d1t5ba_

d1tika_

d1h66a_

d1cxqa_

d1exqa_

d1asua_

d1aswa_

d1b92a_

d1b9fa_

d1bhla_

d1bi4b_

d1bisa_

d1biza_

d1e3ha2

d1e3ha3

d2je6a1

d2je6b1

d1oypa1

d1oysa1

d1r6la1

d1udna1

d1udoa1

d1udqa1

d1xbpg1

d1y39a1

d1y39b1

d2gycg1

d2qami1

d2zjqf1

d3cc2i1

d3cjrb1

d3cjtb1

d1foxa_

d2j01s1

d1vs6o1

d2gycm1

d2qalk1

d2qamo1

d2vqek1

d2zjrl1

d1i94k_

d1ilya_

d1ovya_

d1i4ja_

d1yj9r1

d2gycq1

d2j01w1

d2qams1

d2zjrp1

d1bxea_

d1vqor1

d3cf5p1

d2zjqp1

d2j01p1

d2hgqo1

d2qaml1

d2zjri1

d1ffkj_

d1vqol1

d1vs6l1

d2gycj1

d3cf5i1

d2zjqi1

d1khva_

d2pgga1

d1c2pa_

d1c2pb_

d1csja_

d1gx5a_

d1gx6a_

d1nb4a_

d1nb4b_

d1nhua_

d1imva_

d1jmja_

d1lj5a_

d2arra_

d2b4xi1

d2d26a1

d2gd4c1

d2gd4i1

d2hiji1

d3evji1

d1ayaa_

d1d4ta_

d1lkka_

d1mila_

d3buxb3

d1y57a2

d1a09b_

d1a1aa_

d1a1ab_

d1a1bb_

d1ac0a_

d3bmva2

d1cgta2

d1cxla2

d1cyga2

d1ot1a2

d1pama2

d1peza2

d1qhoa2

d1tcma2

d1enfa2

d1esfa2

d1et9a2

d1eu3a2

d1an8a2

d1aw7a2

d1bxta2

d1ck1a2

d1d5mc2

d1et6a2

d2fbqa1

d2g7sa1

d2vkea1

d2vkva1

d3bt9a1

d3btia1

d1a6ia1

d1jt6a1

d1qpia1

d1qvta1

d1cbfa_

d2bb3a1

d1s4da_

d1s4db_

d1s4dd_

d1s4de_

d1s4df_

d1s4dg_

d1s4dh_

d1s4di_

d1gv8a_

d1lcfa2

d1a8ea_

d1aiva1

d1b0la1

d1b1xa1

d1b1xa2

d1b3ea_

d1biya1

d1bkaa1

d2dy1a3

d2qale1

d1efga3

d1fnma3

d1i94e1

d1n0ua3

d1pkpa1

d2bv3a3

d2efga3

d2gy9i1

d1geqa_

d1i4na_

d1a53a_

d1beua_

d1dl3a_

d1j5ta_

d1jcmp_

d1k8ya_

d1kfba_

d1kfca_

d2btoa1

d1z2ba1

d1z2bb1

d2vapa1

d1jffa1

d1jffb1

d1ofua1

d1rq2a1

d1tuba1

d1tubb1

d1cnva_

d1edqa2

d1edta_

d1eoka_

d1goia2

d1itxa1

d1jnda1

d1kfwa1

d2ebna_

d2dpea1

d1i7ka_

d1jatb_

d2f4wa1

d2fo3a1

d3h8ka_

d2gmia1

d2nvuc1

d2uyza1

d3e46a2

d1a3sa_

d1a0ca_

d1a0da_

d1a0ea_

d1bxba_

d1bxca_

d1clka_

d1dida_

d1dxia_

d1gw9a_

d1muwa_

d1jbfa_

d1alza_

d1alzb_

d1al4a_

d1al4b_

d1alxa_

d1alxb_

d1tk2b_

d1av2a_

d1av2b_

d1av2c_

d1av2d_

d1bdwa_

d1bdwb_

d1c4da_

d1a5ta2

d1d2na_

d1fnna2

d1g41a_

d1g8pa_

d1in4a2

d1iqpa2

d1jqlb_

d1l8qa2

d1lv7a_

d2a5yb3

d2fnaa2

d2gnoa2

d3cf1a3

d1do0a_

d1byia_

d1cp2a_

d1g3qa_

d1ihua1

d1ihua2

d1ls1a2

d2g0ta1

d3hida_

d2qy9a2

d2vo1a1

d1cg1a_

d1cg3a_

d1cg4a_

d1de0a_

d1dj2a_

d1ez4a2

d1guza2

d1hyha2

d1ldma2

d1llda2

d2cmda2

d1a5za2

d1b8pa2

d1bdma2

d1bmda2

d1civa2

d1d3aa2

d1ez4b2

d1guya2

d1gv0a2

d1aym.1

d1aym1_

d1aym3_

d1b35.1

d1b35a_

d1b35b_

d1bev1_

d2mev.1

d2mev1_

d1al21_

d1ar61_

d1ar71_

d1ar91_

d1bbt1_

d1bev3_

d1b5ea_

d1bkpa_

d4geva_

d2oipa2

d2oipc2

d1aiqa_

d1b02a_

d1b5da_

d1bo7a_

d1bo8a_

d1bp0a_

d1bp6a_

d1bpja_

d1bq1a_

d1dnaa_

d3dy8a_

d1sojl_

d4kp6a_

d3o57a_

d2qyla_

d3hmva_

d3hmvb_

d3w5ea_

d3w5eb_

d3ly2a_

d3ly2b_

d3ly2c_

d3ly2d_

d3ly2e_

d3ly2f_

d1f8fa2

d1gu7a2

d1h2ba2

d1iz0a2

d1jqba2

d1jvba2

d1kola2

d2dm6a2

d2jhfa2

d2nvba2

d1a71a2

d1cdoa2

d1d1ta2

d1e3ia2

d1e3ja2

d1w7ca2

d1w7ca3

d1d6za2

d1d6za3

d1ekma2

d1n9ea2

d1n9ea3

d1w2za2

d1w2za3

d1w6ga2

d1w6ga3

d2oqea2

d2oqea3

d2e2ub2

d2e2ub3

d1a4ia1

d1bgva1

d1c1da1

d1edza1

d1gpja2

d1gtma1

d1leha1

d1luaa1

d1aupa1

d1b0aa1

d1b26a1

d1b3ba1

d1bvua1

d1bw9a1

d1c1db1

d1gq6a_

d2a0ma1

d2aeba_

d1ceva_

d1d3va_

d1hqga_

d1hqxa_

d1p8ma_

d1p8na_

d1p8oa_

d1p8pa_

d1p8qa_

d1p8ra_

d1p8sa_

d1pq3a_

d1lnia_

d2c4ba1

d1a2pa_

d1b20a_

d1b20b_

d1b21a_

d1b21b_

d1b2sa_

d1b2uc_

d1b2xa_

d1b2za_

d1b2zc_

d1b3sa_

d1bana_

d1banc_

d2euta_

d2ghcx_

d3m5qa_

d4jn0a_

d2b11c_

d3r98a_

d2e3ba_

d2e39a_

d2e3aa_

d1ly9a_

d1arya_

d1apxb_

d1apxc_

d1apxd_

d2ggnx_

d1bi5a1

d1bi5a2

d1hnja1

d1hnja2

d2qnza1

d2qo0a1

d2qo0a2

d1bq6a1

d1cgza1

d1chwa1

d1cmla1

d1d6ha2

d1d6ia2

d1ebla2

d1ee0a1

d1a04a2

d1a2oa1

d1dbwa_

d1dz3a_

d1j56a_

d1jbea_

d1k66a_

d1k68a_

d1kgsa2

d1mb3a_

d2ayxa1

d2b4aa1

d2jbaa_

d1ab5a_

d1ab6a_

d3e0ia_

d1d9ea_

d1g7ua_

d1gg1a_

d1gg1b_

d1gg1c_

d1hfbc_

d1kfla_

d1lroa_

d1lrob_

d1lrqb_

d1n8fa_

d1o60a_

d1of6a_

d1of6b_

d1a33a_

d1a58a_

d1c5fg_

d1c5fi_

d1c5fk_

d1clha_

d1cyna_

d1h0pa_

d1lopa_

d1m9db_

d1m9ea_

d1m9eb_

d1qnga_

d1qnha_

d1qnhb_

d1aoea_

d1d1ga_

d1juva_

d1kmva_

d2bl9a_

d3daua_

d3dfra_

d1boza_

d1cz3b_

d1ddsa_

d1df7a_

d1dhfa_

d1dhia_

d1dhja_

d1disa_

d1ih7a1

d1kfsa1

d2f96a1

d2guia_

d2hbja2

d2hhva1

d2igia_

d3hl8a_

d2p5gd1

d2py5a1

d2vwja1

d2vwka1

d3cq8a1

d1d5aa1

d1d8ya1

d2bgra1

d2onca1

d3c45a1

d1j2ea1

d1orva1

d1pfqb1

d1r9ma1

d1xfda1

d1xfdb1

d1xfdc1

d1xfdd1

d2bgrb1

d3c45b1

d2qt9a1

d2qt9b1

d1ec7a2

d1jpdx2

d1jpma2

d1kkoa2

d1muca2

d2akza2

d2gdqa2

d2gl5a2

d2mnra2

d2ptza2

d1bqga2

d1ec7d2

d1ec9d2

d1f9ca2

d1fhua2

d1b5qa1

d1gpea1

d1ju2a1

d1k0ia1

d1kdga1

d2dw4a2

d2f5va1

d2gf3a1

d2gmha1

d2iida1

d2ivda1

d3c96a1

d1ykja1

d2v5za1

d2v5zb1

d1d7ya1

d1ebda2

d1fcda1

d1fcda2

d1feca1

d1feca2

d1fl2a1

d1fl2a2

d1h6va1

d1h6va2

d1lvla1

d1lvla2

d1m6ia1

d1m6ia2

d1mo9a1

d1ftpa_

d1ifca_

d1lfoa_

d1mdca_

d2a0aa1

d2g7ba_

d1a57a_

d1ab0a_

d1adla_

d1b4ma_

d1bwya_

d1crba_

d1dc9a_

d1eala_

d1g5wa_

d1coja2

d1ix9a2

d2qkaa2

d3c3sa2

d3c3ta2

d1b06a2

d1bsma2

d1d5na2

d1dt0a2

d1dt0c2

d1em1a2

d1en4a2

d1en6a2

d1gn2a2

d1gn3a2

d1d3ga_

d1djqa1

d1ep3a_

d1gtea2

d1kbia1

d2e6fa_

d2f6ua1

d2oz0a1

d1bwka_

d1bwla_

d1djna1

d1ea0a2

d1ep1a_

d1f76a_

d1goxa_

d1b9ha_

d1bs0a_

d1fc4a_

d1mdoa_

d2bwna1

d2byla1

d2dkja_

d2fnua_

d1b8ga_

d1b8gb_

d1b9ia_

d1bj4a_

d1bjna_

d1d7sa_

d1dfoa_

d1j8ia_

d1a15a_

d1a15b_

d1b2ta_

d1b3aa_

d1b50a_

d1cm9b_

d1doka_

d1dola_

d1doma_

d1eqta_

d1f2la_

d1f2ld_

d1f9pa_

d1f9qa_

d2gj4a_

d1a8ia_

d1ahpa_

d1axra_

d1b4da_

d1c8ka_

d1fa9a_

d1fc0a_

d1gpab_

d1gpba_

d1gpya_

d1l5qa_

d1l5sa_

d1l5wa_

d1l7xa_

d1cv8a_

d1deua_

d1dkia_

d1k3b.1

d2cb5a_

d2e01a_

d3f5vb_

d3i06a_

d1vsna1

d1a6ra_

d1aeca_

d1aima_

d1by8a_

d1cb5a_

d1cjla_

d1a3ca_

d1bd3a_

d1dqna_

d1ecfa1

d1fsga_

d1g2qa_

d1hgxa_

d1l1qa_

d2aeea1

d2jbha_

d1a4xa_

d1a95a_

d1a95d_

d1a98a_

d1a98b_

d1alla_

d1allb_

d1b8da_

d3l0fa_

d1b33a_

d1b33b_

d1b8db_

d1cpca_

d1cpcb_

d1eyxa_

d1eyxb_

d1f99a_

d1f99b_

d1gh0a_

d1gh0b_

d3dm2b1

d1bqna2

d1bqnb_

d1c0ub_

d1c1ba2

d1c1bb_

d1dloa2

d1dttb_

d1eeta2

d1eetb_

d1ep4b_

d1fkoa2

d1fkob_

d1fkpb_

d1hara_

d1ekea_

d1i39a_

d1jl1a_

d2etja1

d3hyfa_

d1bqna1

d1c1ba1

d1dloa1

d1eeta1

d1f21a_

d1g15a_

d1goaa_

d1goba_

d1goca_

d1hmva1

d2incb_

d3ge3a_

d3mjoa_

d3rmka_

d1t0qa_

d3dhgd_

d3n1ya_

d3q3na_

d3rmkd_

d1t0ra_

d3ri7a_

d1xmfa_

d3rnca_

d1smqd_

d1t0sa_

d2d69a1

d2v63a1

d2v67a1

d2v68a1

d2v69a1

d2v6aa1

d1aa1b1

d1bwva1

d1bxna1

d1ej7l1

d1geha1

d1gk8a1

d1rbaa1

d1rbab1

d1rbla1

d2egdb_

d3d10a_

d4ggfk_

d2egda_

d4etoa_

d4etob_

d4ggfc_

d4ggfl_

d4ggft_

d4ggfv_

d3iqoa_

d3iqob_

d3lk1a_

d3cr5x_

d3llea_

d2ah2a2

d2aepa1

d1e8ua_

d1e8ub_

d1eura_

d1eusa_

d1euta3

d1f8ea_

d1infa_

d1inva_

d1inya_

d1ivga_

d1kita3

d1l7ga_

d1mr5a2

d1gcia_

d2id4a2

d1a2qa_

d1ak9a_

d1aqna_

d1avta_

d1bh6a_

d1c3la_

d1c9ma_

d1c9na_

d1duia_

d1ea7a_

d1gnsa_

d1ht3a_

d1ndua_

d1abaa_

d1egra_

d1fo5a_

d2f51a_

d2fwha_

d2i4aa_

d3m9ja_

d1aaza_

d1aiua_

d1b4qa_

d1cqga_

d1dbya_

d1egoa_

d1ep7a_

d1ep8a_

d1c9sc_

d1gtfa_

d1gtfb_

d1gtfc_

d1gtfj_

d1gtfl_

d1gtfm_

d1qawb_

d1wapa_

d1wapb_

d1gtfd_

d1gtfe_

d1gtff_

d1gtfg_

d1gtfh_

d1jfua_

d1knga_

d1lu4a_

d2a4va1

d2b1la_

d2b5xa1

d2b7ka_

d2bmxa1

d2cvba1

d2cx4a_

d2f8aa1

d2h30a_

d3hvva_

d3mnga_

d1e2ya_

d1e2yb_

d1e2yc_

d1e2yd_

d1e2ye_

d1e2yf_

d1cpta_

d1lfka_

d2ij2a_

d3czha1

d1s1fa_

d2dkka_

d1f4tb_

d1og5b_

d2frza_

d2ij5d_

d3fwfb_

d3p6ma_

d4cp4a_

d2zwta_

d3kx5a_

d3p6na_

d4ek1a_

d1t88b_

d2h7sa_

d2nz5a_

d1c9ka_

d1cr1a_

d1e9ra_

d1g5ta_

d1g6oa_

d2i3ba1

d2jdia3

d2jdid3

d1cr2a_

d1e0ja_

d1e0jb_

d1e79d3

d1e9rb_

d1e9rd_

d1e9re_

d1e9rg_

d1e9sa_

d1e9sb_

d1e9sd_

d1e9se_

d2bxpa1

d2bxpa2

d1o9xa3

d3lu6b3

d4l8ua1

d4l8ua2

d4l8ua3

d4emxa1

d4emxa2

d4emxa3

d4emxb1

d4emxb2

d4emxb3

d3uiva1

d3uiva2

d3uiva3

d3uivh1

d3uivh2

d3uivh3

d2bxpa3

d2c4wa_

d1d0ia_

d1d0id_

d1d0ie_

d1d0ih_

d1gqoa_

d1gqob_

d1gtza_

d1gu1a_

d1h05a_

d1h0sa_

d1uqra_

d1uqrc_

d1uqrd_

d2dhqa_

d1uqrb_

d1uqre_

d1uqrf_

d1uqrg_

d1uqrh_

d4kf3a_

d4kf3b_

d4hg9a_

d4hg9d_

d3ux7a_

d3ux7b_

d3ux7d_

d3ux7e_

d3ux7f_

d3ux7g_

d3ux7h_

d4h0sa_

d4h0sb_

d4h0sc_

d4h0qb_

d2baxa_

d2bcha_

d1unea_

d4bp2a_

d1bp2a_

d1ajsa_

d1bw0a_

d1fg7a_

d1gdea_

d1lc5a_

d1m6sa_

d2f8ja_

d2gb3a1

d2hoxa_

d3fvsa_

d1aama_

d1aata_

d1ahxa_

d1aiaa_

d1akaa_

d1arha_

d1aria_

d1asga_

d1b5oa_

d1b5pa_

d1dx4a_

d1ea5a_

d2bcea_

d2h7ca_

d1akna_

d1amna_

d1aqla_

d1b41a_

d1c2oa_

d1c7ia_

d1c7ja_

d1e3qa_

d1evea_

d1f6wa_

d1f8ua_

d1h22a_

d1jmya_

d1k4ya_

d1ku6a_

d1maaa_

d1bupa1

d1bupa2

d1e4ft1

d1e4ft2

d1jcfa1

d1jcfa2

d1k8ka2

d2fxua1

d2fxua2

d2e8aa1

d2e8aa2

d2oana2

d2qw9a1

d2qwla2

d2qwna1

d2qwoa1

d2zgya1

d2zgya2

d3d2fb1

d1atna1

d1gvea_

d1lqaa_

d3eaua_

d1a80a_

d1abna_

d1afsa_

d1ah4a_

d1az1a_

d1cwna_

d1dlaa_

d1el3a_

d1gveb_

d1hqta_

d1hw6a_

d1ihia_

d1j96a_

d1jezb_

d1lwia_

d1m9ha_

d1mi3a_

d1bf2a2

d1e43a1

d1hx0a1

d1j0ha2

d1ji1a2

d1ktba1

d1m7xa2

d2fhfa4

d2guya1

d3bmva3

d2qpsa1

d2qpua1

d3baxa1

d3bsga1

d3dhpa1

d1avaa1

d1baga1

d1blia1

d1cgta3

d1cxla3

d1bf2a3

d1e43a2

d1g5aa2

d1gcya2

d1gjwa2

d1h3ga3

d1ht6a2

d1hx0a2

d1iv8a2

d1ji1a3

d1kwga2

d1lwha2

d1m7xa3

d2bhua3

d2fhfa5

d2guya2

d3bmva4

d3baxa2

d3dhpa2

d1avaa2

d1dmha_

d1ykkb1

d1ykob1

d2buxa1

d1eo9a_

d3pcca_

d3pccm_

d3pcdm_

d3mi1n_

d3t67n_

d1ykoh_

d3lktq_

d3t63o_

d1ykld_

d1ykll_

d1ykmj_

d1yknd_

d1yknf_

d1ykph_

d3lxvn_

d2f01a_

d2qcba1

d1avda_

d1avdb_

d1df8a_

d1ij8a_

d1ij8b_

d1kffa_

d1kl3a_

d1kl3b_

d1kl3d_

d1kl4a_

d1kl4b_

d1kl4c_

d1kl5a_

d1lcva_

d1ldqa_

d1luqa_

d1luqb_

d1mepa_

d1bhga1

d1jz8a1

d1jz8a2

d2je8a1

d2je8a2

d2je8a3

d1yq2a1

d1yq2a2

d2vzsa1

d2vzsa2

d2vzsa3

d3hn3a2

d3hn3b2

d3hn3d2

d3hn3e2

d2je8b2

d2vqub3

d2x05a2

d2x05a4

d2x05a5

d1ci9a_

d1djaa_

d1e25a_

d1es5a_

d1k38a_

d1k55a_

d1m40a_

d2bg1a1

d2dcfa1

d2drwa1

d3beca2

d2b5ra1

d2g2wa1

d2zc3b1

d1alqa_

d1blpa_

d1bsga_

d1buea_

d1bzaa_

d1ck3a_

d1jbaa_

d3fwba_

d3jtdc_

d1cm4a_

d4anjb_

d3ucya_

d3suia_

d2i08a_

d4dckb_

d1yrtb_

d1yrub_

d2pq3a_

d4g27r_

d3b32a_

d4g28r_

d4j9zr_

d3sjqa_

d3sjqb_

d3sd6a_

d4gjea_

d2f6ia1

d1tg6a1

d1y7oa1

d2cbya1

d3tt7a_

d3tt7b_

d3tt7c_

d3tt7d_

d3tt7e_

d3tt7f_

d3tt7g_

d3tt6a_

d3tt6b_

d3tt6c_

d3tt6d_

d3tt6e_

d3tt6f_

d3tt6g_

d1tyfa_

d1tyfb_

d1ej8a_

d2aqma_

d1azva_

d1b4ta_

d1bzoa_

d1cb4a_

d1do5a_

d1do5b_

d1do5d_

d1dswa_

d1e9pa_

d1e9pb_

d1e9qa_

d1e9qb_

d1eqwa_

d1eqwb_

d1eqwc_

d1esoa_

d1f18a_

d1f1aa_

d1kxua1

d2f2ca1

d3bhtb1

d3bhtd1

d3my5b1

d3my5b2

d3my5d1

d3my5d2

d3bhvb1

d3bhvd1

d3bhub1

d3bhud1

d4eojb1

d4eojb2

d4eojd1

d4eojd2

d4eopb1

d4eopb2

d4eopd1

d4eopd2

d2czda_

d3gdla_

d1dbta_

d1dbtb_

d1dqwa_

d1dv7a_

d1dvja_

d1dvjb_

d1dvjc_

d1dvjd_

d1eixa_

d1eixc_

d1klya_

d1klza_

d1km0a_

d1km0b_

d1km0c_

d1km0d_

d1km1a_

d1km1b_

d1axib1

d1axib2

d1bpva_

d1bqua1

d1bqua2

d1cd9b1

d1cd9b2

d1cfba1

d1cfba2

d1eerb1

d1eerb2

d1f6fb1

d1f6fb2

d1fnaa_

d1fnfa1

d1fnfa2

d1fnfa3

d1fnha1

d1fnha2

d1fyhb1

d2rh7a1

d1b9ca_

d1bfpa_

d1c4fa_

d1cv7a_

d1emaa_

d1emca_

d1emcc_

d1emea_

d1emfa_

d1emka_

d1emla_

d1emma_

d1f09a_

d1f0ba_

d1g7ka_

d1gfla_

d1ggxa_

d1h6ra_

d1hcja_

d1biha1

d1biha2

d1biha3

d1biha4

d1cs6a1

d1cs6a2

d1cs6a3

d1cs6a4

d1epfa1

d1epfa2

d1ev2e2

d1f2qa2

d1f97a2

d1fhga_

d1fltx_

d1g1ca_

d1gl4b_

d1gsma1

d1gsma2

d1gxea_

d1hyea1

d1hyha1

d1i0za1

d1ldna1

d1llda1

d2cmda1

d1a5za1

d1b8pa1

d1bdma1

d1ceqa1

d1civa1

d1ez4a1

d1guya1

d1guza1

d1gv0a1

d1gv1a1

d1hlpa1

d1i10a1

d1ib6a1

d1ldga1

d1c52a_

d1cc5a_

d1ctja_

d1dw0a_

d1e29a_

d1gu2a_

d1h32b_

d1i8oa_

d2bh4x_

d451ca_

d2b0zb1

d2b10b1

d2b11b1

d2b12b1

d155ca_

d1a56a_

d1c2ra_

d1c53a_

d1c6oa_

d1c6ra_

d2huec_

d3mvdc_

d3mvdg_

d3azla_

d3azia_

d3azie_

d3azje_

d3azme_

d2hueb_

d4eo5b_

d4eo5c_

d2nqbc_

d2nqbg_

d2pyoc_

d2pyog_

d4h9na_

d3nqja_

d4h9sb_

d3azge_

d3azlb_

d1ckea_

d1deka_

d1e2ka_

d1gkya_

d1gsia_

d1kgda_

d1khta_

d1kjwa2

d1lw7a2

d1ly1a_

d2ak3a1

d2c95a_

d2grja_

d2h92a_

d3akya1

d3iija_

d3kb2a_

d1ak2a1

d1akea1

d1akya1

d1f56a_

d2bz7a_

d2cala_

d3cvba_

d3fsaa_

d2jxma1

d1a3za_

d1a4aa_

d1a8za_

d1adwa_

d1ag0a_

d1ag6a_

d1azca_

d1azna_

d1azra_

d1azua_

d1bqka_

d1bxua_

d1byoa_

d1bypa_

d1bj7a_

d1dzka_

d1epba_

d1kt7a_

d1kxoa_

d1lf7a_

d2joza1

d3kffa_

d1aqba_

d1bbpa_

d1bsoa_

d1bsqa_

d1df3a_

d1dfva_

d1dfvb_

d1dv9a_

d1dzpa_

d1dzpb_

d1epbb_

d1erba_

d1dy5a_

d1gqva_

d2bwka_

d1a5pa_

d1a5qa_

d1agia_

d1b1ea_

d1b1ja_

d1c8wa_

d1c9va_

d1c9xa_

d1dyta_

d1dzaa_

d1dzab_

d1e21a_

d1eica_

d1eida_

d1eiea_

d1gv7a_

d1h0dc_

d1afwa1

d1m3ka1

d1m3ka2

d2vbaa1

d2vbaa2

d1afwa2

d1e5ma1

d1e5ma2

d1ek4a1

d1ek4a2

d1h4fa2

d1j3na1

d1j3na2

d1m1ta1

d1m3za1

d1m4sa1

d1m4ta1

d1ox0a1

d1ox0a2

d1oxha1

d4ky2a_

d4ky2b_

d4hisa_

d4hisb_

d4fi8a_

d4fi8b_

d3p3ra_

d3p3rb_

d3tcta_

d3tctb_

d4fi7a_

d4fi6a_

d4fi6b_

d3p3ta_

d3p3tb_

d3p3ua_

d3p3ub_

d3p3sa_

d3p3sb_

d4i87b_

d1amka_

d1aw1a_

d1aw1b_

d1b9ba_

d1b9bb_

d1btma_

d1dkwa_

d1hg3a_

d1kv5a_

d1m6ja_

d1ml1a_

d1mo0a_

d1mo0b_

d1n55a_

d1neya_

d1o5xa_

d1qdsa_

d1r2ra_

d1r2rb_

d1spqa_

d1lm8b_

d2o6vd1

d1a5ra_

d1c3ta_

d1cmxd_

d1euvb_

d1gjza_

d1iyfa_

d1l2na_

d1lqba_

d1ndda_

d1nddb_

d1nddc_

d1ogwa_

d1oqya4

d1p0ra_

d1p1aa_

d1p98a_

d1sifa_

d1t0ya_

d1g3ka_

d2h6jh1

d1e94a_

d1g0u2_

d1g0ua_

d1g0ub_

d1g0uc_

d1g0ud_

d1g0ue_

d1g0uf_

d1g0ug_

d1g4aa_

d1g65k_

d1iru1_

d1iru2_

d1irua_

d1irub_

d1iruc_

d1irud_

d1irue_

d1iruf_

d1irug_

d1iruh_

d1irui_

d1iruj_

d1bhga3

d1bqca_

d1ceoa_

d1ecea_

d1edga_

d1h1na_

d1hjua_

d1i1wa_

d1jz8a5

d2c0ha1

d2cyga1

d2je8a5

d3emca_

d7a3ha_

d1aq0a_

d1bg4a_

d1ceca_

d1clxa_

d1e0xa_

d1e5na_

d1egza_

d1eqpa_

d1fh9a_

d1foba_

d1g01a_

d1jd0a_

d1kopa_

d3fw3a_

d3k34a_

d12caa_

d1bica_

d1bzma_

d1caia_

d1caja_

d1cama_

d1caza_

d1ccsa_

d1ccta_

d1ccua_

d1cnca_

d1cnga_

d1cnha_

d1cnia_

d1cnja_

d1cnka_

d1crma_

d1cvaa_

d1cvca_

d1cvda_

d1cvea_

d1f74a_

d1l6wa_

d2a4aa1

d3a5fa_

d3l21a_

d2qapa1

d1a5ca_

d1adoa_

d1euaa_

d1ewda_

d1ewea_

d1ex5a_

d1f05a_

d1f2ja_

d1fbaa_

d1fdja_

d1fdya_

d1fdyb_

d1fwra_

d1gqna_

d1hl2a_

d1hl2b_

d1i2na_

d1i2oa_

d1i2pa_

d1eema1

d1g7oa1

d2fnoa1

d2ljra1

d2a2ra1

d2c80a1

d2c80b1

d2ca8a1

d3ktla2

d3ktlb2

d3q74a2

d3q74b2

d3ik7a2

d3ik7b2

d3ik7c2

d3ik7d2

d3l0ha2

d3l0hb2

d4hj2a2

d4hj2b2

d3ik9a2

d3ik9b2

d3ik9c2

d3ik9d2

d3ik9e2

d1eema2

d1gnwa2

d1jlva2

d1k0da2

d1k0ma2

d2c4ja2

d2fnoa2

d1u87a2

d1y6ea2

d2r3xa2

d2r3xb2

d2r6ka2

d2r6kb2

d13gsb2

d17gsa2

d17gsb2

d1aw9a2

d1axda2

d1b48a2

d1bx9a2

d1duga2

d1e6ba2

d1ev4a2

d1f2ea2

d1f3ba2

d1cf2o1

d1drua1

d1e5qa1

d1ebfa1

d1f06a1

d1gr0a1

d1h6da1

d1h9aa1

d1j5pa4

d1lc0a1

d2g17a1

d2g82a1

d2gz1a1

d2hjsa1

d3cina1

d2czca2

d2czcb1

d2czcd1

d2pkqo1

d3cmco1

d3e1ka1

d1arzb1

d1arzc1

d1b7go1

d1brma1

d1de4a2

d1hdma2

d1hdmb2

d1hyrc2

d1jfma_

d1k5na2

d1kcgc_

d1lqva_

d2akra2

d2h26a2

d3frua2

d1u58a2

d1vgka2

d1ydpa2

d1zs8a2

d1zt1a2

d1zvsa2

d2d31a2

d2dypa2

d2jcca2

d2nnab2

d2p24a2

d2po6a2

d2pxyd2

d2q7ya2

d1aoza1

d1aoza2

d1aoza3

d1gska1

d1gska2

d1gska3

d1hfua1

d1hfua2

d1hfua3

d1kbva1

d1kbva2

d1kv7a1

d1kv7a2

d1kv7a3

d2bw4a1

d2bw4a2

d2j5wa1

d2j5wa2

d2j5wa3

d2j5wa4

d2e86a1

d2e86a2

d2ppea1

d2ppfa1

d2q9oa1

d1k28a3

d102la_

d103la_

d104la_

d107la_

d108la_

d109la_

d110la_

d111la_

d112la_

d113la_

d114la_

d115la_

d118la_

d119la_

d120la_

d122la_

d123la_

d125la_

d126la_

d127la_

d128la_

d129la_

d130la_

d131la_

d1a99a_

d1al3a_

d1atga_

d1elja_

d1eu8a_

d1h3da1

d1i6aa_

d1ii5a_

d1ixca2

d1ixha_

d1jeta_

d1lsta_

d2a5sa1

d2czla1

d2esna2

d2fyia1

d2i6ea1

d3e13x_

d3fasa_

d3kjta_

d4f1va_

d2onkj1

d3csba2

d1a40a_

d1a54a_

d1g2oa_

d1je0a_

d2a8ya_

d3ddoa_

d3emva_

d3fuca_

d1a9oa_

d1a9ra_

d1b8na_

d1b8oa_

d1cb0a_

d1fxua_

d1i80a_

d1i80b_

d1je1b_

d1jysa_

d1k3fa_

d1k9sa_

d1lv8a_

d1lvua_

d1lvuc_

d1lx7b_

d1m73e_

d1nc1a_

d1nc3a_

d2e2ra_

d3a40x_

d3ilza_

d1erra_

d2q6ra_

d2r40d_

d2p7ga_

d2p7aa_

d2p7za_

d3v3eb_

d3v3qa1

d3v3qb1

d2qw4b_

d2qw4c_

d2qw4d_

d3falb_

d3fald_

d3et1a_

d2xkwa_

d3b0qa_

d4em9b_

d3b0ra_

d3an4a_

d3bc5a_

d3an3a_

d4emaa_

d3sp9a_

d3r8da_

d2qnva_

d2pina_

d1csna_

d1gz8a_

d1j1ba_

d1koba_

d1mp8a_

d3fpqa_

d3gu4a_

d2h8ha3

d2iw8a1

d2psqa1

d2z8ca1

d3d7ua1

d3dcva1

d1a9ua_

d1ad5a3

d1apme_

d1b6cb_

d1bi7a_

d1bi8a_

d1blxa_

d1buha_

d1byga_

d1cdka_

d1ckia_

d1ckib_

d1ckja_

d1cmke_

d1ctpe_

d1ds5a_

d1erka_

d1b9oa_

d133la_

d134la_

d1a2yc_

d1alca_

d1b5ua_

d1b5va_

d1b5wa_

d1b5xa_

d1b5ya_

d1b5za_

d1b7la_

d1b7ma_

d1b7na_

d1b7oa_

d1b7pa_

d1b7qa_

d1b7ra_

d1b7sa_

d1bb3a_

d1bb4a_

d1c43a_

d1c45a_

d1c46a_

d1c7pa_

d1cj6a_

d1cj7a_

d1cj8a_

d1cj9a_

d1ckca_

d2cf7a_

d2fzfa1

d3ak8i_

d3bvfa_

d3e6sa_

d3rgdf_

d3rgdg_

d3rgdh_

d3rgdi_

d3rgdn_

d3rgdr_

d3rgds_

d3rgdt_

d3rgdx_

d4de6a_

d3kxua_

d3hx7a_

d3hx7b_

d3hx7c_

d3hx7d_

d3hx7e_

d3hx7f_

d3hx7g_

d3hx7h_

d3hx7i_

d3hx7j_

d3hx7k_

d3hx7l_

d3hx7m_

d3hx7n_

d1f5na2

d1f60a3

d1f6ba_

d1g7sa4

d1h65a_

d1jala1

d1kk1a3

d1mh1a_

d1mkya1

d1mkya2

d2akab1

d2bv3a2

d2c78a3

d2ce2x_

d2clsa_

d2cxxa1

d2dy1a2

d2erxa1

d2fh5b1

d2gf9a_

d2gj8a_

d2iwra_

d3gj0a_

d4ac9a4

d2g77b1

d2om2a2

d2om2c2

d2ot3b1

d2qn6a3

d2rgnc1

d1cyda_

d1d7oa_

d1e6ua_

d1ek6a_

d1eq2a_

d1fjha_

d1fmca_

d1geea_

d1gega_

d1gy8a_

d1gz6a_

d1h5qa_

d1hdca_

d1hdoa_

d1hxha_

d1i24a_

d1iy8a_

d1ja9a_

d1jtva_

d1k2wa_

d2a35a1

d2ae2a_

d2ag5a1

d2b69a1

d2bd0a1

d2bgka1

d2bkaa1

d2blla1

d2c07a1

d2c5aa1

d1asha_

d1b0ba_

d1cg5a_

d1cg5b_

d1cqxa1

d1ecaa_

d1h97a_

d1hlba_

d1it2a_

d1itha_

d1jl7a_

d1mbaa_

d2gdma_

d3boma_

d3g46a_

d3lb2a_

d3mkbb_

d1s0ha1

d1yihb1

d2aa1b1

d2b7hb1

d3ciua1

d102ma_

d1a00b_

d1a01b_

d1a3oa_

d1a4fa_

d1a4fb_

d1a6ga_

d1a6ma_

d1a9we_

d1aj9a_

d1baba_

d1bjea_

d1buwb_

d1bz1a_

d1bzza_

d1c40a_

d1c7ca1

d1c7cb_

d1c7da2

d1ch1a_

d1ch2a_

d1ch3a_

d1ch5a_

d1ch7a_

d1ch9a_

d1cika_

d1cioa_

d1cmyb_

d2qupa_

d3et6a_

d3et6b_

d2wz1a_

d2wz1b_

d3uvjb_

d3uvjd_

d1yk9a_

d3mr7a_

d3mr7b_

d2w01a_

d2w01b_

d2w01c_

d2w01d_

d2w01e_

d3r5ga_

d3iqca_

d3iqcb_

d3k1ia_

d3k1ib_

d3ldza_

d3zyqa1

d4avxa1

d3zyla1

d3zylb1

d3zyka1

d3zykb1

d3ldzb_

d3ldzc_

d3ldzd_

d2j9wa_

d2j9wb_

d4f53a_

d4lera_

d4gcoa_

d2if4a2

d2f42a1

d3gzsa_

d3gzsb_

d4f53b_

d3sgha_

d3sghb_

d2y3ya_

d2y3yb_

d2y3yc_

d2y3yd_

d1wola_

d3s7xa_

d3s7xc_

d4jcea_

d4jceb_

d4jcec_

d4jced_

d4jcee_

d4jcda_

d4jcdb_

d4jcdc_

d4jcdd_

d4jcde_

d3nxga_

d3nxgb_

d3nxgc_

d3nxgd_

d3nxge_

d3nxda_

d3nxdb_

d3nxdc_

d3nxdd_

d3nxde_

d4jcfa_

d4jcfb_

d4jcfc_

d4jcfd_

d4jcfe_

d3s7va_

d3s7vb_

d3s7vc_

d3s7vd_

d3s7ve_

d3s7vf1

d3s7vg_

d3s7vh_

d3s7vi_

d3s7vj_

d3bwra_

d3bwrc_

d3bwrd_

d3bwre_

d3bwqa_

d3bwqc_

d3bwqd_

d3bwqe_

d3s7xb_

d3s7xd_

d3s7xe_

d3d4ja2

d3d4jb2

d3f0na2

d3f0nb2

d2deja2

d1ccza1

d1cida1

d1dr9a1

d1eaja_

d1f97a1

d1fo0b_

d1hkfa_

d1hnfa1

d1hxma1

d1hxmb1

d1jmaa_

d1kgcd1

d1l6za1

d1lp9e1

d1mjuh1

d1mjul1

d2ak4d1

d2atpb1

d2bnqd1

d2crya1

d2g5ra_

d2giya1

d2gj6d1

d2hp4a_

d2ij0c1

d1ri8a1

d1rjca1

d1u3ha1

d1u3hb1

d1ymmd1

d1ypze1

d1ypzf1

d1z3gl1

d1zmya1

d1zv5a1

d1zvha1

d1zvya1

d2aabl1

d2agjh1

d2ak4e1

d2aq2a1

d2aq3a1

d2arja1

d2arjb1

d2bdnh1

d2bmkb1

d2bnub1

d2cdea1

d2dd8h1

d2dd8l1

d2dqch1

d2dqdh1

d2dqeh1

d2dqfb1

d2dqgh1

d2dqhh1

d2dqil1

d2dtgb1

d2dtgc1

d2dtgd1

d2esvd1

d2esve1

d2f53d1

d2f53e1

d2f54d1

d2fath1

d2fatl1

d2ghwb1

d2ghwb2

d2gsib1

d2hwzl1

d2icwj1

d2j4wh1

d2j6eh1

d2j6el1

d2jb5h1

d2jixd1

d2ntsp1

d2ok0l1

d2q86b1

d2qqnh1

d2qr0a1

d2qr0b1

d2qscl1

d2r0kh1

d2r0lh1

d2r69l1

d2rcja1

d2uudh1

d2v7na1

d2vlre1

d2z92b1

d3b2uc1

d3bkyh1

d3bn9d1

d3c08h1

d3c09c1

d3c2ah1

d3c6la1

d3cmol1

d1u3hd1

d2iwga2

d2bmkb2

d2nxyc2

d2ok0l2

d2ck0l2

d7fabl2

d1cicb2

d1a0qh2

d2cgrh2

d1mrdh2

d1qblh2

d1emth2

d1kcuh2

d1c1eh2

d1dqdh2

d2ck0h2

d1ay1h2

d1mlbb2

d1mh5h2

d1t66d2

d1ikfh2

d1indh2

d1ngph2

d1cf8h2

d1a6tb2

d1eapb2

d1ub6a2

d2pcpb2

d2j4wh2

d1fdlh2

d1iqwh2

d2q8ah1

d1qbmh2

d1gigh2

d1mh5b2

d1ub5a2

d1nc2d2

d12e8h2

d1hilb2

d1qkzh2

d1p2ce2

d1p2cb2

d1mfbh2

d1uywh1

d2qhrh1

d1sbsh2

d1mjjh2

d1mjjb2

d1f58h2

d2q76b1

d1f8th2

d1osph2

d1nc2b2

d1e4xi2

d1e4xh2

d1fe8h2

d1teth2

d1h0db2

d2vdrh1

d6fabh2

d1lo0h2

d35c8h2

d1sm3h2

d2g60h1

d1jfqh2

d1ejoh2

d1m7db2

d1ae6h2

d1jrhh2

d1fbih2

d1egjh2

d1nsnh2

d1kfai2

d1kfah2

d1a5fh2

d1otsc2

d2z93a1

d2gsib2

d1dbjh2

d1iaih2

d1iaii2

d1hi6b2

d1pz5b2

d2gfbb2

d1t4kb2

d1rjlb2

d2dtgc2

d1afvh2

d2hmid2

d1orqb2

d1fskc2

d32c2b2

d1yuhb2

d1r0ah2

d1ghfh2

d1jnhb2

d1h3ph2

d1ztxh1

d1c12b2

d2bdnh2

d1nj9b2

d1fgnh2

d1etzb2

d1forh2

d1mpah2

d1pg7x2

d1plgh2

d1k6qh2

d1mnuh2

d2j88h1

d1igch2

d1rmfh2

d1kcrh2

d1nldh2

d1igtb2

d1hq4b2

d1lo4h2

d1fh5h2

d1fj1b2

d1pskh2

d2f19h2

d1ibgh2

d1ggbh2

d2a6ib1

d2jelh2

d2f5ah2

d1nfdf2

d1f6ab2

d1o0vb3

d1o0va3

d1fp5a2

d1f6ad1

d1f6ab1

d1o0va2

d1fp5a1

d1g84a_

d1o0va1

d1mcph2

d1c5ch2

d1um5h2

d1rzfh2

d1u8ib2

d2f5bh2

d1ce1h2

d1iqdb2

d1tjgh2

d1aqkh2

d7fabh2

d2fb4h2

d1op3h2

d1t3fb2

d8fabb2

d2fx7h2

d2fbjh2

d3frua1

d1exua1

d1zglb1

d2fsed1

d2fseb1

d1sebb1

d1a6ab1

d1bx2e1

d1bx2b1

d1aqdk1

d2nnab1

d1d5mb1

d1d5zb1

d1fv1b1

d1ymmb1

d1k8ib1

d1iakb1

d1i3rb1

d1ktdb1

d1fngb1

d1d9kd1

d1f3jb1

d1iaob1

d2iadb1

d3c5zd1

d1es0b1

d1k2db1

d2pxyd1

d1mujb1

d1klub1

d1aj7h2

d1q0yh2

d1p7kh2

d1p7kb2

d1qygh2

d1yqvh1

d1q0xh2

d1mj8h2

d3bkjh1

d1rurh2

d1ncwh2

d1mexh2

d1mjuh2

d2hh0h2

d1q72h2

d1kcvh2

d1jguh2

d1a3rh2

d1cr9h2

d1kelh2

d2fath2

d2brrh1

d2a77h1

d1r3jb2

d1ndgb2

d1fnsh2

d1wejh2

d1jgvh2

d1nbyb2

d1uj3b2

d1s3kh2

d1ngzb2

d1bz7b2

d1a4jb2

d2p8lb2

d1ad0b2

d3fctb2

d1u92b2

d2ny7h2

d1om3k2

d2dd8h2

d3c2ah2

d1u8kb2

d1nl0h2

d2g75a2

d1cz8h2

d1ucbh2

d2rcja2

d1mcoh2

d3c09c2

d2ig2h2

d1bbjb2

d1uweh2

d1hzhh2

d1mimh2

d1g9mh2

d1a4kh2

d1a4kb2

d1pg7h2

d1tzgh2

d1mcol2

d1aqkl2

d2fb4l2

d8faba2

d1rzfl2

d1q0xl2

d1nfde2

d2v7nc2

d1fn4a2

d1c5da2

d1bfoa2

d1lk3l2

d2gfba2

d1lgva2

d1jvka2

d1jvkb2

d1mcww2

d2j6el2

d1adql2

d1lila2

d1q1jl2

d1nj9a2

d1pg7w2

d2g75b2

d2dd8l2

d1nl0l2

d1nc2c2

d1nc2a2

d1t4ka2

d2r69l2

d2dtgb2

d1v7ml2

d1blna2

d1eo8l2

d1ifhl2

d1igcl2

d2aabl2

d2j88l2

d1fl3b2

d1c12a2

d2gcya2

d2jell2

d1ejol2

d1kcrl2

d1h3pl2

d1ghfl2

d1z3gl2

d1hysc2

d1r24a2

d2hmic2

d1fptl2

d1hi6a2

d1boga2

d1jrhl2

d1egjl2

d1kfam2

d1kfal2

d2z93d2

d1cf8l2

d1mfbl2

d1zagd1

d1hxma2

d1ypze2

d1hxmb2

d1ktkf2

d1ktke2

d1ymme2

d1zglr2

d1zglp2

d1qsfe2

d1qsee2

d1qrne2

d1bd2e2

d1ypzf2

d1lp9e2

d1xe0g_

d1xe0h_

d2vtxa_

d4fwpa1

d4fwqa2

d4fwqa1

d3mvta_

d3mvtc_

d1qxla_

d1xvfe_

d1xvff_

d1fz7e_

d1lqjd_

d1lqme_

d5euga_

d3i3tc_

d2wdta_

d3i3te_

d2y5ba_

d3mtna_

d3mtnc_

d3i3ta_

d2etlb_

d4i6la_

d3tb3b_

d3tb3a_

d3riib_

d3riia_

d3a7sa_

d3risb_

d3risc_

d3risd_

d3s3rc_

d3qt4a_

d3qj3b_

d3morb_

d3mora_

d3qj3a_

d3hhib_

d3hhia_

d3f75a_

d2we6b_

d2we6a_

d2wdtc_

d2zfya_

d3u8ea_

d2o6xa_

d3hbha_

d3hbex_

d3cb7a_

d2goic_

d2goia_

d3cb7b_

d2h5zb_

d2h5za_

d2fbdb_

d2fbda_

d2goib_

d3hbda_

d2cjlb_

d2cjla_

d3hzsa_

d3rgha_

d4alsa_

d4a02a_

d3uypb_

d2oxhe_

d2yowa_

d2yoya_

d2bcma_

d3rghb_

d2okma_

d2oxhz_

d3e9tc_

d2bcmb_

d2bcmc_

d4alqa_

d4alea_

d4alta_

d2bp3b_

d2oxga_

d2oxgc_

d2oxge_

d2oxgz_

d2ox5a_

d2ox5c_

d2ox5e_

d2brqb_

d2ox5z_

d2oxha_

d2oxhc_

d4alra_

d4alca_

d3umna_

d3umnc_

d3sz6a_

d3sz6b_

d4h8pa_

d3sika_

d3sikb_

d3ul4a_

d2ylkc_

d2ylkb_

d2wobe_

d2wobc_

d2woba_

d2wo4a_

d2wnxa_

d3zqxa_

d2xbta_

d3zu8a_

d3zqwa_

d4h8qa_

d3h6ab_

d3h6aa_

d3umnb_

d3vtta_

d2ciob_

d3e9ta_

d3e9tb_

d3e9td_

d3eada_

d2amua_

d3qzba_

d3eadb_

d3eadc_

d3eadd_

d3e9ua_

d3fsoa_

d3fsob_

d3fq4a_

d3fq4b_

d3zuca_

d1zvna_

d1zvnb_

d2e46a_

d2a4ca_

d2a4cb_

d3b83h_

d3b83g_

d2e47b_

d2e47a_

d1zxkb_

d3b83f_

d3b83e_

d3b83d_

d3b83c_

d3b83b_

d3b83a_

d2g2nd_

d2g2pa_

d2g2pb_

d2g2pc_

d2g2pd_

d3qvad_

d2g2nc_

d2g2nb_

d2g2na_

d2igld_

d2iglc_

d2iglb_

d2igla_

d2h0fb_

d2h6uf_

d2gpza_

d2gpzc_

d3q1ec_

d3q1eb_

d3q1ea_

d2h1xd_

d2h1xc_

d2h1xb_

d2h1xa_

d2h6uh_

d2h6ug_

d2h6ue_

d2h6ud_

d2h6uc_

d2h6ub_

d2h6ua_

d3q1ed_

d4en2m_

d3p73b_

d1u2ha_

d3gbla_

d2yf6b_

d2yf5b_

d3bewe_

d3bewb_

d3jvgd_

d3jvgc_

d3bevb_

d3p77b_

d2yxma_

d3bw9a1

d2jjwa_

d1z5lc1

d1z5la1

d1zoxa_

d4jvua_

d3k3qa_

d3kvqa_

d2nmsa_

d3bj91_

d3q5ob_

d2y9rt_

d2q87b_

d3hzba_

d3hzbb_

d3iaja_

d3i9hh_

d3hzbc_

d3so1b_

d3so1c_

d3so1d_

d3so1e_

d3hzbh_

d3hzbg_

d3hzbf_

d3hzbe_

d3hzbd_

d3i9hg_

d3i9hf_

d3so1h_

d3so0a_

d3so0b_

d3so1f_

d3so0d_

d3so0e_

d3so0f_

d3so0g_

d3so0h_

d3snza_

d3i9ha_

d3i9hb_

d3i9hc_

d3i9hd_

d3i9he_

d3so1g_

d3so1a_

d3snya_

d3ef4c_

d3ft1d_

d3fbka_

d3so0c_

d3fbkb_

d2b3ra_

d2b3rb_

d3kwua1

d3kwta_

d3ft1a_

d3ft1b_

d3ft1c_

d3ef4b_

d3ef4a_

d3heip_

d1x9ua_

d1x9ub_

d1x9ra_

d1x9rb_

d3heib_

d3heid_

d3heif_

d3heih_

d3heij_

d3heil_

d3hein_

d3ft9a_

d4b30b_

d3gl4b_

d2c9ja_

d2z1oa_

d2otbb_

d2otba_

d2c9jb_

d2c9jg_

d2oteb_

d2c9jc_

d2otea_

d2hqka_

d2c9jh_

d2c9jd_

d2c9je_

d2c9jf_

d1xssb_

d2a47a_

d2a46a_

d3rmuc_

d3rmud_

d2c21b_

d2c21c_

d2c21d_

d2c21e_

d2c21f_

d3fcda_

d3fcdb_

d2rk9b_

d4jeta_

d4jetc_

d4jetd_

d4jete_

d3jv1a_

d2zmua_

d2zo7a_

d3rmub_

d2z1ob_

d3zuff_

d3zuja_

d3zujb_

d3zujc_

d3zulb_

d3zujd_

d3zuje_

d3zujf_

d3zula_

d3zulc_

d3zuld_

d3zule_

d3zulf_

d2ddda_

d2dddb_

d2ddca_

d2ddcb_

d3zufe_

d3zufd_

d2z1oc_

d2z1od_

d2ie2a_

d2ie2b_

d2ie2c_

d2ie2d_

d2ie2e_

d2ie2f_

d2gx2l_

d2gx0d_

d2z6ya_

d2z6yb_

d2z6yc_

d2z6yd_

d3zufa_

d3zufb_

d3zufc_

d1xssa_

d3oe2a_

d3mgfd_

d2gw3b_

d2gw3a_

d3tmtd_

d3tmtc_

d3tmtb_

d3tmta_

d3p8ud_

d3p8uc_

d3p8ub_

d3p8ua_

d3s05d_

d3s05c_

d3s05b_

d3s05a_

d2vvic_

d2vvib_

d2icrd_

d2ojkb_

d3ihzb_

d3ihza_

d4itzb_

d3ni6b_

d3ni6a_

d2vn1b_

d3prab_

d3praa_

d3pr9a_

d4dz2b_

d4dz3b_

d1zuxd_

d2vvid_

d1xaeb_

d1xa9a_

d2fl1d_

d2pxwb_

d2vvia_

d2vvjd_

d2vvjc_

d2vvha_

d3rmua_

d2a48a_

d2vvjb_

d2vvhb_

d3omsa_

d3hdpa_

d2qh0a_

d2vvhc_

d2vvhd_

d3tmra_

d3tmrb_

d2vvja_

d1zuxc_

d1zuxb_

d3tmrd_

d1zuxa_

d3tmrc_

d3q90b_

d4fcma_

d3imab_

d2ch9a_

d3q90a_

d3grda_

d3grdb_

d3fgya_

d3fgyb_

d1zo2b_

d3ujmb_

d3duka_

d3imad_

d4f4ab_

d2vdxb_

d3ayxd_

d3ayzd_

d3myrg_

d3myrc_

d3ayyd_

d3myra_

d3mjzj_

d2ceoa_

d2wbua_

d2c2vt_

d3zvyb_

d3soub_

d3mjzd_

d3mjzh_

d3mjzb_

d3mjze_

d2vdxa_

d2v95a_

d4dy0b_

d2vdya_

d3n2sd_

d2zv6b_

d2h4ra_

d2vdyb_

d3le2a_

d3mjzg_

d3n1tf_

d3oxka_

d3oj7a_

d4egub_

d4egua_

d3ksva_

d2zpoa_

d2p6zb_

d2p7sa_

d2vq8a_

d3omfa_

d3n1sa_

d3n1te_

d3n1tb_

d3n1ta_

d3n1sm_

d3n1sj_

d3n1si_

d3n1sf_

d2p6za_

d3n1sb_

d3ljda_

d3ljdb_

d2q8td_

d3n1sn_

d2q8tb_

d2q8ta_

d2q8rh_

d2q8rg_

d2q8rf_

d2q8re_

d1zxtd_

d2q8tc_

d1zxta_

d3n1se_

d4furd_

d3ln8b_

d3ljea_

d1zxtc_

d1zxtb_

d3ln8a_

d3ohvc_

d2z8ha_

d3m52b_

d3lmba_

d2wp4b_

d2wp4a_

d4ap8d_

d3s4kb_

d4ap8c_

d4ap8b_

d4iend_

d4i83a_

d4i83c_

d4i45a_

d2gf6b_

d2gf6c_

d2gf6d_

d3rdya_

d3rdzc_

d3rdzd_

d4ap8a_

d3m52a_

d2ihcd_

d2ihcc_

d3ohua_

d3ga1b_

d2q81d_

d2q81c_

d2q81b_

d2q81a_

d3m4ta_

d3m5bb_

d3m5ba_

d3b84a_

d3fkca_

d3ohub_

d3ohud_

d2ihcb_

d2ihca_

d3ohvf_

d3ohve_

d3ohvd_

d3ohvb_

d3ohva_

d2nn2b_

d2nn2a_

d3ohuf_

d3ohue_

d3ohuc_

d3s4ka_

d2egra_

d1y7uc_

d3e29a_

d3e29b_

d3e29c_

d2egja_

d3e29d_

d4h4gi_

d3d6xa_

d1y7ub_

d2egii_

d2egih_

d2egjb_

d2egia_

d2egic_

d2egid_

d2egie_

d2egif_

d2egig_

d2if5a_

d3lqna_

d3fwsb_

d3kpdb_

d3kpdc_

d3kpdd_

d2p9md_

d3k6ea_

d3k6eb_

d3g1ba_

d3d6xe_

d3d6xf_

d2egrb_
