# Supplementary material for: CoMOGrad and PHOG: From Computer Vision to Fast and Accurate Protein Tertiary Structure Retrieval
Source: Sci Rep. 2015 Aug 21;5:13275. doi: 10.1038/srep13275 (PMC4543952; doi:10.1038/srep13275)
Supplement: Supplementary Information [file srep13275-s2.doc]

CoMOGrad and PHOG: From Computer Vision to Fast and Accurate Protein Tertiary Structure Retrieval

Rezaul Karim, Mohd. Momin Al Aziz, Swakkhar Shatabda, M. Sohel Rahman, Md. Abul Kashem Mia, Farhana Zaman and Salman Rakin

* Supplementary Table 2

Id of the structures used in the experiments as search space (All structures):

ScopID SunID

------------------

d1ux8a_ 113449

d1dlwa_ 14982

d1uvya_ 100068

d1dlya_ 14983

d1uvxa_ 100067

d2gkma_ 164742

d2gkmb_ 164743

d2gl3a_ 164754

d2gl3b_ 164755

d1idra_ 62301

d1idrb_ 62302

d1rtea_ 105096

d1rteb_ 105097

d2glna_ 164760

d2glnb_ 164761

d1s61a_ 105283

d1s61b_ 105284

d2gkna_ 164744

d2gknb_ 164745

d1s56a_ 105260

d1s56b_ 105261

d2qrwa_ 167780

d2qrwb_ 167781

d2qrwc_ 167782

d2qrwd_ 167783

d2qrwe_ 167784

d2qrwf_ 167785

d2qrwg_ 167786

d2qrwh_ 167787

d2qrwi_ 167788

d2qrwj_ 167789

d2qrwk_ 167790

d2qrwl_ 167791

d1ngka_ 85673

d1ngkb_ 85674

d1ngkc_ 85675

d1ngkd_ 85676

d1ngke_ 85677

d1ngkf_ 85678

d1ngkg_ 85679

d1ngkh_ 85680

d1ngki_ 85681

d1ngkj_ 85682

d1ngkk_ 85683

d1ngkl_ 85684

d1s69a_ 105305

d1s6aa_ 105306

d2hz1a_ 136906

d2hz3a_ 165336

d1rtxa_ 97827

d2hz2a_ 165335

d1mwba_ 79572

d2bkma_ 163114

d2bkmb_ 163115

d4l2ma_ 197344

d4l2mb_ 197421

d2bmma_ 163124

d1asha_ 15622

d1vhba_ 15627

d1vhbb_ 15628

d4vhba_ 15631

d4vhbb_ 15632

d2vhba_ 15629

d2vhbb_ 15630

d3vhba_ 15633

d3vhbb_ 15634

d1ch4a_ 15593

d1ch4b_ 15594

d1ch4c_ 15595

d1ch4d_ 15596

d1urva_ 113414

d1urvb_ 113415

d1ut0a_ 108012

d1ut0b_ 108013

d1ux9a_ 108089

d1ux9b_ 108090

d1urya_ 113416

d1uryb_ 113417

d1umoa_ 107959

d1umob_ 107960

d1v5ha_ 108375

d4hsxb_ 196961

d3lb2a_ 180152

d3lb2b_ 180153

d3kuoa_ 179718

d3kuob_ 179719

d3kuna_ 179716

d3kunb_ 179717

d3dr9a_ 174204

d3dr9b_ 174205

d2qfna_ 167567

d2qfnb_ 167568

d3k3ua_ 179048

d3k3ub_ 179049

d2qfka_ 150738

d2qfkb_ 150739

d3oj1b_ 196221

d3myma_ 181700

d3mymb_ 181701

d3moua_ 181469

d3moub_ 181470

d3lb1a_ 180150

d3lb1b_ 180151

d1ew6a_ 15637

d1ew6b_ 15638

d3lb3a_ 180154

d3lb3b_ 180155

d3lb4a_ 180156

d3lb4b_ 180157

d3ok5a_ 183072

d3ok5b_ 183073

d3o7nb_ 196329

d4fh7a_ 196894

d1ewaa_ 15639

d1ewab_ 15640

d3myna_ 181702

d3mynb_ 181703

d1ecoa_ 15208

d1ecda_ 15211

d1ecaa_ 15209

d1ecna_ 15210

d1x9fd_ 114986

d1x9fh_ 114990

d1x9fl_ 114994

d2gtld1 135658

d2gtlh1 135662

d2gtll1 135666

d1x9fb_ 114984

d1x9ff_ 114988

d1x9fj_ 114992

d2gtlb1 135656

d2gtlf1 135660

d2gtlj1 135664

d1x9fc_ 114985

d1x9fg_ 114989

d1x9fk_ 114993

d2gtlc1 135657

d2gtlg1 135661

d2gtlk1 135665

d1x9fa_ 114983

d1x9fe_ 114987

d1x9fi_ 114991

d2gtla1 135655

d2gtle1 135659

d2gtli1 135663

d1cqxa1 15635

d1cqxb1 15636

d1gvha1 70601

d1jl7a_ 71726

d1jl6a_ 71725

d1jf4a_ 71643

d2hbga_ 15014

d1jf3a_ 71642

d1hbga_ 15015

d1vrfa_ 15016

d1vrea_ 15017

d1it2a_ 66365

d1it2b_ 66366

d1it3a_ 66367

d1it3b_ 66368

d1it3c_ 66369

d1it3d_ 66370

d1or4a_ 93447

d1or4b_ 93448

d1or6a_ 93449

d1or6b_ 93450

d1itha_ 15623

d1ithb_ 15624

d3g46a_ 176334

d3g46b_ 176335

d2z8aa_ 171102

d2z8ab_ 171103

d4hrra_ 192675

d4hrrc_ 192917

d4hrre_ 192918

d4hrrg_ 192919

d4hrta_ 192915

d4hrtc_ 192914

d4hrte_ 192570

d4hrtg_ 192916

d3sdha_ 14984

d3sdhb_ 14985

d2grza_ 164825

d2grzb_ 164826

d2grha_ 164813

d2grhb_ 164814

d2av0a_ 127347

d2av0b_ 127348

d2auoa_ 162900

d2auob_ 162901

d1jzla_ 67865

d1jzlb_ 67866

d4sdha_ 14988

d4sdhb_ 14989

d2z85a_ 171100

d2z85b_ 171101

d4hbia_ 14990

d4hbib_ 14991

d7hbia_ 14994

d7hbib_ 14995

d5hbia_ 14992

d5hbib_ 14993

d3g4ra_ 176352

d3g4rb_ 176353

d3g4qa_ 176350

d3g4qb_ 176351

d3g53a_ 176373

d3g53b_ 176374

d1hbia_ 14996

d1hbib_ 14997

d3g4ya_ 176363

d3g4yb_ 176364

d3g52a_ 176371

d3g52b_ 176372

d2av3a_ 127355

d2av3b_ 127356

d2r4za_ 167983

d2r4zb_ 167984

d6hbia_ 15000

d6hbib_ 15001

d1jzma_ 67867

d1jzmb_ 67868

d1nxfa_ 92298

d1nxfb_ 92299

d2r4wa_ 167977

d2r4wb_ 167978

d3g4wa_ 176358

d3g4wb_ 176359

d2r4ya_ 167981

d2r4yb_ 167982

d2r4xa_ 167979

d2r4xb_ 167980

d3g4ua_ 176354

d3g4ub_ 176355

d1jzka_ 67861

d1jzkb_ 67862

d1jzkc_ 67863

d1jzkd_ 67864

d3g4va_ 176356

d3g4vb_ 176357

d1jwna_ 67394

d1jwnb_ 67395

d1jwnc_ 67396

d1jwnd_ 67397

d1nwia_ 92237

d1nwib_ 92238

d1nwic_ 92239

d1nwid_ 92240

d1nwna_ 92243

d1nwnb_ 92244

d1b0ba_ 15010

d1flpa_ 15011

d1ebta_ 15013

d1moha_ 15012

d1wmua_ 109416

d2z6na_ 154180

d1v75a_ 100444

d3d1ka_ 157208

d2aa1a_ 126463

d2aa1c_ 126464

d1la6a_ 73782

d3nfea_ 182228

d3nfec_ 182230

d1t1na_ 15408

d3ng6a_ 182254

d3ng6c_ 182256

d1a4fa_ 15394

d1c40a_ 15395

d1hv4a_ 15396

d1hv4c_ 15397

d1hv4e_ 15398

d1hv4g_ 15399

d1v4xa_ 108363

d1v4xc_ 108365

d1v4wa_ 108359

d1v4wc_ 108361

d1v4ua_ 108355

d1v4uc_ 108357

d1cg5a_ 15406

d1cg8a_ 15407

d1hbra_ 15392

d1hbrc_ 15393

d2qssa_ 151322

d2qssc_ 151324

d2qspa_ 151318

d2qspc_ 151320

d1g08a_ 15380

d1g08c_ 15381

d3piaa_ 183767

d3piac_ 183769

d1g0aa_ 15382

d1g0ac_ 15383

d1g09a_ 15384

d1g09c_ 15385

d1fsxa_ 60012

d1fsxc_ 60014

d3pi8a_ 183759

d3pi8c_ 183761

d1hdaa_ 15386

d1hdac_ 15387

d3pi9a_ 183763

d3pi9c_ 183765

d3ciua1 156693

d3ciuc1 156695

d1hdsa_ 15378

d1hdsc_ 15379

d3pela_ 183685

d1s0ha1 144372

d2b7ha_ 128036

d2b7hb1 144965

d2b7hc_ 128037

d2h8fa_ 136239

d2h8fc_ 136241

d3gkva_ 176726

d2pega_ 149403

d3gqga_ 176909

d3gqgc_ 176911

d2h8da_ 136235

d2h8dc_ 136237

d1hbha_ 15404

d1hbhc_ 15405

d1s5xa_ 98585

d1pbxa_ 15403

d4iroc_ 193244

d1s5ya_ 98587

d1s5yc_ 98589

d1spga_ 15409

d1fawa_ 65002

d1fawc_ 65004

d2d5xa_ 131282

d1iwha_ 76882

d1ns9a_ 92095

d1ibea_ 15374

d1g0ba_ 15375

d1ns6a_ 92093

d2zlta_ 154668

d2zlua_ 154670

d2zlva_ 154672

d2mhba_ 15376

d1y8ka_ 122753

d1y8kc_ 122755

d1y8ia_ 122748

d1y8ic_ 122750

d1y8ha1 122744

d1y8hc1 122746

d2zlxa_ 154678

d2zlxc_ 154680

d2dhba_ 15377

d2zlwa_ 154674

d2zlwc_ 154676

d1gcva_ 15410

d1gcvc_ 15411

d1gcwa_ 15412

d1gcwc_ 15413

d1irda_ 66286

d2dn3a_ 131583

d2dn1a_ 131577

d2dn2a_ 131579

d2dn2c_ 131581

d1j40a_ 84096

d1j40c_ 84098

d1j40e_ 84100

d1j40g_ 84102

d1j41a_ 84104

d1j41c_ 84106

d1j41e_ 84108

d1j41g_ 84110

d2d5za_ 131284

d2d5zc_ 131286

d1uiwa_ 99438

d1uiwc_ 99440

d1uiwe_ 99442

d1uiwg_ 99444

d1baba_ 15235

d1babc_ 15236

d3s66a_ 193673

d1j3ya_ 84080

d1j3yc_ 84082

d1j3ye_ 84084

d1j3yg_ 84086

d1bz0a_ 15237

d1bz0c_ 15238

d3qjda_ 192774

d3qjdc_ 184422

d1j3za_ 84088

d1j3zc_ 84090

d1j3ze_ 84092

d1j3zg_ 84094

d3nmma_ 182408

d3nmmc_ 182410

d1bzza_ 15239

d1bzzc_ 15240

d1bz1a_ 15241

d1bz1c_ 15242

d1j7ya_ 66426

d1j7yc_ 66428

d1thba_ 15243

d1thbc_ 15244

d1dxva_ 15259

d1dxvc_ 15260

d1dxua_ 15269

d1dxuc_ 15270

d1dxta_ 15267

d1dxtc_ 15268

d2hhba_ 15245

d2hhbc_ 15246

d2w6va_ 169077

d2w6vc_ 169079

d4hhba_ 15251

d4hhbc_ 15252

d1qsha_ 15249

d1qshc_ 15250

d1nqpa_ 92051

d1nqpc_ 92053

d1a3na_ 15247

d1a3nc_ 15248

d3qjba_ 184417

d1bbba_ 15255

d1bbbc_ 15256

d3duta_ 174246

d3dutc_ 174248

d1qsia_ 15257

d1qsic_ 15258

d1c7ca1 15261

d1c7ca2 15262

d1sdla_ 15253

d1sdlc_ 15254

d3nl7a_ 182354

d1o1oa_ 81068

d1o1oc_ 81070

d1sdka_ 15265

d1sdkc_ 15266

d1yvqa_ 124112

d1yvqc_ 124114

d1qi8a_ 15263

d1qi8c_ 15264

d1shra_ 112083

d1shrc_ 112085

d1yvta_ 124118

d1a01a_ 15271

d1a01c_ 15272

d1o1pa1 81072

d1o1pa2 81073

d3hhba_ 15273

d3hhbc_ 118676

d1o1na1 81064

d1o1na2 81065

d1a3oa_ 15274

d1a3oc_ 15275

d1o1ja1 81048

d1o1ja2 81049

d1qxea_ 96523

d1qxec_ 96525

d1ye2a_ 116640

d1ye2c_ 116642

d1k0ya_ 67987

d1k0yc_ 67989

d1xz2a_ 116237

d1xz2c_ 116239

d1r1ya_ 96836

d1r1yc_ 96838

d1c7ba_ 15276

d1c7bc_ 15277

d2d60a_ 131288

d2d60c_ 131290

d1g9va_ 15280

d1g9vc_ 15281

d1c7da1 15278

d1c7da2 15279

d1gbua_ 15284

d1gbuc_ 15285

d1rq3a_ 97722

d1rq3c_ 97724

d3ic0a_ 178230

d3ic0c_ 178232

d3qjea_ 184424

d3qjec_ 184426

d1vwta_ 15282

d1vwtc_ 15283

d1xz7a_ 116250

d1xz7c_ 116252

d3d7oa_ 173744

d1o1la1 81056

d1o1la2 81057

d1abwa1 15288

d1abwa2 15289

d1o1ma1 81060

d1o1ma2 81061

d1y4va_ 116466

d1y4vc_ 116468

d1clsa_ 15292

d1clsc_ 15293

d1a00a_ 15290

d1a00c_ 15291

d3oo4a_ 183198

d1buwa_ 15294

d1buwc_ 15295

d3qjca_ 184419

d1y45a_ 116432

d1y45c_ 116434

d1hbba_ 15300

d1hbbc_ 15301

d2hbsa_ 15296

d2hbsc_ 15297

d2hbse_ 15298

d2hbsg_ 15299

d1y4ga_ 116448

d1y4gc_ 116450

d1xxta_ 116207

d1xxtc_ 116209

d1y4fa_ 116444

d1y4fc_ 116446

d1xz4a_ 116241

d1xz4c_ 116243

d1kd2a_ 84382

d1kd2c_ 84384

d1a0za_ 15302

d1a0zc_ 15303

d1j7wa_ 66421

d1j7wc_ 66423

d1y83a_ 116552

d1y83c_ 116554

d1y4pa_ 116452

d1y4pc_ 116454

d1y7za_ 116548

d1y7zc_ 116550

d1o1ka_ 81052

d1o1kc_ 81054

d1yhea_ 123172

d1yhec_ 123174

d3p5qa_ 183540

d1y0wa_ 116312

d1y0wc_ 116314

d6hbwa_ 15304

d6hbwc_ 15305

d1y7da_ 116528

d1y7dc_ 116530

d1yiha_ 123293

d1yihc_ 123294

d1k1ka_ 77227

d1j7sa_ 66417

d1j7sc_ 66419

d2hhda_ 15312

d2hhdc_ 15313

d1xy0a_ 116211

d1xy0c_ 116213

d1y5ja_ 116476

d1y5jc_ 116478

d2dxma_ 161407

d2dxmc_ 161408

d1rpsa_ 97712

d1rpsc_ 97714

d1gbva_ 15310

d1gbvc_ 15311

d1hbaa_ 15316

d1hbac_ 15317

d1y2za_ 116419

d1y2zc_ 116421

d1hdba_ 15320

d1hdbc_ 15321

d1y09a_ 116281

d1y09c_ 116283

d1xzva_ 116266

d1xzvc_ 116268

d1rqaa_ 111910

d1rqac_ 111912

d1y4ba_ 116440

d1y4bc_ 116442

d1y0da_ 116293

d1y0dc_ 116295

d1y85a_ 116556

d1y85c_ 116558

d1y0ta_ 116306

d1y0tc_ 116308

d1xz5a_ 116245

d1xz5c_ 116247

d1y4qa_ 116456

d1y4qc_ 116458

d1y35a_ 116427

d1y35c_ 116429

d1rq4a_ 97726

d1rq4c_ 97728

d1y5fa_ 116472

d1y5fc_ 116474

d2hbca_ 15324

d1a0ua_ 15322

d1a0uc_ 15323

d2hbea_ 15329

d1yeoa_ 123012

d1yeoc_ 123014

d1y5ka_ 116480

d1y5kc_ 116482

d1y22a_ 116390

d1y22c_ 116392

d1y31a_ 116423

d1y31c_ 116425

d1hgaa_ 15335

d1hgac_ 15336

d1xzua_ 116262

d1xzuc_ 116264

d1y4ra_ 116460

d1y4rc_ 116462

d1y0aa_ 116285

d1y0ac_ 116287

d1xyea_ 116217

d1xyec_ 116219

d1yeva_ 116660

d1yevc_ 116662

d1yzia_ 124279

d1dkea_ 15333

d1dkec_ 15334

d3hxna_ 177905

d3hxnc_ 177907

d1ljwa_ 73945

d3onza_ 183195

d1hgba_ 15339

d1hgbc_ 15340

d1aj9a_ 15332

d1y7ga_ 116534

d1y7gc_ 116536

d2yrsa_ 170872

d2yrsc_ 170874

d2yrsi_ 170876

d2yrsm_ 170878

d2hhea_ 15337

d2hhec_ 15338

d1yeua_ 116656

d1yeuc_ 116658

d1r1xa_ 96834

d1yh9a_ 123164

d1yh9c_ 123166

d1y0ca_ 116289

d1y0cc_ 116291

d3r5ia_ 184810

d3r5ic_ 184812

d1hgca_ 15343

d1hgcc_ 15344

d1y46a_ 116436

d1y46c_ 116438

d1mkoa_ 79246

d1mkoc_ 79248

d1gzxa_ 70825

d1gzxc_ 70827

d4fc3a_ 197171

d3oo5a_ 183200

d2hbda_ 15342

d2hbfa_ 15341

d1o1ia_ 81046

d1y7ca_ 116524

d1y7cc_ 116526

d3kmfa_ 179444

d3kmfe_ 179446

d1m9pa_ 91237

d1m9pc_ 91239

d1si4a_ 112087

d1si4c_ 112089

d1hhoa_ 15347

d1bija_ 15345

d1bijc_ 15346

d1neja_ 91845

d1nejc_ 91847

d1qxda_ 96519

d1qxdc_ 96521

d3ic2a_ 178234

d3ic2c_ 178236

d1yiea_ 123279

d1yiec_ 123281

d1abya1 15349

d1abya2 15350

d1rvwa_ 15348

d1b86a_ 15361

d1b86c_ 15362

d1ye0a_ 116632

d1ye0c_ 116634

d1z8ub_ 124725

d1z8ud_ 124727

d1yffa_ 116672

d1yffc_ 116674

d1yffe_ 116676

d1yffg_ 116678

d1haba_ 15351

d1habc_ 15352

d1yhra_ 123209

d1yhrc_ 123211

d1glia_ 15353

d1glic_ 15354

d1lfta_ 77937

d1haca_ 15355

d1hacc_ 15356

d1lfqa_ 77935

d1lfla_ 77927

d1lflc_ 77929

d1lflp_ 77931

d1lflr_ 77933

d3b75a_ 161604

d3b75c_ 161605

d3b75e_ 161606

d3b75g_ 161607

d3b75s_ 161608

d1ygfa_ 116690

d1ygfc_ 116692

d1a9wa_ 15359

d1a9wc_ 15360

d1niha_ 15357

d1nihc_ 15358

d1ygda_ 116686

d1ygdc_ 116688

d1yena_ 116648

d1yenc_ 116650

d1yeqa_ 116652

d1yeqc_ 116654

d1yg5a_ 116682

d1yg5c_ 116684

d1ydza_ 116628

d1ydzc_ 116630

d1y8wa_ 116564

d1y8wc_ 116566

d1lfva_ 77940

d1coha_ 15364

d1cohc_ 15365

d1fdha_ 15363

d1fdhb_ 118454

d1jy7a_ 71944

d1jy7c_ 71946

d1jy7p_ 71948

d1jy7r_ 71950

d1jy7u_ 71952

d1jy7w_ 71954

d3d17a_ 161634

d3d17c_ 161635

d2hcoa_ 15366

d1lfza_ 77946

d1lfya_ 77944

d1fn3a_ 90494

d1fn3c_ 90496

d1y01b_ 116277

d1hcoa_ 15367

d1cmya_ 15368

d1cmyc_ 15369

d1hbsa_ 15370

d1hbsc_ 15371

d1hbse_ 15372

d1hbsg_ 15373

d1ye1a_ 116636

d1ye1c_ 116638

d2h35a1 136028

d2h35c1 136030

d3w4ua_ 194017

d3w4uc_ 194018

d3w4ue_ 194016

d1jeba_ 66591

d1jebc_ 66593

d1fhja_ 59837

d1fhjc_ 59839

d4h2la_ 196780

d1qpwa_ 15390

d1qpwc_ 15391

d2pgha_ 15388

d2pghc_ 15389

d4f4oa_ 192417

d4f4od_ 192418

d4f4og_ 192419

d4f4oj_ 192420

d1outa_ 15400

d1ouua_ 15401

d1ouuc_ 15402

d1xq5a_ 115825

d1xq5c_ 115827

d1wmub_ 109417

d2z6nb_ 154181

d1v75b_ 100445

d3d1kb_ 157209

d2aa1b1 144793

d2aa1d_ 144794

d1la6b_ 73783

d3nfeb_ 182229

d3nfed_ 182231

d1t1nb_ 15587

d3ng6b_ 182255

d3ng6d_ 182257

d1a4fb_ 15573

d1c40b_ 15574

d1hv4b_ 15575

d1hv4d_ 15576

d1hv4f_ 15577

d1hv4h_ 15578

d1v4xb_ 108364

d1v4xd_ 108366

d1v4wb_ 108360

d1v4wd_ 108362

d1v4ub_ 108356

d1v4ud_ 108358

d1cg5b_ 15585

d1cg8b_ 15586

d1hbrb_ 15571

d1hbrd_ 15572

d2qssb_ 151323

d2qssd_ 151325

d2qspb_ 151319

d2qspd_ 151321

d1g08b_ 15559

d1g08d_ 15560

d3piab_ 183768

d3piad_ 183770

d1g0ab_ 15561

d1g0ad_ 15562

d1g09b_ 15563

d1g09d_ 15564

d1fsxb_ 60013

d1fsxd_ 60015

d3pi8b_ 183760

d3pi8d_ 183762

d1hdab_ 15565

d1hdad_ 15566

d3pi9b_ 183764

d3pi9d_ 183766

d3ciub1 156694

d3ciud1 156696

d1hdsb_ 15557

d1hdsd_ 15558

d3pelb_ 183686

d2qlsb1 150864

d2qlsd1 150865

d1s0hb1 118837

d2h8fb_ 136240

d2h8fd_ 136242

d3gkvb_ 176727

d2pegb_ 149404

d3gqgb_ 176910

d3gqgd_ 176912

d2h8db_ 136236

d2h8dd_ 136238

d1hbhb_ 15583

d1hbhd_ 15584

d1s5xb_ 98586

d1pbxb_ 15582

d4irob_ 193973

d4irod_ 193972

d1s5yb_ 98588

d1s5yd_ 98590

d1spgb_ 15588

d1fawb_ 65003

d1fawd_ 65005

d2d5xb_ 131283

d1iwhb_ 76883

d1ns9b_ 92096

d1ibeb_ 15553

d1g0bb_ 15554

d1ns6b_ 92094

d2zltb_ 154669

d2zlub_ 154671

d2zlvb_ 154673

d2mhbb_ 15555

d1y8kb_ 122754

d1y8kd_ 122756

d1y8ib_ 122749

d1y8id_ 122751

d1y8hb1 122745

d1y8hd1 122747

d2zlxb_ 154679

d2zlxd_ 154681

d2dhbb_ 15556

d2zlwb_ 154675

d2zlwd_ 154677

d1gcvb_ 15589

d1gcvd_ 15590

d1gcwb_ 15591

d1gcwd_ 15592

d2w72b_ 169084

d2w72d_ 169086

d1irdb_ 66287

d2dn3b_ 131584

d2dn1b_ 131578

d2dn2b_ 131580

d2dn2d_ 131582

d1j40b_ 84097

d1j40d_ 84099

d1j40f_ 84101

d1j40h_ 84103

d1j41b_ 84105

d1j41d_ 84107

d1j41f_ 84109

d1j41h_ 84111

d2d5zb_ 131285

d2d5zd_ 131287

d1uiwb_ 99439

d1uiwd_ 99441

d1uiwf_ 99443

d1uiwh_ 99445

d1babb_ 15414

d1babd_ 15415

d3s66b_ 192017

d1j3yb_ 84081

d1j3yd_ 84083

d1j3yf_ 84085

d1j3yh_ 84087

d1bz0b_ 15416

d1bz0d_ 15417

d3qjdb_ 184421

d3qjdd_ 184423

d1j3zb_ 84089

d1j3zd_ 84091

d1j3zf_ 84093

d1j3zh_ 84095

d3nmmb_ 182409

d3nmmd_ 182411

d1bzzb_ 15418

d1bzzd_ 15419

d1bz1b_ 15420

d1bz1d_ 15421

d1j7yb_ 66427

d1j7yd_ 66429

d1thbb_ 15422

d1thbd_ 15423

d1dxvb_ 15436

d1dxvd_ 15437

d1dxub_ 15442

d1dxud_ 15443

d1dxtb_ 15450

d1dxtd_ 15451

d2hhbb_ 15424

d2hhbd_ 15425

d2w6vb_ 169078

d2w6vd_ 169080

d4hhbb_ 15428

d4hhbd_ 15429

d1qshb_ 15426

d1qshd_ 15427

d1nqpb_ 92052

d1nqpd_ 92054

d1a3nb_ 15430

d1a3nd_ 15431

d3qjbb_ 184418

d1bbbb_ 15434

d1bbbd_ 15435

d3dutb_ 174247

d3dutd_ 174249

d1qsib_ 15440

d1qsid_ 15441

d1c7cb_ 15438

d1c7cd_ 15439

d1sdlb_ 15432

d1sdld_ 15433

d3nl7b_ 182355

d1o1ob_ 81069

d1o1od_ 81071

d1sdkb_ 15444

d1sdkd_ 15445

d1yvqb_ 124113

d1yvqd_ 124115

d1qi8b_ 15446

d1qi8d_ 15447

d1yvtb_ 124119

d1a01b_ 15448

d1a01d_ 15449

d1o1pb_ 81074

d1o1pd_ 81075

d3hhbb_ 15452

d3hhbd_ 118677

d1o1nb_ 81066

d1o1nd_ 81067

d1cbma_ 15286

d1cbmb_ 15465

d1cbmc_ 15287

d1cbmd_ 15466

d1a3ob_ 15453

d1a3od_ 15454

d1o1jb_ 81050

d1o1jd_ 81051

d1qxeb_ 96524

d1qxed_ 96526

d1ye2b_ 116641

d1ye2d_ 116643

d1k0yb_ 67988

d1k0yd_ 67990

d1xz2b_ 116238

d1xz2d_ 116240

d1r1yb_ 96837

d1r1yd_ 96839

d1c7bb_ 15455

d1c7bd_ 15456

d2d60b_ 131289

d2d60d_ 131291

d1g9vb_ 15459

d1g9vd_ 15460

d1c7db_ 15457

d1c7dd_ 15458

d1gbub_ 15461

d1gbud_ 15462

d1rq3b_ 97723

d1rq3d_ 97725

d3ic0b_ 178231

d3ic0d_ 178233

d3qjeb_ 184425

d3qjed_ 184427

d1vwtb_ 15463

d1vwtd_ 15464

d1xz7b_ 116251

d1xz7d_ 116253

d3d7ob_ 173745

d1o1lb_ 81058

d1o1ld_ 81059

d1abwb_ 15467

d1abwd_ 15468

d1o1mb_ 81062

d1o1md_ 81063

d1y4vb_ 116467

d1y4vd_ 116469

d1clsb_ 15471

d1clsd_ 15472

d1cbla_ 15314

d1cblb_ 15491

d1cblc_ 15315

d1cbld_ 15492

d1a00b_ 15469

d1a00d_ 15470

d3oo4b_ 183199

d1buwb_ 15475

d1buwd_ 15476

d3qjcb_ 184420

d1y45b_ 116433

d1y45d_ 116435

d1hbbb_ 15473

d1hbbd_ 15474

d2hbsb_ 15477

d2hbsd_ 15478

d2hbsf_ 15479

d2hbsh_ 15480

d1y4gb_ 116449

d1y4gd_ 116451

d1xxtb_ 116208

d1xxtd_ 116210

d1y4fb_ 116445

d1y4fd_ 116447

d1xz4b_ 116242

d1xz4d_ 116244

d1kd2b_ 84383

d1kd2d_ 84385

d1a0zb_ 15481

d1a0zd_ 15482

d3w4ub_ 192624

d3w4ud_ 192859

d3w4uf_ 192860

d1j7wb_ 66422

d1j7wd_ 66424

d1y83b_ 116553

d1y83d_ 116555

d1y4pb_ 116453

d1y4pd_ 116455

d1y7zb_ 116549

d1y7zd_ 116551

d1o1kb_ 81053

d1o1kd_ 81055

d1yheb_ 123173

d1yhed_ 123175

d3p5qb_ 183541

d1y0wb_ 116313

d1y0wd_ 116315

d6hbwb_ 15483

d6hbwd_ 15484

d1y7db_ 116529

d1y7dd_ 116531

d1yihb1 144640

d1yihd_ 144641

d1k1kb_ 77228

d1j7sb_ 66418

d1j7sd_ 66420

d2hhdb_ 15489

d2hhdd_ 15490

d1xy0b_ 116212

d1xy0d_ 116214

d1y5jb_ 116477

d1y5jd_ 116479

d2dxmb_ 146603

d2dxmd_ 146604

d1rpsb_ 97713

d1rpsd_ 97715

d1gbvb_ 15487

d1gbvd_ 15488

d1hbab_ 15497

d1hbad_ 15498

d1y2zb_ 116420

d1y2zd_ 116422

d1hdbb_ 15499

d1hdbd_ 15500

d1y09b_ 116282

d1y09d_ 116284

d1xzvb_ 116267

d1xzvd_ 116269

d1rqab_ 111911

d1rqad_ 111913

d1y4bb_ 116441

d1y4bd_ 116443

d1y0db_ 116294

d1y0dd_ 116296

d1y85b_ 116557

d1y85d_ 116559

d3s65d_ 192016

d1y0tb_ 116307

d1y0td_ 116309

d1xz5b_ 116246

d1xz5d_ 116248

d1y4qb_ 116457

d1y4qd_ 116459

d1y35b_ 116428

d1y35d_ 116430

d1rq4b_ 97727

d1rq4d_ 97729

d1y5fb_ 116473

d1y5fd_ 116475

d2hbcb_ 15501

d1a0ub_ 15506

d1a0ud_ 15507

d2hbeb_ 15510

d1yeob_ 123013

d1yeod_ 123015

d1y5kb_ 116481

d1y5kd_ 116483

d1y22b_ 116391

d1y22d_ 116393

d1y31b_ 116424

d1y31d_ 116426

d1hgab_ 15514

d1hgad_ 15515

d1xzub_ 116263

d1xzud_ 116265

d1y4rb_ 116461

d1y4rd_ 116463

d1y0ab_ 116286

d1y0ad_ 116288

d1xyeb_ 116218

d1xyed_ 116220

d1yevb_ 116661

d1yevd_ 116663

d1yzib_ 124280

d1dkeb_ 15512

d1dked_ 15513

d3hxnb_ 177906

d3hxnd_ 177908

d1ljwb_ 73946

d3onzb_ 183196

d1hgbb_ 15518

d1hgbd_ 15519

d1aj9b_ 15511

d1y7gb_ 116535

d1y7gd_ 116537

d2hheb_ 15516

d2hhed_ 15517

d1yeub_ 116657

d1yeud_ 116659

d1r1xb_ 96835

d1yh9b_ 123165

d1yh9d_ 123167

d1y0cb_ 116290

d1y0cd_ 116292

d3r5ib_ 184811

d3r5id_ 184813

d1hgcb_ 15522

d1hgcd_ 15523

d1y46b_ 116437

d1y46d_ 116439

d1mkob_ 79247

d1mkod_ 79249

d1gzxb_ 70826

d1gzxd_ 70828

d4fc3b_ 197215

d3oo5b_ 183201

d2hbdb_ 15520

d2hbfb_ 15521

d1o1ib_ 81047

d1y7cb_ 116525

d1y7cd_ 116527

d3kmfc_ 179445

d3kmfg_ 179447

d1m9pb_ 91238

d1m9pd_ 91240

d1hhob_ 15526

d1bijb_ 15524

d1bijd_ 15525

d1nejb_ 91846

d1nejd_ 91848

d1qxdb_ 96520

d1qxdd_ 96522

d3ic2b_ 178235

d3ic2d_ 178237

d1yieb_ 123280

d1yied_ 123282

d1abyb_ 15527

d1abyd_ 15528

d1rvwb_ 15529

d1b86b_ 15538

d1b86d_ 15539

d1ye0b_ 116633

d1ye0d_ 116635

d1yffb_ 116673

d1yffd_ 116675

d1yfff_ 116677

d1yffh_ 116679

d1habb_ 15530

d1habd_ 15531

d1yhrb_ 123210

d1yhrd_ 123212

d1glib_ 15534

d1glid_ 15535

d1lftb_ 77938

d1hacb_ 15532

d1hacd_ 15533

d1lfqb_ 77936

d1lflb_ 77928

d1lfld_ 77930

d1lflq_ 77932

d1lfls_ 77934

d3b75b_ 154916

d3b75d_ 154917

d3b75f_ 154918

d3b75h_ 154919

d3b75t_ 154920

d1ygfb_ 116691

d1ygfd_ 116693

d1nihb_ 15536

d1nihd_ 15537

d1ygdb_ 116687

d1ygdd_ 116689

d1yenb_ 116649

d1yend_ 116651

d1yeqb_ 116653

d1yeqd_ 116655

d1yg5b_ 116683

d1yg5d_ 116685

d1ydzb_ 116629

d1ydzd_ 116631

d1y8wb_ 116565

d1y8wd_ 116567

d1lfvb_ 77941

d1cohb_ 15540

d1cohd_ 15541

d1jy7b_ 71945

d1jy7d_ 71947

d1jy7q_ 71949

d1jy7s_ 71951

d1jy7v_ 71953

d1jy7x_ 71955

d3d17b_ 157174

d3d17d_ 157175

d2hcob_ 15542

d1lfzb_ 77947

d1lfyb_ 77945

d1fn3b_ 90495

d1fn3d_ 90497

d1hcob_ 15543

d1cmyb_ 15544

d1cmyd_ 15545

d1hbsb_ 15546

d1hbsd_ 15547

d1hbsf_ 15548

d1hbsh_ 15549

d1ye1b_ 116637

d1ye1d_ 116639

d2h35b1 136029

d2h35d1 136031

d1shrb_ 112084

d1shrd_ 112086

d1si4b_ 112088

d1si4d_ 112090

d1a9we_ 15551

d1a9wf_ 15552

d1i3da_ 61587

d1i3db_ 61588

d1i3ea_ 61589

d1i3eb_ 61590

d1fdhg_ 15550

d1fdhh_ 118455

d1fhjb_ 59838

d1fhjd_ 59840

d1jebb_ 66592

d1jebd_ 66594

d1qpwb_ 15569

d1qpwd_ 15570

d2pghb_ 15567

d2pghd_ 15568

d1outb_ 15579

d1ouub_ 15580

d1ouud_ 15581

d3bj1b_ 155323

d3bj1d_ 155324

d3bj2b_ 155325

d3bj2d_ 155326

d3bj3b_ 155327

d3bj3d_ 155328

d1xq5b_ 115826

d1xq5d_ 115828

d1hlba_ 15625

d1hlma_ 15626

d1tu9a_ 107320

d1uc3a_ 88438

d1uc3b_ 88439

d1uc3c_ 88440

d1uc3d_ 88441

d1uc3e_ 88442

d1uc3f_ 88443

d1uc3g_ 88444

d1uc3h_ 88445

d1uc3i_ 88446

d1uc3j_ 88447

d1uc3k_ 88448

d1uc3l_ 88449

d2lhba_ 15597

d3lhba_ 15604

d3lhbb_ 15605

d3lhbc_ 15606

d3lhbd_ 15607

d3lhbe_ 15608

d3lhbf_ 15609

d3lhbg_ 15610

d3lhbh_ 15611

d3lhbi_ 15612

d3lhbj_ 15613

d3lhbk_ 15614

d3lhbl_ 15615

d1f5oa_ 15598

d1f5ob_ 15599

d1f5oc_ 15600

d1f5od_ 15601

d1f5oe_ 15602

d1f5of_ 15603

d1f5pa_ 15616

d1f5pb_ 15617

d1f5pc_ 15618

d1f5pd_ 15619

d1f5pe_ 15620

d1f5pf_ 15621

d1fsla_ 15229

d1fslb_ 15230

d1bina_ 15231

d1binb_ 15232

d2gdma_ 15212

d1gdja_ 15213

d1gdia_ 15214

d1gdla_ 15216

d1gdka_ 15215

d2lh1a_ 15217

d2lh2a_ 15220

d2lh5a_ 15219

d2lh7a_ 15218

d2lh3a_ 15224

d1lh1a_ 15222

d2lh6a_ 15225

d1lh2a_ 15226

d1lh5a_ 15227

d1lh7a_ 15223

d1lh3a_ 15221

d1lh6a_ 15228

d1emya_ 15204

d1mbsa_ 15156

d3vm9a_ 192807

d3vm9b_ 192478

d2v1fa_ 140055

d2v1ia_ 140058

d2v1ka_ 140060

d2frfa_ 133987

d2v1ha_ 140057

d3rj6a_ 195447

d3rj6b_ 195448

d2vlxa_ 168693

d2v1ea_ 140054

d2v1ga_ 140056

d2frkx_ 133992

d2frjx_ 133991

d2v1ja_ 140059

d1gjna_ 70191

d2vlza_ 153310

d1dwta_ 15190

d1dwsa_ 15191

d4dc7a_ 197082

d1dwra_ 15192

d4dc8a_ 197081

d3lr9a_ 180524

d2vm0a_ 153311

d2frix_ 133990

d2vlya_ 153309

d2o5sx_ 148614

d1hrma_ 15193

d3lr7a_ 180523

d3v2za_ 192556

d2o5ox_ 148612

d2o5tx_ 148615

d2o58x_ 148592

d1nz3a_ 86438

d2o5mx_ 148611

d2o5lx_ 148610

d3v2va_ 192558

d3vaua_ 192199

d1wlaa_ 15195

d1xcha_ 15194

d1rsea_ 15196

d1nz5a_ 86440

d1npga_ 92037

d2nsra_ 148390

d3ba2a_ 172513

d3rjnb_ 195446

d3hena_ 177453

d1nz2a_ 86437

d1bjea_ 15197

d1nz4a_ 86439

d1hsya_ 15198

d1npfa_ 92036

d2o5qx_ 148613

d1ymba_ 15199

d3hc9a_ 177365

d1ymca_ 15200

d3hepa_ 177455

d2o5bx_ 148595

d2nssa_ 148391

d3heoa_ 177454

d1azia_ 15201

d1ymaa_ 15202

d2in4a_ 137523

d3rgka_ 184952

d1lhta_ 15205

d1lhsa_ 15206

d1mwca_ 15157

d1mwcb_ 15158

d1mwda_ 15159

d1mwdb_ 15160

d1myga_ 15161

d1mygb_ 15162

d1m6ma_ 15163

d1m6mb_ 15164

d1m6ca_ 15165

d1m6cb_ 15166

d1mnoa_ 15167

d1mnob_ 15168

d1myja_ 15171

d1myjb_ 15172

d1mdna_ 15169

d1mdnb_ 15170

d1mnia_ 15173

d1mnib_ 15174

d1myia_ 15175

d1myib_ 15176

d1mnja_ 15179

d1mnjb_ 15180

d1mnka_ 15181

d1mnkb_ 15182

d1myha_ 15177

d1myhb_ 15178

d1ycba_ 15183

d1ycbb_ 15184

d1mnha_ 15187

d1pmba_ 15188

d1pmbb_ 15189

d1ycaa_ 15185

d1ycab_ 15186

d1mbaa_ 15149

d2fala_ 15150

d5mbaa_ 15151

d3mbaa_ 15152

d1dm1a_ 15153

d2fama_ 15154

d4mbaa_ 15155

d1a6ma_ 15018

d1naza_ 85505

d1a6ka_ 15019

d2ekta_ 146883

d1bzpa_ 15020

d4h07a_ 192549

d1a6na_ 15022

d1bzra_ 15023

d1bz6a_ 15024

d1a6ga_ 15021

d3o89a_ 182875

d1u7ra_ 119621

d2z6ta_ 154183

d2zspa_ 171476

d2zsra_ 171478

d3edba_ 174864

d3e4na_ 174656

d3u3ea_ 192567

d3ecza_ 174849

d3ecla_ 174829

d2zt2a_ 171498

d2zt3a_ 171499

d2zt4a_ 171500

d4h0ba_ 192548

d2zssa_ 171479

d3e5ia_ 174672

d3edaa_ 174863

d2zsoa_ 171475

d3e55a_ 174669

d2zsza_ 171495

d2zt1a_ 171497

d2zt0a_ 171496

d2zsna_ 171474

d3ecxa_ 174848

d3e5oa_ 174673

d2zsta_ 171480

d2zsqa_ 171477

d1jw8a_ 67376

d3ed9a_ 174862

d2zsxa_ 171493

d2zsya_ 171494

d2z6sa_ 154182

d1h1xa_ 90542

d1dxca_ 15026

d1dxda_ 15025

d2ekua_ 146884

d1u7sa_ 119622

d1j3fa_ 103835

d4it8a_ 196797

d2jhoa1 138322

d3sdna_ 185360

d2evka_ 132433

d1v9qa_ 119893

d2mbwa_ 15030

d1bvda_ 15027

d1do1a_ 15049

d2g14a_ 134511

d1co9a_ 15028

d2g11a_ 134509

d1cioa_ 15029

d2g12a_ 134510

d1wvpa_ 121346

d1bvca_ 15031

d1mz0a_ 85238

d1l2ka_ 73507

d2w6ya_ 169083

d1myza_ 91495

d2g0va_ 134502

d1mbca_ 15032

d2g0xa_ 134505

d3m3aa_ 180784

d2g0za_ 134507

d3m38a_ 180782

d1do3a_ 15077

d1n9xa_ 85477

d1absa_ 15034

d1mbda_ 15033

d3ogba_ 183010

d1vxha_ 15036

d1n9ia_ 85467

d1yoia_ 15035

d1yoha_ 15037

d1cika_ 15039

d1vxfa_ 15038

d2g10a_ 134508

d3nmla_ 182407

d104ma_ 15041

d1vxda_ 15042

d1luea_ 91126

d1vxca_ 15043

d1yoga_ 15040

d1hjta_ 15046

d2spma_ 15045

d2myea_ 15044

d1ufja_ 107815

d1mlsa_ 15058

d1mlla_ 15057

d2spla_ 15047

d1n9ha_ 85466

d1do4a_ 15115

d1f65a_ 15051

d110ma_ 15050

d2spoa_ 15053

d1myma_ 15054

d1ltwa_ 15060

d2oh8a_ 166697

d2blia_ 128731

d1vxga_ 15048

d1mtja_ 15052

d1ofka_ 15061

d2mgea_ 15056

d1tesa_ 15059

d1ajga_ 15067

d1ajha_ 15068

d1fcsa_ 15055

d1ch2a_ 15064

d109ma_ 15063

d1ofja_ 15065

d3a2ga_ 171658

d2ohaa_ 166699

d1vxea_ 15066

d1ch9a_ 15062

d1dtia_ 15074

d2oh9a_ 166698

d1f63a_ 15072

d2eb8a_ 146762

d102ma_ 15073

d2spna_ 15075

d1mcya_ 15076

d2ohba_ 166700

d1jp9a_ 67010

d1swma_ 15070

d2mgga_ 15069

d2blha_ 128730

d1n9fa_ 85465

d2myda_ 15071

d2bw9m_ 129326

d2mgfa_ 15080

d3h57a_ 177193

d1mloa_ 15085

d1co8a_ 15079

d3asea_ 172323

d1ch7a_ 15083

d1mlma_ 15087

d2mgda_ 15078

d1jdoa_ 15084

d2g0sa_ 134498

d1jpba_ 67013

d1mtka_ 15086

d3h58a_ 177194

d2myba_ 15081

d2evpa_ 132439

d2mgca_ 15090

d2myca_ 15082

d1do7a_ 15130

d1mlua_ 15093

d1o16a_ 92405

d1obma_ 15091

d1mboa_ 15089

d1j52a_ 90880

d111ma_ 15088

d2mgma_ 15092

d1mgna_ 15097

d1mlka_ 15108

d1moaa_ 15095

d1mlra_ 15096

d3m39a_ 180783

d1ch1a_ 15094

d2g0ra_ 134497

d2cmma_ 15098

d1cq2a_ 15099

d4fwya_ 194937

d1mlna_ 15106

d1cp0a_ 15109

d2mgba_ 15102

d2bljm_ 128732

d1mlha_ 15105

d1mlga_ 15104

d1mlfa_ 15103

d3m3ba_ 180785

d1moda_ 15107

d1ebca_ 15100

d2eb9a_ 146763

d106ma_ 15101

d1ch3a_ 15111

d2ef2a_ 146809

d1mlqa_ 15112

d2myaa_ 15131

d2mgla_ 15110

d2mgka_ 15117

d1mlja_ 15116

d2mgia_ 15118

d2w6wa_ 169081

d1mtia_ 15114

d1moca_ 15113

d2mgja_ 15119

d101ma_ 15125

d4mbna_ 15120

d1cp5a_ 15123

d107ma_ 15122

d1mbia_ 15121

d103ma_ 15124

d105ma_ 15128

d2bwha1 129365

d4fwxa_ 194936

d5mbna_ 15126

d1ch5a_ 15127

d2mgha_ 15129

d1jp6a_ 67005

d2mgaa_ 15133

d1spea_ 15132

d1moba_ 15134

d1cpwa_ 15136

d2mb5a_ 15138

d1duoa_ 15137

d112ma_ 15135

d1jp8a_ 67009

d1ufpa_ 107818

d1vxaa_ 15139

d1dtma_ 15140

d1irca_ 15141

d108ma_ 15142

d4fwza_ 194938

d1duka_ 15143

d1iopa_ 15144

d1vxba_ 15145

d1mbna_ 15146

d2d6ca_ 131301

d2d6cb_ 131302

d1myfa_ 15147

d1f6ha_ 15148

d1myta_ 15207

d1oj6a_ 93088

d1oj6b_ 93089

d1oj6c_ 93090

d1oj6d_ 93091

d1q1fa_ 104476

d3gk9a_ 176718

d3gkta_ 176725

d1w92a_ 114393

d3glna_ 176734

d2gnva_ 164776

d2gnvb_ 164777

d1d8ua_ 15233

d1d8ub_ 15234

d2gnwa_ 164778

d2gnwb_ 164779

d1h97a_ 60812

d1h97b_ 60813

d1kfra_ 72424

d3ixfa_ 178667

d3ixfb_ 178668

d4hrtb_ 196783

d3uhha_ 186280

d3uhhb_ 186281

d3ugza_ 186260

d3ugzb_ 186261

d3uhca_ 186272

d3uhcb_ 186273

d3uhda_ 186274

d3uhdb_ 186275

d3uhba_ 186270

d3uhbb_ 186271

d3uh7a_ 186268

d3uh7b_ 186269

d3uhxa_ 186306

d3uhxb_ 186307

d3uhva_ 186302

d3uhvb_ 186303

d2auqa_ 162904

d2auqb_ 162905

d3uhqa_ 186292

d3uhqb_ 186293

d3uhga_ 186278

d3uhgb_ 186279

d3ui0a_ 186312

d3ui0b_ 186313

d3uhra_ 186294

d3uhrb_ 186295

d2aupa_ 162902

d2aupb_ 162903

d3uh3a_ 186262

d3uh3b_ 186263

d3uhza_ 186310

d3uhzb_ 186311

d3uhna_ 186290

d3uhnb_ 186291

d3uhta_ 186298

d3uhtb_ 186299

d3uhwa_ 186304

d3uhwb_ 186305

d3uhka_ 186286

d3uhkb_ 186287

d3uhkc_ 186288

d3uhkd_ 186289

d3uhsa_ 186296

d3uhsb_ 186297

d3uhua_ 186300

d3uhub_ 186301

d3ugya_ 186258

d3ugyb_ 186259

d3uh5a_ 186264

d3uh5b_ 186265

d3uhya_ 186308

d3uhyb_ 186309

d2grfa_ 164811

d2grfb_ 164812

d3uh6a_ 186266

d3uh6b_ 186267

d2aura_ 162906

d2aurb_ 162907

d3uhea_ 186276

d3uheb_ 186277

d3uhia_ 186282

d3uhib_ 186283

d3uhic_ 186284

d3uhid_ 186285

d2oifa_ 166703

d2oifb_ 166704

d2oifc_ 166705

d2oifd_ 166706

d2oife_ 166707

d2oiff_ 166708

d2oifg_ 166709

d2oifh_ 166710

d3bcqa_ 172546

d3bcqb_ 172547

d3bcqc_ 172548

d3bcqd_ 172549

d3cy5a_ 173536

d3cy5b_ 173537

d3cy5c_ 173538

d3cy5d_ 173539

d3gdja_ 176533

d3gdjb_ 176534

d3gdjc_ 191712

d3gdjd_ 176535

d3d4xa_ 173683

d3d4xb_ 191706

d3d4xc_ 192726

d3d4xd_ 173684

d3gqpa_ 176916

d3gqpb_ 191720

d3gqpc_ 176917

d3gqpd_ 191721

d3gqra_ 176918

d3gqrb_ 191722

d3gqrc_ 176919

d3gqrd_ 191723

d3gqre_ 176920

d3gqrf_ 191724

d3gqrg_ 176921

d3gqrh_ 191725

d3gysa_ 177098

d3gysb_ 191732

d3gysc_ 177099

d3gysd_ 191733

d3gyse_ 177100

d3gysf_ 191734

d3gysg_ 177101

d3gysh_ 191735

d3mjpa_ 181306

d3mjpb_ 181307

d3mjpc_ 192772

d3mjpd_ 181308

d2r80a_ 168002

d2r80b_ 168003

d2r80c_ 168004

d2r80d_ 168005

d3dhra_ 173959

d3dhrb_ 173960

d3dhrc_ 173961

d3dhrd_ 173962

d3dhre_ 173963

d3dhrf_ 173964

d3dhrg_ 173965

d3dhrh_ 173966

d3eoka_ 175119

d3eokb_ 175120

d2b7hd_ 144966

d4esaa_ 194363

d4esac_ 194364

d4esad_ 193402

d3zhwa_ 196658

d3d1aa_ 173613

d3d1ab_ 173614

d3d1ac_ 173615

d3d1ad_ 173616

d2ri4a_ 168129

d2ri4b_ 168130

d2ri4c_ 168131

d2ri4d_ 168132

d2ri4i_ 168133

d2ri4j_ 168134

d2ri4k_ 168135

d2ri4l_ 168136

d3hyua_ 177949

d3hyub_ 177950

d3a0ga_ 171630

d3a0gb_ 171631

d2w72c_ 169085

d2yrsb_ 170873

d2yrsd_ 170875

d2yrsk_ 170877

d2yrso_ 170879

d4b3wa_ 194093

d4b3wb_ 194092

d3mkba_ 181340

d3mkbb_ 181341

d3mkbc_ 192773

d3mkbd_ 181342

d3lqda_ 180505

d3lqdb_ 180506

d3lqdc_ 191770

d3lqdd_ 180507

d2r50a_ 167985

d2r50b_ 167986

d2r50c_ 167987

d2r50d_ 167988

d3vrga_ 194345

d3vrgb_ 194343

d3vrfa_ 194344

d3vrfb_ 194341

d3vrea_ 194342

d3vreb_ 194338

d3vrec_ 194340

d3vred_ 194339

d2vrya_ 153525

d3hrwa_ 177796

d3hrwb_ 177797

d3hrwc_ 191739

d3hrwd_ 177798

d3hf4a_ 177460

d3hf4b_ 177461

d3hf4e_ 177462

d3hf4f_ 177463

d3dhta_ 173967

d3dhtb_ 173968

d3qqrb_ 193709

d4h2lb_ 196782

d3at5a_ 172328

d3at5b_ 172329

d3at6a_ 172330

d3at6b_ 172331

d3fh9a_ 175781

d3fh9b_ 175782

d2raoa_ 168031

d2raob_ 168032

d2raoc_ 168033

d2raod_ 168034

d3boma_ 172731

d3bomb_ 172732

d3bomc_ 172733

d3bomd_ 172734

d2r1ha_ 167915

d2r1hb_ 167916

d2r1hc_ 167917

d2r1hd_ 167918

d2qu0a_ 167802

d2qu0b_ 167803

d2qu0c_ 167804

d2qu0d_ 167805

d2e2ya_ 163807

d2w6xa_ 169082

d3mn0a_ 181439

d3k9za_ 179218

d3fs4a_ 176009

d3fs4b_ 176010

d3fs4c_ 176011

d3fs4d_ 176012

d2nrla_ 166333

d3qm7a_ 196059

d2nrma_ 166334

d2nx0a_ 166392

d3qqqa_ 196354

d3qqqb_ 196353

d2qmba_ 167720

d2qmbb_ 192698

d2qmbc_ 167721

d2qmbd_ 167722

d3k8ba_ 179180

d3k8bb_ 192734

d3k8bc_ 192732

d3k8bd_ 192733

d3bj1a_ 161614

d3bj1c_ 161615

d3bj2a_ 161616

d3bj2c_ 161617

d3bj3a_ 161618

d3bj3c_ 161619

d1b33a_ 15649

d1b33c_ 15651

d1b33e_ 15653

d1b33h_ 15655

d1b33j_ 15657

d1b33l_ 15659

d1kn1a_ 77455

d1alla_ 15647

d1b33b_ 15650

d1b33d_ 15652

d1b33f_ 15654

d1b33i_ 15656

d1b33k_ 15658

d1b33m_ 15660

d1kn1b_ 77456

d1allb_ 15648

d1phna_ 15641

d1f99a_ 59722

d1f99k_ 59724

d1f99m_ 59726

d1ha7a_ 70935

d1ha7c_ 70937

d1ha7e_ 70939

d1ha7g_ 70941

d1ha7i_ 70943

d1ha7k_ 70945

d1ha7m_ 70947

d1ha7o_ 70949

d1ha7q_ 70951

d1ha7s_ 70953

d1ha7u_ 70955

d1ha7w_ 70957

d1gh0a_ 60491

d1gh0c_ 60493

d1gh0e_ 60495

d1gh0g_ 60497

d1gh0i_ 60499

d1gh0k_ 60501

d1gh0m_ 60503

d1gh0o_ 60505

d1gh0q_ 60507

d1gh0s_ 60509

d1gh0u_ 60511

d1gh0w_ 60513

d2uuma1 152185

d2uumc1 152186

d2uume1 152187

d2uumg1 152188

d2uumi1 152189

d2uumk1 152190

d2uumm1 152191

d2uumo1 152192

d2uumq1 152193

d2uums1 152194

d2uumu1 152195

d2uumw1 152196

d1jboa_ 84150

d3o18a_ 182724

d3o2ca_ 182752

d1ktpa_ 72990

d1i7ya_ 61917

d4gy3a_ 193919

d1on7a_ 87121

d3l0fa_ 179840

d1phnb_ 15642

d1f99b_ 59723

d1f99l_ 59725

d1f99n_ 59727

d1ha7b_ 70936

d1ha7d_ 70938

d1ha7f_ 70940

d1ha7h_ 70942

d1ha7j_ 70944

d1ha7l_ 70946

d1ha7n_ 70948

d1ha7p_ 70950

d1ha7r_ 70952

d1ha7t_ 70954

d1ha7v_ 70956

d1ha7x_ 70958

d1gh0b_ 60492

d1gh0d_ 60494

d1gh0f_ 60496

d1gh0h_ 60498

d1gh0j_ 60500

d1gh0l_ 60502

d1gh0n_ 60504

d1gh0p_ 60506

d1gh0r_ 60508

d1gh0t_ 60510

d1gh0v_ 60512

d1gh0x_ 60514

d1jbob_ 84151

d3o18b_ 182725

d3o2cb_ 182753

d1ktpb_ 72991

d1i7yb_ 61918

d1on7b_ 87122

d3l0fb_ 179841

d4gy3b_ 193918

d1b8da_ 15665

d1b8dk_ 15667

d1liaa_ 15661

d1liak_ 15663

d1eyxa_ 15669

d1eyxk_ 15671

d1xg0c_ 115275

d1xg0d_ 115276

d1xf6c_ 115242

d1xf6d_ 115243

d1qgwc_ 15673

d1qgwd_ 15674

d1b8db_ 15666

d1b8dl_ 15668

d1liab_ 15662

d1lial_ 15664

d1eyxb_ 15670

d1eyxl_ 15672

d2vjha_ 168645

d2vjhb_ 168646

d2vjhc_ 168647

d2vjhd_ 168648

d2vmla_ 168702

d2vmlb_ 168703

d2vmlc_ 168704

d2vmld_ 168705

d2vmle_ 168706

d2vmlf_ 168707

d2vmlg_ 168708

d2vmlh_ 168709

d2vmli_ 168710

d2vmlj_ 168711

d2vmlk_ 168712

d2vmll_ 168713

d2vjta_ 168661

d2vjtb_ 168662

d2vjra_ 168659

d2vjrb_ 168660

d2j96a_ 165947

d2j96b_ 165948

d2c7la_ 163287

d2c7lb_ 163288

d3v58b_ 194585

d3v58c_ 194586

d3kvsa_ 179738

d3kvsb_ 179739

d3brpa_ 172801

d3brpb_ 172802

d2bv8a_ 163172

d2bv8b_ 163173

d2bv8c_ 163174

d2bv8d_ 163175

d2bv8e_ 163176

d2bv8f_ 163177

d2bv8k_ 163178

d2bv8l_ 163179

d2bv8m_ 163180

d2bv8n_ 163181

d2bv8o_ 163182

d2bv8p_ 163183

d4h0me_ 193232

d4h0mp_ 193915

d4h0mr_ 193916

d4f0ub_ 193925

d4f0uc_ 193920

d4f0uf_ 193922

d4f0ta_ 193924

d4f0tb_ 193923

d3dbja_ 173798

d3dbjb_ 173799

d3dbjc_ 173800

d3dbjd_ 173801

d3dbje_ 173802

d3dbjf_ 173803

d3dbjg_ 173804

d3dbjh_ 173805

d2xkia_ 170181

d4avda_ 192678

d1kr7a_ 72890

d4f6ja_ 195268

d2vyya_ 168950

d4f6ba_ 195267

d4f6ga_ 195266

d1v07a_ 108201

d4f6da_ 195271

d2vyza_ 168951

d4avea_ 192677

d2xkga_ 170179

d4f69a_ 195270

d4f68a_ 195269

d2xkha_ 170180

d2xyka_ 170484

d2xykb_ 170485

d2wy4a_ 169686

d2ig3a_ 165538

d2ig3b_ 165539

d3pt8a_ 183967

d3pt8b_ 196095

d2olpa_ 166763

d2olpb_ 166764

d3pi2a_ 183752

d3pi2b_ 183753

d3pi3a_ 183754

d3pi3b_ 183755

d3pi1a_ 183750

d3pi1b_ 183751

d3pt7a_ 183966

d3pt7b_ 196096

d2vywa_ 168937

d2bk9a_ 193844

d2g3ha_ 164573

d2c0kb_ 193836

d3ubca_ 195690

d2wtga_ 169615

d2wtha_ 169616

d2wthb_ 169617

d2zs0a_ 171448

d2zs0b_ 171449

d2zs0c_ 171450

d2zs0d_ 171451

d2zs1a_ 171452

d2zs1b_ 171453

d2zs1c_ 171454

d2zs1d_ 171455

d2zfoa_ 171194

d2zfob_ 171195

d2zfoc_ 171196

d2zfod_ 171197

d2d2ma_ 163559

d2d2mb_ 163560

d2d2mc_ 163561

d2d2md_ 163562

d1x46a_ 162036

d1x3ka_ 193197

d1kf6b1 72397

d1kf6n1 72404

d1l0vb1 73415

d1l0vn1 73422

d1kfyb1 72430

d1kfyn1 72437

d2b76b1 128012

d2b76n1 128019

d3cirb1 156682

d3cirn1 156689

d2bs2b1 129033

d2bs2e1 129039

d2bs3b1 129045

d2bs3e1 129050

d1qlbb1 15681

d1qlbe1 15682

d2bs4b1 129055

d2bs4e1 129060

d1e7pb1 59350

d1e7pe1 59356

d1e7ph1 59362

d1e7pk1 59368

d1nekb1 80429

d1nenb1 80436

d2aczb1 126564

d1gtea1 70440

d1gteb1 70445

d1gtec1 70450

d1gted1 70455

d1h7wa1 15683

d1h7wb1 15684

d1h7wc1 15685

d1h7wd1 15686

d1h7xa1 15687

d1h7xb1 15688

d1h7xc1 15689

d1h7xd1 15690

d1gtha1 70483

d1gthb1 70488

d1gthc1 70493

d1gthd1 70498

d1gt8a1 70418

d1gt8b1 70423

d1gt8c1 70428

d1gt8d1 70433

d1grja1 15691

d2etna1 132365

d2etnb1 132367

d2etnc1 132369

d2f23a1 132797

d2f23b1 132799

d2eula1 132392

d2eulb1 132394

d2eulc1 132396

d2euld1 132398

d2zjrv1 154575

d3cf5v1 156562

d2aarw1 146044

d2d3ow1 146453

d2zjqv1 154546

d2zjpv1 154514

d3dllv1 157799

d1xbpw1 145887

d2qamx1 150264

d2qaox1 150317

d2qbex1 150484

d2qbgx1 150538

d2i2ty1 137005

d2i2vy1 137018

d2qozx1 151140

d2qp1x1 151193

d3df4x1 157707

d3df2x1 157653

d2qbax1 150377

d2qbcx1 150430

d2qoxx1 151087

d2qovx1 151034

d1vs8x1 120515

d2qbix1 150592

d2qbkx1 150646

d2awbx1 127428

d2aw4x1 127406

d1vs6x1 120501

d2vhmx1 153087

d2vhnx1 153119

d2z4lx1 154102

d2z4nx1 154156

d2rdox1 151964

d2gycw1 135855

d2j28x1 137970

d2gyaw1 135843

d2vrhd1 153511

d3bbxx1 155121

d1vq8v1 120209

d1vqov1 120383

d1vqpv1 120412

d1yhqv1 123204

d1s72v_ 105341

d1vqmv1 120325

d1jj2u_ 63106

d1vqlv1 120296

d1vqkv1 120267

d1vqnv1 120354

d1yijv1 123319

d1yi2v1 123251

d1vq7v1 120180

d1vq9v1 120238

d1vq5v1 120122

d1vq4v1 120093

d1vq6v1 120151

d1yitv1 123362

d1yjwv1 123486

d1m90w_ 78861

d2otlv1 139367

d2otjv1 139338

d1njiw_ 85814

d1yjnv1 123454

d1kqsu_ 68836

d1yj9v1 123414

d1qvgu_ 96413

d1kc8w_ 84378

d1n8rw_ 85450

d1q82w_ 96150

d1qvfu_ 96383

d1q81w_ 96120

d1k73w_ 84339

d1k9mw_ 72234

d1kd1w_ 72345

d1k8aw_ 72167

d1m1kw_ 74405

d1q86w_ 96188

d1q7yw_ 96086

d1ffks_ 15692

d1r73a_ 104827

d2j0321 137886

d2j0121 137860

d2hgq11 145329

d2hgj11 145297

d2hgu11 145361

d1vsaw1 120519

d1yl3w1 123594

d2b6621 127954

d2b9p21 128183

d2b9n21 128146

d1nz6a_ 86441

d1nz6b_ 86442

d1n4ca_ 91617

d1wjza_ 114717

d1xbla_ 15697

d1bqza_ 15699

d1bq0a_ 15698

d1fpoa1 15693

d1fpob1 15694

d1fpoc1 15695

d1hdja_ 15696

d1iura_ 90704

d1fafa_ 15700

d1gh6a_ 65191

d2o37a_ 166515

d2ocha_ 166642

d3ag7a_ 196422

d1fxkc_ 15702

d1fxka_ 15703

d1fxkb_ 15704

d1cxzb_ 15705

d1urfa_ 99827

d2rmkb1 152167

d1seta1 15708

d1setb1 15709

d1srya1 15706

d1sryb1 15707

d1sesa1 15710

d1sesb1 15711

d1sera1 15712

d1serb1 15713

d2iy5a1 137792

d1eiya1 15714

d1ivsa1 76855

d1ivsb1 76859

d1gaxa4 75842

d1gaxb4 75844

d1iywa1 83807

d1iywb1 83811

d1lrza1 78167

d1k4ta1 77263

d1rrja1 105078

d1a36a1 15715

d1sc7a1 118944

d1seua1 118952

d1tl8a1 119299

d1t8ia1 119172

d1lpqa1 74174

d1r49a1 96983

d1qoja_ 15716

d1qojb_ 15717

d1e52a_ 59256

d1e52b_ 59257

d1e79h1 15721

d1aqta1 15718

d1bsna1 15719

d1bsha1 15720

d1fs0e1 59997

d1uera1 107790

d1uerb1 107792

d1uerc1 107794

d1uerd1 107796

d1uesa1 107798

d1uesb1 107800

d1uesc1 107802

d1uesd1 107804

d1qnna1 15792

d1qnnb1 15793

d1qnnc1 15794

d1qnnd1 15795

d1bsma1 15780

d1bsmb1 15781

d1avma1 15782

d1avmb1 15783

d1bs3a1 15784

d1bs3b1 15785

d1ar5a1 15786

d1ar5b1 15787

d1ar4a1 15788

d1ar4b1 15789

d1bt8a1 15790

d1bt8b1 15791

d1coja1 15737

d1unfx1 113314

d2nyba1 138804

d2nybb1 138806

d2nybc1 138808

d2nybd1 138810

d2bkba1 128667

d2bkbb1 128669

d2bkbc1 128671

d2bkbd1 128673

d1isca1 15733

d1iscb1 15734

d1isaa1 15731

d1isab1 15732

d1isba1 15735

d1isbb1 15736

d1za5a1 124802

d1za5b1 124804

d1ma1a1 74609

d1ma1b1 74611

d1ma1c1 74613

d1ma1d1 74615

d1ma1e1 74617

d1ma1f1 74619

d1idsa1 15722

d1idsb1 15723

d1idsc1 15724

d1idsd1 15725

d1gn4a1 65379

d1gn4b1 65381

d1gn4c1 65383

d1gn4d1 65385

d1gn6a1 65387

d1gn6b1 65389

d1gn6c1 65391

d1gn6d1 65393

d1gn3a1 65375

d1gn3b1 65377

d1gn2a1 65359

d1gn2b1 65361

d1gn2c1 65363

d1gn2d1 65365

d1gn2e1 65367

d1gn2f1 65369

d1gn2g1 65371

d1gn2h1 65373

d1dt0a1 15726

d1dt0b1 15727

d1dt0c1 15728

d3sdpa1 15729

d3sdpb1 15730

d1p7ga1 94223

d1p7gb1 94225

d1p7gc1 94227

d1p7gd1 94229

d1p7ge1 94231

d1p7gf1 94233

d1p7gg1 94235

d1p7gh1 94237

d1p7gi1 94239

d1p7gj1 94241

d1p7gk1 94243

d1p7gl1 94245

d1p7gm1 94247

d1p7gn1 94249

d1p7go1 94251

d1p7gp1 94253

d1p7gq1 94255

d1p7gr1 94257

d1p7gs1 94259

d1p7gt1 94261

d1p7gu1 94263

d1p7gv1 94265

d1p7gw1 94267

d1p7gx1 94269

d1b06a1 15740

d1b06b1 15741

d1b06c1 15742

d1b06d1 15743

d1b06e1 15744

d1b06f1 15745

d1wb8a1 114482

d1wb8b1 114484

d1wb7a1 114478

d1wb7b1 114480

d1my6a1 85232

d1my6b1 85234

d1gv3a1 70585

d1gv3b1 70587

d1kkca1 68660

d1kkcb1 68662

d1kkcx1 68664

d1kkcy1 68666

d1jr9a1 71822

d1y67a1 116496

d1y67b1 116498

d1y67c1 116500

d1y67d1 116502

d1ix9a1 76899

d1ix9b1 76901

d1ixba1 76903

d1ixbb1 76905

d1i0ha1 15760

d1i0hb1 15761

d1zlza1 125272

d1zlzb1 125274

d1d5na1 15762

d1d5nb1 15763

d1d5nc1 15764

d1d5nd1 15765

d1en4a1 59457

d1en4b1 59459

d1en4c1 59461

d1en4d1 59463

d1en6a1 59473

d1en6b1 59475

d1en6c1 59477

d1en6d1 59479

d1vewa1 15766

d1vewb1 15767

d1vewc1 15768

d1vewd1 15769

d1i08a1 15770

d1i08b1 15771

d1i08c1 15772

d1i08d1 15773

d1en5a1 59465

d1en5b1 59467

d1en5c1 59469

d1en5d1 59471

d1mmma1 15774

d1mmmb1 15775

d2p4ka1 139487

d2p4kb1 139489

d2p4kc1 139491

d2p4kd1 139493

d1xila1 122014

d1xilb1 122016

d1pl4a1 94854

d1pl4b1 94856

d1pl4c1 94858

d1pl4d1 94860

d1pm9a1 94897

d1pm9b1 94899

d1xdca1 121864

d1xdcb1 121866

d1ztea1 125641

d1zteb1 125643

d1ztec1 125645

d1zted1 125647

d1ap6a1 15746

d1ap6b1 15747

d1n0na1 79750

d1n0nb1 79752

d1zspa1 125612

d1zspb1 125614

d3c3ta1 155919

d3c3tb1 155921

d1szxa1 99049

d1szxb1 99051

d2qkaa1 150839

d2qkac1 150841

d1luva1 74263

d1luvb1 74265

d1n0ja1 79740

d1n0jb1 79742

d1zuqa1 125683

d1zuqb1 125685

d1ap5a1 15750

d1ap5b1 15751

d1ja8a1 62813

d1ja8b1 62815

d1em1a1 15752

d1em1b1 15753

d2qkca1 150843

d2qkcc1 150845

d2gdsa1 135024

d2gdsb1 135026

d2gdsc1 135028

d2gdsd1 135030

d2adpa1 126591

d1qnma1 15754

d1qnmb1 15755

d2adqb1 126593

d3c3sa1 155915

d3c3sb1 155917

d1vara1 15756

d1varb1 15757

d1luwa1 74267

d1luwb1 74269

d1msda1 15758

d1msdb1 15759

d1mnga1 15776

d1mngb1 15777

d3mdsa1 15778

d3mdsb1 15779

d1pv0a_ 95141

d3fyra_ 176151

d3fyrb_ 176152

d3fyrc_ 176153

d3d36c_ 173656

d1rfya_ 111798

d1rfyb_ 111799

d1us6a_ 107999

d1us6b_ 108000

d1upga_ 107992

d1upgb_ 107993

d2hjda_ 165126

d2hjdb_ 165127

d2hjdc_ 165128

d2hjdd_ 165129

d1tjla1 107027

d1tjlb1 107029

d1tjlc1 107031

d1tjld1 107033

d1tjle1 107035

d1tjlf1 107037

d1tjlg1 107039

d1tjlh1 107041

d1tjli1 107043

d1tjlj1 107045

d1x4ta1 121698

d2a26a1 126027

d2a26b_ 126028

d2a26c_ 126029

d1ysma1 123979

d2f6ma_ 133051

d2f6mc_ 133053

d2f66a1 133027

d2f66d_ 133030

d2caza1 130169

d2cazd1 130172

d2f6mb_ 133052

d2f6md_ 133054

d2f66b1 133028

d2f66e_ 133031

d2cazb1 130170

d2caze1 130173

d2p22b_ 166951

d2f66c1 133029

d2f66f_ 133032

d2cazc1 130171

d1z0pa1 124325

d1z0jb1 124320

d1yzma1 124283

d1z0kb1 124322

d1z0kd_ 124324

d2g0ua1 134501

d2ca5a1 130149

d2ca5b_ 130150

d2x9cb_ 193727

d2hepa1 147271

d3bhpa_ 172639

d3bhpb_ 172640

d3bhpc_ 172641

d1gu2a_ 76348

d1gu2b_ 76349

d1oaea_ 92704

d1oaeb_ 92705

d1e8ea_ 64795

d1cota_ 15899

d155ca_ 15900

d1vyda_ 108900

d1vydb_ 108901

d1c2ra_ 15887

d1c2rb_ 15888

d1c2na_ 15889

d1cxca_ 15890

d2cxba_ 15891

d2cxbb_ 15892

d1cxaa_ 15893

d1l9bc_ 73711

d1l9jc_ 73722

d1l9jd_ 73723

d1hroa_ 15897

d1hrob_ 15898

d1i8oa_ 61979

d1hh7a_ 15896

d1fj0a_ 65011

d1fj0b_ 65012

d1fj0c_ 65013

d1fj0d_ 65014

d1i8pa_ 61980

d1i8pb_ 61981

d1i8pc_ 61982

d1i8pd_ 61983

d1co6a_ 15894

d1io3a_ 62613

d1crya_ 15895

d1jdla_ 66557

d3c2ca_ 15885

d2c2ca_ 15886

d1cc5a_ 15901

d1mz4a_ 91496

d2axtv1 127499

d1gksa_ 15909

d2gc7d_ 134957

d2gc7h_ 134961

d2gc7l_ 134965

d2gc7p_ 134969

d2gc4d_ 134937

d2gc4h_ 134941

d2gc4l_ 134945

d2gc4p_ 134949

d1mg2d_ 79062

d1mg2h_ 79066

d1mg2l_ 79070

d1mg2p_ 79074

d2mtac_ 15908

d1mg3d_ 79078

d1mg3h_ 79082

d1mg3l_ 79086

d1mg3p_ 79090

d451ca_ 15904

d351ca_ 15905

d1dvva_ 15906

d2paca_ 15907

d1cora_ 15903

d1fi3a_ 59846

d1ccha_ 15902

d1ynra_ 123750

d1ynrb_ 123751

d1ynrc_ 123752

d1ynrd_ 123753

d3vyma_ 192538

d2ai5a1 126817

d1ayga_ 15831

d1a56a_ 15832

d1a8ca_ 15833

d1ql3a_ 15822

d1ql3b_ 15823

d1ql3c_ 15824

d1ql3d_ 15825

d1ql4a_ 15826

d1ql4b_ 15827

d1ql4c_ 15828

d1ql4d_ 15829

d1i6da_ 66038

d1i6ea_ 66039

d1c7ma_ 15830

d1cnoa_ 15814

d1cnob_ 15815

d1cnoc_ 15816

d1cnod_ 15817

d1cnoe_ 15818

d1cnof_ 15819

d1cnog_ 15820

d1cnoh_ 15821

d1c52a_ 15810

d1qyza_ 104662

d1r0qa_ 104755

d1dt1a_ 15811

d3vnwa_ 194071

d1foca_ 15812

d1focb_ 15813

d2fwla1 134247

d1f1fa_ 59571

d1kiba_ 72502

d1kibb_ 72503

d1kibc_ 72504

d1kibd_ 72505

d1kibe_ 72506

d1kibf_ 72507

d1kibg_ 72508

d1kibh_ 72509

d1c75a_ 15796

d1b7va_ 15797

d1k3ha_ 68112

d1k3ga_ 68111

d1n9ca_ 80340

d2dvha_ 15803

d1c53a_ 15801

d1dvha_ 15802

d1cyia_ 15804

d1cyja_ 15805

d1ls9a_ 78177

d1c6ra_ 15807

d1c6oa_ 15808

d1c6ob_ 15809

d1ctja_ 15798

d1ceda_ 15799

d1a2sa_ 15800

d1gdva_ 60458

d1c6sa_ 15806

d1qn2a_ 15882

d1qn2b_ 15883

d1qn2c_ 15884

d1ycca_ 15834

d3tyia_ 192162

d3tyib_ 192163

d1ytca_ 15835

d2bcnb_ 128300

d1ciha_ 15836

d1s6vb_ 98610

d1s6vd_ 98612

d1csua_ 15837

d1ciea_ 15838

d1ciga_ 15840

d1cswa_ 15839

d1csxa_ 15841

d1crja_ 15842

d1yeba_ 15843

d1raqa_ 15844

d1csva_ 15850

d1crha_ 15849

d1cria_ 15847

d3cx5w_ 161630

d1yeaa_ 15845

d1chia_ 15848

d1cifa_ 15846

d1chja_ 15851

d1chha_ 15852

d1crga_ 15854

d1irwa_ 15855

d1ctza_ 15853

d2ycca_ 15856

d1irva_ 15857

d2pccb_ 15860

d2pccd_ 15861

d1rapa_ 15858

d1ctya_ 15859

d3cxhw_ 161631

d2b11b1 144945

d2b11d_ 144946

d1u74b_ 107705

d1u74d_ 107707

d1kyow_ 73279

d2b0zb1 144942

d2b10b1 144943

d2b10d_ 144944

d2b12b1 144947

d2gb8b1 134910

d2hv4a1 136782

d1fhba_ 15862

d2orla1 139273

d1yica_ 15863

d1nmia_ 80659

d1yfca_ 15864

d1lmsa_ 84633

d5cytr_ 15878

d1lfma_ 73883

d1lfmb_ 73884

d1i54a_ 61768

d1i54b_ 61769

d1i55a_ 61770

d1i55b_ 61771

d3cyti_ 15880

d3cyto_ 15879

d1cyca_ 15881

d1cycb_ 118450

d3o1ya_ 182738

d3o1yb_ 182739

d3o1yc_ 182740

d1wejf_ 15865

d1hrca_ 15866

d3o20a_ 182742

d3o20b_ 182743

d3o20c_ 182744

d3nbta_ 182131

d3nbtb_ 182132

d3nbtc_ 182133

d3nbtd_ 182134

d3nbte_ 182135

d3nbtf_ 182136

d3nbsa_ 182127

d3nbsb_ 182128

d3nbsc_ 182129

d3nbsd_ 182130

d1crca_ 15867

d1crcb_ 15868

d2pcbb_ 15869

d1u75b_ 107709

d1i5ta_ 61823

d1m60a_ 74519

d1lc1a_ 84578

d1giwa_ 15874

d1akka_ 15873

d1fi7a_ 15872

d2giwa_ 15875

d1lc2a_ 84579

d1fi9a_ 15870

d2frca_ 15876

d1ocda_ 15871

d3nwva_ 182593

d3nwvb_ 182594

d3nwvc_ 182595

d3nwvd_ 182596

d1j3sa_ 103838

d1ccra_ 15877

d1kx7a_ 68880

d1kx2a_ 68878

d1h32b_ 76620

d1h33b_ 76623

d2oz1b_ 149087

d2oz1d_ 149090

d2oz1f_ 149093

d2oz1h_ 149096

d1h31b_ 76608

d1h31d_ 76611

d1h31f_ 76614

d1h31h_ 76617

d1diqc_ 15923

d1diqd_ 15924

d1diic_ 15925

d1diid_ 15926

d1f1ca_ 59569

d1f1cb_ 59570

d1e29a_ 59161

d1dw0a_ 15911

d1dw0b_ 15912

d1dw0c_ 15913

d1dw1a_ 15914

d1dw1b_ 15915

d1dw1c_ 15916

d1dw3a_ 15917

d1dw3b_ 15918

d1dw3c_ 15919

d1dw2a_ 15920

d1dw2b_ 15921

d1dw2c_ 15922

d2b4za_ 162992

d2yk3a_ 170831

d2yk3b_ 170832

d2yk3c_ 170833

d2zboa_ 171136

d2zboc_ 171137

d2zboe_ 171138

d2zbog_ 171139

d2zboi_ 171140

d2zbok_ 171141

d4gedb_ 194460

d2aiua_ 162821

d2bh4x_ 163082

d2bgvx_ 163081

d2bh5x_ 163083

d3dmia_ 174056

d3ph2b_ 183731

d2v08a_ 168271

d2v08b_ 168272

d2exva_ 164210

d2exvc_ 164211

d1wvec_ 121335

d1wved_ 121336

d3bz2v_ 172963

d3bz1v_ 172952

d3arcv_ 172320

d4il6v_ 196655

d1qksa1 15951

d1qksb1 15952

d1e2ra1 15953

d1e2rb1 15954

d1gq1a1 76264

d1gq1b1 76266

d1hj5a1 15927

d1hj5b1 15928

d1dy7b1 15929

d1hj3a1 15930

d1hj3b1 15931

d1hj4a1 15932

d1hj4b1 15933

d1aoqa1 15934

d1aoqb1 15935

d1aomb1 15936

d1aofa1 15937

d1aofb1 15938

d1h9xa1 60855

d1h9xb1 60857

d1hcma1 60958

d1hcmb1 60960

d1h9ya1 60859

d1h9yb1 60861

d1nira1 15939

d1nirb1 15940

d1gjqa1 70193

d1gjqb1 70195

d1hzua1 61460

d1nnoa1 15941

d1nnob1 15942

d1n15a1 15943

d1n15b1 15944

d1n90a1 15945

d1n90b1 15946

d1n50a1 15949

d1n50b1 15950

d1bl9a1 15947

d1bl9b1 15948

d1hzva1 61462

d3cx5d1 157082

d3cx5o1 157098

d1kb9d1 77317

d2ibzd1 137221

d1ezvd1 59546

d3cxhd1 157115

d3cxho1 157131

d1p84d1 87856

d1kyod1 73254

d1kyoo1 73269

d1bccd2 15959

d2bccd2 15960

d3bccd2 15961

d2a06d1 125923

d2a06q1 125932

d1ppjd1 104258

d1ppjq1 104273

d1pp9d1 104228

d1pp9q1 104243

d2fyud1 134398

d1ntmd1 92127

d1l0ld1 84488

d1ntzd1 92155

d1sqxd1 119041

d1ntkd1 92111

d1l0nd1 84504

d1sqbd1 105896

d1sqvd1 119026

d1sqpd1 119001

d1sqqd1 119013

d1nu1d1 92173

d1be3d2 15955

d1bgyd2 15957

d1bgyp2 15958

d1qcrd2 15956

d1m70a1 91200

d1m70a2 91201

d1m70b1 91202

d1m70b2 91203

d1m70c1 91204

d1m70c2 91205

d1m70d1 91206

d1m70d2 91207

d1m6za1 91192

d1m6za2 91193

d1m6zb1 91194

d1m6zb2 91195

d1m6zc1 91196

d1m6zc2 91197

d1m6zd1 91198

d1m6zd2 91199

d1etpa1 15962

d1etpa2 15963

d1etpb1 15964

d1etpb2 15965

d1h1oa1 83454

d1h1oa2 83455

d1h1ob1 83456

d1h1ob2 83457

d1fcdc1 15966

d1fcdc2 15967

d1fcdd1 15968

d1fcdd2 15969

d1iqca1 66266

d1iqca2 66267

d1iqcb1 66268

d1iqcb2 66269

d1iqcc1 66270

d1iqcc2 66271

d1iqcd1 66272

d1iqcd2 66273

d2vhda1 153050

d2vhda2 153051

d2vhdb1 153052

d2vhdb2 153053

d1eb7a1 59404

d1eb7a2 59405

d1nmla1 91976

d1nmla2 91977

d1rz6a1 105135

d1rz6a2 105136

d1rz5a1 105133

d1rz5a2 105134

d1kb0a1 68378

d1kv9a1 73056

d1pbya1 94417

d1pbya2 94418

d1jjua1 66774

d1jjua2 66775

d1jmxa1 66901

d1jmxa2 66902

d1jmza1 66908

d1jmza2 66909

d1h32a1 76618

d1h32a2 76619

d1h33a1 76621

d1h33a2 76622

d2oz1a1 149085

d2oz1a2 149086

d2oz1c1 149088

d2oz1c2 149089

d2oz1e1 149091

d2oz1e2 149092

d2oz1g1 149094

d2oz1g2 149095

d1h31a1 76606

d1h31a2 76607

d1h31c1 76609

d1h31c2 76610

d1h31e1 76612

d1h31e2 76613

d1h31g1 76615

d1h31g2 76616

d2zong_ 171386

d2zxya_ 171602

d3cu4a_ 173463

d3dp5a_ 174142

d2d0sa_ 163536

d2d0wa_ 163543

d2d0wb_ 163544

d2c1db_ 163223

d2c1dd_ 163224

d2c1df_ 163225

d2c1dh_ 163226

d1w2la_ 193857

d4eifa_ 196765

d4eiea_ 196766

d2ce0a_ 163376

d2ce1a_ 163377

d2dgea_ 163627

d2dgeb_ 163628

d2dgec_ 163629

d2dged_ 163630

d2v07a_ 168270

d9anta_ 16004

d9antb_ 16005

d1ahdp_ 16006

d1sana_ 16008

d2hoaa_ 16009

d1homa_ 16007

d1wi3a_ 114660

d2hosa_ 165179

d2hosb_ 165180

d1p7ia_ 94279

d1p7ib_ 94280

d1p7ic_ 94281

d1p7id_ 94282

d2hdda_ 15971

d2hddb_ 15972

d1p7ja_ 94283

d1p7jb_ 94284

d1p7jc_ 94285

d1p7jd_ 94286

d1enha_ 15973

d1du0a_ 15974

d1du0b_ 15975

d2hota_ 165181

d2hotb_ 165182

d3hdda_ 15976

d3hddb_ 15977

d1hddc_ 15978

d1hddd_ 15979

d2jwta1 148230

d2p81a1 139518

d1ztra1 125655

d1jgga_ 62950

d1jggb_ 62951

d1b8ib_ 16011

d1ftza_ 16012

d1ic8a1 76740

d1ic8b1 76742

d1lfba_ 15989

d2lfba_ 15990

d1s7ea1 112044

d1mija_ 79150

d1xpxa_ 122230

d1pufa_ 95131

d1b72a_ 16000

d2craa1 130736

d1x2na1 121646

d2e1oa1 131965

d2cufa1 130809

d2ecca1 132104

d2hi3a1 136522

d1uhsa_ 107853

d1zq3p1 125499

d1x58a1 121705

d1bw5a_ 16003

d2cqxa1 130731

d1x2ma1 121645

d1k61a_ 77270

d1k61b_ 77271

d1k61c_ 77272

d1k61d_ 77273

d1mnmc_ 15983

d1mnmd_ 15984

d1akhb_ 15985

d1le8b_ 73868

d1yrnb_ 15986

d1aplc_ 15987

d1apld_ 15988

d1akha_ 15980

d1le8a_ 73867

d1yrna_ 15981

d1f43a_ 15982

d1mh3a1 79112

d1mh4a1 79114

d1ig7a_ 62365

d1e3oc1 59197

d1gt0c1 76335

d1hf0a1 65820

d1hf0b1 65822

d1octc1 15991

d1cqta1 15992

d1cqtb1 15993

d1o4xa1 92470

d1poga_ 15994

d1hdpa_ 15998

d1ocpa_ 15999

d2cuea1 130808

d1fjla_ 16017

d1fjlb_ 16018

d1fjlc_ 16019

d1pufb_ 95132

d1b72b_ 16001

d1lfup_ 77939

d1du6a_ 16002

d1au7a1 15995

d1au7b1 15996

d2lkxa1 191830

d1ftta_ 15997

d1x41a1 121676

d1b8ia_ 16010

d1nk3p_ 16013

d1nk2p_ 16015

d1vnda_ 16014

d1qrya_ 16016

d1wh7a_ 114635

d1wh5a_ 114633

d2ecba1 146789

d3a02a_ 171620

d3lnqa_ 180423

d3a01b_ 171618

d3a01f_ 171619

d2r5zb_ 151598

d2r5yb_ 151597

d3rkqa_ 195443

d3rkqb_ 195444

d3cmya_ 173335

d3k2aa_ 178978

d3k2ab_ 178979

d1gdta1 16021

d1gdtb1 16022

d1zr4a1 125517

d1zr4b1 125519

d1zr4d1 125521

d1zr4e1 125523

d2gm4a1 135360

d2gm4b1 135362

d1zr2a1 125509

d1zr2b1 125511

d1reta_ 16024

d1resa_ 16023

d1jj6c_ 66756

d1ijwc_ 66171

d1jkoc_ 66809

d1hcra_ 16020

d1jj8c_ 66757

d1jkrc_ 66812

d1jkpc_ 66810

d1jkqc_ 66811

d2ezla_ 16026

d2ezka_ 16027

d2ezia_ 16028

d2ezha_ 16029

d1tc3c_ 16025

d1u78a1 107712

d1u78a2 107713

d1ug2a_ 107823

d1a5ja1 16043

d1a5ja2 16044

d1gvda_ 83338

d1gv5a_ 83337

d1guua_ 83332

d1gv2a1 83335

d1gv2a2 83336

d1h88c1 65721

d1h88c2 65722

d1h88c3 65723

d1mbja_ 16035

d1mbka_ 16031

d1mbha_ 16036

d1mbea_ 16038

d1mbga_ 16033

d1mbfa_ 16037

d1idza_ 16032

d1idya_ 16034

d1msec1 16039

d1msec2 16040

d1msfc1 16041

d1msfc2 16042

d1h89c1 65726

d1h89c2 65727

d1h89c3 65728

d2cqqa1 130728

d2cqra1 130729

d1wgxa_ 114626

d2crga1 130739

d2cu7a1 130805

d1xc5a1 121850

d2cjja1 130540

d1fexa_ 59803

d2iw5b1 137739

d2uxnb1 140024

d1ofcx1 92826

d2ckxa1 146411

d2qhba_ 150786

d2qhbb_ 150787

d2ajea1 146056

d1h8ac1 65731

d1h8ac2 65732

d1w0ta_ 114070

d1w0tb_ 114071

d1iv6a_ 71441

d1itya_ 71427

d1ba5a_ 16045

d1w0ua_ 114072

d1w0ub_ 114073

d1xg1a1 121973

d1vf9a1 120031

d1vfca1 120032

d3sjma_ 194392

d3sjmb_ 194391

d1pdnc1 161344

d1pdnc2 161345

d1k78a1 68255

d1k78a2 68256

d1k78e1 68258

d1k78e2 68259

d1k78i1 68261

d1mdma1 79010

d1mdma2 79011

d6paxa1 64758

d6paxa2 64759

d1igna1 16048

d1igna2 16049

d1ignb1 16050

d1ignb2 16051

d1iufa1 71439

d1iufa2 71440

d1hlva1 65854

d1hlva2 65855

d1bw6a_ 16052

d1bl0a1 16053

d1bl0a2 16054

d1d5ya1 16055

d1d5ya2 16056

d1d5yb1 16057

d1d5yb2 16058

d1d5yc1 16059

d1d5yc2 16060

d1d5yd1 16061

d1d5yd2 16062

d1ui5a1 107856

d1ui5b1 107858

d1ui6a1 107860

d1ui6b1 107862

d1t56a1 106428

d1u9na1 113246

d1u9oa1 113248

d2fx0a1 134269

d3loca1 180465

d3locb1 180467

d3locc1 180469

d3locd1 180471

d1rkta1 97623

d1rktb1 97625

d1vi0a1 100720

d1vi0b1 100722

d1jt6a1 67247

d1jt6b1 67249

d1jt6d1 67251

d1jt6e1 67253

d1rkwa1 104967

d1rkwb1 104969

d1rkwd1 104971

d1rkwe1 104973

d2hq5a1 147329

d2hq5b1 147331

d2hq5d1 147333

d2hq5e1 147335

d3btla1 155582

d3btlb1 155584

d3btld1 155586

d3btle1 155588

d3btia1 155566

d3btib1 155568

d3btid1 155570

d3btie1 155572

d2dtza1 146578

d2dtzb1 146580

d2dtzd1 146582

d2dtze1 146584

d3bt9a1 155550

d3bt9b1 155552

d3bt9d1 155554

d3bt9e1 155556

d1jusa1 67326

d1jusb1 67328

d1jusd1 67330

d1juse1 67332

d1jtxa1 67284

d1jtxb1 67286

d1jtxd1 67288

d1jtxe1 67290

d2g0ea1 147058

d2g0eb1 147060

d2g0ed1 147062

d2g0ee1 147064

d3btja1 155574

d3btjb1 155576

d3btjd1 155578

d3btje1 155580

d1rpwa1 105036

d1rpwb1 105038

d1rpwc1 105040

d1rpwd1 105042

d1qvta1 104602

d1qvtb1 104604

d1qvtd1 104606

d1qvte1 104608

d1qvua1 104610

d1qvub1 104612

d1qvud1 104614

d1qvue1 104616

d3btca1 155558

d3btcb1 155560

d3btcd1 155562

d3btce1 155564

d1jt0a1 71850

d1jt0b1 71852

d1jt0c1 71854

d1jt0d1 71856

d2gbya1 147102

d2gbyb1 147104

d2gbyd1 147106

d2gbye1 147108

d1juma1 67304

d1jumb1 67306

d1jumd1 67308

d1jume1 67310

d1jupa1 67314

d1jupb1 67316

d1jupd1 67318

d1jupe1 67320

d1jtya1 67292

d1jtyb1 67294

d1jtyd1 67296

d1jtye1 67298

d2gena1 135062

d2fd5a1 133291

d2gfna1 135101

d2gfnb1 135103

d2d6ya1 131313

d2d6yb1 131315

d2oi8a1 148778

d2g3ba1 134558

d2g3bb1 134560

d2g7sa1 134737

d2g7ga1 134732

d2hkua1 147302

d2hkub1 147304

d2i10a1 136960

d2i10b1 136962

d2np3a1 138420

d2np3b1 138422

d3c07a1 155807

d2hyja1 147456

d2id3a1 137261

d2id3b1 137263

d2g7la1 134734

d1sgma1 105537

d1sgmb1 105539

d1t33a1 106295

d1t33b1 106297

d2vkva1 153230

d2vkea1 153221

d2o7oa1 138930

d2fj1a1 133541

d2tcta1 16063

d1bjza1 16065

d2x9da1 169968

d2trta1 16064

d1orka1 16067

d1a6ia1 16066

d1bj0a1 16068

d1bjya1 16069

d1bjyb1 16070

d1qpia1 16072

d2iu5a1 147801

d2iu5b1 147803

d2fq4a1 133937

d1zk8a1 125177

d1zk8b1 125179

d2o7ta1 148670

d2zoya1 171391

d2zoyb1 171393

d2zoza1 154740

d1z0xa1 124334

d2fbqa1 133256

d2np5a1 138424

d2np5b1 138426

d2np5c1 138428

d2np5d1 138430

d2ieka1 147647

d1z77a1 124588

d1zkga1 125194

d1zkgb1 125196

d1irza_ 76772

d1ntca_ 19004

d1ntcb_ 19005

d4ihwb_ 193093

d1etxa_ 18978

d1etxb_ 18979

d1fipa_ 18980

d1fipb_ 18981

d1etoa_ 18982

d1etob_ 18983

d1etva_ 18986

d1etvb_ 18987

d1fiaa_ 18984

d1fiab_ 18985

d1etwa_ 18988

d1etwb_ 18989

d1etya_ 18990

d1etyb_ 18991

d1etka_ 18992

d1etkb_ 18993

d3fisa_ 18994

d3fisb_ 18995

d4fisa_ 18996

d4fisb_ 18997

d1f36a_ 18998

d1f36b_ 18999

d1etqa_ 19000

d1etqb_ 19001

d1etqc_ 19002

d1etqd_ 19003

d1umqa_ 99625

d1g2ha_ 60224

d3iv5a_ 178622

d3iv5b_ 178623

d3jr9a_ 178720

d3jr9b_ 178721

d3jrha_ 178722

d3jrhb_ 178723

d1ofcx2 92827

d1rr7a_ 105075

d2coba1 130673

d2ga1a1 134850

d2ga1b_ 134851

d2ao9a1 127069

d2dw4a1 146594

d2z3ya1 154024

d2z5ua1 154167

d2iw5a1 145539

d2ejra1 146877

d2uxxa1 152310

d2uxna1 145760

d2h94a1 147245

d2coma1 130679

d2fq3a1 133936

d2aqfa1 127180

d2cuja1 130812

d2aqea1 127179

d2jn6a1 148144

d2o3fa1 148573

d2h1ka_ 164912

d2h1kb_ 164913

d3a03a_ 171621

d2vi6a_ 168625

d2vi6b_ 168626

d2vi6c_ 168627

d2vi6d_ 168628

d2vi6e_ 168629

d2vi6f_ 168630

d2vi6g_ 168631

d2vi6h_ 168632

d1sfea1 16073

d1qnta1 16074

d1eh7a1 16075

d1eh6a1 16076

d1eh8a1 16077

d1yfha1 123062

d1yfhb1 123064

d1yfhc1 123066

d1t38a1 106333

d1t39a1 106335

d1t39b1 106337

d1mgta1 16078

d1c20a_ 16079

d1kqqa_ 72885

d1ig6a_ 62364

d2oeha1 148752

d1ryua_ 105127

d1kkxa_ 72662

d1kn5a_ 72769

d2cxya_ 163507

d2eh9a_ 164025

d1biaa1 16083

d1hxda1 61360

d1hxdb1 61363

d1biba1 16084

d2ewna1 132470

d1j5ya1 71592

d1jhfa1 63057

d1jhha1 63060

d1leaa_ 16085

d1leba_ 16086

d1b4aa1 16088

d1b4ab1 16089

d1b4ac1 16090

d1b4ad1 16091

d1b4ae1 16092

d1b4af1 16093

d2p5ka1 149249

d1f9na1 64990

d1f9nb1 64992

d1f9nc1 64994

d1f9nd1 64996

d1f9ne1 64998

d1f9nf1 65000

d2p5lc1 149250

d2p5ld1 149251

d2p5lg1 149252

d2p5lh1 149253

d1aoya_ 16087

d1i5za1 83669

d1i5zb1 83671

d1hw5a1 16098

d1hw5b1 16099

d1zrfb1 125545

d1g6na1 16100

d1g6nb1 16101

d1i6xa1 83673

d1i6xb1 83675

d2gzwa1 135917

d2gzwb1 135919

d2gzwc1 135921

d2cgpa1 16102

d1j59a1 71532

d1j59b1 71534

d1runa1 16103

d1runb1 16104

d1zrda1 125535

d1zrdb1 125537

d1zrea1 125539

d1zreb1 125541

d1zrca1 125531

d1zrcb1 125533

d1ruoa1 16105

d1ruob1 16106

d1o3ra1 86607

d1lb2a1 77869

d1o3qa1 86605

d1cgpa1 16096

d1cgpb1 16097

d1o3sa1 86609

d1o3ta1 86611

d1o3tb1 86613

d2h6ca1 147232

d2h6cb1 147234

d3e5ua1 158022

d3e5ub1 158024

d3e5uc1 158026

d2h6ba1 147228

d1ft9a1 16107

d1ft9b1 16108

d2oz6a1 149103

d2bgca1 128458

d2bgcb1 128460

d2bgcd1 128462

d2bgce1 128464

d2bgcf1 128466

d2bgcg1 128468

d2bgch1 128470

d2bgci1 128472

d2beoa1 128381

d2beob1 128383

d1omia2 87080

d1omib2 87082

d1zyba1 125815

d2gaua1 134894

d2zcwa1 171157

d1u2wa1 119487

d1u2wb_ 119488

d1u2wc_ 119489

d1u2wd_ 119490

d1r1ua_ 104771

d1r1ub_ 104772

d1r1uc_ 104773

d1r1ud_ 104774

d1r1va_ 104775

d1r1vb_ 104776

d1y0ua_ 116310

d1y0ub_ 116311

d1r1ta_ 104769

d1r1tb_ 104770

d1r23a_ 104779

d1r23b_ 104780

d1smta_ 16109

d1smtb_ 16110

d1r22a_ 104777

d1r22b_ 104778

d3f72a_ 175529

d3f72b_ 175530

d3f72c_ 175531

d3f72d_ 175532

d3f72e_ 175533

d3f72f_ 175534

d4ggga_ 193061

d4gggb_ 193062

d1hw1a1 16111

d1hw1b1 16112

d1e2xa1 16113

d1h9ga1 60827

d1hw2a1 16114

d1hw2b1 16115

d1h9ta1 60847

d1h9tb1 60849

d2hs5a1 147379

d3bwga1 155693

d1v4ra1 119835

d1bm9a_ 16116

d1bm9b_ 16117

d1f4ka_ 59646

d1f4kb_ 59647

d1j0ra_ 90745

d1j0rb_ 90746

d2efwa_ 146820

d2efwb_ 146821

d2efwf_ 146822

d2efwg_ 146823

d2dpua1 131617

d2dpda1 131610

d2dpdb1 131611

d1b9ma1 16118

d1b9mb1 16119

d1b9na1 16120

d1b9nb1 16121

d1o7la1 81147

d1o7lb1 81150

d1o7lc1 81153

d1o7ld1 81156

d1bjaa_ 16122

d1bjab_ 16123

d1i1sa_ 16124

d1repc1 16125

d1repc2 16126

d2z9oa1 154260

d2z9oa2 154261

d2z9ob1 154262

d2z9ob2 154263

d2nrac1 148360

d2nrac2 148361

d1hkqa_ 83555

d1hkqb_ 83556

d1fnna1 16129

d1fnnb1 16130

d1w5sa1 114251

d1w5sb1 114253

d1w5ta1 114255

d1w5tb1 114257

d1w5tc1 114259

d1in4a1 62597

d1j7ka1 62695

d1in6a1 62601

d1in7a1 62603

d1in8a1 62605

d1in5a1 62599

d1ixsb1 76933

d1hqca1 16127

d1hqcb1 16128

d1ixrc1 76930

d2fnaa1 133809

d2fnab1 133811

d2foka1 16131

d2foka2 16132

d2foka3 16133

d2fokb1 16134

d2fokb2 16135

d2fokb3 16136

d1foka1 16137

d1foka2 16138

d1foka3 16139

d1yqaa1 123875

d1usta_ 99884

d1ussa_ 99883

d1uhma_ 99398

d1ghca_ 16142

d1hsta_ 16140

d1hstb_ 16141

d1d5va_ 16144

d3l2ca_ 179869

d1e17a_ 16143

d2a07f1 125938

d2hfha_ 16145

d2hdca_ 16146

d1kq8a_ 68797

d1jxsa_ 84254

d3co6c_ 173367

d2c6ya_ 130011

d2c6yb_ 130012

d1vtnc_ 196324

d3qrff_ 184593

d3qrfg_ 184594

d3qrfh_ 184595

d3qrfi_ 184596

d2bbya_ 16148

d1bbya_ 16149

d1dpua_ 16150

d1z1da1 124351

d1cf7b_ 16152

d1cf7a_ 16151

d1d8ka_ 16154

d1d8ja_ 16153

d1sfua_ 105503

d1sfub_ 105504

d1j75a_ 62673

d1oyia_ 93729

d1xmka1 122165

d1qbja_ 16155

d1qbjb_ 16156

d1qbjc_ 16157

d2gxba1 135830

d2gxbb1 135831

d3f21a_ 175416

d3f21b_ 175417

d3f21c_ 175418

d3f22a_ 175419

d3f22b_ 175420

d3f22c_ 175421

d3f23a_ 175422

d3f23b_ 175423

d3f23c_ 175424

d2acja1 126555

d2acjb1 126556

d2acjc1 126557

d2acjd1 126558

d1qgpa_ 16158

d2heoa_ 165075

d2heod_ 165076

d1dp7p_ 16159

d1ka8a_ 72241

d1ka8b_ 72242

d1ka8c_ 72243

d1ka8d_ 72244

d1ka8e_ 72245

d1ka8f_ 72246

d1wwxa1 121379

d1duxc_ 16170

d1duxf_ 16171

d1gvja_ 90525

d1gvjb_ 90526

d2stta_ 16163

d2stwa_ 16164

d1md0a_ 79000

d1md0b_ 79001

d1k78b_ 68257

d1k78f_ 68260

d1k79a_ 68262

d1k79d_ 68263

d1k7aa_ 68264

d1k7ad_ 68265

d1mdmb_ 79012

d1r36a_ 111675

d1flia_ 16160

d1awca_ 16167

d1yo5c1 123768

d1bc8c_ 16168

d1bc7c_ 16169

d1k6oa_ 68226

d1hbxg_ 60934

d1hbxh_ 60935

d1puee_ 16165

d1puef_ 16166

d2yprb_ 193284

d3jtga_ 178823

d1hksa_ 16172

d1hkta_ 16173

d2htsa_ 16174

d3htsb_ 16175

d1fbua_ 16176

d1fbub_ 16177

d1fbqa_ 16178

d1fbqb_ 16179

d1fbsa_ 16180

d1fbsb_ 16181

d1fyma_ 65070

d1fymb_ 65071

d1fyla_ 65068

d1fylb_ 65069

d1fyka_ 65067

d3hsfa_ 16182

d1if1a_ 16183

d1if1b_ 16184

d2pi0a_ 149489

d2pi0b_ 149490

d2pi0c_ 149491

d2pi0d_ 149492

d2o6ge1 148628

d2o6gf1 148629

d2o6gg1 148630

d2o6gh1 148631

d1t2ka_ 112220

d1t2kb_ 112221

d2irfg_ 16185

d2irfh_ 16186

d2irfi_ 16187

d2irfj_ 16188

d2irfk_ 16189

d2irfl_ 16190

d1irfa_ 16192

d1irga_ 16191

d3qu6a_ 184629

d3qu6b_ 184630

d3qu6c_ 184631

d2dtra1 16193

d1g3wa1 65133

d1g3sa1 65124

d1bi1a1 16196

d1bi0a1 16202

d1fwza1 60077

d1p92a1 104085

d1xcva1 121862

d1g3ta1 65127

d1g3tb1 65130

d1bi3a1 16199

d1bi3b1 16200

d1bi2a1 16197

d1bi2b1 16198

d2tdxa1 16201

d1g3ya1 65136

d1f5ta1 16203

d1f5tb1 16204

d1f5tc1 16205

d1f5td1 16206

d1ddna1 16207

d1ddnb1 16208

d1ddnc1 16209

d1ddnd1 16210

d1dpra1 16194

d1dprb1 16195

d1c0wa1 16211

d1c0wb1 16212

d1c0wc1 16213

d1c0wd1 16214

d2isya1 137612

d2isyb1 137614

d1fx7a1 60088

d1fx7b1 60091

d1fx7c1 60094

d1fx7d1 60097

d2isza1 137616

d2iszb1 137618

d2iszc1 137620

d2iszd1 137622

d2it0a1 137624

d2it0b1 137626

d2it0c1 137628

d2it0d1 137630

d1u8ra1 113168

d1u8rb1 113171

d1u8rc1 113174

d1u8rd1 113177

d1u8rg1 113180

d1u8rh1 113183

d1u8ri1 113186

d1u8rj1 113189

d1b1ba1 16215

d2ev0a1 132413

d2ev0b1 132415

d1on2a1 87103

d1on2b1 87105

d2ev6a1 132421

d2ev6b1 132423

d1on1a1 87099

d1on1b1 87101

d2f5da1 132979

d2f5db1 132981

d2ev5a1 132417

d2ev5b1 132419

d2f5ea1 132983

d2f5eb1 132985

d2f5fa1 132987

d2f5fb1 132989

d2f5ca1 132977

d2hyfa1 136880

d2hyfb1 136882

d2hygd1 136888

d1b6aa1 16223

d1qzya1 96717

d1bn5a1 16224

d1yw9a1 124138

d1b59a1 16225

d1boaa1 16226

d1kq9a1 91021

d1yw7a1 124134

d1r58a1 111695

d1kq0a1 91018

d2ga2a1 134852

d1r5ga1 111702

d2oaza1 138999

d1r5ha1 111704

d2adua1 126595

d2ea4a1 146759

d2ea2a1 146757

d1yw8a1 124136

d1xgsa1 16216

d1xgsb1 16217

d1xgna1 16218

d1xgnb1 16219

d1xgma1 16220

d1xgmb1 16221

d1xgoa1 16222

d1t0fa1 112195

d1t0fb1 112197

d1f1za1 16081

d1f1zb1 16082

d1ub9a_ 99159

d3echa_ 174825

d3echb_ 174826

d3mexa_ 181031

d3mexb_ 181032

d1lnwa_ 78104

d1lnwb_ 78105

d1lnwc_ 78106

d1lnwd_ 78107

d1lnwe_ 78108

d1lnwf_ 78109

d1lnwg_ 78110

d1lnwh_ 78111

d1jgsa_ 66683

d1z91a1 124736

d1z9ca_ 124747

d1z9cb_ 124748

d1z9cc_ 124749

d1z9cd_ 124750

d1z9ce_ 124751

d1z9cf_ 124752

d2frha_ 133988

d2frhb_ 133989

d2fnpa1 133825

d2fnpb_ 133826

d1fzpb_ 19008

d1fzpd_ 19007

d2hr3a1 136678

d2hr3b_ 136679

d2hr3c_ 136680

d2hr3d_ 136681

d2fbia1 133246

d2fxaa1 134304

d2etha1 132358

d2ethb_ 132359

d1s3ja_ 98437

d1s3jb_ 98438

d1hsja1 61237

d1hsjb1 61239

d1p4xa1 87784

d1p4xa2 87785

d3ctaa1 156979

d2fbka1 133247

d2bv6a1 146218

d3broa1 155517

d3brob_ 155518

d3broc_ 155519

d3brod_ 155520

d2fbha1 133245

d1lj9a_ 78035

d1lj9b_ 78036

d3deua1 157600

d3qpta_ 184556

d3q5fa_ 184218

d3q5fb_ 184219

d2a61a1 126187

d2fbkb_ 133248

d3voda_ 193220

d3vodb_ 193221

d3vb2a_ 194054

d3vb2b_ 194053

d3voea_ 193219

d2a61b_ 126188

d2a61c_ 126189

d2a61d_ 126190

d4aihe_ 193530

d1qzza1 96719

d1r00a1 96721

d1xdsa1 115174

d1xdsb1 115176

d1xdua1 115178

d1kyza1 73312

d1kyzc1 73314

d1kyze1 73316

d1kywa1 73296

d1kywc1 73298

d1kywf1 73300

d1tw3a1 107373

d1tw3b1 107375

d1tw2a1 107369

d1fp1d1 59939

d1fpqa1 59947

d1fp2a1 59941

d1fpxa1 59950

d1i27a_ 61555

d1j2xa_ 77066

d1nhaa_ 80509

d1onva_ 87171

d2csoa1 130778

d1w4ma_ 114193

d1uhwa_ 99410

d1v3fa_ 100289

d1o7fa1 81131

d1fsha_ 60006

d1i1ga1 65982

d1i1gb1 65984

d2cyya1 131028

d1ri7a1 97504

d2cg4a1 130417

d2cg4b1 130419

d2cfxa1 130395

d2cfxb1 130397

d2cfxc1 130399

d2cfxd1 130401

d2cfxe1 130403

d2cfxf1 130405

d2cfxg1 130407

d2cfxh1 130409

d1mkma1 79242

d1mkmb1 79244

d1ldda_ 73841

d1lddb_ 73842

d1lddc_ 73843

d1lddd_ 73844

d1ldja1 73847

d1u6ga1 113062

d1ldkb1 73852

d1iuya_ 90705

d2hyec1 145414

d2v9va1 152809

d2v9va2 152810

d1lvaa1 74276

d1lvaa2 74277

d1lvaa3 74278

d1lvaa4 74279

d2uwma1 139992

d2uwma2 139993

d2uwma3 139994

d2uwmb1 139995

d2uwmb2 139996

d2uwmb3 139997

d1wsua1 121245

d1wsua2 121246

d1wsub1 121247

d1wsub2 121248

d1wsuc1 121249

d1wsuc2 121250

d1wsud1 121251

d1wsud2 121252

d2plya1 149651

d2plya2 149652

d2plya3 149653

d2plya4 149654

d1ku9a_ 77544

d1ku9b_ 77545

d1ixca1 83764

d1ixcb1 83766

d1iz1a1 83823

d1iz1b1 83825

d1iz1p1 83827

d1iz1q1 83829

d2esna1 132333

d2esnd1 132339

d2dt5a1 131708

d2dt5b1 131710

d1xcba1 109552

d1xcbb1 109554

d1xcbc1 109556

d1xcbd1 109558

d1xcbe1 109560

d1xcbf1 109562

d1xcbg1 109564

d2g9wa1 147097

d1okra_ 93269

d1okrb_ 93270

d1sd6a_ 98803

d1sd6b_ 98804

d1sd7a_ 98805

d1sd7b_ 98806

d1saxa_ 98787

d1saxb_ 98788

d2d45a1 131242

d2d45b1 131243

d2d45c1 131244

d2d45d1 131245

d1p6ra_ 94187

d2p7cb1 139517

d1sd4a_ 105428

d1sd4b_ 105429

d1xsda_ 122269

d1p4aa1 94090

d1p4ab1 94092

d1p4ac1 94094

d1p4ad1 94096

d1o57a1 92483

d1o57b1 92485

d1o57c1 92487

d1o57d1 92489

d1q1ha_ 95580

d1mzba_ 91497

d1oywa1 93760

d1oyya1 93772

d2p6ra1 149270

d2p6ua1 149274

d2axla1 127495

d1pp7u_ 94973

d1pp8f_ 94974

d1pp8m_ 94975

d1pp8o_ 94976

d1pp8p_ 94977

d1pp8u_ 94978

d1pp8v_ 94979

d1ucra_ 99188

d1ucrb_ 99189

d1wq2a_ 121161

d1wq2b_ 121162

d2cqka1 130725

d1zh5a1 125073

d1zh5b1 125075

d2vooa1 153373

d2voob1 153375

d2vona1 153369

d2vonb1 153371

d2voda1 153365

d2vodb1 153367

d1ytya1 124018

d1ytyb1 124020

d2vopa1 153377

d1s7aa_ 98633

d1s29a_ 98373

d1ufma_ 107816

d1wi9a_ 114663

d1tbxa_ 106748

d1tbxb_ 106749

d2co5a1 146418

d2pg4a1 149455

d2pg4b_ 149456

d2co5b_ 146419

d1r7ja_ 104836

d1xsxa1 122289

d1xsxb1 122290

d1sfxa_ 105505

d1sfxb_ 105506

d2d1ha1 131125

d2d1hb_ 131126

d1stza1 106009

d1stzb1 106011

d1stzc1 106013

d2zxxc_ 171598

d2zxxf_ 171601

d1rz4a1 105131

d1xb4a1 121832

d1xb4a2 121833

d1xb4b1 121834

d1xb4b2 121835

d1xb4c1 121836

d1xb4c2 121837

d1xb4d1 121838

d1xb4d2 121839

d1u5tc1 107688

d1u5tc2 107689

d1u5td1 107690

d1u5td2 107691

d1w7pb1 114321

d1w7pb2 114322

d1w7pc1 114323

d1w7pc2 114324

d1u5tb1 107686

d1u5tb2 107687

d1u5ta1 107684

d1u5ta2 107685

d1w7pa1 114319

d1w7pa2 114320

d1ylfa1 123648

d1xd7a_ 109566

d1zara1 124843

d1zaoa1 124841

d1tqia1 107226

d1tqma1 107236

d1tqpa1 107240

d1w1we_ 114084

d1w1wf_ 114085

d1w1wg_ 114086

d1w1wh_ 114087

d1ulya_ 113297

d2cwea1 130921

d1wj5a_ 114692

d1t6sa1 112271

d1t6sa2 112272

d1t6sb1 112273

d1t6sb2 112274

d1yg2a_ 116681

d2esha1 132325

d1xmaa_ 115477

d1xmab_ 115478

d1xn7a_ 115576

d4awxb_ 193943

d1z6ra1 124557

d1z6rb1 124560

d1z6rc1 124563

d1z6rd1 124566

d3bp8a1 155463

d3bp8b1 155466

d2hoea1 136639

d1z05a1 124298

d2p4wa_ 139495

d2p4wb_ 139496

d1t98a1 119196

d2b0la1 127641

d2b0lb_ 127642

d2b0lc_ 127643

d2j5pa1 138050

d2j5oa1 138049

d2ve8a_ 153008

d2ve8b_ 153009

d2ve8c_ 153010

d2ve8d_ 153011

d2ve8e_ 153012

d2ve8f_ 153013

d2ve8g_ 153014

d2ve8h_ 153015

d2ve9a_ 153016

d2ve9b_ 153017

d2ve9c_ 153018

d2ve9d_ 153019

d2ve9e_ 153020

d2ve9f_ 153021

d2fb1a1 133225

d2fb1b1 133227

d2fb1c1 133229

d2fb1d1 133231

d2fmla1 133778

d1z7ua1 124672

d2f2ea1 132808

d2f2eb_ 132809

d2fswa1 134042

d2fswb_ 134043

d2hzta1 147462

d1yyva1 124254

d1yyvb_ 124255

d1z7ub_ 124673

d2od5a1 148736

d2obpa1 148718

d2obpb_ 148719

d2p8ta1 149315

d2htja1 147397

d2ipqx1 147770

d3bz6a1 155739

d3bz6a2 155740

d2ns0a1 148381

d2hgca1 147283

d2vqca1 153426

d1s6la1 145773

d2a5yb1 144786

d2doaa1 146548

d2gmga1 147131

d1zela1 145994

d2v7fa1 152721

d2dk5a1 146534

d2dk8a1 146535

d4esfa_ 193767

d3bjaa_ 172659

d4esba_ 194251

d1ylfb_ 123649

d1ylfc_ 123650

d2hztb_ 147463

d2hztc_ 147464

d2hztd_ 147465

d4etsa_ 195106

d4etsb_ 195105

d3jw4a_ 178861

d3jw4b_ 178862

d3jw4c_ 178863

d3hhha_ 177574

d3hhhb_ 177575

d2xiga_ 170114

d2xigb_ 170115

d2xigc_ 170116

d2xigd_ 170117

d2a07g_ 125939

d2a07h_ 125940

d2a07i_ 125941

d2a07j_ 125942

d2a07k_ 125943

d2as5f_ 127235

d2as5g_ 127236

d3k69a_ 179112

d3f8ba_ 175565

d3f8bb_ 175566

d3f8ca_ 196111

d3qu3a_ 184626

d3qu3b_ 184627

d3qu3c_ 184628

d2o03a_ 166480

d4hqma_ 193083

d4hqmb_ 193084

d3ecoa_ 174833

d3ecob_ 174834

d3l7wa_ 180067

d4i7ha_ 193092

d4i7hb_ 193091

d3mwmb_ 196368

d3eyyb_ 196572

d3elkb_ 196080

d3df8a_ 173882

d1opca_ 16231

d1odda_ 16232

d2jpba1 138360

d1gxqa_ 70721

d1gxpa_ 70717

d1gxpb_ 70718

d1gxpe_ 70719

d1gxpf_ 70720

d2z33a1 153956

d1qqia_ 16233

d1kgsa1 68596

d2ff4a1 133367

d2ff4b1 133370

d2feza1 133357

d1p2fa1 87720

d1ys7a1 123961

d1ys7b1 123963

d1ys6a1 123957

d1ys6b1 123959

d1fsea_ 60000

d1fseb_ 60001

d1fsec_ 60002

d1fsed_ 60003

d1fsee_ 60004

d1fsef_ 60005

d1a04a1 16234

d1a04b1 16235

d1je8a_ 77108

d1je8b_ 77109

d1je8e_ 77110

d1je8f_ 77111

d1rnla1 16236

d1zg5a1 125010

d1zg5b1 125011

d1zg5e1 125012

d1zg5f1 125013

d1zg1a1 125005

d1zg1b1 125006

d1zg1e1 125007

d1zg1f1 125008

d1l3la1 73541

d1l3lb1 73543

d1l3lc1 73545

d1l3ld1 73547

d1h0ma1 76442

d1h0mb1 76444

d1h0mc1 76446

d1h0md1 76448

d1yioa1 123326

d1zn2a1 125371

d1p4wa_ 87783

d1fc3a_ 16237

d1fc3b_ 16238

d1fc3c_ 16239

d1lq1a_ 74179

d1lq1b_ 74180

d1lq1c_ 74181

d1lq1d_ 74182

d3c57b_ 195256

d1zlja_ 162519

d1zljb_ 162520

d1zljc_ 162521

d1zljd_ 162522

d1zlje_ 162523

d1zljf_ 162524

d1zljg_ 162525

d1zljh_ 162526

d1hc8a_ 70963

d1hc8b_ 70964

d1y39a1 145899

d1y39b1 145900

d1qa6a_ 16240

d1qa6b_ 16241

d1foxa_ 16242

d1fowa_ 16243

d1foya_ 16245

d2fowa_ 16244

d1acia_ 16246

d3cf5f1 156545

d2zjqf1 154529

d2zjpf1 154497

d1xbpg1 145872

d2qami1 150248

d2qaoi1 150301

d2qbei1 150468

d2qbgi1 150522

d2i2ti1 145450

d2i2vi1 145492

d2qozi1 151124

d2qp1i1 151177

d3df4i1 157691

d3df2i1 157637

d2qbai1 150361

d2qbci1 150414

d2qoxi1 151071

d2qovi1 151018

d1vs8i1 144506

d2qbii1 150576

d2qbki1 150630

d2awbi1 144915

d2aw4i1 144874

d1vs6i1 144465

d2vhmi1 153071

d2vhni1 153103

d2z4li1 154086

d2z4ni1 154140

d3degh1 157574

d2rdoi1 151948

d2gycg1 145267

d2j28i1 145631

d2gyag1 145245

d1vq8i1 120196

d1vqoi1 120370

d1vqpi1 120399

d1yhqi1 123191

d3cc2i1 156178

d1s72i_ 105328

d1vqmi1 120312

d1vqli1 120283

d1vqki1 120254

d1vqni1 120341

d1yiji1 123306

d1yi2i1 123238

d3ccmi1 156339

d1vq7i1 120167

d1vq9i1 120225

d3cc7i1 156227

d1vq5i1 120109

d3ccei1 156267

d3ccui1 156435

d1vq4i1 120080

d1vq6i1 120138

d3ccvi1 156459

d1yiti1 123349

d3cd6i1 156483

d3ccli1 156315

d1yjwi1 123473

d3cc4i1 156203

d3ccji1 156291

d2otli1 139354

d3ccqi1 156363

d3cmai1 156779

d3ccsi1 156411

d2otji1 139325

d3ccri1 156387

d2qexi1 150697

d1yjni1 123441

d1yj9i1 123402

d3cmei1 156816

d2qa4i1 150182

d1mmsa1 16247

d1mmsb_ 16248

d3cjrb1 156712

d3cjtb1 156716

d3cjtf1 156718

d3cjtj1 156720

d3cjtn1 156722

d2nxnb1 145699

d3cjqb1 156703

d3cjqe1 156706

d3cjqh1 156709

d2h8wa1 147243

d2e35a1 146672

d2e36a1 146674

d2e34a1 146670

d2hgql1 145346

d2hgjl1 145314

d2hgul1 145378

d1yl3l1 123586

d2b66k1 127961

d2b9pk1 128190

d2b9nk1 128153

d2qanr1 150284

d2qalr1 150230

d2qbfr1 150504

d2qbdr1 150450

d2qoyr1 151107

d2qp0r1 151160

d3df1r1 157620

d3df3r1 157674

d2avyr1 144859

d2aw7r1 144902

d1vs5r1 144452

d1vs7r1 144493

d2qb9r1 150344

d2qbbr1 150397

d2i2pr1 145437

d2i2ur1 145479

d2qour1 151001

d2qowr1 151054

d2qbhr1 150558

d2qbjr1 150612

d2vhor1 153140

d2z4kr1 154068

d2z4mr1 154122

d2vhpr1 153162

d2uubr1 139949

d2vqer1 153442

d2vqfr1 153461

d2uuar1 139929

d1j5er_ 71561

d1fjgr_ 16252

d2uucr1 139969

d2uxcr1 140021

d1xmqr_ 115547

d2uu9r1 139909

d1n32r_ 79887

d1xnqr_ 115621

d1xnrr_ 115643

d1hr0r_ 16253

d1hnzr_ 16254

d2j02r1 137883

d2j00r1 137856

d1xmor_ 115517

d1i94r_ 62009

d2uxdr1 152298

d1hnwr_ 16255

d1hnxr_ 16256

d3d5cr1 157381

d3d5ar1 157351

d2e5lr1 132042

d1n33r_ 79909

d2hhhr1 136494

d2uxbr1 152279

d1n34r_ 79931

d2v48r1 152536

d1i96r_ 62053

d2v46r1 152500

d2f4vr1 132955

d1n36r_ 79954

d1i97r_ 62076

d1i95r_ 62031

d2hgpu1 136437

d2hgiu1 136416

d2hgru1 136458

d2qnhs1 150942

d2ow8s1 139416

d1yl4u1 123614

d1x18h1 121576

d2b9or1 128180

d2b64r1 127950

d2b9mr1 128143

d1g1xc_ 16249

d1g1xh_ 16250

d1whua_ 114651

d1e3pa1 16258

d1e3ha1 16257

d1k6ya1 68239

d1k6yb1 68241

d1k6yc1 68243

d1k6yd1 68245

d1wjfa_ 16267

d1wjfb_ 16268

d1wjca_ 16261

d1wjcb_ 16262

d1wjba_ 16265

d1wjbb_ 16266

d1wjda_ 16269

d1wjdb_ 16270

d1wjea_ 16263

d1wjeb_ 16264

d1wjaa_ 16259

d1wjab_ 16260

d1e0ea_ 59147

d1e0eb_ 59148

d1twfj_ 112735

d1i50j_ 61764

d1k83j_ 68285

d1i3qj_ 61617

d1twcj_ 112721

d1twaj_ 112706

d1twhj_ 112761

d1i6hj_ 61841

d1twgj_ 112748

d2r7zj1 151664

d2r92j1 151751

d2nvyj1 138690

d2e2ij1 132004

d2nvtj1 138657

d2e2jj1 132017

d2vumj1 153554

d2r93j1 151760

d2ja7j1 138203

d2ja7v1 138219

d2nvxj1 138677

d2b63j1 127929

d2ja5j1 138171

d2ja8j1 138235

d2yu9j1 140074

d2ja6j1 138187

d2e2hj1 131991

d2nvzj1 138703

d2b8kj1 128089

d1ef4a_ 16272

d3cqzj_ 156933

d2nvqj_ 138644

d1jhga_ 19009

d3ssxn_ 185511

d3ssxr_ 185512

d3sswn_ 185509

d3sswr_ 185510

d2oz9r_ 139442

d1zt9a_ 125631

d1zt9b_ 125632

d1zt9d_ 125633

d1zt9e_ 125634

d1troa_ 19011

d1troc_ 19012

d1troe_ 19013

d1trog_ 19014

d3wrpa_ 19015

d1wrpr_ 19016

d1trra_ 19017

d1trrb_ 19018

d1trrd_ 19019

d1trre_ 19020

d1trrg_ 19021

d1trrh_ 19022

d1trrj_ 19023

d1trrk_ 19024

d1mi7r_ 91278

d1rcsa_ 19025

d1rcsb_ 19026

d1wrsr_ 19027

d1wrss_ 19028

d1wrtr_ 19029

d1wrts_ 19030

d1co0a_ 90426

d1co0b_ 90427

d1l8qa1 77809

d2hcba1 136325

d2hcbb1 136327

d2hcbc1 136329

d2hcbd1 136331

d1j1va_ 83981

d2oa4a1 148690

d1ku2a1 83096

d1ku2b1 83098

d1rp3a1 97678

d1rp3c1 97682

d1rp3e1 97686

d1rp3g1 97690

d1sc5a1 98798

d1smyf1 105781

d1smyp1 105791

d2a6hf1 126267

d2a6hp1 126277

d2be5f1 128358

d2be5p1 128368

d2a68f1 126198

d2a68p1 126208

d2a69f1 126218

d2a69p1 126228

d1iw7f1 83086

d1iw7p1 83089

d2a6ef1 126247

d2a6ep1 126257

d1zyrf1 125856

d1zyrp1 125866

d2cw0f1 130905

d2cw0p1 130915

d1rp3a2 97679

d1rp3c2 97683

d1rp3e2 97687

d1rp3g2 97691

d1sc5a2 98799

d2p7vb_ 149292

d1tlhb_ 112507

d1ttya_ 112638

d1ku3a_ 73000

d1rioh_ 97515

d1ku7a_ 73001

d1ku7d_ 73002

d1smyf2 105782

d1smyp2 105792

d2a6hf2 126268

d2a6hp2 126278

d2be5f2 128359

d2be5p2 128369

d2a68f2 126199

d2a68p2 126209

d2a69f2 126219

d2a69p2 126229

d1iw7f2 83087

d1iw7p2 83090

d2a6ef2 126248

d2a6ep2 126258

d1zyrf2 125857

d1zyrp2 125867

d2cw0f2 130906

d2cw0p2 130916

d1or7a1 87332

d1or7b1 87334

d2h27a1 135993

d2h27d1 135994

d1l0oc_ 73405

d4g6da_ 193282

d1xsva_ 116003

d1xsvb_ 116004

d1s7oa_ 105354

d1s7ob_ 105355

d1s7oc_ 105356

d1vz0a1 108929

d1vz0b1 108931

d1vz0c1 108933

d1vz0d1 108935

d1vz0e1 108937

d1vz0f1 108939

d1vz0g1 108941

d1vz0h1 108943

d1r71a_ 104823

d1r71b_ 104824

d1r71c_ 104825

d1r71d_ 104826

d1rq6a_ 111907

d1cuka1 16273

d1hjpa1 16274

d1c7ya1 16275

d1bdxa1 16276

d1bdxb1 16277

d1bdxc1 16278

d1bdxd1 16279

d1bvsa1 16280

d1bvsb1 16281

d1bvsc1 16282

d1bvsd1 16283

d1bvse1 16284

d1bvsf1 16285

d1bvsg1 16286

d1bvsh1 16287

d1ixsa_ 76932

d1ixrb1 76927

d1veja1 120020

d1pgya_ 94696

d2cpwa1 130707

d1qzea1 96629

d1qzea2 96630

d1dv0a_ 16289

d1f4ia_ 16288

d1ifya_ 71207

d1oqya1 93439

d1oqya2 93440

d2bwea1 129342

d2bweb1 129343

d2bwec1 129344

d2bwed1 129345

d2bwee1 129346

d2bwef1 129347

d2bweg1 129348

d2bweh1 129349

d2bwei1 129350

d2bwej1 129351

d2bwek1 129352

d2bwel1 129353

d2bwem1 129354

d2bwen1 129355

d2bweo1 129356

d2bwep1 129357

d2bweq1 129358

d2bwer1 129359

d1wr1b1 121185

d2g3qa1 145181

d2cp8a1 130695

d1vega_ 113634

d1vg5a_ 113640

d2k0bx1 148253

d2jy7a1 148239

d1q02a_ 95490

d2jy8a1 148240

d2cosa1 130683

d2crna1 130741

d2dkla1 131555

d1wjia_ 114698

d1z96a1 145960

d1z96b_ 145961

d1whca_ 114640

d2daha1 131352

d2dnaa1 131586

d1vdla_ 108528

d1veka_ 113636

d1wiva_ 114680

d1wgna_ 114617

d1wj7a1 120976

d1ylaa1 123641

d1ttea1 107295

d2bwba_ 129329

d2bwbb_ 129330

d2bwbc_ 129331

d2bwbd_ 129332

d2bwbe_ 129333

d2bwbf_ 129334

d2bwbg_ 129335

d2bwbh_ 129336

d2bwbi_ 129337

d3b0fa_ 172401

d3b0fb_ 172402

d1xb2b1 115057

d1efub3 58975

d1efud3 58977

d2cp9a1 130696

d1aipc1 58930

d1aipd1 58932

d1aipg1 58934

d1aiph1 58936

d1oaia_ 81255

d1go5a_ 65410

d1v92a_ 100531

d2di0a1 131521

d1otra_ 87413

d1wgla_ 114615

d1mn3a_ 85024

d1p3qq_ 87748

d1p3qr_ 87749

d1r5la1 97099

d1oiza1 93079

d1oizb1 93081

d1oipa1 93071

d1auaa1 16290

d1olma1 104008

d1olmc1 104011

d1olme1 104014

d1o6ua1 92589

d1o6uc1 92592

d1o6ue1 92595

d1enwa_ 16291

d1eija_ 16298

d2crua1 130742

d1sr9a1 105960

d1sr9b1 105963

d1nvma1 86249

d1nvmc1 86253

d1nvme1 86257

d1nvmg1 86261

d1rqba1 105057

d1rqha1 105066

d1rr2a1 105073

d1rqea1 105061

d1s3ha1 105235

d1u5ja1 107682

d1t95a1 106705

d1p9qc1 104093

d1ufza_ 107821

d1t3ta1 106381

d1jjcb1 66759

d1jjcb2 66760

d1pysb1 16299

d1pysb2 16300

d2alyb1 126988

d2alyb2 126989

d2amcb1 127003

d2amcb2 127004

d1b7yb1 16301

d1b7yb2 16302

d2akwb1 126938

d2akwb2 126939

d1b70b1 16303

d1b70b2 16304

d2iy5b1 137794

d2iy5b2 137795

d1eiyb1 16305

d1eiyb2 16306

d1xpaa1 16308

d1d4ua1 16307

d1r8da_ 104841

d1r8db_ 104842

d1jbga_ 66480

d1r8ea1 104843

d3d6za1 157414

d3d6ya1 157412

d3d71a1 157418

d3d70a1 157416

d1exja1 16309

d1exia1 16310

d1q06a_ 95493

d1q06b_ 95494

d1q05a_ 95491

d1q05b_ 95492

d1q07a_ 95495

d1q07b_ 95496

d1q08a_ 95497

d1q08b_ 95498

d1q0aa_ 95500

d1q0ab_ 95501

d1q09a_ 95499

d1l8ra_ 73700

d1l8rb_ 73701

d1sbxa_ 105414

d1j9ia_ 71619

d1j9ib_ 71620

d1nd9a_ 85575

d1rh6a_ 104935

d1rh6b_ 104936

d1pm6a_ 94894

d1lx8a_ 84734

d1qpma_ 16230

d1g4da_ 16227

d1tnsa_ 16228

d1tnta_ 16229

d2vz4a_ 168956

d1quua1 16320

d1quua2 16321

d1hcia1 60950

d1hcia2 60951

d1hcia3 60952

d1hcia4 60953

d1hcib1 60954

d1hcib2 60955

d1hcib3 60956

d1hcib4 60957

d1u5pa1 113046

d1u5pa2 113047

d1cuna1 16313

d1cuna2 16314

d1cunb1 16315

d1cunb2 16316

d1cunc1 16317

d1cunc2 16318

d1u4qa1 113024

d1u4qa2 113025

d1u4qa3 113026

d1u4qb1 113027

d1u4qb2 113028

d1u4qb3 113029

d1aj3a_ 16319

d2spca_ 16311

d2spcb_ 16312

d1owaa_ 93640

d1s35a1 98420

d1s35a2 98421

d3f31a_ 175428

d3f31b_ 175429

d2e2aa_ 88499

d2e2ab_ 88500

d2e2ac_ 88501

d1e2aa_ 16322

d1e2ab_ 16323

d1e2ac_ 16324

d3k1sa_ 178955

d3k1sb_ 178956

d3k1sc_ 178957

d3k1sd_ 178958

d3k1se_ 178959

d3k1sf_ 178960

d3k1sg_ 178961

d3k1sh_ 178962

d3k1si_ 178963

d3l8ra_ 180084

d3l8rb_ 180085

d3l8rc_ 180086

d3l8rd_ 180087

d3l8re_ 180088

d3l8rf_ 180089

d3l8rg_ 180090

d3l8rh_ 180091

d1jnra1 66966

d1jnrc1 66970

d1jnza1 71766

d1jnzc1 71770

d1kf6a1 72394

d1kf6m1 72401

d1l0va1 73412

d1l0vm1 73419

d1kfya1 72427

d1kfym1 72434

d2b76a1 128009

d2b76m1 128016

d3cira1 156679

d3cirm1 156686

d2bs2a1 129030

d2bs2d1 129036

d1qlba1 16330

d1qlbd1 16331

d1e7pa1 59347

d1e7pd1 59353

d1e7pg1 59359

d1e7pj1 59365

d1chua1 16325

d1knra1 72788

d1knpa1 72783

d1neka1 80426

d1nena1 80433

d1g73a_ 16332

d1g73b_ 16333

d1fewa_ 16334

d1qsda_ 16335

d1qsdb_ 16336

d1h7ca_ 70914

d2qant1 150286

d2qalt1 150232

d2qbft1 150506

d2qbdt1 150452

d2qoyt1 151109

d2qp0t1 151162

d3df1t1 157622

d3df3t1 157676

d2avyt1 144861

d2aw7t1 144904

d1vs5t1 144454

d1vs7t1 144495

d2qb9t1 150346

d2qbbt1 150399

d2i2pt1 145439

d2i2ut1 145481

d2qout1 151003

d2qowt1 151056

d2qbht1 150560

d2qbjt1 150614

d2vhot1 153142

d2z4kt1 154070

d2z4mt1 154124

d2vhpt1 153164

d2gy9t1 145239

d2gybt1 145261

d2uubt1 139951

d2vqet1 153444

d2vqft1 153463

d2uuat1 139931

d1j5et_ 71563

d1fjgt_ 16338

d2uuct1 139971

d2uxct1 140023

d1xmqt_ 115549

d2uu9t1 139911

d1n32t_ 79889

d1xnqt_ 115623

d1xnrt_ 115645

d1hr0t_ 16339

d1hnzt_ 16340

d2j02t1 137885

d2j00t1 137858

d1xmot_ 115519

d1i94t_ 62011

d2uxdt1 152300

d1hnwt_ 16341

d1hnxt_ 16342

d3d5ct1 157383

d3d5at1 157353

d2e5lt1 132044

d1n33t_ 79911

d2hhht1 136496

d2uxbt1 152281

d1n34t_ 79933

d2v48t1 152538

d1i96t_ 62055

d2v46t1 152502

d2f4vt1 132957

d1n36t_ 79956

d1i97t_ 62078

d1i95t_ 62033

d2hgpw1 136439

d2hgiw1 136418

d2hgrw1 136460

d2qnhu1 150944

d2ow8u1 139418

d1yl4w1 123616

d2b9ot1 128182

d2b64t1 127952

d2b9mt1 128145

d3ldqb_ 180199

d1hx1b_ 61350

d3fzhb_ 176211

d3m3zb_ 180810

d3fzkb_ 176212

d3fzfb_ 176210

d3fzlb_ 176213

d3fzmb_ 176214

d1i6za_ 61861

d1t7sa_ 106636

d1t7sb_ 106637

d1uk5a_ 99487

d1ugoa_ 107830

d1m62a_ 74520

d1m7ka_ 74573

d1j2jb_ 84017

d1o3xa_ 86617

d1x79a_ 114924

d1nwmx_ 86301

d1oxza_ 87542

d1nafa_ 85487

d1wr6a1 144561

d1yd8g1 144631

d1yd8h_ 144632

d1wrda1 144565

d1hx8a1 90362

d1hx8b1 90364

d1hf8a1 90354

d1hg5a1 90360

d1hg2a1 90358

d1hfaa1 90356

d1rrza_ 105086

d1z8ua_ 124724

d1z8uc_ 124726

d3ovua_ 196163

d1y01a_ 116276

d1w09a_ 108983

d1w0aa_ 108984

d1xzya_ 116275

d1w0ba_ 108985

d1vcta1 119986

d2bkoa1 128702

d2bkpa1 128704

d2bkna1 128700

d1t72a_ 112284

d1t72b_ 112285

d1t72d_ 112286

d1t72e_ 112287

d1t72f_ 112288

d1t72g_ 112289

d1t8ba_ 112314

d1t8bb_ 112315

d1xwma_ 116132

d1sumb_ 106025

d1vp7a_ 113941

d1vp7b_ 113942

d1vp7c_ 113943

d1vp7d_ 113944

d1vp7e_ 113945

d1vp7f_ 113946

d1wfda_ 114578

d1yxra1 124200

d2jq9a1 148173

d2jqha1 148177

d1wr0a1 121183

d2jqka1 148179

d2cpta1 130706

d2v6xa_ 168362

d1xdpa1 121896

d1xdpb1 121900

d1xdoa1 121888

d1xdob1 121892

d2o8ra1 138933

d2o8rb1 138937

d2crba1 130737

d2goma1 147149

d2gomb_ 147150

d2goxb1 147151

d2goxd_ 147152

d3d5rc1 157405

d3d5rd_ 157406

d3d5sc1 157409

d3d5sd_ 157410

d2nojb1 148325

d1lp1a_ 84664

d1lp1b_ 84665

d1deeg_ 16343

d1deeh_ 16344

d1fc2c_ 16345

d2jwda1 148228

d1edia_ 16349

d1edka_ 16348

d1edla_ 16347

d1edja_ 16346

d1h0ta_ 83440

d1h0tb_ 83441

d2spza_ 16352

d1q2na_ 95631

d1ss1a_ 98977

d1bdca_ 16350

d1bdda_ 16351

d3qwoc_ 184677

d3qwop_ 184678

d1gjta_ 60579

d1gjsa_ 60578

d1tf0b_ 106825

d1gaba_ 16353

d1prba_ 16354

d2j5ya_ 147887

d2j5yb_ 147888

d2vdbb_ 152972

d1gvna_ 76353

d1gvnc_ 76355

d3q8xa_ 184274

d3q8xc_ 184276

d3bvua1 155667

d3bvxa1 155676

d2ow6a1 149039

d3ddfa1 157547

d3bvwa1 155673

d3buia1 155607

d1tqwa1 119324

d1qwna1 96487

d3bvva1 155670

d2f7pa1 133095

d1tqsa1 119312

d3blba1 155390

d2f18a1 132719

d1qx1a1 96512

d3bvta1 155664

d2f7ra1 133101

d3czsa1 157156

d3d4za1 157313

d3buba1 155601

d3czna1 157151

d1htya1 83064

d2f1aa1 132722

d2f1ba1 132725

d3d51a1 157319

d2f7oa1 133092

d3d4ya1 157310

d1hxka1 83070

d3cv5a1 156999

d3ddga1 157550

d1r33a1 111667

d3d50a1 157316

d2ow7a1 149042

d3d52a1 157322

d2alwa1 126983

d2f7qa1 133098

d3buda1 155604

d1tqta1 119315

d1r34a1 111670

d2fyva1 134405

d1tqua1 119318

d1ps3a1 95065

d1hwwa1 83067

d1qwua1 96503

d3buqa1 155638

d3bupa1 155635

d1tqva1 119321

d1o7d.1 86639

d1k1xa1 84279

d1k1xb1 84282

d1k1ya1 84285

d1k1yb1 84288

d1k1wa1 84276

d2b5dx1 127886

d1ufaa1 99329

d1u00a1 112900

d1dkza1 90345

d1dkxa1 90339

d1dkya1 90341

d1dkyb1 90343

d1ud0a_ 99198

d1ud0b_ 99199

d1ud0c_ 99200

d1ud0d_ 99201

d1oksa_ 93271

d1t6oa_ 106578

d1r4ga_ 96996

d1nu9c1 92200

d1nu9c2 92201

d1nu9f1 92203

d1nu9f2 92204

d1nu7d1 92191

d1nu7d2 92192

d1nu7h1 92193

d1nu7h2 92194

d1r8ia_ 97239

d2qkwa1 150848

d1r5ea_ 111701

d2ahma1 126760

d1ysya1 123986

d2ahmb_ 126761

d2ahmc_ 126762

d2ahmd_ 126763

d3ub0c_ 195994

d2gf4a1 135074

d2gf4b_ 161430

d2oo2a1 148917

d2pmra1 149670

d1ebdc_ 16355

d1w4ha1 120626

d1bbla_ 16356

d2cyua1 131027

d1bala_ 16357

d1w85i_ 114347

d1w85j_ 114348

d1w88i_ 114361

d1w88j_ 114362

d1w3da_ 109151

d2pdda_ 16358

d2pdea_ 16359

d3rnme_ 185064

d3rnmf_ 185065

d2erla_ 16360

d1erca_ 16361

d1erpa_ 16363

d1erya_ 16364

d1erda_ 16362

d1hd6a_ 16365

d1x9ba_ 114982

d1hb6a_ 70959

d1hb8a_ 70960

d1hb8b_ 70961

d1hb8c_ 70962

d1nvla_ 92208

d1ntia_ 103872

d1acaa_ 16367

d2abda_ 16366

d1hbka_ 60887

d2cb8a_ 163356

d2cb8b_ 163357

d2fj9a_ 164403

d3epya_ 175152

d3epyb_ 175153

d2wh5a_ 169341

d2wh5b_ 169342

d2wh5c_ 169343

d2wh5d_ 169344

d2wh5e_ 169345

d2wh5f_ 169346

d3fp5a_ 175949

d1gg3a1 16372

d1gg3b1 16373

d1gg3c1 16374

d1ni2a1 80525

d1ni2b1 80528

d2al6a1 126966

d2al6b1 126969

d2aeha1 126625

d2aehb1 126628

d2j0ma1 147845

d1h4ra1 65616

d1h4rb1 65619

d1isna1 71400

d1ef1a1 16368

d1ef1b1 16369

d1e5wa1 59280

d1sgha1 105529

d2zpya1 154758

d1j19a1 83958

d2d10a1 131097

d2d10b1 131100

d2d10c1 131103

d2d10d1 131106

d1gc7a1 16370

d2d11a1 131109

d2d11b1 131112

d2d11c1 131115

d2d11d1 131118

d2d2qa1 131182

d2d2qb1 131185

d1gc6a1 16371

d2emta1 146928

d2emtb1 146931

d2emsa1 146925

d2yvca1 140078

d2yvcb1 140081

d2yvcc1 140084

d1mixa1 79166

d2hrja1 147376

d1mizb1 79172

d1mk7b1 79219

d1mk7d1 79221

d1mk9b1 79223

d1mk9d1 79225

d1mk9f1 79227

d1mk9h1 79229

d1y19b1 116329

d1y19d1 116331

d1y19f1 116333

d1y19h1 116335

d1y19j1 116337

d1y19l1 116339

d4i9oa_ 196858

d1sb0a_ 98789

d1kdxa_ 16375

d2aghb1 126725

d2fcwa1 133288

d1nrea_ 16377

d1lrea_ 16376

d2ftua1 134082

d1op1a_ 93396

d1ov2a_ 93580

d2fyla1 134378

d1ujsa_ 99467

d1unda_ 107966

d1qzpa_ 96654

d1yu5x_ 124039

d2rjya_ 152105

d2rjva_ 152104

d1yu8x_ 124044

d1yu7x_ 162302

d3nkja_ 182343

d2rjwa_ 168153

d2rjwb_ 168154

d3myca_ 181695

d3myex_ 181696

d2rjxa_ 168155

d2rjxb_ 168156

d3myaa_ 181693

d3myab_ 181694

d1viia_ 16378

d1qqva_ 16379

d1unca_ 107965

d1tbaa_ 16380

d1aila_ 16381

d1ns1a_ 16382

d1ns1b_ 16383

d1xeqa1 121915

d1xeqb_ 161361

d2zkoa_ 171289

d2zkob_ 171290

d2z0aa_ 170948

d2z0ab_ 170949

d2z0ac_ 170950

d2z0ad_ 170951

d3m8aa_ 180945

d3m8ab_ 180946

d3m8ac_ 180947

d3m8ad_ 180948

d3m8ae_ 180949

d3m8af_ 180950

d3m8ag_ 180951

d3m8ah_ 180952

d3m8ai_ 180953

d3m8aj_ 180954

d3m8ak_ 180955

d3m8al_ 180956

d3rt3c_ 193691

d1a32a_ 16384

d3df1o1 157617

d3df3o1 157671

d2avyo1 144856

d2aw7o1 144899

d1vs5o1 144449

d1vs7o1 144490

d2i2po1 145434

d2i2uo1 145476

d2vhoo1 153137

d2vhpo1 153159

d1dk1a_ 16385

d1f7ya_ 16386

d1kuqa_ 84470

d2uubo1 139946

d2uuao1 139926

d1j5eo_ 71558

d1fjgo_ 16388

d2uuco1 139966

d2uxco1 140018

d1xmqo_ 115544

d2uu9o1 139906

d1n32o_ 79884

d1xnqo_ 115618

d1xnro_ 115640

d1hr0o_ 16389

d1hnzo_ 16390

d2j02o1 137880

d2j00o1 137853

d1xmoo_ 115514

d1i94o_ 62006

d1hnwo_ 16391

d1hnxo_ 16392

d2e5lo1 132039

d1n33o_ 79906

d2hhho1 136491

d1n34o_ 79928

d2fkxa1 133685

d1i96o_ 62050

d2f4vo1 132952

d1n36o_ 79951

d1ab3a_ 16395

d1i97o_ 62073

d1i95o_ 62028

d2hgpr1 136434

d2hgir1 136413

d2hgrr1 136455

d2ow8p1 139413

d1yl4r1 123611

d2b9oo1 128177

d2b64o1 127947

d2b9mo1 128140

d1g1xb_ 16393

d1g1xg_ 16394

d1r1ba_ 16396

d1d2da_ 16397

d1fyja_ 60115

d1r6ta1 97160

d2hp8a_ 16398

d1hp8a_ 16399

d1u97a_ 113233

d1u96a_ 113232

d2enda_ 16400

d1enja_ 16401

d1enka_ 16402

d1enia_ 16403

d2fcca_ 133264

d2fccb_ 133265

d1vasa_ 16404

d3lyna_ 16413

d3lynb_ 16414

d2lisa_ 16405

d1lisa_ 16406

d2lyna_ 16407

d2lynb_ 16408

d2lync_ 16409

d2lynd_ 16410

d1lyna_ 16411

d1lynb_ 16412

d1gaka_ 16415

d2wkxa1 169423

d2bh7a1 128516

d1lbua1 16416

d1su3a1 112117

d1su3b1 112120

d1eaka1 70093

d1eakb1 70098

d1eakc1 70103

d1eakd1 70108

d1ck7a6 58969

d1gxda1 70691

d1gxdb1 70697

d1l6ja1 73619

d1slma1 59042

d2gzka1 135907

d1hsma_ 16421

d1nhma_ 16422

d1nhna_ 16423

d1hsna_ 16424

d1ckta_ 16417

d1hmea_ 16418

d1hmfa_ 16419

d1aaba_ 16420

d1j3da_ 103833

d1j3xa_ 103840

d1j3ca_ 103832

d1qrva_ 16425

d1qrvb_ 16426

d1e7ja_ 59344

d1hmaa_ 16427

d2lefa_ 16433

d1lwma_ 78274

d1cg7a_ 16428

d1j5na_ 77083

d1k99a_ 68341

d1l8ya_ 73708

d1l8za_ 73709

d1v64a_ 108392

d1wgfa_ 114611

d1v63a_ 108391

d1o4xb_ 92472

d1gt0d_ 76337

d1i11a_ 16431

d1j46a_ 66372

d1j47a_ 66373

d2gzka2 135908

d1hrya_ 16429

d1hrza_ 16430

d3nm9a_ 182390

d3nm9d_ 182391

d3nm9g_ 182392

d3nm9j_ 182393

d3nm9m_ 182394

d3nm9p_ 182395

d2hdza_ 165062

d3u2bc_ 186063

d3f27d_ 175425

d4a3na_ 186718

d1kx5c_ 77595

d1kx5g_ 77599

d1s32c_ 98414

d1s32g_ 98418

d1kx3c_ 77579

d1kx3g_ 77583

d3c1bc_ 155853

d3c1bg_ 155857

d1m19c_ 78381

d1m19g_ 78385

d1p3ic_ 94016

d1p3ig_ 94020

d3mgrc_ 181202

d3mgrg_ 181204

d1m18c_ 78373

d1m18g_ 78377

d1p3lc_ 94034

d1p3lg_ 94038

d1p3pc_ 94059

d1p3pg_ 94063

d3mgpc_ 181194

d3mgpg_ 181196

d1p3oc_ 94051

d1p3og_ 94055

d3mnnc_ 181452

d3mnng_ 181454

d1p3gc_ 94008

d1p3gg_ 94012

d1p34c_ 93972

d1p34g_ 93976

d1m1ac_ 78389

d1m1ag_ 78393

d2nzdc_ 138850

d2nzdg_ 138853

d1kx4c_ 77587

d1kx4g_ 77591

d3mgqc_ 181198

d3mgqg_ 181200

d1p3mc_ 94042

d1p3mg_ 94046

d1p3kc_ 94026

d1p3kg_ 94030

d1aoic_ 16440

d1aoig_ 16441

d1p3ac_ 93982

d1p3ag_ 93986

d3lz0c_ 180644

d3lz0g_ 180646

d3lz1c_ 180648

d3lz1g_ 180650

d1zlac1 125242

d1zlag_ 125246

d1p3fc_ 94000

d1p3fg_ 94004

d1p3bc_ 93990

d1p3bg_ 93994

d3kuyc_ 179729

d3kuyg_ 179731

d3lelc_ 191755

d3lelg_ 191758

d3lelm_ 191761

d3lelq_ 191764

d3c1cc1 155860

d3c1cg1 155863

d3b6fc1 154886

d3b6fg1 154890

d3b6gc1 154894

d3b6gg1 154898

d1id3c_ 66114

d1id3g_ 66118

d2jssa1 148192

d1tzya_ 107530

d1tzye_ 107534

d2aroa_ 127209

d2aroe_ 127213

d1hq3a_ 16434

d1hq3e_ 16435

d1eqza_ 16436

d1eqze_ 16437

d2hioa_ 16438

d1hioa_ 16439

d2cv5c1 130849

d2cv5g_ 130853

d1f66c_ 16442

d1f66g_ 16443

d2f8nk1 145150

d1kx5d_ 77596

d1kx5h_ 77600

d1s32d_ 98415

d1s32h_ 98419

d1kx3d_ 77580

d1kx3h_ 77584

d1m19d_ 78382

d1m19h_ 78386

d1p3id_ 94017

d1p3ih_ 94021

d3mgrd_ 191782

d3mgrh_ 191784

d1m18d_ 78374

d1m18h_ 78378

d1p3ld_ 94035

d1p3lh_ 94039

d1f66d_ 16450

d1f66h_ 16451

d3mvdd_ 191790

d3mvdh_ 191792

d1p3pd_ 94060

d1p3ph_ 94064

d3mgpd_ 191774

d3mgph_ 191776

d3ljad_ 191767

d3ljah_ 191769

d1p3od_ 94052

d1p3oh_ 94056

d3mnnd_ 191786

d3mnnh_ 191788

d1p3gd_ 94009

d1p3gh_ 94013

d1p34d_ 93973

d1p34h_ 93977

d1m1ad_ 78390

d1m1ah_ 78394

d2nzdd_ 191677

d2nzdh_ 191678

d1kx4d_ 77588

d1kx4h_ 77592

d3mgqd_ 191778

d3mgqh_ 191780

d1p3md_ 94043

d1p3mh_ 94047

d1p3kd_ 94027

d1p3kh_ 94031

d1aoid_ 16452

d1aoih_ 16453

d1p3ad_ 93983

d1p3ah_ 93987

d1zlad_ 125243

d1zlah_ 125247

d2f8nh_ 133139

d1p3fd_ 94001

d1p3fh_ 94005

d1p3bd_ 93991

d1p3bh_ 93995

d3kuyd_ 191750

d3kuyh_ 191752

d3leld_ 191756

d3lelh_ 191759

d3leln_ 191762

d3lelr_ 191765

d2fj7d1 133550

d2fj7h1 133553

d3b6fd1 154887

d3b6fh1 154891

d3b6gd1 154895

d3b6gh1 154899

d1zbbd1 145987

d1id3d_ 66115

d1id3h_ 66119

d2jssa2 148193

d1tzyb_ 107531

d1tzyf_ 107535

d2arob_ 127210

d2arof_ 127214

d1hq3b_ 16444

d1hq3f_ 16445

d1eqzb_ 16446

d1eqzf_ 16447

d2hiob_ 16448

d1hiob_ 16449

d2cv5d1 130850

d2cv5h_ 130854

d2f8nd_ 133135

d1u35d1 119496

d1u35h1 119500

d3c1bd_ 155854

d3c1bh_ 155858

d3c1cd1 155861

d3c1ch1 155864

d1kx5a_ 77593

d1kx5e_ 77597

d1s32a_ 98412

d1s32e_ 98416

d1kx3a_ 77577

d1kx3e_ 77581

d3c1ba_ 155851

d3c1be_ 155855

d1m19a_ 78379

d1m19e_ 78383

d1p3ia_ 94014

d1p3ie_ 94018

d3mgra_ 181201

d3mgre_ 181203

d1m18a_ 78371

d1m18e_ 78375

d1p3la_ 94032

d1p3le_ 94036

d1f66a_ 16460

d1f66e_ 16461

d3mvda_ 181592

d3mvde_ 181593

d1p3pa_ 94057

d1p3pe_ 94061

d3mgpa_ 181193

d3mgpe_ 181195

d3ljaa_ 180318

d3ljae_ 180319

d1p3oa_ 94049

d1p3oe_ 94053

d3mnna_ 181451

d3mnne_ 181453

d1p3ga_ 94006

d1p3ge_ 94010

d1p34a_ 93970

d1p34e_ 93974

d1m1aa_ 78387

d1m1ae_ 78391

d2nzda_ 138848

d2nzde_ 138851

d1kx4a_ 77585

d1kx4e_ 77589

d3mgqa_ 181197

d3mgqe_ 181199

d1p3ma_ 94040

d1p3me_ 94044

d1p3ka_ 94024

d1p3ke_ 94028

d1aoia_ 16462

d1aoie_ 16463

d2io5b_ 161465

d1p3aa_ 93980

d1p3ae_ 93984

d3lz0a_ 180643

d3lz0e_ 180645

d3lz1a_ 180647

d3lz1e_ 180649

d1zlaa_ 125240

d1zlae_ 125244

d2f8na_ 133133

d2f8ne_ 133136

d1p3fa_ 93998

d1p3fe_ 94002

d1p3ba_ 93988

d1p3be_ 93992

d3kuya_ 179728

d3kuye_ 179730

d3lela_ 180229

d3lele_ 180230

d3lelk_ 180231

d3lelo_ 180232

d2fj7a1 133548

d2fj7e1 133551

d3b6fa1 154884

d3b6fe1 154888

d3b6ga1 154892

d3b6ge1 154896

d1zbba1 145985

d1zbbe1 145988

d1id3a_ 66112

d1id3e_ 66116

d1tzyc_ 107532

d1tzyg_ 107536

d2aroc_ 127211

d2arog_ 127215

d1hq3c_ 16454

d1hq3g_ 16455

d1eqzc_ 16456

d1eqzg_ 16457

d2hioc_ 16458

d1hioc_ 16459

d2pyoa_ 149949

d2pyoe_ 149951

d2cv5a_ 130847

d2cv5e_ 130851

d3afaa_ 172034

d3afae_ 172035

d3av1a_ 172344

d3av1e_ 172345

d3av2a_ 172346

d3av2e_ 172347

d3a6na_ 171819

d3a6ne_ 171820

d1u35a1 119493

d1u35e1 119497

d2huec_ 136776

d1kx5b_ 77594

d1kx5f_ 77598

d1s32b_ 98413

d1s32f_ 98417

d1kx3b_ 77578

d1kx3f_ 77582

d3c1bb_ 155852

d3c1bf_ 155856

d1m19b_ 78380

d1m19f_ 78384

d1p3ib_ 94015

d1p3if_ 94019

d3mgrb_ 191781

d3mgrf_ 191783

d1m18b_ 78372

d1m18f_ 78376

d1p3lb_ 94033

d1p3lf_ 94037

d3mvdb_ 191789

d3mvdf_ 191791

d1p3pb_ 94058

d1p3pf_ 94062

d3mgpb_ 191773

d3mgpf_ 191775

d3ljab_ 191766

d3ljaf_ 191768

d1p3ob_ 94050

d1p3of_ 94054

d3mnnb_ 191785

d3mnnf_ 191787

d1p3gb_ 94007

d1p3gf_ 94011

d1p34b_ 93971

d1p34f_ 93975

d1m1ab_ 78388

d1m1af_ 78392

d2nzdb_ 138849

d2nzdf_ 138852

d1kx4b_ 77586

d1kx4f_ 77590

d3mgqb_ 191777

d3mgqf_ 191779

d1p3mb_ 94041

d1p3mf_ 94045

d1p3kb_ 94025

d1p3kf_ 94029

d1aoib_ 16470

d1aoif_ 16471

d2io5c_ 137542

d1p3ab_ 93981

d1p3af_ 93985

d1zlab_ 125241

d1zlaf_ 125245

d2f8nb_ 133134

d2f8nf_ 133137

d1p3fb_ 93999

d1p3ff_ 94003

d1p3bb_ 93989

d1p3bf_ 93993

d3kuyb_ 191749

d3kuyf_ 191751

d3lelb_ 191754

d3lelf_ 191757

d3lell_ 191760

d3lelp_ 191763

d3c1cb1 155859

d3c1cf1 155862

d2fj7b1 133549

d2fj7f1 133552

d3b6fb1 154885

d3b6ff1 154889

d3b6gb1 154893

d3b6gf1 154897

d1zbbb1 145986

d1zbbf1 145989

d1id3b_ 66113

d1id3f_ 66117

d1tzyd_ 107533

d1tzyh_ 107537

d2arod_ 127212

d2aroh_ 127216

d1hq3d_ 16464

d1hq3h_ 16465

d1eqzd_ 16466

d1eqzh_ 16467

d2hiod_ 16468

d1hiod_ 16469

d2nqba_ 148340

d2nqbb_ 148341

d2nqbe_ 148342

d2nqbf_ 148343

d2pyob_ 149950

d2pyof_ 149952

d4h9nb_ 192497

d4h9qb_ 192511

d3nqjb_ 182485

d4h9ob_ 192498

d4h9pb_ 192510

d4h9rb_ 192509

d3r45b_ 191959

d4h9sc_ 194488

d4h9sd_ 194489

d2cv5b_ 130848

d2cv5f_ 130852

d3nqub_ 191902

d4hgac_ 192531

d2yfvb_ 170781

d1f66b_ 16472

d1f66f_ 16473

d1u35b1 119494

d1u35f1 119498

d2f8ng_ 133138

d1u35c1 119495

d1u35g1 119499

d4h9qa_ 194487

d4h9oa_ 194548

d4h9pa_ 194490

d4h9ra_ 194491

d4h9sa_ 193447

d3azga_ 196166

d3azle_ 193637

d3azfa_ 196167

d4hgab_ 194356

d3aywa_ 196168

d3azja_ 193636

d3azma_ 193638

d1f1ea_ 64927

d1b67a_ 16474

d1b67b_ 16475

d1htaa_ 16476

d1a7wa_ 16477

d1b6wa_ 16478

d1bfma_ 16479

d1bfmb_ 16480

d1ku5a_ 91035

d1ku5b_ 91036

d2bykb1 129487

d2byka1 129486

d1q9ca_ 96258

d1q9cb_ 96259

d1q9cc_ 96260

d1q9cd_ 96261

d1q9ce_ 96262

d1q9cf_ 96263

d1q9cg_ 96264

d1q9ch_ 96265

d1q9ci_ 96266

d1jfia_ 62936

d1jfib_ 62937

d1n1ja_ 79813

d1n1jb_ 79814

d1h3oa_ 76644

d1h3oc_ 76646

d1h3ob_ 76645

d1h3od_ 76647

d1bh9a_ 16483

d1bh8a_ 16484

d1bh9b_ 16485

d1bh8b_ 16486

d1tafa_ 16481

d1tafb_ 16482

d4g92b_ 194415

d1r4va_ 97049

d1wwia1 121362

d1wwsa_ 121370

d1wwsb_ 121371

d1wwsc_ 121372

d1wwsd_ 121373

d1wwse_ 121374

d1wwsf_ 121375

d1wwsg_ 121376

d1wwsh_ 121377

d1fpoa2 16487

d1fpob2 16488

d1fpoc2 16489

d1eexg_ 16490

d1eexm_ 16491

d1egvg_ 16492

d1egvm_ 16493

d1uc4g_ 88453

d1uc4m_ 88455

d1iwbg_ 83753

d1iwbm_ 83755

d1egmg_ 16494

d1egmm_ 16495

d1diog_ 16496

d1diom_ 16497

d1uc5g_ 88459

d1uc5m_ 88461

d1iwpg_ 76887

d1iwpm_ 76889

d1mmfg_ 85021

d1mmfm_ 85023

d1mtyg_ 16498

d1mtyh_ 16499

d1xvbe_ 122348

d1xvbf_ 122349

d1xvge_ 122378

d1xvgf_ 122379

d1fz1e_ 16500

d1fz1f_ 16501

d1xu5e_ 122330

d1xu5f_ 122331

d1fz3e_ 16502

d1fz3f_ 16503

d1xvfe_ 122372

d1xvff_ 122373

d1fz7e_ 16504

d1fz7f_ 16505

d1xvce_ 122354

d1xvcf_ 122355

d1fz0e_ 16506

d1fz0f_ 16507

d1fyze_ 16508

d1fyzf_ 16509

d1fz2e_ 16510

d1fz2f_ 16511

d1fz8e_ 60126

d1fz8f_ 60127

d1fz6e_ 60120

d1fz6f_ 60121

d1mmog_ 16512

d1mmoh_ 16513

d1xmge_ 122157

d1xmgf_ 122158

d1xvde_ 122360

d1xvdf_ 122361

d1xmhe_ 122163

d1xmhf_ 122164

d1xu3e_ 122324

d1xu3f_ 122325

d1fz9e_ 60132

d1fz9f_ 60133

d1xvee_ 122366

d1xvef_ 122367

d1xmfe_ 122151

d1xmff_ 122152

d1fz5e_ 16516

d1fz5f_ 16517

d1fz4e_ 16514

d1fz4f_ 16515

d1fzhe_ 60138

d1fzhf_ 60139

d4gamr_ 194029

d1fzie_ 60144

d1fzif_ 60145

d1mhyg_ 16518

d1mhzg_ 16519

d1om2a_ 16520

d1jw2a_ 67372

d4icgc_ 196794

d4icgd_ 196793

d2gboa1 134923

d2gbob_ 134924

d2odma_ 166650

d2odmb_ 166651

d2gsva1 147174

d2gsvb_ 147175

d2js1a1 148189

d2js1b1 148190

d1nfoa_ 16526

d1le2a_ 16527

d1nfna_ 16523

d1h7ia_ 60725

d1ea8a_ 59400

d1lpea_ 16524

d1bz4a_ 16521

d1or3a_ 16522

d1or2a_ 16525

d1gs9a_ 83313

d1b68a_ 59076

d1le4a_ 16528

d2asra_ 16529

d2liga_ 16530

d2ligb_ 16531

d1vlsa_ 16532

d1liha_ 16535

d1jmwa_ 63181

d1vlta_ 16533

d1vltb_ 16534

d1wasa_ 16536

d1wata_ 16537

d1watb_ 16538

d256ba_ 16539

d256bb_ 16540

d1m6ta_ 78705

d1lm3b_ 74027

d1lm3d_ 74028

d1qq3a_ 16541

d1qpua_ 16542

d1apca_ 16543

d3de8a_ 173861

d3de8b_ 173862

d3de8c_ 173863

d3de8d_ 173864

d3c63a_ 173056

d3c63b_ 173057

d3c63c_ 173058

d3c63d_ 173059

d3qw0a_ 184655

d3qw0b_ 184656

d3qw0c_ 184657

d3qw0d_ 184658

d3m4ca_ 180824

d3m4cb_ 180825

d3m4cc_ 180826

d3m4cd_ 180827

d3tolb_ 194998

d3tolc_ 194997

d3c62a_ 173052

d3c62b_ 173053

d3c62c_ 173054

d3c62d_ 173055

d3nmia_ 182397

d3nmib_ 182398

d3nmic_ 182399

d3nmid_ 182400

d3nmie_ 182401

d3nmif_ 182402

d3hnka_ 177718

d3hnkb_ 177719

d3m79a_ 180901

d3m79b_ 180902

d3m79c_ 180903

d3m79d_ 180904

d3m79e_ 180905

d3m79f_ 180906

d3m79g_ 180907

d3m79h_ 180908

d3hnja_ 177714

d3hnjb_ 177715

d3hnjc_ 177716

d3hnjd_ 177717

d3de9a_ 173865

d3hnla_ 177720

d3hnlb_ 177721

d3iq5a_ 178521

d3iq5b_ 178522

d3iq5c_ 178523

d3iq5d_ 178524

d2bc5a_ 163036

d2bc5b_ 163037

d2bc5c_ 163038

d2bc5d_ 163039

d3hnia_ 177706

d3hnib_ 177707

d3hnic_ 177708

d3hnid_ 177709

d3hnie_ 177710

d3hnif_ 177711

d3hnig_ 177712

d3hnih_ 177713

d3m4ba_ 180820

d3m4bb_ 180821

d3m4bc_ 180822

d3m4bd_ 180823

d3fooa_ 175935

d3foob_ 175936

d3fooc_ 175937

d3food_ 175938

d3fooe_ 175939

d3foof_ 175940

d3foog_ 175941

d3fooh_ 175942

d3fooi_ 175943

d3fooj_ 175944

d3fook_ 175945

d3fool_ 175946

d3qvya_ 184647

d3qvyb_ 184648

d3qvyc_ 184649

d3qvyd_ 184650

d3iq6a_ 178525

d3iq6b_ 178526

d3iq6c_ 178527

d3iq6d_ 178528

d3iq6e_ 178529

d3iq6f_ 178530

d3iq6g_ 178531

d3iq6h_ 178532

d3l1ma_ 179857

d3nmka_ 182403

d3nmkb_ 182404

d3nmkc_ 182405

d3nmkd_ 182406

d3m15a_ 180706

d3m15b_ 180707

d3m15c_ 180708

d3qvza_ 184651

d3qvzb_ 184652

d3qvzc_ 184653

d3qvzd_ 184654

d2qlaa_ 167702

d2qlab_ 167703

d2qlac_ 167704

d2qlad_ 167705

d3qw1a_ 184659

d3qw1b_ 184660

d3qw1c_ 184661

d3qw1d_ 184662

d1cgna_ 16548

d1e85a_ 16549

d1cgoa_ 16550

d1e86a_ 16551

d1e84a_ 16552

d1e83a_ 16553

d1bbha_ 16546

d1bbhb_ 16547

d1cpqa_ 16556

d1rcpa_ 16557

d1rcpb_ 16558

d1cpra_ 16559

d1nbba_ 16560

d1nbbb_ 16561

d1gqaa_ 76275

d1gqad_ 76276

d1jafa_ 16554

d1jafb_ 16555

d1mqva_ 79415

d1mqvb_ 79416

d1a7va_ 16562

d1a7vb_ 16563

d2ccya_ 16544

d2ccyb_ 16545

d1s05a_ 98254

d2ykza_ 170841

d3zqva_ 186519

d2yl7a_ 170845

d3ztma_ 186565

d2yl0a_ 170842

d2yl1a_ 170843

d2yl3a_ 170844

d3zqya_ 186521

d2ylga_ 170854

d2xl6a_ 170194

d3ztza_ 186572

d2xm4a_ 170222

d2xlha_ 170216

d2xlea_ 170215

d2xl8a_ 170195

d2xlwa_ 170220

d2xlma_ 170217

d3zwia_ 192215

d2ylda_ 170853

d2xloa_ 170218

d2xlva_ 170219

d2xm0a_ 170221

d2ylia_ 191840

d2xlda_ 170214

d2j9ba_ 147935

d2j9bb_ 147936

d2j8wa_ 147931

d2j8wb_ 147932

d3vrca_ 194694

d3vrcb_ 194693

d1i4ya_ 61738

d1i4yb_ 61739

d1i4yc_ 61740

d1i4yd_ 61741

d1i4ye_ 61742

d1i4yf_ 61743

d1i4yg_ 61744

d1i4yh_ 61745

d1i4za_ 61746

d1i4zb_ 61747

d1i4zc_ 61748

d1i4zd_ 61749

d1i4ze_ 61750

d1i4zf_ 61751

d1i4zg_ 61752

d1i4zh_ 61753

d1hrba_ 16580

d1hrbb_ 118511

d2hmza_ 16564

d2hmzb_ 16565

d2hmzc_ 16566

d2hmzd_ 16567

d2hmqa_ 16568

d2hmqb_ 16569

d2hmqc_ 16570

d2hmqd_ 16571

d1hmda_ 16572

d1hmdb_ 16573

d1hmdc_ 16574

d1hmdd_ 16575

d1hmoa_ 16576

d1hmob_ 16577

d1hmoc_ 16578

d1hmod_ 16579

d2mhra_ 16581

d1a7da_ 16582

d1a7ea_ 16583

d1cgme_ 16588

d1rmva_ 16589

d1ei7a_ 16584

d1ei7b_ 16585

d2tmvp_ 16586

d1vtmp_ 16587

d2om3a1 148865

d3j06a_ 178682

d3fapb_ 16612

d1nsgb_ 16613

d2fapb_ 16614

d4fapb_ 16617

d1auea_ 16615

d1aueb_ 16616

d1fapb_ 16618

d2gaqa1 134893

d4drhe_ 193177

d1ya7o1 144608

d1avo.1 16619

d1avo.2 16620

d1avo.3 16621

d1avo.4 16622

d1avo.5 16623

d1avo.6 16624

d1avo.7 16625

d1yaro_ 144615

d1yarp_ 144616

d1yarq_ 144617

d1yarr_ 144618

d1yars_ 144619

d1yart_ 144620

d1yaru_ 144621

d1ya7p_ 144609

d1ya7q_ 144610

d1ya7r_ 144611

d1ya7s_ 144612

d1ya7t_ 144613

d1ya7u_ 144614

d1yauo_ 144622

d1yaup_ 144623

d1yauq_ 144624

d1yaur_ 144625

d1yaus_ 144626

d1yaut_ 144627

d1yauu_ 144628

d3jrmo_ 178738

d3jrmp_ 178739

d3jrmq_ 178740

d3jrmr_ 178741

d3jrms_ 178742

d3jrmt_ 178743

d3jrmu_ 178744

d3jseo_ 178772

d3jsep_ 178773

d3jseq_ 178774

d3jser_ 178775

d3jses_ 178776

d3jset_ 178777

d3jseu_ 178778

d1h6ga1 60669

d1h6ga2 60670

d1h6gb1 60671

d1h6gb2 60672

d1l7ca1 73647

d1l7ca2 73648

d1l7cb1 73649

d1l7cb2 73650

d1l7cc1 73651

d1l7cc2 73652

d1dova_ 16627

d1dowa_ 16626

d1qkra_ 16628

d1qkrb_ 16629

d1t01a1 106188

d1t01a2 106189

d1st6a1 106000

d1st6a2 106001

d1st6a3 106002

d1st6a4 106003

d1st6a5 106004

d1st6a6 106005

d1st6a7 106006

d1st6a8 106007

d1syqa1 106124

d1syqa2 106125

d1rkea1 97608

d1rkea2 97609

d1rkeb_ 97610

d1rkca1 97606

d1rkca2 97607

d2b0ha1 127638

d2a0ba_ 16630

d1a0ba_ 16631

d1bdjb_ 16632

d1fr0a_ 59996

d2q4fa_ 139847

d2q4fb_ 139848

d1yvia1 124099

d1yvib_ 124100

d1wn0a1 121070

d1wn0b_ 121071

d1wn0c_ 121072

d1wn0d_ 121073

d2r25a_ 151529

d1c02a_ 16633

d1c02b_ 16634

d1oxka_ 93690

d1oxkc_ 93692

d1oxke_ 93694

d1oxkg_ 93696

d1oxki_ 93698

d1oxkk_ 93700

d1c03a_ 16635

d1c03b_ 16636

d1c03c_ 16637

d1c03d_ 16638

d1oxba_ 93681

d1qspa_ 16639

d1qspb_ 16640

d1i5na_ 61805

d1i5nb_ 61806

d1i5nc_ 61807

d1i5nd_ 61808

d1tqga_ 107224

d1sr2a_ 112109

d1y6da_ 116504

d2ooca1 148919

d2oocb_ 148920

d1he1a_ 16641

d1he1b_ 16642

d1he9a_ 60971

d1g4us1 16643

d1g4wr1 16644

d1hy5a_ 65955

d1hy5b_ 65956

d1f1ma_ 59598

d1f1mb_ 59599

d1f1mc_ 59600

d1f1md_ 59601

d1g5za_ 60298

d1ggqa_ 60486

d1ggqb_ 60487

d1ggqc_ 60488

d1ggqd_ 60489

d1yjga_ 162215

d1yjgb_ 162216

d1yjgd_ 162217

d1yjge_ 162218

d2ga0a_ 164612

d2ga0b_ 164613

d2ga0c_ 164614

d2ga0d_ 164615

d2ga0e_ 164616

d2ga0f_ 164617

d2ga0g_ 164618

d2ga0h_ 164619

d1wgwa_ 114625

d2qy9a1 151469

d1ftsa1 16968

d1vmaa1 108887

d1vmab1 108889

d1okkd1 93261

d2j7pd1 138123

d2j7pe1 138125

d1rj9a1 97553

d2iyld1 137801

d2cnwd1 130655

d2cnwe1 130657

d2cnwf1 130659

d1j8yf1 62747

d1j8mf1 62742

d1qzxa1 96711

d1qzxb1 96714

d1qzwa1 96699

d1qzwc1 96702

d1qzwe1 96705

d1qzwg1 96708

d1ls1a1 78170

d2j45a1 137983

d2j45b1 137985

d2j46a1 137987

d2j46b1 137989

d2c04a1 129570

d2c04b1 129572

d2c03a1 129566

d2c03b1 129568

d1okka1 93259

d2j7pa1 138119

d2j7pb1 138121

d1rj9b1 97555

d1jpna1 67039

d1jpnb1 67041

d1ffha1 16960

d1ng1a1 16961

d2ng1a1 16962

d1o87a1 92640

d1o87b1 92642

d3ng1a1 16963

d3ng1b1 16964

d2cnwa1 130649

d2cnwb1 130651

d2cnwc1 130653

d1jpja1 67024

d2ffha1 16965

d2ffhb1 16966

d2ffhc1 16967

d2iy3a1 137790

d1qvxa_ 96448

d1pv3a_ 104321

d1ktma_ 77541

d1k04a_ 67904

d1ow6a_ 93631

d1ow6b_ 93632

d1ow6c_ 93633

d1ow7a_ 93634

d1ow7b_ 93635

d1ow7c_ 93636

d1k05a_ 67905

d1k05b_ 67906

d1k05c_ 67907

d1ow8a_ 93637

d1ow8b_ 93638

d1ow8c_ 93639

d1k40a_ 68123

d3s9oa_ 195214

d3s9ob_ 195213

d3s9oc_ 195212

d3b71a_ 154912

d3b71b_ 154913

d3b71c_ 154914

d3gm3a_ 176740

d3gm2a_ 176739

d3gm1a_ 176737

d3gm1b_ 176738

d3u5sa_ 194476

d3tk0a_ 194758

d3o55a_ 182806

d3u2la_ 195210

d3u2ma_ 195211

d1oqca_ 87264

d1oqcb_ 87265

d1oqcc_ 87266

d1oqcd_ 87267

d1jr8a_ 67115

d1jr8b_ 67116

d1jraa_ 67117

d1jrab_ 67118

d1jrac_ 67119

d1jrad_ 67120

d4e0ha_ 194766

d3w4ya_ 196691

d3w4yc_ 196692

d2hj3a_ 165120

d2hj3b_ 165121

d1knya1 75896

d1knyb1 75898

d1kana1 75878

d1kanb1 75880

d1joga_ 77142

d1jogb_ 77143

d1jogc_ 77144

d1jogd_ 77145

d1wtya_ 114880

d1wtyb_ 114881

d1wtyc_ 114882

d1wtyd_ 114883

d2ywaa1 153807

d2ywab1 153808

d2ywac1 153809

d2ywad1 153810

d1o3ua_ 86615

d1ufba_ 99331

d1ufbb_ 99332

d1ufbc_ 99333

d1ufbd_ 99334

d1v4aa1 108350

d1wola_ 161974

d1l3pa_ 84520

d1nlxa_ 80637

d1nlxb_ 80638

d1nlxc_ 80639

d1nlxd_ 80640

d1nlxe_ 80641

d1nlxf_ 80642

d1nlxg_ 80643

d1nlxh_ 80644

d1nlxi_ 80645

d1nlxj_ 80646

d1nlxk_ 80647

d1nlxl_ 80648

d1nlxm_ 80649

d1nlxn_ 80650

d1nzea_ 92374

d1orja_ 93456

d1orjb_ 93457

d1orjc_ 93458

d1orjd_ 93459

d1orya_ 93466

d1vh6a_ 100644

d1vh6b_ 100645

d3iqca_ 178534

d3iqcb_ 178535

d3k1ia_ 178949

d3k1ib_ 178950

d1v74b_ 100443

d1tfkb_ 119232

d1tfob_ 119234

d1gm5a1 65298

d1t6ua_ 106584

d1t6ub_ 106585

d1t6uc_ 106586

d1t6ud_ 106587

d1t6ue_ 106588

d1t6uf_ 106589

d1t6ug_ 106590

d1t6uh_ 106591

d1t6ui_ 106592

d1t6uj_ 106593

d1t6uk_ 106594

d1t6ul_ 106595

d1t6qa_ 106579

d1t6qb_ 106580

d1t6qc_ 106581

d1t6ia_ 106571

d1t6ib_ 106572

d1t6ic_ 106573

d1q0ga_ 104428

d1q0gb_ 104429

d1q0gc_ 104430

d1q0gd_ 104431

d1q0ge_ 104432

d1q0gf_ 104433

d1q0gg_ 104434

d1q0gh_ 104435

d1q0gi_ 104436

d1q0gj_ 104437

d1q0gk_ 104438

d1q0gl_ 104439

d1q0ma_ 104458

d1q0mb_ 104459

d1q0mc_ 104460

d1q0md_ 104461

d1q0me_ 104462

d1q0mf_ 104463

d1q0fa_ 104416

d1q0fb_ 104417

d1q0fc_ 104418

d1q0fd_ 104419

d1q0fe_ 104420

d1q0ff_ 104421

d1q0fg_ 104422

d1q0fh_ 104423

d1q0fi_ 104424

d1q0fj_ 104425

d1q0fk_ 104426

d1q0fl_ 104427

d1q0da_ 104404

d1q0db_ 104405

d1q0dc_ 104406

d1q0dd_ 104407

d1q0de_ 104408

d1q0df_ 104409

d1q0dg_ 104410

d1q0dh_ 104411

d1q0di_ 104412

d1q0dj_ 104413

d1q0dk_ 104414

d1q0dl_ 104415

d1q0ka_ 104443

d1q0kb_ 104444

d1q0kc_ 104445

d1q0kd_ 104446

d1q0ke_ 104447

d1q0kf_ 104448

d1q0kg_ 104449

d1q0kh_ 104450

d1q0ki_ 104451

d1q0kj_ 104452

d1q0kk_ 104453

d1q0kl_ 104454

d3g4za_ 176365

d3g4zb_ 176366

d3g4zc_ 176367

d3g50a_ 176368

d3g50b_ 176369

d3g50c_ 176370

d3g4xa_ 176360

d3g4xb_ 176361

d3g4xc_ 176362

d1szia_ 106159

d1ug7a_ 107824

d1xzpa1 116254

d1xzqa1 116258

d2huja1 136777

d2etsa1 132371

d2im8a_ 137506

d2im8b_ 137507

d2hfia1 136385

d2ap3a1 127110

d2g3ka1 134562

d2g3kb1 134563

d2g3kc1 134564

d2g3kd1 134565

d2g3ke1 134566

d2g3kf1 134567

d2g3kg1 134568

d2j9ua_ 138155

d2j9uc_ 138156

d2j9va_ 138157

d2j9wa_ 165957

d2j9wb_ 165958

d2p61a1 149266

d1bg7a_ 16686

d1rcda_ 16687

d1rcia_ 16688

d1rcga_ 16689

d1rcea_ 16691

d1rcca_ 16690

d1mfra_ 16692

d1mfrb_ 16693

d1mfrc_ 16694

d1mfrd_ 16695

d1mfre_ 16696

d1mfrf_ 16697

d1mfrg_ 16698

d1mfrh_ 16699

d1mfri_ 16700

d1mfrj_ 16701

d1mfrk_ 16702

d1mfrl_ 16703

d1mfrm_ 16704

d1mfrn_ 16705

d1mfro_ 16706

d1mfrp_ 16707

d1mfrq_ 16708

d1mfrr_ 16709

d1mfrs_ 16710

d1mfrt_ 16711

d1mfru_ 16712

d1mfrv_ 16713

d1mfrw_ 16714

d1mfrx_ 16715

d1z6om1 124543

d1z6oa1 124531

d2v2sa_ 168318

d2za7a_ 154275

d3f37a_ 175435

d3np0x_ 182453

d2w0oa_ 153737

d3nozx_ 182452

d2v2na_ 168314

d3af7x_ 172031

d2zg8x_ 171202

d2z5pa_ 154164

d3af8x_ 172032

d3f32a_ 175430

d2zg7x_ 154419

d3f36a_ 175434

d3f33a_ 175431

d3f38a_ 175436

d3h7ga_ 177305

d3f34a_ 175432

d2zg9x_ 171203

d1xz3a_ 122468

d2zurx_ 171533

d2za6a_ 154274

d2gyda_ 135856

d3o7sa_ 182862

d1xz1a_ 122467

d3af9x_ 172033

d3fi6a_ 175820

d3f39a_ 175437

d3rava_ 184874

d3u90a_ 195499

d2v2oa_ 168315

d3f35a_ 175433

d3o7ra_ 182861

d3rd0a_ 184884

d2v2ia_ 152423

d1gwga_ 70669

d2g4ha_ 134594

d1aewa_ 16676

d2z5qa_ 154165

d1data_ 16677

d2v2ja_ 152424

d1iera_ 16678

d1hrsa_ 16685

d1iesa_ 16679

d1iesb_ 16680

d1iesc_ 16681

d1iesd_ 16682

d1iese_ 16683

d1iesf_ 16684

d2z5ra_ 154166

d2ffxj_ 164342

d2fg4a_ 164344

d2fg8a_ 164345

d2fg8b_ 164346

d2fg8c_ 164347

d2fg8d_ 164348

d2fg8e_ 164349

d2fg8f_ 164350

d2fg8g_ 164351

d2fg8h_ 164352

d2fhaa_ 16674

d1fhaa_ 16675

d1r03a_ 104675

d1lb3a_ 77873

d1h96a_ 60811

d1sofa1 118973

d1nf4a_ 85598

d1nf4b_ 85599

d1nf4c_ 85600

d1nf4d_ 85601

d1nf4e_ 85602

d1nf4f_ 85603

d1nf4g_ 85604

d1nf4h_ 85605

d1nf4i_ 85606

d1nf4j_ 85607

d1nf4k_ 85608

d1nf4l_ 85609

d1nf4m_ 85610

d1nf4n_ 85611

d1nf4o_ 85612

d1nf4p_ 85613

d1nfva_ 85642

d1nfvb_ 85643

d1nfvc_ 85644

d1nfvd_ 85645

d1nfve_ 85646

d1nfvf_ 85647

d1nfvg_ 85648

d1nfvh_ 85649

d1nfvi_ 85650

d1nfvj_ 85651

d1nfvk_ 85652

d1nfvl_ 85653

d1nfvm_ 85654

d1nfvn_ 85655

d1nfvo_ 85656

d1nfvp_ 85657

d1nf6a_ 85614

d1nf6b_ 85615

d1nf6c_ 85616

d1nf6d_ 85617

d1nf6e_ 85618

d1nf6f_ 85619

d1nf6g_ 85620

d1nf6h_ 85621

d1nf6i_ 85622

d1nf6j_ 85623

d1nf6k_ 85624

d1nf6l_ 85625

d1nf6m_ 85626

d1nf6n_ 85627

d1nf6o_ 85628

d1nf6p_ 85629

d3e2ca_ 174561

d3e2cb_ 191711

d3ghqa_ 176638

d3ghqb_ 176639

d3ghqc_ 176640

d3ghqd_ 176641

d3ghqe_ 176642

d3ghqf_ 176643

d3ghqg_ 176644

d3ghqh_ 176645

d3ghqi_ 176646

d3ghqj_ 176647

d3ghqk_ 176648

d3ghql_ 176649

d3e1ja_ 174449

d3e1jb_ 174450

d3e1jc_ 174451

d3e1jd_ 174452

d3e1je_ 174453

d3e1jf_ 174454

d3e1jg_ 174455

d3e1jh_ 174456

d3e1ji_ 174457

d3e1jj_ 174458

d3e1jk_ 174459

d3e1jl_ 174460

d2y3qa_ 170565

d2y3qb_ 170566

d2y3qc_ 170567

d2y3qd_ 170568

d2y3qe_ 170569

d2y3qf_ 170570

d2y3qg_ 170571

d2y3qh_ 170572

d2y3qi_ 170573

d2y3qj_ 170574

d2y3qk_ 170575

d2y3ql_ 170576

d2vxia_ 168905

d2vxib_ 168906

d2vxic_ 168907

d2vxid_ 168908

d2vxie_ 168909

d2vxif_ 168910

d2vxig_ 168911

d2vxih_ 168912

d2vxii_ 168913

d2vxij_ 168914

d2vxik_ 168915

d2vxil_ 168916

d2htna_ 136753

d2htnb_ 136754

d2htnc_ 136755

d2htnd_ 136756

d2htne_ 136757

d2htnf_ 136758

d2htng_ 136759

d2htnh_ 136760

d3e1pa_ 174509

d3e1pb_ 174510

d3e1pc_ 174511

d3e1pd_ 174512

d3e1pe_ 174513

d3e1pf_ 174514

d3e1pg_ 174515

d3e1ph_ 174516

d3e1pi_ 174517

d3e1pj_ 174518

d3e1pk_ 174519

d3e1pl_ 174520

d3e1la_ 174461

d3e1lb_ 174462

d3e1lc_ 174463

d3e1ld_ 174464

d3e1le_ 174465

d3e1lf_ 174466

d3e1lg_ 174467

d3e1lh_ 174468

d3e1li_ 174469

d3e1lj_ 174470

d3e1lk_ 174471

d3e1ll_ 174472

d3e1qa_ 174521

d3e1qb_ 174522

d3e1qc_ 174523

d3e1qd_ 174524

d3e1qe_ 174525

d3e1qf_ 174526

d3e1qg_ 174527

d3e1qh_ 174528

d3e1qi_ 174529

d3e1qj_ 174530

d3e1qk_ 174531

d3e1ql_ 174532

d3e1ma_ 174473

d3e1mb_ 174474

d3e1mc_ 174475

d3e1md_ 174476

d3e1me_ 174477

d3e1mf_ 174478

d3e1mg_ 174479

d3e1mh_ 174480

d3e1mi_ 174481

d3e1mj_ 174482

d3e1mk_ 174483

d3e1ml_ 174484

d1bcfa_ 16648

d1bcfb_ 16649

d1bcfc_ 118423

d1bcfd_ 118424

d1bcfe_ 118425

d1bcff_ 118426

d1bcfg_ 118427

d1bcfh_ 118428

d1bcfi_ 118429

d1bcfj_ 118430

d1bcfk_ 118431

d1bcfl_ 118432

d1bfra_ 16650

d1bfrb_ 16651

d1bfrc_ 16652

d1bfrd_ 16653

d1bfre_ 16654

d1bfrf_ 16655

d1bfrg_ 16656

d1bfrh_ 16657

d1bfri_ 16658

d1bfrj_ 16659

d1bfrk_ 16660

d1bfrl_ 16661

d1bfrm_ 16662

d1bfrn_ 16663

d1bfro_ 16664

d1bfrp_ 16665

d1bfrq_ 16666

d1bfrr_ 16667

d1bfrs_ 16668

d1bfrt_ 16669

d1bfru_ 16670

d1bfrv_ 16671

d1bfrw_ 16672

d1bfrx_ 16673

d3e1oa_ 174497

d3e1ob_ 174498

d3e1oc_ 174499

d3e1od_ 174500

d3e1oe_ 174501

d3e1of_ 174502

d3e1og_ 174503

d3e1oh_ 174504

d3e1oi_ 174505

d3e1oj_ 174506

d3e1ok_ 174507

d3e1ol_ 174508

d3e1na_ 174485

d3e1nb_ 174486

d3e1nc_ 174487

d3e1nd_ 174488

d3e1ne_ 174489

d3e1nf_ 174490

d3e1ng_ 174491

d3e1nh_ 174492

d3e1ni_ 174493

d3e1nj_ 174494

d3e1nk_ 174495

d3e1nl_ 174496

d1jgca_ 66673

d1jgcb_ 66674

d1jgcc_ 66675

d1o9ra_ 86706

d1o9rb_ 86707

d1o9rc_ 86708

d1o9rd_ 86709

d1o9re_ 86710

d1o9rf_ 86711

d1ji5a_ 71678

d1ji5b_ 71679

d1ji5c_ 71680

d1ji5d_ 71681

d1jiga_ 71685

d1jigb_ 71686

d1jigc_ 71687

d1jigd_ 71688

d1n1qa_ 85260

d1n1qb_ 85261

d1n1qc_ 85262

d1n1qd_ 85263

d1dpsa_ 16716

d1dpsb_ 16717

d1dpsc_ 16718

d1dpsd_ 16719

d1dpse_ 16720

d1dpsf_ 16721

d1dpsg_ 16722

d1dpsh_ 16723

d1dpsi_ 16724

d1dpsj_ 16725

d1dpsk_ 16726

d1dpsl_ 16727

d1f33a_ 83228

d1f33b_ 83229

d1f33c_ 83230

d1f33d_ 83231

d1f33e_ 83232

d1f33f_ 83233

d1f33g_ 83234

d1f33h_ 83235

d1f33i_ 83236

d1f33j_ 83237

d1f33k_ 83238

d1f33l_ 83239

d1jrea_ 84193

d1jreb_ 84194

d1jrec_ 84195

d1jred_ 84196

d1jree_ 84197

d1jref_ 84198

d1jreg_ 84199

d1jreh_ 84200

d1jrei_ 84201

d1jrej_ 84202

d1jrek_ 84203

d1jrel_ 84204

d1jtsa_ 84206

d1jtsb_ 84207

d1jtsc_ 84208

d1jtsd_ 84209

d1jtse_ 84210

d1jtsf_ 84211

d1jtsg_ 84212

d1jtsh_ 84213

d1jtsi_ 84214

d1jtsj_ 84215

d1jtsk_ 84216

d1jtsl_ 84217

d1jtsm_ 84218

d1jtsn_ 84219

d1jtso_ 84220

d1jtsp_ 84221

d1jtsq_ 84222

d1jtsr_ 84223

d1jtss_ 84224

d1jtst_ 84225

d1jtsu_ 84226

d1jtsv_ 84227

d1jtsw_ 84228

d1jtsx_ 84229

d1l8ia_ 84550

d1l8ib_ 84551

d1l8ic_ 84552

d1l8id_ 84553

d1l8ie_ 84554

d1l8if_ 84555

d1l8ig_ 84556

d1l8ih_ 84557

d1l8ii_ 84558

d1l8ij_ 84559

d1l8ik_ 84560

d1l8il_ 84561

d1f30a_ 83216

d1f30b_ 83217

d1f30c_ 83218

d1f30d_ 83219

d1f30e_ 83220

d1f30f_ 83221

d1f30g_ 83222

d1f30h_ 83223

d1f30i_ 83224

d1f30j_ 83225

d1f30k_ 83226

d1f30l_ 83227

d1l8ha_ 84538

d1l8hb_ 84539

d1l8hc_ 84540

d1l8hd_ 84541

d1l8he_ 84542

d1l8hf_ 84543

d1l8hg_ 84544

d1l8hh_ 84545

d1l8hi_ 84546

d1l8hj_ 84547

d1l8hk_ 84548

d1l8hl_ 84549

d1tjoa_ 112461

d1tjob_ 112462

d1tjoc_ 112463

d1tjod_ 112464

d1moja_ 91367

d1mojb_ 91368

d1mojc_ 91369

d1mojd_ 91370

d1tk6a_ 112466

d1tk6b_ 112467

d1tk6c_ 112468

d1tk6d_ 112469

d1tkpa_ 112478

d1tkpb_ 112479

d1tkpc_ 112480

d1tkpd_ 112481

d1tkoa_ 112474

d1tkob_ 112475

d1tkoc_ 112476

d1tkod_ 112477

d1ji4a_ 77117

d1ji4b_ 77118

d1ji4c_ 77119

d1ji4d_ 77120

d1ji4e_ 77121

d1ji4f_ 77122

d1ji4g_ 77123

d1ji4h_ 77124

d1ji4i_ 77125

d1ji4j_ 77126

d1ji4k_ 77127

d1ji4l_ 77128

d1zuja1 125672

d1zujb_ 125673

d1zujc_ 125674

d1zujd_ 125675

d1zs3a1 125573

d1zs3b_ 125574

d1zs3c_ 125575

d1zs3d_ 125576

d1zs3e_ 125577

d1zs3f_ 125578

d1zs3g_ 125579

d1zs3h_ 125580

d1zs3i_ 125581

d1zs3j_ 125582

d1zs3k_ 125583

d1zs3l_ 125584

d2bk6a1 128660

d2bkca1 128675

d1qgha_ 16728

d1qghb_ 16729

d1qghc_ 16730

d1qghd_ 16731

d1qghe_ 16732

d1qghf_ 16733

d1qghg_ 16734

d1qghh_ 16735

d1qghi_ 16736

d1qghj_ 16737

d1qghk_ 16738

d1qghl_ 16739

d2bjya1 128634

d2pyba_ 167338

d2pybb_ 167339

d2pybc_ 167340

d2pybd_ 167341

d2yw6a_ 153794

d2yw6b_ 153795

d2yw6c_ 153796

d1vela_ 108532

d1velb_ 108533

d1velc_ 108534

d1veld_ 108535

d1vele_ 108536

d1velf_ 108537

d1veia_ 108531

d2yw7a1 153797

d2yw7b1 153798

d2yw7c1 153799

d2yw7d1 153800

d2yw7e1 153801

d2yw7f1 153802

d2yw7g1 153803

d2yw7h1 153804

d2yw7i1 153805

d2yw7j1 153806

d1veqa_ 108538

d1veqb_ 108539

d1veqc_ 108540

d1veqd_ 108541

d1veqe_ 108542

d1veqf_ 108543

d1veqg_ 108544

d1veqh_ 108545

d1veqi_ 108546

d1veqj_ 108547

d1veqk_ 108548

d1veql_ 108549

d2ux1a_ 152237

d2ux1b_ 152238

d2ux1c_ 152239

d2ux1d_ 152240

d2ux1e_ 152241

d2ux1f_ 152242

d2ux1g_ 152243

d2ux1h_ 152244

d2ux1i_ 152245

d2ux1j_ 152246

d2ux1k_ 152247

d2ux1l_ 152248

d1umna_ 99607

d1umnb_ 99608

d1umnc_ 99609

d1umnd_ 99610

d1umne_ 99611

d1umnf_ 99612

d1umng_ 99613

d1umnh_ 99614

d1umni_ 99615

d1umnj_ 99616

d1umnk_ 99617

d1umnl_ 99618

d2xjna_ 170146

d2xjnb_ 170147

d2xjnc_ 170148

d2xjnd_ 170149

d2xjne_ 170150

d2xjnf_ 170151

d2xjng_ 170152

d2xjnh_ 170153

d2xjni_ 170154

d2xjnj_ 170155

d2xjnk_ 170156

d2xjnl_ 170157

d2xjoa_ 170158

d2xjob_ 170159

d2xjoc_ 170160

d2xjod_ 170161

d2xjoe_ 170162

d2xjof_ 170163

d2xjog_ 170164

d2xjoh_ 170165

d2xjoi_ 170166

d2xjoj_ 170167

d2xjok_ 170168

d2xjol_ 170169

d2v15a_ 152372

d2v15b_ 152373

d2v15c_ 152374

d2v15d_ 152375

d2v15e_ 152376

d2v15f_ 152377

d2v15g_ 152378

d2v15h_ 152379

d2v15i_ 152380

d2v15j_ 152381

d2v15k_ 152382

d2v15l_ 152383

d2bw1a_ 129305

d2bw1b_ 129306

d2bw1c_ 129307

d2bw1d_ 129308

d2bw1e_ 129309

d2bw1f_ 129310

d2bw1g_ 129311

d2bw1h_ 129312

d2bw1i_ 129313

d2bw1j_ 129314

d2bw1k_ 129315

d2bw1l_ 129316

d2xjma_ 170134

d2xjmb_ 170135

d2xjmc_ 170136

d2xjmd_ 170137

d2xjme_ 170138

d2xjmf_ 170139

d2xjmg_ 170140

d2xjmh_ 170141

d2xjmi_ 170142

d2xjmj_ 170143

d2xjmk_ 170144

d2xjml_ 170145

d2xkqa_ 170182

d2xkqb_ 170183

d2xkqc_ 170184

d2xkqd_ 170185

d2xkqe_ 170186

d2xkqf_ 170187

d2xkqg_ 170188

d2xkqh_ 170189

d2xkqi_ 170190

d2xkqj_ 170191

d2xkqk_ 170192

d2xkql_ 170193

d2fjca1 133559

d2fzfa1 134437

d2fzfb_ 134438

d1vjxa_ 100837

d1j30a_ 90806

d1j30b_ 90807

d1yuza1 124077

d1yuzb1 124079

d1yv1a1 124084

d1yv1b1 124086

d1yuxa1 124073

d1yuxb1 124075

d1s3qa1 118848

d1krqa_ 68855

d1euma_ 59507

d1eumb_ 59508

d1eumc_ 59509

d1eumd_ 59510

d1eume_ 59511

d1eumf_ 59512

d3e6sa_ 174701

d3e6sb_ 174702

d3e6sc_ 174703

d3e6sd_ 174704

d3e6se_ 174705

d3e6sf_ 174706

d4ispc_ 197406

d4ispe_ 196687

d3e6ra_ 174695

d3e6rb_ 174696

d3e6rc_ 174697

d3e6rd_ 174698

d3e6re_ 174699

d3e6rf_ 174700

d1vlga_ 108816

d1vlgb_ 108817

d1vlgc_ 108818

d1vlgd_ 108819

d1vlge_ 108820

d1vlgf_ 108821

d1vlgg_ 108822

d1vlgh_ 108823

d1lkpa1 78065

d1lkoa1 78063

d1lkma1 78061

d1s2za1 105230

d1qyba1 96579

d1s30a1 105232

d1dvba1 16645

d1ryta1 16646

d1b71a1 16647

d1jyba1 77206

d1nnqa1 92006

d1nnqb1 92008

d2hr5a1 136682

d2hr5b1 136684

d2fkza_ 133688

d2fkzb_ 133689

d2fkzc_ 133690

d2fkzd_ 133691

d2fkze_ 133692

d2fkzf_ 133693

d2fkzg_ 133694

d2fkzh_ 133695

d1sofb_ 118974

d1sofc_ 118975

d1sofd_ 118976

d1sofe_ 118977

d1soff_ 118978

d1sofg_ 118979

d1sofh_ 118980

d2fl0a_ 133696

d2fl0b_ 133697

d2fl0c_ 133698

d2fl0d_ 133699

d2fl0e_ 133700

d2fl0f_ 133701

d2fl0g_ 133702

d2fl0h_ 133703

d4am4b_ 193424

d3ge4a_ 176555

d3ge4b_ 176556

d3ge4c_ 176557

d3ge4d_ 176558

d3ge4e_ 176559

d3ge4f_ 176560

d3ge4g_ 176561

d3ge4h_ 176562

d3ge4i_ 176563

d3ge4j_ 176564

d3ge4k_ 176565

d3ge4l_ 176566

d3ka8a_ 179222

d3shxa_ 195943

d3ka3a_ 179219

d3ka6a_ 179221

d3ka4a_ 179220

d3ka9a_ 179223

d3sh6a_ 195944

d3se1a_ 195945

d4dasf_ 193291

d4di0b_ 196066

d3bvfa_ 172845

d3bvfb_ 172846

d3bvfc_ 172847

d3bvfd_ 172848

d3bvfe_ 172849

d3bvff_ 172850

d3bvka_ 172857

d3bvkb_ 172858

d3bvkc_ 172859

d3bvkd_ 172860

d3bvke_ 172861

d3bvkf_ 172862

d3bvla_ 172863

d3bvlb_ 172864

d3bvlc_ 172865

d3bvld_ 172866

d3bvle_ 172867

d3bvlf_ 172868

d3bvea_ 172839

d3bveb_ 172840

d3bvec_ 172841

d3bved_ 172842

d3bvee_ 172843

d3bvef_ 172844

d3bvia_ 172851

d3bvib_ 172852

d3bvic_ 172853

d3bvid_ 172854

d3bvie_ 172855

d3bvif_ 172856

d3egma_ 174926

d3egmb_ 174927

d3egmc_ 174928

d3egmd_ 174929

d3egme_ 174930

d3egmf_ 174931

d2v2pa_ 168316

d2za8a_ 154276

d2v2ma_ 168313

d2v2la_ 168312

d2v2ra_ 168317

d3np2x_ 182454

d2ciha_ 163415

d3ajoa_ 172211

d3ajqa_ 172213

d2chia_ 163404

d2cn7a_ 163457

d2ceia_ 130344

d4dyxa_ 194091

d2iu2a_ 165691

d3ajpa_ 172212

d4dyya_ 193757

d2clua_ 163445

d2cn6a_ 163456

d4dz0a_ 193756

d2z6ma_ 171072

d2z6mb_ 171073

d2z6mc_ 171074

d2z6md_ 171075

d2z6me_ 171076

d2z6mf_ 171077

d2z6mg_ 171078

d2z6mh_ 171079

d2z6mi_ 171080

d2z6mj_ 171081

d2z6mk_ 171082

d2z6ml_ 171083

d2bk6b_ 128661

d2bk6c_ 128662

d2bk6d_ 128663

d2bk6e_ 128664

d2bk6f_ 128665

d2bkcb_ 128676

d2bkcc_ 128677

d2bkcd_ 128678

d2bkce_ 128679

d2bkcf_ 128680

d2bkcg_ 128681

d2bkch_ 128682

d2bkci_ 128683

d2bkcj_ 128684

d2bkck_ 128685

d2bkcl_ 128686

d2bkcm_ 128687

d2bkcn_ 128688

d2bkco_ 128689

d2bkcp_ 128690

d2bkcq_ 128691

d2bkcr_ 128692

d2bkcs_ 128693

d2bkct_ 128694

d2bkcu_ 128695

d2bkcv_ 128696

d2bkcx_ 128697

d2bkcy_ 128698

d2bjyb_ 128635

d2bjyc_ 128636

d2bjyd_ 128637

d2bjye_ 128638

d2bjyf_ 128639

d2bjyg_ 128640

d2bjyh_ 128641

d2bjyi_ 128642

d2bjyj_ 128643

d2bjyk_ 128644

d2bjyl_ 128645

d2iy4a_ 165741

d2iy4b_ 165742

d2iy4c_ 165743

d2iy4d_ 165744

d2iy4e_ 165745

d2iy4f_ 165746

d2iy4g_ 165747

d2iy4h_ 165748

d2iy4i_ 165749

d2iy4j_ 165750

d2iy4k_ 165751

d2iy4l_ 165752

d2iy4m_ 165753

d2iy4n_ 165754

d2iy4o_ 165755

d2iy4p_ 165756

d2iy4q_ 165757

d2iy4r_ 165758

d2iy4s_ 165759

d2iy4t_ 165760

d2iy4u_ 165761

d2iy4v_ 165762

d2iy4x_ 165763

d2iy4y_ 165764

d1uvha_ 119706

d1uvhb_ 119707

d1uvhc_ 119708

d1uvhd_ 119709

d4e6kf_ 193610

d3is7s_ 178588

d3gvya_ 177048

d3gvyb_ 177049

d3gvyc_ 177050

d3ak8i_ 172214

d3ak9l_ 196387

d2cf7a_ 163381

d2cf7b_ 163382

d2cf7c_ 163383

d2cf7d_ 163384

d2cf7e_ 163385

d2cf7f_ 163386

d2cf7g_ 163387

d2cf7h_ 163388

d2cf7i_ 163389

d2cf7j_ 163390

d2cf7k_ 163391

d2cf7l_ 163392

d1z4aa_ 162377

d1z4ab_ 162378

d1z4ac_ 162379

d1z4ad_ 162380

d1z4ae_ 162381

d1z4af_ 162382

d1z4ag_ 162383

d1z4ah_ 162384

d1z6ob_ 124532

d1z6oc_ 124533

d1z6od_ 124534

d1z6oe_ 124535

d1z6of_ 124536

d1z6og_ 124537

d1z6oh_ 124538

d1z6oi_ 124539

d1z6oj_ 124540

d1z6ok_ 124541

d1z6ol_ 124542

d3qz3a_ 184709

d3qz3b_ 184710

d3qz3c_ 184711

d4dyuk_ 195838

d1oq9a_ 87257

d1afra_ 16805

d1afrb_ 16806

d1afrc_ 16807

d1afrd_ 16808

d1afre_ 16809

d1afrf_ 16810

d1oq4a_ 87245

d1oq4b_ 87246

d1oq4c_ 87247

d1oq4d_ 87248

d1oq4e_ 87249

d1oq4f_ 87250

d1oqba_ 87258

d1oqbb_ 87259

d1oqbc_ 87260

d1oqbd_ 87261

d1oqbe_ 87262

d1oqbf_ 87263

d1oq7a_ 87251

d1oq7b_ 87252

d1oq7c_ 87253

d1oq7d_ 87254

d1oq7e_ 87255

d1oq7f_ 87256

d1mtyd_ 16742

d1mtye_ 16743

d1fz1a_ 16744

d1fz1b_ 16745

d1fz3a_ 16748

d1fz3b_ 16749

d1fz7a_ 16752

d1fz7b_ 16753

d1fz0a_ 16756

d1fz0b_ 16757

d1fyza_ 16760

d1fyzb_ 16761

d1fz2a_ 16764

d1fz2b_ 16765

d1fz8a_ 60122

d1fz8b_ 60123

d1fz6a_ 60116

d1fz6b_ 60117

d1mmod_ 16770

d1mmoe_ 16771

d1fz9a_ 60128

d1fz9b_ 60129

d1fz5a_ 16776

d1fz5b_ 16777

d1fz4a_ 16772

d1fz4b_ 16773

d1fzha_ 60134

d1fzhb_ 60135

d1fzia_ 60140

d1fzib_ 60141

d1mhyd_ 16780

d1mhzd_ 16782

d1mtyb_ 16740

d1mtyc_ 16741

d1xvbc_ 122346

d1xvbd_ 122347

d1xvgc_ 122376

d1xvgd_ 122377

d1fz1c_ 16746

d1fz1d_ 16747

d1xu5c_ 122328

d1xu5d_ 122329

d1fz3c_ 16750

d1fz3d_ 16751

d1xvfc_ 122370

d1xvfd_ 122371

d1fz7c_ 16754

d1fz7d_ 16755

d1xvcc_ 122352

d1xvcd_ 122353

d1fz0c_ 16758

d1fz0d_ 16759

d1fyzc_ 16762

d1fyzd_ 16763

d1fz2c_ 16766

d1fz2d_ 16767

d1fz8c_ 60124

d1fz8d_ 60125

d1fz6c_ 60118

d1fz6d_ 60119

d1mmob_ 16768

d1mmoc_ 16769

d1xmgc_ 122155

d1xmgd_ 122156

d1xvdc_ 122358

d1xvdd_ 122359

d1xmhc_ 122161

d1xmhd_ 122162

d1xu3c_ 122322

d1xu3d_ 122323

d1fz9c_ 60130

d1fz9d_ 60131

d1xvec_ 122364

d1xved_ 122365

d1xmfc1 122149

d1xmfd_ 122150

d1fz5c_ 16778

d1fz5d_ 16779

d1fz4c_ 16774

d1fz4d_ 16775

d1fzhc_ 60136

d1fzhd_ 60137

d4gamq_ 194032

d1fzic_ 60142

d1fzid_ 60143

d1mhyb_ 16781

d1mhzb_ 16783

d1otka_ 93521

d1otkb_ 93522

d1za0a1 124774

d1jk0a_ 63142

d1smqa_ 105766

d1smqb_ 105767

d1smqc_ 105768

d1smqd_ 105769

d1jk0b_ 63143

d1smsa_ 105770

d1smsb_ 105771

d1syya_ 106126

d3mjoa_ 181304

d3mjob_ 181305

d3dhza_ 157741

d3dhzb_ 157742

d1kgna_ 68584

d1kgnb_ 68585

d1kgnc_ 68586

d1kgnd_ 68587

d1kgpa_ 68592

d1kgpb_ 68593

d1kgpc_ 68594

d1kgpd_ 68595

d1oqua_ 93435

d1oqub_ 93436

d1oquc_ 93437

d1oqud_ 93438

d1kgoa_ 68588

d1kgob_ 68589

d1kgoc_ 68590

d1kgod_ 68591

d1mxra_ 85197

d1mxrb_ 85198

d1jqca_ 63229

d1jqcb_ 63230

d1piya_ 94710

d1piyb_ 94711

d1jpra_ 63224

d1jprb_ 63225

d1pm2a_ 94887

d1pm2b_ 94888

d1yfda1 123059

d1xika_ 16784

d1xikb_ 16785

d1piza_ 94712

d1pizb_ 94713

d1pj1a_ 94716

d1pj1b_ 94717

d1pj0a_ 94714

d1pj0b_ 94715

d1r65a_ 97144

d1r65b_ 97145

d1rsra_ 97815

d1rsrb_ 97816

d1pima_ 88117

d1pimb_ 88118

d1biqa_ 16786

d1biqb_ 16787

d1piua_ 88119

d1piub_ 88120

d1riba_ 16788

d1ribb_ 16789

d1pfra_ 16790

d1pfrb_ 16791

d1rsva_ 97817

d1rsvb_ 97818

d2av8a_ 16792

d2av8b_ 16793

d1rnra_ 16798

d1rnrb_ 16799

d1av8a_ 16796

d1av8b_ 16797

d1mrra_ 16794

d1mrrb_ 16795

d2alxa_ 126986

d2uw2a1 152205

d1w68a_ 109216

d1w69a_ 109217

d1h0oa_ 70845

d1xsma_ 16804

d1h0na_ 70844

d1uzra_ 108178

d1uzrb_ 108179

d1uzrc_ 108180

d1r2fa_ 16800

d1r2fb_ 16801

d2r2fa_ 16802

d2r2fb_ 16803

d2bq1i1 128957

d2bq1j1 128958

d3n20a_ 181830

d3rnfa_ 185056

d3n1ya_ 181824

d3n1xa_ 181821

d3rnea_ 185053

d3rn9a_ 185043

d3rnba_ 185046

d3rnga_ 185059

d3n1za_ 181827

d3rnca_ 185049

d2inca_ 137524

d1t0qa_ 106220

d2inda_ 137527

d2rdba_ 161548

d1t0sa_ 106226

d1t0ra_ 106223

d3n20b_ 181831

d3rnfb_ 185057

d3n1yb_ 181825

d3n1xb_ 181822

d3rneb_ 185054

d3rn9b_ 185044

d3rnbb_ 185047

d3rngb_ 185060

d3n1zb_ 181828

d3rncb_ 185050

d2incb_ 137525

d1t0qb_ 106221

d2indb_ 137528

d2rdbb_ 151922

d1t0sb_ 106227

d1t0rb_ 106224

d2j2fa_ 165816

d2j2fb_ 165817

d2j2fc_ 165818

d2j2fd_ 165819

d2j2fe_ 165820

d2j2ff_ 165821

d3pvtb_ 184017

d3pvtc_ 184018

d3pvrb_ 184015

d3pvrc_ 184016

d3pvyb_ 184023

d3pvyc_ 184024

d3pw1b_ 184025

d3pw1c_ 184026

d3pwqa_ 184043

d3pwqb_ 184044

d3pwqe_ 184045

d3pwqg_ 184046

d3pwqi_ 184047

d3pwqj_ 184048

d3pwqk_ 184049

d3pwqr_ 184050

d3pw8a_ 184027

d3pw8b_ 184028

d2uw1a_ 168185

d2uw1b_ 168186

d3oljd_ 196199

d4djna_ 195471

d4djnb_ 195470

d3vpnb_ 193905

d3vpob_ 193904

d3vpmb_ 193903

d3ge3a_ 176552

d3q3ma_ 184186

d3q3md_ 184188

d3dhia_ 173947

d3q14a_ 184143

d3q3na_ 184192

d3dhga_ 173940

d3dhgd_ 173942

d3rmka_ 196049

d3i5ja_ 178096

d3q3oa_ 184195

d3dhha_ 173944

d3q2aa_ 184167

d3i63a_ 178105

d3ri7a_ 196051

d3ge8a_ 176570

d3ge8d_ 176572

d1o9ia_ 92671

d1o9ib_ 92672

d1o9ic_ 92673

d1o9id_ 92674

d1o9ie_ 92675

d1o9if_ 92676

d1jkva_ 71719

d1jkvb_ 71720

d1jkvc_ 71721

d1jkvd_ 71722

d1jkve_ 71723

d1jkvf_ 71724

d1jkua_ 71713

d1jkub_ 71714

d1jkuc_ 71715

d1jkud_ 71716

d1jkue_ 71717

d1jkuf_ 71718

d2cwla1 130927

d2v8ta_ 168377

d2v8tb_ 168378

d2v8ua_ 168379

d2v8ub_ 168380

d2cwlb_ 130928

d2gs4a1 135575

d2gs4b_ 135576

d2gyqa1 135863

d2gyqb_ 135864

d4erub_ 195227

d3k6ca_ 179119

d3k6cb_ 179120

d3k6cc_ 179121

d3k6cd_ 179122

d3k6ce_ 179123

d3k6cf_ 179124

d3k6cg_ 179125

d3k6ch_ 179126

d3k6ci_ 179127

d3k6cj_ 179128

d2oc5a1 148720

d2itba1 147793

d2itbb_ 147794

d2oh3a1 148774

d2ib0a1 147597

d2ib0b_ 161461

d1s3qb_ 118849

d1s3qc_ 118850

d1s3qd_ 118851

d1s3qe_ 118852

d1s3qf_ 118853

d1s3qg_ 118854

d1s3qh_ 118855

d1s3qi_ 118856

d1s3qj_ 118857

d1s3qk_ 118858

d1s3ql_ 118859

d1sq3a_ 118983

d1sq3b_ 118984

d1sq3c_ 118985

d1sq3d_ 118986

d1sq3e_ 118987

d1sq3f_ 118988

d1sq3g_ 118989

d1sq3h_ 118990

d1sq3i_ 118991

d1sq3j_ 118992

d1sq3k_ 118993

d1sq3l_ 118994

d2chpa_ 192685

d2chpb_ 192687

d2chpc_ 192686

d2chpd_ 163405

d3kwoa_ 179755

d3kwob_ 179756

d3kwoc_ 179757

d3kwod_ 179758

d4a25a_ 197070

d2z90a_ 171110

d2z90b_ 171111

d2z90c_ 171112

d2z90d_ 171113

d3bkna_ 172681

d3bknb_ 172682

d3bknc_ 172683

d3bknd_ 172684

d3bkne_ 172685

d3bknf_ 172686

d3bkng_ 172687

d3bknh_ 172688

d3bkni_ 172689

d3bknj_ 172690

d3bknk_ 172691

d3bknl_ 172692

d3uoiv_ 193335

d3uoiw_ 193334

d3qb9f_ 196064

d2wtlf_ 196492

d3r2ka_ 184782

d3r2ra_ 184790

d3r2ha_ 184781

d3r2ma_ 184784

d3r2la_ 184783

d3r2oa_ 184789

d3r2sa_ 184791

d2jd60_ 166011

d2jd61_ 166012

d2jd62_ 166013

d2jd63_ 166014

d2jd64_ 166015

d2jd65_ 166016

d2jd66_ 166017

d2jd67_ 166018

d2jd68_ 166019

d2jd69_ 166020

d2jd6a_ 166021

d2jd6b_ 166022

d2jd6c_ 166023

d2jd6d_ 166024

d2jd6e_ 166025

d2jd6f_ 166026

d2jd6g_ 166027

d2jd6h_ 166028

d2jd6i_ 166029

d2jd6j_ 166030

d2jd6k_ 166031

d2jd6l_ 166032

d2jd6m_ 166033

d2jd6n_ 166034

d2jd6o_ 166035

d2jd6p_ 166036

d2jd6q_ 166037

d2jd6r_ 166038

d2jd6s_ 166039

d2jd6t_ 166040

d2jd6u_ 166041

d2jd6v_ 166042

d2jd6w_ 166043

d2jd6x_ 166044

d2jd6y_ 166045

d2jd6z_ 166046

d2jd70_ 166047

d2jd71_ 166048

d2jd72_ 166049

d2jd73_ 166050

d2jd74_ 166051

d2jd75_ 166052

d2jd76_ 166053

d2jd77_ 166054

d2jd78_ 166055

d2jd79_ 166056

d2jd7a_ 166057

d2jd7b_ 166058

d2jd7c_ 166059

d2jd7d_ 166060

d2jd7e_ 166061

d2jd7f_ 166062

d2jd7g_ 166063

d2jd7h_ 166064

d2jd7i_ 166065

d2jd7j_ 166066

d2jd7k_ 166067

d2jd7l_ 166068

d2jd7m_ 166069

d2jd7n_ 166070

d2jd7o_ 166071

d2jd7p_ 166072

d2jd7q_ 166073

d2jd7r_ 166074

d2jd7s_ 166075

d2jd7t_ 166076

d2jd7u_ 166077

d2jd7v_ 166078

d2jd7w_ 166079

d2jd7x_ 166080

d2jd7y_ 166081

d2jd7z_ 166082

d2jd80_ 166083

d2jd81_ 166084

d2jd82_ 166085

d2jd83_ 166086

d2jd84_ 166087

d2jd85_ 166088

d2jd86_ 166089

d2jd87_ 166090

d2jd88_ 166091

d2jd89_ 166092

d2jd8a_ 166093

d2jd8b_ 166094

d2jd8c_ 166095

d2jd8d_ 166096

d2jd8e_ 166097

d2jd8f_ 166098

d2jd8g_ 166099

d2jd8h_ 166100

d2jd8i_ 166101

d2jd8j_ 166102

d2jd8k_ 166103

d2jd8l_ 166104

d2jd8m_ 166105

d2jd8n_ 166106

d2jd8o_ 166107

d2jd8p_ 166108

d2jd8q_ 166109

d2jd8r_ 166110

d2jd8s_ 166111

d2jd8t_ 166112

d2jd8u_ 166113

d2jd8v_ 166114

d2jd8w_ 166115

d2jd8x_ 166116

d2jd8y_ 166117

d2jd8z_ 166118

d2d5ka_ 163581

d2d5kb_ 163582

d2d5kc_ 163583

d2d5kd_ 163584

d2vxxa_ 168929

d2vxxb_ 168930

d2vxxc_ 168931

d2vxxd_ 168932

d2c41a_ 163244

d2c41b_ 163245

d2c41c_ 163246

d2c41d_ 163247

d2c41e_ 163248

d2c41f_ 163249

d2c41g_ 163250

d2c41h_ 163251

d2c41i_ 163252

d2c41j_ 163253

d2c41k_ 163254

d2c41l_ 163255

d2fjcb_ 133560

d2fjcc_ 133561

d2fjcd_ 133562

d2fjce_ 133563

d2fjcf_ 133564

d2fjcg_ 133565

d2fjch_ 133566

d2fjci_ 133567

d2fjcj_ 133568

d2fjck_ 133569

d2fjcl_ 133570

d2fjcm_ 133571

d2fjcn_ 133572

d2fjco_ 133573

d2fjcp_ 133574

d1z6on_ 124544

d1z6oo_ 124545

d1z6op_ 124546

d1z6oq_ 124547

d1z6or_ 124548

d1z6os_ 124549

d1z6ot_ 124550

d1z6ou_ 124551

d1z6ov_ 124552

d1z6ow_ 124553

d1z6ox_ 124554

d1niga_ 85740

d1noga_ 85923

d1rtya_ 97828

d1rtyb_ 97829

d1rtyc_ 97830

d2ah6a_ 162799

d2ah6b_ 162800

d2ah6c_ 162801

d2idxa_ 165518

d2idxb_ 165519

d2idxc_ 165520

d3ci3a_ 173246

d3gaha_ 176476

d3gaja_ 176478

d3gaia_ 176477

d3ci1a_ 173245

d3ci4a_ 173247

d2r6xa_ 168000

d2r6xb_ 168001

d2r6ta_ 167998

d2r6tb_ 167999

d2g2da_ 164561

d1wa8a1 120809

d3favb_ 175627

d3favd_ 175629

d1wa8b1 120810

d3fava_ 175626

d3favc_ 175628

d4j42a_ 193970

d4j11a_ 194002

d4j11b_ 194003

d4j11c_ 194006

d4j11d_ 194001

d4j10a_ 194004

d4j10b_ 194005

d4j7jb_ 193228

d4j41d_ 193242

d4j7kd_ 193227

d4iyhd_ 193243

d2g38a1 134549

d2g38c_ 134551

d2g38b1 134550

d2gtsa1 147176

d3fx7a_ 176128

d3fx7b_ 176129

d2nr5a1 148349

d2nr5b_ 148350

d2nr5c_ 148351

d2nr5d_ 148352

d2nr5e_ 148353

d2nr5f_ 148354

d2nr5g_ 148355

d2nr5h_ 148356

d1cnt1_ 16840

d1cnt2_ 16841

d1cnt3_ 16842

d1cnt4_ 16843

d1bgca_ 16822

d1bgea_ 16823

d1bgeb_ 16824

d1bgda_ 16825

d1rhga_ 16812

d1rhgb_ 16813

d1rhgc_ 16814

d1cd9a_ 16815

d1cd9c_ 16816

d1pgra_ 16817

d1pgrc_ 16818

d1pgre_ 16819

d1pgrg_ 16820

d1gnca_ 16821

d1huwa_ 16831

d1axia_ 16832

d1hwga_ 16834

d1a22a_ 16833

d3hhra_ 16835

d1kf9a_ 77351

d1kf9d_ 77356

d1hwha_ 16836

d1hgua_ 16837

d1bp3a_ 16838

d1f45b_ 59642

d1alua_ 16826

d1p9mb_ 87994

d1il6a_ 16827

d2il6a_ 16828

d1i1rb_ 61548

d1ax8a_ 16844

d1pvhb_ 95167

d1pvhd_ 95170

d1emra_ 59456

d2q7nb1 150094

d2q7nd1 150095

d1lkia_ 16829

d1a7ma_ 16830

d1evsa_ 16845

d3d48p_ 195459

d2q98a_ 167483

d1rw5a1 118792

d1f6fa_ 16839

d3ncea_ 196418

d3ncba_ 196420

d1z7ca_ 162396

d3ncca_ 196419

d3ncfa_ 196417

d2d9qa_ 131346

d1eera_ 16846

d1cn4c_ 16847

d1buya_ 16848

d1etea_ 16869

d1eteb_ 16870

d1etec_ 16871

d1eted_ 16872

d2gmfa_ 16849

d2gmfb_ 16850

d1csga_ 16851

d1csgb_ 16852

d3cxeb1 157108

d4i77z_ 196843

d3l5xa_ 179989

d3l5wi_ 179987

d3l5wj_ 179988

d3l5ya_ 179990

d1ik0a_ 71240

d1ijza_ 71239

d1ga3a_ 60411

d2z3qa1 154002

d2z3qc_ 154004

d2z3ra_ 154006

d2z3rc_ 154008

d2z3re_ 154010

d2z3rg_ 154012

d2z3ri_ 154014

d2z3rk_ 154016

d2z3rm_ 154018

d2z3ro_ 154020

d4gs7a_ 193400

d2psma1 149820

d2psmb_ 149821

d1m48a_ 74443

d1m48b_ 74444

d1m47a_ 74442

d1m49a_ 74445

d1m49b_ 74446

d1nbpa_ 80392

d1m4ba_ 74448

d2b5ia_ 127895

d1m4aa_ 74447

d3inkc_ 16881

d3inkd_ 16882

d1m4ca_ 74449

d1m4cb_ 74450

d1pw6a_ 95200

d1pw6b_ 95201

d1qvna_ 161678

d1qvnb_ 161679

d1qvnc_ 161680

d1qvnd_ 161681

d2erjd1 132294

d2erjh1 132301

d1z92a_ 124737

d1py2a_ 95319

d1py2b_ 95320

d1py2c_ 95321

d1py2d_ 95322

d1irla_ 16883

d3tgxj_ 196026

d3tgxp_ 196025

d2oqpa1 148997

d1jlia_ 16884

d2b8ua_ 128117

d1hzia_ 61449

d1iara_ 16853

d1rcba_ 16854

d2inta_ 16855

d1hika_ 16856

d3bpla_ 155485

d1hija_ 16857

d3bpna1 155488

d1itma_ 16858

d1bbna_ 16861

d1itla_ 16859

d2cyka_ 16863

d1itia_ 16864

d1cyla_ 16860

d1bcna_ 16862

d1hula_ 16865

d1hulb_ 16866

d1hmca_ 16867

d1hmcb_ 16868

d1scfa_ 16873

d1scfb_ 16874

d1scfc_ 16875

d1scfd_ 16876

d1exza_ 16877

d1exzb_ 16878

d1exzc_ 16879

d1exzd_ 16880

d2e9wc1 146738

d1v7mv_ 100458

d1v7mx_ 100459

d1v7nv_ 100476

d1v7nx_ 100477

d1v7ny_ 100478

d1v7nz_ 100479

d2d48a_ 163575

d2b8xa_ 163008

d2b8ya_ 163009

d2b91a_ 163012

d2b90a_ 163011

d3uf2i_ 193558

d4fa8e_ 193550

d4fa8g_ 193551

d2b8za_ 163010

d3qt2c_ 184613

d3qt2d_ 184614

d3qt2e_ 184615

d3qt2f_ 184616

d3va2b_ 193617

d2xqba_ 170286

d2o27a_ 166500

d2o27b_ 166501

d3uf5a_ 194779

d3uf5b_ 194778

d3ejja_ 174984

d3ejjb_ 174985

d2o26a_ 166496

d2o26b_ 166497

d2o26e_ 166498

d2o26f_ 166499

d3b5ka_ 172432

d3b5kb_ 172433

d3s9dc_ 196189

d1itfa_ 16899

d2hymb1 136900

d1rh2a_ 16893

d1rh2b_ 16894

d1rh2c_ 16895

d1rh2d_ 16896

d1rh2e_ 16897

d1rh2f_ 16898

d1au1a_ 16889

d1au1b_ 16890

d1wu3i_ 114890

d1ifaa_ 16892

d1d9ca_ 16901

d1d9cb_ 16902

d1d9ga_ 16903

d1d9gb_ 16904

d1rfba_ 16905

d1rfbb_ 16906

d1fyha1 16907

d1fyha2 16908

d1fyhd1 16909

d1fyhd2 16910

d1fg9a_ 16915

d1fg9b_ 16916

d1higa_ 16917

d1higb_ 16918

d1higc_ 16919

d1higd_ 16920

d1ekua1 16911

d1ekua2 16912

d1ekub1 16913

d1ekub2 16914

d2riga_ 16921

d1b5la_ 16900

d1vlka_ 16888

d1y6nl1 144600

d1y6ml_ 122665

d2ilka_ 16885

d1ilka_ 16886

d1lk3a_ 73950

d1lk3b_ 73951

d1inra_ 16887

d1y6kl_ 122662

d1j7vl_ 62704

d1lqsl_ 74192

d1lqsm_ 74193

d1n1fa_ 79803

d3dlqi_ 157802

d1m4ra_ 84799

d1m4rb_ 84800

d3q1si_ 184155

d1ykba_ 123503

d1ykbb_ 123504

d1ykbc_ 123505

d1ykbd_ 123506

d1ykbe_ 123507

d1ykbf_ 123508

d3g9vb_ 176455

d3g9vd_ 176456

d2h24a_ 135988

d3besl_ 155196

d3dgcl_ 173922

d3dgcm_ 173923

d3ux9a_ 195916

d3ux9c_ 195915

d3oq3a_ 183231

d4dohc_ 194952

d1f7ua1 59673

d1f7va1 59676

d1bs2a1 16930

d1iq0a1 66257

d1u0bb1 112904

d1li5a1 73912

d1li5b1 73914

d1li7a1 73917

d1li7b1 73919

d1ffya1 16926

d1qu2a1 16925

d1qu3a1 16927

d1ilea1 16924

d1jzsa1 67880

d1jzqa1 67869

d1h3na1 76641

d1obca1 86764

d1obha1 86773

d1pfva1 94660

d1pfwa1 94663

d1pg2a1 94671

d1p7pa1 94309

d1f4la1 59648

d1pfya1 94666

d1pfua1 94657

d1pg0a1 94669

d1qqta1 16923

d1rqga1 105063

d2d5ba1 131268

d1woya1 121130

d2d54a1 131264

d1a8ha1 16922

d1ivsa2 76856

d1ivsb2 76860

d1gaxa5 75843

d1gaxb5 75845

d1iywa2 83808

d1iywb2 83812

d2af8a_ 16933

d1af8a_ 16932

d1f80d_ 59686

d1f80e_ 59687

d1f80f_ 59688

d1hy8a_ 65959

d1t8ka_ 106666

d1l0ia_ 77643

d2fada_ 133191

d2fadb_ 133192

d2faca_ 133189

d2facb_ 133190

d1l0ha_ 77642

d2fhsc_ 133503

d1acpa_ 16931

d1klpa_ 72721

d4etwb_ 192817

d4etwd_ 192503

d1vkua_ 108690

d2qnwa_ 167737

d1or5a_ 87331

d2jq4a1 148170

d1nq4a_ 92045

d2pnga1 139742

d2ehsa_ 164042

d2ehta_ 164043

d2x2ba_ 169820

d2faea_ 133193

d2faeb_ 133194

d3ny7b_ 182625

d2cg5b_ 163399

d1x3oa_ 162033

d2gdwa1 135040

d1dnya_ 16934

d2gdxa1 135041

d2gdya1 135042

d1hqba_ 61128

d1dv5a_ 59139

d3gzma_ 177112

d3gzmb_ 177113

d3gzla_ 177111

d1unka_ 16935

d1unkb_ 16936

d1unkc_ 16937

d1unkd_ 16938

d1ceia_ 16939

d1ayia_ 16940

d2jb0a_ 138246

d1mz8a_ 79683

d1mz8c_ 79685

d1znva_ 125410

d1znvc_ 125411

d2jaza_ 138244

d2jazc_ 138245

d2jbga_ 138248

d2jbgc_ 138249

d1ujza_ 99475

d7ceia_ 16941

d1gxga_ 70705

d1gxha_ 70706

d2vlqa_ 153298

d2gyka1 145276

d2gyke_ 145277

d2gzja_ 147204

d2gzje_ 147206

d2vlna_ 153295

d2gzga_ 161447

d1fr2a_ 83256

d1emva_ 16944

d2gzfa_ 161446

d2gzea_ 161445

d2gzia_ 161448

d2vlpa_ 153297

d1bxia_ 16945

d3gklc_ 176723

d3gkld_ 176724

d3gjna_ 176705

d3gjnd_ 176708

d1impa_ 16946

d1imqa_ 16948

d1e0ha_ 16947

d3u43a_ 195766

d2vloa_ 153296

d2wpta_ 169554

d2eiaa1 16949

d2eiab1 16950

d1eiaa1 16951

d1a8oa_ 16953

d1baja_ 16954

d1a43a_ 16955

d1e6jp1 16956

d1auma_ 16957

d3dika1 157751

d1qrjb1 16952

d1d1da1 16958

d1eoqa_ 16959

d1y7qa1 122714

d1y7qb1 122715

d2fi2a1 133509

d2fi2b1 133510

d3lhra_ 180288

d3lhrb_ 180289

d4bhxa_ 196820

d4e6sa_ 195469

d3dwya_ 157925

d3dwyb_ 157926

d2d82a1 131327

d2rnya1 152175

d1jspb_ 71843

d1e6ia_ 16969

d1f68a_ 16970

d3gg3a_ 176610

d3gg3b_ 176611

d1wuma1 121297

d1zs5a1 125589

d1wuga1 121286

d1n72a_ 80213

d2rnxa1 152174

d2rnwa1 152173

d1jm4b_ 71746

d1eqfa1 16972

d1eqfa2 16973

d3p1fa_ 183451

d3p1fb_ 183452

d4a9ka_ 186740

d4a9kb_ 186741

d3p1ca_ 183445

d3p1cb_ 183446

d3p1ea_ 183449

d3p1eb_ 183450

d3svhb_ 192084

d3p1da_ 183447

d3p1db_ 183448

d3d7ca_ 157420

d3d7cb_ 157421

d3i3ja_ 178050

d3i3jb_ 178051

d3i3jc_ 178052

d3i3jd_ 178053

d3i3je_ 178054

d3i3jf_ 178055

d3i3jg_ 178056

d3i3jh_ 178057

d3i3ji_ 178058

d3i3jj_ 178059

d3i3jk_ 178060

d3i3jl_ 178061

d3u5la_ 186087

d3uvwa_ 186424

d4f3ia_ 192475

d2ossa_ 166839

d2nxba_ 166409

d2nxbb_ 166410

d3zyua_ 186663

d3zyub_ 186664

d3qzta_ 196346

d3ljwb_ 196449

d3u5ja_ 186082

d3onia_ 196416

d3mxfa_ 181665

d3p5oa_ 183539

d4gpja_ 192508

d3hmfa_ 196562

d4a9la_ 186742

d4hbya_ 192525

d4hbxa_ 192524

d2yela_ 170766

d4j0ra_ 192634

d3svga_ 192083

d4hbva_ 192526

d2grca_ 164810

d4j0sa_ 192633

d3qzsb_ 196347

d3nxba_ 182608

d3nxbb_ 182609

d1x0ja_ 162015

d1x0jb_ 162016

d1x0jc_ 162017

d3uvxa_ 186425

d3u5ka_ 186083

d3u5kb_ 186084

d3u5kc_ 186085

d3u5kd_ 186086

d3svfa_ 192082

d3uvda_ 186415

d3uvya_ 186426

d3s91a_ 185320

d4e96a_ 192366

d2rfja1 168085

d2rfjb1 168086

d2rfjc_ 168087

d2dvsa_ 163725

d2dvsb_ 163726

d2dvsc_ 163727

d2dvqa_ 163719

d2dvqb_ 163720

d2dvqc_ 163721

d2yema_ 197054

d2dvra_ 163722

d2dvrb_ 163723

d2dvrc_ 163724

d3aqaa_ 172302

d3aqab_ 172303

d3aqac_ 172304

d4flpb_ 194983

d3jvma_ 196527

d2dwwa_ 193390

d1u8va1 113196

d1u8vb1 113198

d1u8vc1 113200

d1u8vd1 113202

d2d29a1 131157

d2d29b1 131159

d2cx9a1 130985

d2cx9b1 130987

d2cx9c1 130989

d2cx9d1 130991

d1ws9a1 121223

d1ws9b1 121225

d1buca1 16590

d1bucb1 16591

d1jqia1 67087

d1jqib1 67089

d1siqa1 105585

d2r0na1 151506

d1sira1 105587

d2r0ma1 151504

d1rx0a1 98007

d1rx0b1 98009

d1rx0c1 98011

d1rx0d1 98013

d1ivha1 16608

d1ivhb1 16609

d1ivhc1 16610

d1ivhd1 16611

d1egda1 16596

d1egdb1 16597

d1egdc1 16598

d1egdd1 16599

d1egca1 16600

d1egcb1 16601

d1egcc1 16602

d1egcd1 16603

d2a1ta1 126005

d2a1tb1 126007

d2a1tc1 126009

d2a1td1 126011

d1egea1 16604

d1egeb1 16605

d1egec1 16606

d1eged1 16607

d1t9ga1 106711

d1t9gb1 106713

d1t9gc1 106715

d1t9gd1 106717

d3mdea1 16594

d3mdeb1 16595

d3mdda1 16592

d3mddb1 16593

d1udya1 99231

d1udyb1 99233

d1udyc1 99235

d1udyd1 99237

d1ukwa1 113263

d1ukwb1 113265

d2c0ua1 129607

d2reha1 151973

d2rehb1 151975

d2rehc1 151977

d2rehd1 151979

d2zafa1 154277

d2zafb1 154279

d2zafc1 154281

d2zafd1 154283

d1r2ja1 96869

d1w07a1 114046

d1w07a2 114047

d1w07b1 114049

d1w07b2 114050

d2ddha1 131394

d2ddha2 131395

d1is2a1 71382

d1is2a2 71383

d1is2b1 71385

d1is2b2 71386

d1gkza1 65268

d1gkxa1 65266

d1gjva1 65220

d2pnra1 149696

d2pnrb1 149698

d2pnre1 149701

d2pnrf1 149703

d1y8oa1 144603

d2q8ia1 150126

d1y8pa1 144605

d1y8na1 144601

d1jm6a1 66876

d1jm6b1 66878

d1rj1a_ 97526

d1rj4a_ 97527

d1rj4b_ 97528

d1rj4c_ 97529

d1rj4d_ 97530

d1x91a_ 114975

d1x90a_ 114973

d1x90b_ 114974

d1x8za_ 114970

d1x8zb_ 114971

d1x8zc_ 114972

d2cj4a_ 130518

d2cj4b_ 130519

d2cj7a_ 130522

d2cj5a_ 130520

d2cj6a_ 130521

d2xqrb_ 170293

d2xqrd_ 170294

d2xqrf_ 170295

d2xqrh_ 170296

d2xqrj_ 170297

d2xqrl_ 170298

d2cj8a_ 130523

d2cj8b_ 130524

d1xg2b_ 162063

d1r3ba_ 104786

d1pi1a_ 94699

d1tdpa_ 106781

d2bl8a1 146154

d2bl7a1 128729

d2bl8b_ 161392

d2bl8c_ 146155

d2etda1 132353

d1v9va1 119898

d2fefa1 133343

d2fefb1 133345

d2fefc1 133347

d2fug11 134121

d2fuga1 134134

d2fugj1 134147

d2fugs1 134160

d3dbya1 157486

d3dbya2 157487

d3dbye1 157494

d3dbyg1 157498

d3dbyi1 157502

d3dbyk1 157506

d3dbym1 157510

d3dbyo1 157514

d3dbys1 157522

d3d19a1 157176

d3d19a2 157177

d3d19b1 157178

d3d19c1 157180

d3d19d1 157182

d3d19e1 157184

d3d19e2 157185

d3d19f1 157186

d3d19f2 157187

d2qzga1 151482

d2qzgb_ 151483

d2qzgc_ 151484

d2qzgd_ 151485

d2qsba1 151315

d2hi7b1 145393

d2rlda1 152150

d2rldb_ 152151

d2rldc_ 152152

d2rldd_ 152153

d2rlde_ 152154

d2gsca1 147169

d2gscb_ 147170

d2gscc_ 147171

d2gscd_ 147172

d2gsce_ 147173

d2hgka1 147284

d1nkda_ 16974

d4do2a_ 194014

d4do2b_ 194013

d1rpoa_ 16975

d2ijka_ 147712

d2ijkb_ 147713

d1ropa_ 16976

d2ijha_ 165583

d2ijhb_ 165584

d2ijhc_ 165585

d3k79a_ 179147

d2ijja_ 165587

d2ijjb_ 165588

d2ijjc_ 165589

d1gtoa_ 16980

d1gtob_ 16981

d1gtoc_ 16982

d2ghya_ 135218

d2ghyb_ 135219

d2ijia_ 165586

d1rpra_ 16989

d1rprb_ 16990

d1b6qa_ 16977

d1f4na_ 16978

d1f4nb_ 16979

d1gmga_ 76234

d1gmgb_ 76235

d1qx8a_ 104641

d1qx8b_ 104642

d1f4ma_ 16983

d1f4mb_ 16984

d1f4mc_ 16985

d1f4md_ 16986

d1f4me_ 16987

d1f4mf_ 16988

d1joya_ 16991

d1joyb_ 16992

d1b3qa1 16993

d1b3qb1 16994

d2c2aa1 129662

d1pd3a_ 94512

d1pd3b_ 94513

d1ufia_ 99336

d1ufib_ 99337

d1ufic_ 99338

d1ufid_ 99339

d1skva_ 105690

d1skvb_ 105691

d1skvc_ 105692

d1skvd_ 105693

d1zkea1 125186

d1zkeb_ 125187

d1zkec_ 125188

d1zked_ 125189

d1zkee_ 125190

d1zkef_ 125191

d2bzba1 129556

d2bzbb1 129557

d2c0sa1 129606

d2az0a1 127578

d2az0b_ 127579

d2az2a_ 127586

d2az2b_ 127587

d2b9za1 128231

d2b9zb1 128232

d3im3a_ 178393

d3im4a_ 178394

d3im4b_ 178395

d2ezwa1 132645

d2ezwb1 132646

d1r2aa_ 16995

d1r2ab_ 16996

d2drna1 131672

d2drnb1 131673

d2h9ra1 136268

d2h9rb1 136269

d1l6ea_ 73607

d1l6eb_ 73608

d2izxa_ 165775

d2izxb_ 165776

d2izya_ 137830

d2izyb_ 137831

d2izyc_ 137832

d2izyd_ 137833

d2izye_ 137834

d2izyf_ 137835

d2izyg_ 137836

d2izyh_ 137837

d2hwna_ 136826

d2hwnb_ 136827

d2hwnc_ 136828

d2hwnd_ 136829

d1nh2b_ 91874

d1ytfb_ 16997

d1nvpb_ 92211

d1nh2d1 91876

d1rm1b1 118780

d1ytfd1 16998

d1nvpd1 92213

d1ecia_ 16999

d1ecib_ 17000

d1b0nb_ 17002

d1b0na1 17001

d1g2ya_ 17003

d1g2yb_ 17004

d1g2yc_ 17005

d1g2yd_ 17006

d1g2za_ 17007

d1g2zb_ 17008

d1g39a_ 17009

d1g39b_ 17010

d1g39c_ 17011

d1g39d_ 17012

d1jb6a_ 62834

d1jb6b_ 62835

d1f93e_ 17013

d1f93f_ 17014

d1f93g_ 17015

d1f93h_ 17016

d1pzqa_ 95466

d1pzqb_ 95467

d1q2ha_ 104498

d1q2hb_ 104499

d1q2hc_ 104500

d1ic8a2 76741

d1ic8b2 76743

d1e3oc2 59198

d1gt0c2 76336

d1hf0a2 65821

d1hf0b2 65823

d1octc2 17017

d1cqta2 17018

d1cqtb2 17019

d1o4xa2 92471

d1poua_ 17020

d1au7a2 17021

d1au7b2 17022

d1r69a_ 17028

d1perl_ 17029

d1perr_ 17030

d2or1l_ 17031

d2or1r_ 17032

d1rpel_ 17033

d1rper_ 17034

d1r63a_ 17035

d1praa_ 17037

d2r63a_ 17036

d1sq8a_ 105888

d2croa_ 17038

d3crol_ 17039

d3cror_ 17040

d1zuga_ 17041

d2ovga_ 166870

d2ecsa_ 163954

d2ecsb_ 163955

d1d1la_ 17057

d5croa_ 17044

d5crob_ 17045

d5croc_ 17046

d5croo_ 17043

d6croa_ 17047

d3orca_ 17061

d4croa_ 17048

d4crob_ 17049

d4croc_ 17050

d4crod_ 17051

d4croe_ 17052

d4crof_ 17053

d1copd_ 17058

d1cope_ 17059

d1orca_ 17054

d1d1ma_ 17056

d1d1mb_ 17055

d2orca_ 17060

d1rzsa_ 105139

d1lmb3_ 17023

d1lmb4_ 17024

d1llia_ 17025

d1llib_ 17026

d1rioa_ 97513

d1riob_ 97514

d1lrpa_ 17027

d1lrpb_ 118551

d1lrpc_ 118552

d1nera_ 17062

d1neqa_ 17063

d2r1jl_ 151525

d2r1jr_ 151526

d3jxbc_ 178900

d3jxbd_ 178901

d3jxcl_ 178902

d3jxcr_ 178903

d3jxdl_ 178904

d3jxdr_ 178905

d1adra_ 17042

d2icta_ 147605

d2icpa1 147604

d1zzcb1 125898

d1zz7a1 125876

d1zz7b1 125878

d1zz6a1 125872

d1zz6b1 125874

d1zz8a1 125880

d1zz8b1 125882

d1zz8c1 125884

d1zzba1 125892

d1zzbb1 125894

d1zz9a1 125886

d1zz9b1 125888

d1zz9c1 125890

d2xi8a_ 170111

d2xi8b_ 170112

d1utxa_ 108034

d1utxb_ 108035

d2gzua1 135915

d2gzub1 135916

d2ofya1 148762

d2ofyb_ 148763

d2b5aa1 127882

d2b5ab_ 127883

d2b5ac_ 127884

d2b5ad_ 127885

d1y7ya1 122729

d1b0na2 17064

d2ppxa1 149783

d1y7yb_ 122730

d2xj3a_ 170127

d2xj3b_ 170128

d2xiua_ 170120

d2xiub_ 170121

d1dwka1 17075

d1dwkb1 17076

d1dwkc1 17077

d1dwkd1 17078

d1dwke1 17079

d1dwkf1 17080

d1dwkg1 17081

d1dwkh1 17082

d1dwki1 17083

d1dwkj1 17084

d1dw9a1 17065

d1dw9b1 17066

d1dw9c1 17067

d1dw9d1 17068

d1dw9e1 17069

d1dw9f1 17070

d1dw9g1 17071

d1dw9h1 17072

d1dw9i1 17073

d1dw9j1 17074

d2iu7a1 137649

d2iu7b1 137651

d2iu7c1 137653

d2iu7d1 137655

d2iu7e1 137657

d2iu7f1 137659

d2iu7g1 137661

d2iu7h1 137663

d2iu7i1 137665

d2iu7j1 137667

d2iuoa1 137689

d2iuob1 137691

d2iuoc1 137693

d2iuod1 137695

d2iuoe1 137697

d2iuof1 137699

d2iuog1 137701

d2iuoh1 137703

d2iuoi1 137705

d2iuoj1 137707

d1uxca_ 17119

d1uxda_ 17118

d1rzra1 111989

d1rzrc1 111991

d1rzrd1 111993

d1rzrg1 111995

d1zvva1 125727

d1zvvb1 125729

d1zvvg1 125731

d1efaa1 17106

d1efab1 17107

d1efac1 17108

d2pe5a1 149392

d2pe5b1 149394

d2pe5c1 149396

d1jwla1 67389

d1jwlb1 67391

d1cjga_ 17112

d1cjgb_ 17113

d1lqca_ 17109

d1l1ma_ 73474

d1l1mb_ 73475

d1lcca_ 17110

d1lcda_ 17111

d2bjca1 128620

d2bjcb1 128621

d1osla_ 93497

d1oslb_ 93498

d1lbga1 17114

d1lbgb1 17115

d1lbgc1 17116

d1lbgd1 17117

d1qpza1 17097

d1jfta1 66652

d2puba1 17086

d1vpwa1 17087

d2puda1 17085

d1qqba1 17099

d2puea1 17091

d1zaya1 17090

d1bdha1 17089

d1jfsa1 66650

d2puga1 17088

d2puca1 17094

d1weta1 17093

d1bdia1 17092

d1jh9a1 66710

d1pnra1 17095

d1qqaa1 17101

d1qp4a1 17096

d2puaa1 17098

d1qp7a1 17103

d1qp0a1 17102

d2pufa1 17100

d1prua_ 17104

d1prva_ 17105

d2ox6a1 149053

d2ox6b_ 161493

d2ox6c_ 161494

d2ox6d_ 161495

d1s4ka_ 105254

d1s4kb_ 105255

d1ysea1 123970

d2csfa1 130767

d1wiza_ 114686

d1s7ea2 112045

d1x2la1 121644

d1wh8a_ 114636

d1wh6a_ 114634

d2o4aa_ 148582

d2o49a_ 148581

d1y9qa1 116592

d1zs4a1 125585

d1zs4b_ 125586

d1zs4c_ 125587

d1zs4d_ 125588

d1xwra1 122406

d1xwrb_ 122407

d1xwrc_ 122408

d1xwrd_ 122409

d1zpqa_ 125469

d1zpqb_ 125470

d1zpqc_ 125471

d1zpqd_ 125472

d2auwa1 127339

d2awia1 127435

d2awib1 127437

d2awic1 127439

d2awid1 127441

d2awie1 127443

d2awif1 127445

d2awig1 127447

d2awih1 127449

d2awii1 127451

d2awij1 127453

d2awik1 127455

d2awil1 127457

d2axua1 127500

d2axub1 127502

d2axuc1 127504

d2axud1 127506

d2axue1 127508

d2axuf1 127510

d2axug1 127512

d2axuh1 127514

d2axui1 127516

d2axuj1 127518

d2axuk1 127520

d2axul1 127522

d2aw6a1 127408

d2aw6b1 127410

d2grla1 135560

d2grlb1 135562

d2grlc1 135564

d2grld1 135566

d2axza1 127536

d2axzb1 127538

d2axzc1 127540

d2axzd1 127542

d2grma1 147161

d2grmb1 147163

d2axva1 127524

d2axvb1 127526

d2axvc1 127528

d2axvd1 127530

d1x57a1 121704

d2a6ca1 126236

d2a6cb_ 126237

d2o38a1 148568

d4f8da_ 196769

d4fn3a_ 197160

d4fn3b_ 197153

d3s8qa_ 185316

d3s8qb_ 185317

d3clca_ 173309

d3clcb_ 173310

d3clcc_ 173311

d3clcd_ 173312

d2pija_ 167196

d2pijb_ 167197

d1hq1a_ 17120

d1dula_ 17121

d3lqxa_ 180521

d2pxea_ 149909

d2pxba_ 149907

d2pxda_ 149908

d2pxka_ 149914

d2pxfa_ 149910

d2pxva1 149921

d2pxta_ 149919

d2pxpa_ 149916

d2pxla_ 149915

d2pxua_ 149920

d2pxqa_ 149917

d1qzxa2 96712

d1qzxb2 96715

d1qzwa2 96700

d1qzwc2 96703

d1qzwe2 96706

d1qzwg2 96709

d2ffha2 17122

d2ffhb2 17123

d2ffhc2 17124

d1qb2a_ 17125

d1qb2b_ 17126

d1mfqc_ 79046

d2go5w1 135433

d1k1va_ 71999

d1sknp_ 17127

d1nlwa_ 80633

d1nlwd_ 80635

d1nkpb_ 80574

d1nkpe_ 80576

d1nlwb_ 80634

d1nlwe_ 80636

d1hloa_ 17128

d1hlob_ 17129

d1r05a_ 96723

d1r05b_ 96724

d1an2a_ 17130

d1nkpa_ 80573

d1nkpd_ 80575

d1mdya_ 17132

d1mdyb_ 17133

d1mdyc_ 17134

d1mdyd_ 17135

d1a0aa_ 17138

d1a0ab_ 17139

d1am9a_ 17140

d1am9b_ 17141

d1am9c_ 17142

d1am9d_ 17143

d1uklc_ 99495

d1ukld_ 99496

d1ukle_ 99497

d1uklf_ 99498

d1an4a_ 17136

d1an4b_ 17137

d1pzra_ 95468

d1pzrb_ 95469

d1qx2a_ 104622

d1qx2b_ 104623

d1ig5a_ 62363

d4icba_ 17145

d1ht9a_ 61252

d1ht9b_ 61253

d1igva_ 62369

d3icba_ 17147

d2bcaa_ 17149

d1cdna_ 17148

d1kcya_ 68450

d1clba_ 17153

d1d1oa_ 17152

d2bcba_ 17150

d1b1ga_ 17151

d1ksma_ 68859

d1kqva_ 68842

d1n65a_ 91685

d1boda_ 17154

d1boca_ 17155

d1cb1a_ 17156

d3iqoa_ 178561

d3iqob_ 178562

d3lk1a_ 180336

d3cr5x_ 156942

d3llea_ 180370

d3lleb_ 180371

d3gk4x_ 176717

d1mhoa_ 17171

d3cr2a_ 156936

d3gk2a_ 176716

d3rlza_ 195532

d3rlzb_ 195533

d3cr4x_ 156941

d3iqqa_ 178563

d3gk1a_ 176715

d3lk0a_ 180332

d3lk0b_ 180333

d3lk0c_ 180334

d3lk0d_ 180335

d1psba_ 95079

d1psbb_ 95080

d1cfpa_ 17172

d1cfpb_ 17173

d1e8aa_ 17188

d1e8ab_ 17189

d1odba_ 86840

d1odbb_ 86841

d1odbc_ 86842

d1odbd_ 86843

d1odbe_ 86844

d1odbf_ 86845

d1gqma_ 70360

d1gqmb_ 70361

d1gqmc_ 70362

d1gqmd_ 70363

d1gqme_ 70364

d1gqmf_ 70365

d1gqmg_ 70366

d1gqmh_ 70367

d1gqmi_ 70368

d1gqmj_ 70369

d1gqmk_ 70370

d1gqml_ 70371

d4ggfa_ 196624

d4ggfs_ 196625

d4ggfu_ 196623

d1xk4a1 122055

d1xk4b_ 122056

d1xk4e_ 122059

d1xk4f_ 122060

d1xk4i_ 122063

d1xk4j_ 122064

d1mr8a_ 17186

d1mr8b_ 17187

d1a4pa_ 17176

d1a4pb_ 17177

d1bt6a_ 17178

d1bt6b_ 17179

d1psra_ 17180

d1psrb_ 17181

d2psra_ 17182

d3psra_ 17183

d3psrb_ 17184

d2egdb_ 191828

d1yuta1 124066

d1yutb1 124067

d1yura1 124062

d1yurb1 124063

d1yuua1 124068

d1yuub1 124069

d1yusa1 124064

d1yusb1 124065

d1ksoa_ 72926

d1ksob_ 72927

d2q91a_ 150135

d2q91b_ 150136

d3ko0a_ 179479

d3ko0b_ 179480

d3ko0c_ 179481

d3ko0d_ 179482

d3ko0e_ 179483

d3ko0f_ 179484

d3ko0g_ 179485

d3ko0h_ 179486

d3ko0i_ 179487

d3ko0j_ 179488

d3ko0k_ 179489

d3ko0l_ 179490

d3ko0m_ 179491

d3ko0n_ 179492

d3ko0o_ 179493

d3ko0p_ 179494

d3ko0q_ 179495

d3ko0r_ 179496

d3ko0s_ 179497

d3ko0t_ 179498

d4hsza_ 194057

d4hszb_ 194056

d4hszc_ 194055

d4hszd_ 194058

d3cgaa_ 156615

d3cgab_ 156616

d3m0wa_ 180690

d3m0wb_ 180691

d3m0wc_ 180692

d3m0wd_ 180693

d3m0we_ 180694

d3m0wf_ 180695

d3m0wg_ 180696

d3m0wh_ 180697

d3m0wi_ 180698

d3m0wj_ 180699

d1m31a_ 78506

d1m31b_ 78507

d1k8ua_ 72181

d1k96a_ 72187

d1k9ka_ 72206

d1k9kb_ 72207

d1k9pa_ 72238

d1irja_ 66289

d1irjb_ 66290

d1irjc_ 66291

d1irjd_ 66292

d1irje_ 66293

d1irjf_ 66294

d1irjg_ 66295

d1irjh_ 66296

d3cztx_ 173564

d3d0ya_ 173606

d3d0yb_ 173607

d3d10a_ 173608

d3d10b_ 173609

d2prua1 149815

d2prub1 149816

d1uwoa_ 17174

d1uwob_ 17175

d1mq1a_ 79392

d1mq1b_ 79393

d1j55a_ 77078

d1ozoa_ 93851

d1ozob_ 93852

d2k2fa1 148258

d2k2fb1 148259

d1zfsa1 125003

d1zfsb1 125004

d1k2ha_ 68056

d1k2hb_ 68057

d1qlka_ 17163

d1qlkb_ 17164

d1mwna_ 79584

d1mwnb_ 79585

d1dt7a_ 17165

d1dt7b_ 17166

d1b4ca_ 17169

d1b4cb_ 17170

d1xyda1 122457

d1xydb1 122458

d1syma_ 17167

d1symb_ 17168

d1qlsa_ 17185

d1a03a_ 17157

d1a03b_ 17158

d2jtta1 148205

d2jttb1 148206

d1jwda_ 71908

d1jwdb_ 71909

d2cnpa_ 17159

d2cnpb_ 17160

d1cnpa_ 17161

d1cnpb_ 17162

d1nsha_ 86135

d1nshb_ 86136

d4fqoa_ 193460

d4duqa_ 192349

d4duqb_ 192350

d3nsla_ 182514

d3nslb_ 182515

d3nslc_ 182516

d3nsld_ 182517

d3nsle_ 182518

d3nslf_ 182519

d3nsoa1 182520

d3nsob1 182521

d3c1va_ 155867

d3c1vb_ 155868

d3c1vc_ 155869

d3c1vd_ 155870

d4aqja_ 194528

d3nska1 182512

d3nskb1 182513

d2rgia_ 168116

d2rgib_ 168117

d2wnda_ 169476

d2wora_ 169530

d2wosa_ 169531

d4aqia_ 194527

d2wcea_ 169214

d2wceb_ 169215

d3zwha_ 195645

d3zwhb_ 195646

d2wc8a_ 169208

d2wc8b_ 169209

d2wc8c_ 169210

d2wc8d_ 169211

d2h61a_ 136169

d2h61b_ 136170

d2h61c_ 136171

d2h61d_ 136172

d2h61e_ 136173

d2h61f_ 136174

d2h61g_ 136175

d2h61h_ 136176

d2wcba_ 169212

d2wcbb_ 169213

d3nsia_ 182510

d3nsib_ 182511

d3hcma_ 177384

d3hcmb_ 177385

d2wcfa_ 169216

d2wcfb_ 169217

d2wcfc_ 169218

d2wcfd_ 169219

d2wcfe_ 169220

d2wcff_ 169221

d2y5ia_ 192787

d2y5ib_ 192788

d2y5ic_ 192789

d2y5id_ 170616

d2y5ie_ 170617

d2y5if_ 192786

d1sraa_ 17190

d1nuba1 17191

d1nubb1 17192

d1bmoa1 17193

d1bmob1 17194

d1rroa_ 17195

d1omda_ 17196

d2nlna1 148283

d1cdpa_ 17197

d4cpva_ 17198

d1b8la_ 17199

d5cpva_ 17200

d1b8ra_ 17201

d1b8ca_ 17202

d1b8cb_ 17203

d1b9aa_ 17204

d3fs7a_ 176014

d3fs7b_ 176015

d3fs7c_ 176016

d3fs7d_ 176017

d3fs7e_ 176018

d3fs7f_ 176019

d3fs7g_ 176020

d3fs7h_ 176021

d1rk9a_ 104965

d1rjva_ 104964

d5pala_ 17215

d1rwya_ 98002

d1rwyb_ 98003

d1rwyc_ 98004

d1g33a_ 65121

d3f45a_ 175449

d1xvja_ 162111

d1xvjb_ 162112

d1s3pa_ 105249

d2jwwa1 148231

d2pvba_ 17205

d1pvba_ 17206

d1pvaa_ 17207

d1pvab_ 17208

d2pala_ 17211

d1pala_ 17209

d4pala_ 17210

d3pala_ 17212

d3pata_ 17214

d2pasa_ 17213

d1rtp1_ 17219

d1rtp2_ 17220

d1rtp3_ 17221

d1bu3a_ 17218

d1a75a_ 17216

d1a75b_ 17217

d2zfda1 154406

d1uhna_ 99399

d1tcob_ 17324

d4f0zb_ 196640

d1auib_ 17325

d2p6bb1 139512

d2p6bd1 139514

d1m63b_ 78678

d1m63f_ 78681

d1mf8b_ 79041

d1c7wa_ 17261

d1c7va_ 17262

d1j7qa_ 62700

d1j7ra_ 62701

d1xo5a_ 115680

d1xo5b_ 115681

d1dgua_ 17333

d1dgva_ 17334

d1s6ia_ 105310

d1s6ja_ 112042

d1jf0a_ 62926

d1qv1a_ 96347

d1qv0a_ 96346

d1sl9a_ 118971

d1el4a_ 59453

d1jf2a_ 62930

d2f8pa_ 164281

d1s36a_ 112009

d1sl7a_ 112098

d1ej3a_ 17259

d1ej3b_ 17260

d1sl8a_ 112099

d1nyaa_ 92335

d1iq5a_ 62643

d1f70a_ 17288

d1sy9a1 145775

d1f71a_ 17289

d2k3sb1 148267

d1cfca_ 17290

d1nwda_ 86297

d1cfda_ 17291

d1muxa_ 17292

d1x02a1 145839

d1ckka_ 17294

d1dmoa_ 17293

d1y0vh1 145893

d1y0vi1 145894

d1y0vj1 145895

d1y0vk1 145896

d1y0vl1 145897

d1y0vm1 145898

d1cffa_ 17295

d2fcea1 133266

d1lkja_ 84623

d1f54a_ 83244

d1f55a_ 83245

d2o5ga_ 148598

d3gp2a_ 176796

d2o60a_ 148619

d1ahra_ 17287

d1up5a_ 119696

d1up5b_ 119697

d2bcxa_ 146134

d2bkib_ 146153

d2bkid_ 161391

d1exra_ 17299

d1osaa_ 17300

d1n0ya_ 91536

d1n0yb_ 91537

d1clma_ 17301

d3if7a_ 178292

d1prwa_ 95062

d1fw4a_ 60056

d1lina_ 17265

d1cdma_ 17270

d1cm1a_ 17271

d1xa5a_ 115029

d1a29a_ 17276

d1qiwa_ 17277

d1qiwb_ 17278

d1qiva_ 17279

d2fota_ 147041

d1ak8a_ 17281

d1cmga_ 17282

d1cmfa_ 17283

d1dega_ 17280

d1mxea_ 79644

d1mxeb_ 79645

d4dbpc_ 194063

d3l9ic_ 180115

d2x51b_ 169880

d4dbqb_ 194062

d2bkhb_ 146152

d2vasb_ 152830

d4clna_ 17296

d3gn4b_ 176770

d3gn4d_ 176771

d3gn4f_ 176772

d3gn4h_ 176773

d2bbma_ 17297

d2bbna_ 17298

d4djca_ 196007

d2f3ya_ 146994

d2w73a_ 169087

d2w73b_ 169088

d2w73e_ 169089

d2w73f_ 169090

d2f3za_ 146995

d1yr5a_ 145922

d2weld_ 169276

d3byaa_ 172928

d2obha_ 148716

d2obhb_ 148717

d1clla_ 17263

d1zuza_ 146028

d2y4va_ 170603

d2be6a_ 146136

d2be6b_ 146137

d2be6c_ 146138

d2vaya_ 152870

d2r28a_ 151531

d2r28b_ 151532

d1iwqa_ 83761

d3g43a_ 176330

d3g43b_ 176331

d3g43c_ 176332

d3g43d_ 176333

d1cdla_ 17272

d1cdlb_ 17273

d1cdlc_ 17274

d1cdld_ 17275

d2v01a_ 152360

d1wrza_ 145833

d2x0gb_ 169753

d3dvea_ 174278

d2ggma1 135152

d2ggmb1 135153

d2v02a_ 152361

d1l7za_ 91054

d3dvka_ 174284

d1y6wa_ 145912

d3hr4b_ 177788

d3hr4d_ 177789

d3hr4f_ 177790

d3hr4h_ 177791

d3oxqa_ 183380

d3oxqb_ 183381

d3oxqc_ 183382

d3oxqd_ 183383

d4gowd_ 193072

d3dvja_ 174283

d1k90d_ 68322

d1k90e_ 68323

d1k90f_ 68324

d1ctra_ 17264

d3dvma_ 174285

d1s26d_ 98370

d1s26e_ 98371

d1s26f_ 98372

d1k93d_ 68330

d1k93e_ 68331

d1k93f_ 68332

d1pk0d_ 94798

d1pk0e_ 94799

d1pk0f_ 94800

d1sk6d_ 105668

d1sk6e_ 105669

d1sk6f_ 105670

d1xfxo1 121955

d1xfxp1 121956

d1xfxq1 121957

d1xfxr1 121958

d1xfxs1 121959

d1xfxt1 121960

d1xfzo1 121967

d1xfzp1 121968

d1xfzq1 121969

d1xfzr1 121970

d1xfzs1 121971

d1xfzt1 121972

d1xfyo1 121961

d1xfyp1 121962

d1xfyq1 121963

d1xfyr1 121964

d1xfys1 121965

d1xfyt1 121966

d1xfwo1 121949

d1xfwp1 121950

d1xfwq1 121951

d1xfwr1 121952

d1xfws1 121953

d1xfwt1 121954

d1xfvo1 121943

d1xfvp1 121944

d1xfvq1 121945

d1xfvr1 121946

d1xfvs1 121947

d1xfvt1 121948

d1j7pa_ 66416

d1j7oa_ 66415

d1lvcd_ 78239

d1lvce_ 78240

d1lvcf_ 78241

d1xfuo1 121937

d1xfup1 121938

d1xfuq1 121939

d1xfur1 121940

d1xfus1 121941

d1xfut1 121942

d1sw8a_ 99022

d2k0ea1 148254

d2k0fa1 148255

d4hexa_ 193914

d4hexb_ 193913

d2dfsb1 146510

d2dfsc1 146511

d2dfsd1 146512

d2dfse1 146513

d2dfsf1 146514

d2dfsg1 146515

d2dfsn1 146516

d2dfso1 146517

d2dfsp1 146518

d2dfsq1 146519

d2dfsr1 146520

d2dfss1 146521

d1ooja_ 87198

d4j9yr_ 196730

d1g4yr_ 60252

d2hqwa_ 147364

d2yggb_ 170785

d1niwa_ 80537

d1niwc_ 80538

d1niwe_ 80539

d1niwg_ 80540

d3bxla_ 155718

d3bxka_ 155716

d3bxkc_ 155717

d1qx5b_ 104628

d1qx5d_ 104629

d1qx5i_ 104630

d1qx5j_ 104631

d1qx5k_ 104632

d1qx5r_ 104633

d1qx5t_ 104634

d1qx5y_ 104635

d1qx7a_ 104636

d1qx7b_ 104637

d1qx7i_ 104638

d1qx7m_ 104639

d1qx7r_ 104640

d1rfja_ 104918

d3clna_ 17286

d1ggza_ 70176

d1tiza_ 107014

d1oqpa_ 87319

d1m39a_ 84782

d1ggwa_ 60490

d2nxqa_ 166443

d2nxqb_ 166444

d1jfka_ 66639

d1jfja_ 66638

d1fpwa_ 17329

d2ju0a1 148208

d1g8ia_ 60363

d1g8ib_ 60364

d1jbaa_ 17330

d1s1ea_ 112007

d1s6ca_ 98594

d1wdcb_ 17302

d1kk8b_ 77430

d1qviy_ 96429

d1b7ty_ 17303

d1scmb_ 17304

d1sr6b_ 105955

d1s5gy_ 105265

d1l2ob_ 77660

d1kk7y_ 77426

d1kqmb_ 77490

d1kwob_ 77571

d1dflw_ 17306

d1dfly_ 17307

d1dfky_ 17305

d2mysb_ 17308

d1br1b_ 17309

d1br1d_ 17310

d1br1f_ 17311

d1br1h_ 17312

d1br4b_ 17313

d1br4d_ 17314

d1br4f_ 17315

d1br4h_ 17316

d3dtpc1 157854

d3dtpd1 157855

d1oe9b_ 92794

d1w7ib1 120687

d1m45a_ 78598

d1n2da_ 91557

d1n2db_ 91558

d1m46a_ 78599

d1wdcc_ 17317

d3jvtc_ 178849

d1kk8c_ 77431

d1qviz_ 96430

d3jtdc_ 178822

d1b7tz_ 17318

d1scmc_ 17319

d1sr6c_ 105956

d1s5gz_ 105266

d1l2oc_ 77661

d1kk7z_ 77427

d1kqmc_ 77491

d1kwoc_ 77572

d1dflx_ 17321

d1dflz_ 17322

d1dfkz_ 17320

d2mysc_ 17323

d1bjfa_ 17331

d1bjfb_ 17332

d1omra_ 93349

d1reca_ 17326

d1omva_ 93353

d2heta1 136356

d2hetb1 136357

d2hetc1 136358

d2hetd1 136359

d1ikua_ 17327

d2i94a1 137109

d1la3a_ 73781

d1jsaa_ 17328

d2sasa_ 17258

d2scpa_ 17256

d2scpb_ 17257

d1q80a_ 104553

d1topa_ 17223

d1ncxa_ 17222

d1ncza_ 17224

d1ncya_ 17225

d1avsa_ 17226

d1avsb_ 17227

d4tnca_ 17228

d1dtla_ 17229

d1ytzc1 124022

d1ctda_ 17230

d1ctdb_ 17231

d1ctaa_ 17232

d1ctab_ 17233

d1zaca_ 17236

d1tnxa_ 17237

d1tnwa_ 17235

d1sbja_ 112064

d1scva_ 112072

d1smga_ 17234

d1jc2a_ 62862

d1la0a_ 77864

d3ctna_ 17241

d1aj4a_ 17244

d1pon.1 17239

d1skta_ 17243

d2ctna_ 17242

d1npqa_ 85983

d1tnpa_ 17238

d1tnqa_ 17245

d1blqa_ 17240

d1yv0c1 124081

d1fi5a_ 17252

d4gjga_ 193067

d1j1da_ 83963

d1j1dd_ 83966

d1j1ea_ 83969

d1j1ed_ 83972

d2hf5a1 147275

d1ap4a_ 17253

d2jt3a1 148197

d1mxlc_ 17255

d2jt0a1 148194

d1spya_ 17254

d1ih0a_ 66140

d2jtza1 148207

d2jt8a1 148201

d1lxfc_ 78292

d1ozsa_ 93857

d1tn4a_ 17248

d2tn4a_ 17249

d1tcfa_ 17250

d1a2xa_ 17251

d1r2ua_ 104783

d1r6pa_ 104822

d5tnca_ 17246

d1trfa_ 17247

d3fwba_ 176100

d3fwca_ 176101

d3fwce_ 176102

d3fwci_ 176103

d3fwcm_ 176104

d3jvtb_ 178848

d3jtdb_ 178821

d2vb6b_ 152872

d1w7jb_ 120690

d2d8na_ 163607

d1y1aa_ 122537

d1y1ab_ 122538

d3ewta_ 175283

d3ewva_ 175284

d1uhka_ 119681

d1uhkb_ 119682

d1uhha_ 119675

d1uhhb_ 119676

d1uhja_ 119679

d1uhjb_ 119680

d1uhia_ 119677

d1uhib_ 119678

d4ds7a_ 195893

d4ds7b_ 195892

d4ds7c_ 195891

d4ds7d_ 195894

d2ix7a_ 147826

d2ix7b_ 147827

d3pn7b_ 183858

d3pn7c_ 183859

d3pn7e_ 183860

d3pn7f_ 183861

d3ts5b_ 185926

d3ts5c_ 185927

d3ts5e_ 185928

d3ts5f_ 185929

d3tuyb_ 185960

d3tuyc_ 185961

d3tuye_ 185962

d3tuyf_ 185963

d3kf9a_ 179324

d3kf9c_ 191747

d4aqra_ 194526

d4aqrb_ 194525

d3i5gc_ 178095

d2jxca1 148234

d1ff1a_ 17338

d1eh2a_ 17339

d1f8ha_ 17336

d1c07a_ 17337

d1qjta_ 17335

d1iq3a_ 62640

d1fi6a_ 59849

d1h8ba_ 60736

d3buxb1 155647

d3buxd1 155650

d3buwb1 155641

d3buwd1 155644

d3bunb1 155626

d1yvha1 124096

d2cbla1 17380

d3bumb1 155623

d1b47a1 17381

d1b47b1 17382

d1b47c1 17383

d3buob1 155629

d3buod1 155632

d1fbva1 17384

d1tuza_ 112670

d1wlza1 121024

d1eg3a1 17376

d1eg3a2 17377

d1eg4a1 17378

d1eg4a2 17379

d1snla_ 105820

d1qasa1 17340

d1qasb1 17341

d1djxa1 17342

d1djxb1 17343

d1djwa1 17344

d1djwb1 17345

d1djha1 17346

d1djhb1 17347

d1djia1 17348

d1djib1 17349

d1djga1 17350

d1djgb1 17351

d2isda1 17352

d2isdb1 17353

d1djya1 17356

d1djyb1 17357

d1djza1 17358

d1djzb1 17359

d1qata1 17354

d1qatb1 17355

d2zkmx1 154587

d2fjub1 145170

d1wlzb_ 121025

d1wlzc_ 121026

d1wlzd_ 121027

d1hqva_ 61160

d1kful1 68571

d1kfxl1 68575

d1df0a1 17375

d1u5ia1 119547

d1qxpa2 96544

d1qxpb2 96548

d1kfus_ 68574

d1kfxs_ 68578

d1np8a_ 92022

d1np8b_ 92023

d1dvia_ 17365

d1dvib_ 17366

d1aj5a_ 17367

d1aj5b_ 17368

d1df0b_ 17369

d1qxpa1 96543

d1qxpb1 96547

d1alva_ 17370

d1alvb_ 17371

d1nx1a_ 92279

d1nx1b_ 92280

d1alwa_ 17372

d1alwb_ 17373

d1nx2a_ 92281

d1nx3a_ 92282

d1nx0a_ 92277

d1nx0b_ 92278

d1k94a_ 68333

d1k94b_ 68334

d1k95a_ 68335

d1f4qa_ 17360

d1f4qb_ 17361

d1f4oa_ 17362

d1f4ob_ 17363

d1y1xa_ 116374

d1y1xb_ 116375

d1gjya_ 70199

d1gjyb_ 70200

d1gjyc_ 70201

d1gjyd_ 70202

d1juoa_ 67312

d1juob_ 67313

d1ij5a_ 76748

d1ij6a_ 76749

d1h4ba_ 90608

d2opoa_ 139209

d2opob_ 139210

d2opoc_ 139211

d2opod_ 139212

d1k9ua_ 84348

d1k9ub_ 84349

d1pula1 118730

d1wlma1 121008

d3nxaa_ 182604

d3nxab_ 182605

d3nxac_ 182606

d3nxad_ 182607

d2ccma_ 163372

d2ccmb_ 163373

d2hpsa_ 165203

d2hq8a_ 165206

d2hq8b_ 165207

d2bl0c_ 163120

d3i5gb_ 178094

d2jpoa1 148166

d1qwva_ 96506

d1twoa1 119368

d1dqea_ 17388

d1dqeb_ 17389

d2fjya_ 133626

d2fjyb_ 133627

d1gm0a_ 65297

d1ls8a_ 78176

d1ooha_ 93383

d1oohb_ 93384

d2gtea_ 135646

d2gteb_ 135647

d1ooga_ 93381

d1oogb_ 93382

d1oofa_ 93379

d1oofb_ 93380

d1t14a_ 119100

d1t14b_ 119101

d3b6xa_ 172449

d3b6xb_ 172450

d3b7aa_ 172454

d3b7ab_ 172455

d3b87a_ 172489

d3b87b_ 172490

d3b88a_ 172491

d3b88b_ 172492

d1ooix_ 93385

d3b86a_ 172487

d3b86b_ 172488

d2qdia_ 167539

d2qdib_ 167540

d1p28a_ 93911

d1p28b_ 93912

d1ow4a_ 93628

d1ow4b_ 93629

d1orga_ 93453

d1orgb_ 93454

d3cz1a_ 173556

d3cz1b_ 173557

d3cz0a_ 173554

d3cz0b_ 173555

d3bjha_ 172661

d3cyza_ 173552

d3cyzb_ 173553

d3fe9a_ 175725

d3fe6a_ 175722

d3fe8a_ 175724

d3caba_ 173097

d3bfha_ 172591

d3cdna_ 173155

d3bfaa_ 172580

d3bfba_ 172581

d3cz2a_ 173558

d3cz2b_ 173559

d2h8va_ 165014

d1c3za_ 17386

d1c3ya_ 17387

d4inwa_ 193911

d4inxa_ 193912

d3d78a_ 173738

d3d78b_ 173739

d3d77a_ 173737

d3d73a_ 173731

d3d73b_ 173732

d3d76a_ 173736

d3d74a_ 173733

d3d74b_ 173734

d3d75a_ 173735

d2p70a_ 139515

d2p71a_ 139516

d4f7fd_ 193462

d3q8ia_ 196275

d4fqtb_ 193181

d3ognb_ 193718

d2wcja_ 169225

d2wcma_ 169228

d2wcla_ 169227

d2wcka_ 169226

d2wcha_ 169222

d2wc6a_ 169207

d2wc5a_ 169206

d3k1ea_ 178947

d3k1eb_ 178948

d2ciwa1 130502

d2ciwa2 130503

d2ciza1 130508

d2ciza2 130509

d2cj2a1 130514

d2cj2a2 130515

d2ciya1 130506

d2ciya2 130507

d2j18a1 137937

d2j18a2 137938

d2cj0a1 130510

d2cj0a2 130511

d2cj1a1 130512

d2cj1a2 130513

d2civa1 130500

d2civa2 130501

d2j19a1 137939

d2j19a2 137940

d2j5ma1 138047

d2j5ma2 138048

d2cixa1 130504

d2cixa2 130505

d1cpoa1 17390

d1cpoa2 17391

d2cpoa1 17392

d2cpoa2 17393

d1iioa_ 66150

d1sh5a1 105543

d1sh5a2 105544

d1sh5b1 105545

d1sh5b2 105546

d1sh6a1 105547

d1sh6a2 105548

d1mb8a1 84925

d1mb8a2 84926

d1bkra_ 17394

d1aa2a_ 17395

d1h67a_ 65643

d1dxxa1 17404

d1dxxa2 17405

d1dxxb1 17406

d1dxxb2 17407

d1dxxc1 17408

d1dxxc2 17409

d1dxxd1 17410

d1dxxd2 17411

d1rt8a_ 105095

d1aoaa1 17396

d1aoaa2 17397

d1wjoa_ 114704

d1pxya_ 104384

d1pxyb_ 104385

d2r8ua1 151743

d2r8ub1 151744

d1pa7a_ 94410

d1vkaa_ 108641

d1vkab_ 108642

d1uega_ 99258

d1v5ka_ 108377

d1p5sa_ 94147

d1p2xa_ 104062

d1ujoa_ 107900

d1bhda_ 17398

d1bhdb_ 17399

d1qaga1 17400

d1qaga2 17401

d1qagb1 17402

d1qagb2 17403

d3co1a_ 173362

d1lnsa1 78101

d1wixa_ 114683

d1a26a1 17412

d1efya1 17413

d2paxa1 17414

d1paxa1 17416

d3paxa1 17415

d2pawa1 17417

d4paxa1 17418

d2rcwa1 151911

d1woka1 121116

d1wokb1 121118

d1wokc1 121120

d1wokd1 121122

d1uk1a1 107901

d1uk1b1 107903

d1uk0a1 99477

d1uk0b1 99479

d1gs0a1 76323

d1gs0b1 76325

d1v32a_ 100277

d1v31a_ 100276

d1ycqa_ 17419

d1ttva_ 112637

d2axia1 127494

d3lbla_ 180164

d3lblc_ 180165

d3lble_ 180166

d3tu1a_ 185951

d2gv2a_ 135756

d3jzsa_ 178936

d4dija_ 195473

d4dijb_ 195472

d3v3ba_ 186458

d3v3bb_ 186459

d1t4fm_ 119144

d3g03a_ 176230

d3g03c_ 176231

d3w69a_ 197281

d4jvra_ 196976

d4hbme_ 193444

d4erea_ 192401

d4ereb_ 192402

d3jzra_ 178935

d4erfa_ 192403

d4erfc_ 192404

d4erfe_ 192405

d3tj2a_ 192477

d3tj2c_ 192476

d4jv7a_ 197256

d3lbka_ 180163

d4jwra_ 197258

d4jwrb_ 196977

d1rv1a_ 97901

d1rv1b_ 97902

d1rv1c_ 97903

d1ycra_ 17420

d3jzka_ 178930

d1t4ea_ 119142

d1t4eb_ 119143

d3vbga_ 195030

d3vbgc_ 195029

d3vbgd_ 195031

d3vzvb_ 193259

d1uhra_ 107852

d3feaa_ 175726

d3fe7a_ 175723

d3fdoa_ 175715

d3lbje_ 180162

d3jzpa_ 178932

d3jzoa_ 178931

d3jzqa_ 178933

d3jzqb_ 178934

d3daba_ 173782

d3dabc_ 173783

d3dabe_ 173784

d3dabg_ 173785

d2vyra_ 168933

d2vyrb_ 168934

d2vyrc_ 168935

d2vyrd_ 168936

d3u15c_ 195034

d1baza_ 17421

d1bazb_ 17422

d1bazc_ 17423

d1bazd_ 17424

d1u9pa1 119649

d1myka_ 17425

d1mykb_ 17426

d1myla_ 17427

d1mylb_ 17428

d1mylc_ 17429

d1myld_ 17430

d1myle_ 17431

d1mylf_ 17432

d1bdta_ 17433

d1bdtb_ 17434

d1bdtc_ 17435

d1bdtd_ 17436

d1para_ 17437

d1parb_ 17438

d1parc_ 17439

d1pard_ 17440

d1bdva_ 17441

d1bdvb_ 17442

d1bdvc_ 17443

d1bdvd_ 17444

d1arqa_ 17447

d1arqb_ 17448

d1arra_ 17449

d1arrb_ 17450

d1qtga_ 17451

d1qtgb_ 17452

d1b28a_ 17445

d1b28b_ 17446

d1nlaa_ 85848

d1nlab_ 85849

d1mnta_ 17453

d1mntb_ 17454

d2hzaa1 136907

d2hzab1 136909

d1q5va1 95937

d1q5vb1 95939

d1q5vc1 95941

d1q5vd1 95943

d2hzva1 136916

d2hzvb1 136918

d2hzvc1 136920

d2hzvd1 136922

d2hzve1 136924

d2hzvf1 136926

d2hzvg1 136928

d2hzvh1 136930

d2bj7a1 128608

d2bj7b1 128610

d2bj8a1 128612

d2bj8b1 128614

d2bj3a1 128600

d2bj3b1 128602

d2bj3c1 128604

d2bj3d1 128606

d2bj9a1 128616

d2bj9b1 128618

d2bj1a1 128596

d2bj1b1 128598

d1p94a_ 94380

d1p94b_ 94381

d2cpga_ 17455

d2cpgb_ 17456

d2cpgc_ 17457

d1b01a_ 17458

d1b01b_ 17459

d1ea4a_ 64861

d1ea4b_ 64862

d1ea4d_ 64863

d1ea4e_ 64864

d1ea4f_ 64865

d1ea4g_ 64866

d1ea4h_ 64867

d1ea4j_ 64868

d1ea4k_ 64869

d1ea4l_ 64870

d1x93a1 145859

d1x93b1 145860

d1irqa_ 66297

d1irqb_ 66298

d2bnwa_ 128857

d2bnwb_ 128858

d2bnwc_ 128859

d2bnwd_ 128860

d2bnza_ 128863

d2bnzb_ 128864

d2bnzc_ 128865

d2bnzd_ 128866

d2caxa_ 130163

d2caxb_ 130164

d2caxc_ 130165

d2caxd_ 130166

d1cmca_ 17462

d1cmcb_ 17463

d1cmba_ 17460

d1cmbb_ 17461

d1mjka_ 17464

d1mjkb_ 17465

d1mjla_ 17470

d1mjlb_ 17471

d1mjoa_ 17466

d1mjob_ 17467

d1mjoc_ 17468

d1mjod_ 17469

d1mjma_ 17472

d1mjmb_ 17473

d1mj2a_ 17474

d1mj2b_ 17475

d1mj2c_ 17476

d1mj2d_ 17477

d1mjqa_ 17478

d1mjqb_ 17479

d1mjqc_ 17480

d1mjqd_ 17481

d1mjqg_ 17482

d1mjqh_ 17483

d1mjqi_ 17484

d1mjqj_ 17485

d1cmaa_ 17486

d1cmab_ 17487

d1mjpa_ 17488

d1mjpb_ 17489

d1zx3a1 125762

d1xrxa1 122264

d1xrxb_ 122265

d1xrxc_ 122266

d1xrxd_ 122267

d2h1oe1 145278

d2h1of1 145279

d2h1og1 145280

d2h1oh1 145281

d2bsqe1 129102

d2bsqf1 129103

d2bsqg1 129104

d2bsqh1 129105

d1y9ba1 122777

d1y9bb_ 122778

d2rh3a1 152027

d2ay0a1 146077

d2ay0b_ 146078

d2ay0c_ 146079

d2ay0d_ 146080

d2ay0e_ 146081

d2ay0f_ 146082

d2rbfa_ 168044

d2rbfb_ 168045

d2gpea_ 164790

d2gpeb_ 164791

d2gpec_ 164792

d2gped_ 164793

d2efva1 146819

d1k0ma1 67949

d1k0mb1 67951

d1rk4a1 97592

d1rk4b1 97594

d1k0oa1 67957

d1k0ob1 67959

d1k0na1 67953

d1k0nb1 67955

d1oe8a1 86905

d1oe8b1 86907

d1oe7a1 86901

d1oe7b1 86903

d2f8fa1 133122

d2f8fb1 133124

d2fhea1 17724

d2fheb1 17725

d1fhea1 17726

d1k3ya1 77245

d1k3yb1 77247

d1k3la1 77229

d1k3lb1 77231

d1pl1a1 104178

d1pl1b1 104180

d1pl2a1 104182

d1pl2b1 104184

d1xwga1 122400

d1xwgb1 122402

d2r3xa1 151561

d2r3xb1 151563

d1pkwa1 104170

d1pkwb1 104172

d1ydka1 122997

d1ydkb1 122999

d1k3oa1 77233

d1k3ob1 77235

d1usba1 108001

d1usbb1 108003

d1pkza1 104174

d1pkzb1 104176

d1gsda1 17669

d1gsdb1 17670

d1gsdc1 118474

d1gsdd1 118476

d1gsea1 17667

d1gseb1 17668

d2r6ka1 151614

d2r6kb1 151616

d1guha1 17671

d1guhb1 17672

d1guhc1 118482

d1guhd1 118484

d1gsfa1 17673

d1gsfb1 17674

d1gsfc1 118478

d1gsfd1 118480

d1gula1 17675

d1gulb1 17676

d1gulc1 17677

d1guld1 17678

d1gule1 17679

d1gulf1 17680

d1gulg1 17681

d1gulh1 17682

d1guma1 17683

d1gumb1 17684

d1gumc1 17685

d1gumd1 17686

d1gume1 17687

d1gumf1 17688

d1gumg1 17689

d1gumh1 17690

d1agsa1 17691

d1agsb1 17692

d1f3ba1 17699

d1f3bb1 17700

d1f3aa1 17701

d1f3ab1 17702

d1b48a1 17703

d1b48b1 17704

d1guka1 17705

d1gukb1 17706

d1ml6a1 85009

d1ml6b1 85011

d1ev4a1 17693

d1ev4c1 17694

d1ev4d1 17695

d1ev9a1 17696

d1ev9c1 17697

d1ev9d1 17698

d1duga1 17718

d1dugb1 17719

d1m9aa1 84882

d1m99a1 84880

d1ua5a1 107757

d1gtaa1 17720

d1m9ba1 84884

d1gnea1 17721

d1gtba1 17722

d1y6ea1 145908

d1y6eb1 145910

d1bg5a1 17723

d1u87a1 145778

d1u88a1 145780

d1u88b1 145782

d1n2aa1 91553

d1n2ab1 91555

d1a0fa1 17737

d1a0fb1 17738

d1b8xa1 17739

d2pmta1 17741

d2pmtb1 17742

d2pmtc1 17743

d2pmtd1 17744

d1pmta1 17740

d1f2ea1 17745

d1f2eb1 17746

d1f2ec1 17747

d1f2ed1 17748

d1pn9a1 94949

d1pn9b1 94951

d1jlva1 71729

d1jlvb1 71731

d1jlvc1 71733

d1jlvd1 71735

d1jlve1 71737

d1jlvf1 71739

d1jlwa1 71741

d1jlwb1 71743

d1r5aa1 97081

d1v2aa1 100263

d1v2ab1 100265

d1v2ac1 100267

d1v2ad1 100269

d1gsua1 17661

d1gsub1 17662

d1c72a1 17663

d1c72b1 17664

d1c72c1 17665

d1c72d1 17666

d2c4ja1 129816

d2c4jb1 129818

d2c4jc1 129820

d2c4jd1 129822

d1xw5a1 116099

d1xw5b1 116101

d1xw6a1 116103

d1xw6b1 116105

d1xw6c1 116107

d1xw6d1 116109

d1hnaa1 17604

d1ykca1 123509

d1ykcb1 123511

d1xwka1 116126

d1xwkb1 116128

d1xwkc1 116130

d1yj6a1 123385

d1yj6b1 123387

d1yj6c1 123389

d2gtua1 17605

d2gtub1 17606

d2ab6a1 126507

d2ab6b1 126509

d2ab6c1 126511

d2ab6d1 126513

d1gtua1 17607

d1gtub1 17608

d1gtuc1 17609

d1gtud1 17610

d3gtua1 17611

d3gtub1 17612

d3gtuc1 17613

d3gtud1 17614

d2f3ma1 132873

d2f3mb1 132875

d2f3mc1 132877

d2f3md1 132879

d2f3me1 132881

d2f3mf1 132883

d1hnca1 17615

d1hncb1 17616

d1hncc1 17617

d1hncd1 17618

d1hnba1 17619

d1hnbb1 17620

d4gtua1 17621

d4gtub1 17622

d4gtuc1 17623

d4gtud1 17624

d4gtue1 17625

d4gtuf1 17626

d4gtug1 17627

d4gtuh1 17628

d2gsta1 17629

d2gstb1 17630

d6gswa1 17631

d6gswb1 17632

d6gsva1 17633

d6gsvb1 17634

d6gsua1 17635

d6gsub1 17636

d6gsxa1 17641

d6gsxb1 17642

d3gsta1 17637

d3gstb1 17638

d4gsta1 17639

d4gstb1 17640

d6gsta1 17645

d6gstb1 17646

d5gsta1 17643

d5gstb1 17644

d5fwga1 17647

d5fwgb1 17648

d6gsya1 17649

d6gsyb1 17650

d3fyga1 17651

d3fygb1 17652

d1mtca1 85103

d1mtcb1 85105

d1gsba1 17653

d1gsbb1 17654

d1gsbc1 17655

d1gsbd1 17656

d1gsca1 17657

d1gscb1 17658

d1gscc1 17659

d1gscd1 17660

d1b4pa1 83158

d1eema1 17717

d1axda1 17730

d1axdb1 17731

d1byea1 17732

d1byeb1 17733

d1byec1 17734

d1byed1 17735

d1aw9a1 17736

d1gnwa1 17727

d1gnwb1 17728

d1bx9a1 17729

d3csia1 156965

d3csib1 156967

d3csic1 156969

d3csid1 156971

d1pgta1 17515

d1pgtb1 17516

d1lbka1 73809

d1lbkb1 73811

d17gsa1 17524

d17gsb1 17525

d1aqva1 17532

d1aqvb1 17533

d18gsa1 17528

d18gsb1 17529

d2pgta1 17526

d2pgtb1 17527

d1px7a1 88337

d1px7b1 88339

d2gssa1 17540

d2gssb1 17541

d22gsa1 17542

d22gsb1 17543

d9gssa1 17536

d9gssb1 17537

d1md3a1 74628

d1md3b1 74630

d3pgta1 17550

d3pgtb1 17551

d16gsa1 17548

d16gsb1 17549

d1px6a1 88333

d1px6b1 88335

d1md4a1 74632

d1md4b1 74634

d10gsa1 17556

d10gsb1 17557

d1kbna1 90947

d1kbnb1 90949

d4pgta1 17554

d4pgtb1 17555

d1eoga1 17560

d1eogb1 17561

d12gsa1 17558

d12gsb1 17559

d1aqxa1 17562

d1aqxb1 17563

d1aqxc1 17564

d1aqxd1 17565

d1aqwa1 17517

d1aqwb1 17518

d1aqwc1 17519

d1aqwd1 17520

d13gsa1 17530

d13gsb1 17531

d8gssa1 17521

d8gssb1 17522

d8gssc1 17523

d4gssa1 17570

d4gssb1 17571

d6gssa1 17534

d6gssb1 17535

d3gssa1 17538

d3gssb1 17539

d19gsa1 17546

d19gsb1 17547

d5gssa1 17552

d5gssb1 17553

d1eoha1 17574

d1eohb1 17575

d1eohc1 17576

d1eohd1 17577

d1eohe1 17578

d1eohf1 17579

d1eohg1 17580

d1eohh1 17581

d14gsa1 17572

d14gsb1 17573

d20gsa1 17582

d20gsb1 17583

d1gssa1 17584

d1gssb1 17585

d7gssa1 17566

d7gssb1 17567

d11gsa1 17568

d11gsb1 17569

d1glqa1 17588

d1glqb1 17589

d1glpa1 17590

d1glpb1 17591

d1baya1 17592

d1bayb1 17593

d2glra1 17594

d2glrb1 17595

d2oa7a1 138961

d2oa7b1 138963

d2oaca1 138965

d2oacb1 138967

d2oada1 138969

d2oadb1 138971

d1gtia1 17598

d1gtib1 17599

d1gtic1 17600

d1gtid1 17601

d1gtie1 17602

d1gtif1 17603

d1gsya1 17596

d1gsyb1 17597

d1tu7a1 112653

d1tu7b1 112655

d1tu8a1 112657

d1tu8b1 112659

d1tu8c1 112661

d1tu8d1 112663

d2gsra1 17586

d2gsrb1 17587

d1m0ua1 78359

d1m0ub1 78361

d1tw9a1 107381

d1tw9b1 107383

d1tw9c1 107385

d1tw9d1 107387

d1tw9e1 107389

d1tw9f1 107391

d1tw9g1 107393

d1tw9h1 107395

d2cvda1 130861

d2cvdb1 130863

d2cvdc1 130865

d2cvdd1 130867

d1iyha1 83790

d1iyhb1 83792

d1iyhc1 83794

d1iyhd1 83796

d1iyia1 83798

d1iyib1 83800

d1iyic1 83802

d1iyid1 83804

d1v40a1 113512

d1v40b1 113514

d1v40c1 113516

d1v40d1 113518

d2vcqa1 152922

d2vcqb1 152924

d2vcqc1 152926

d2vcqd1 152928

d2vcza1 152946

d2vczb1 152948

d2vczc1 152950

d2vczd1 152952

d2vcwa1 152930

d2vcwb1 152932

d2vcwc1 152934

d2vcwd1 152936

d2vcxa1 152938

d2vcxb1 152940

d2vcxc1 152942

d2vcxd1 152944

d2vd0a1 152954

d2vd0b1 152956

d2vd0c1 152958

d2vd0d1 152960

d2vd1a1 152962

d2vd1b1 152964

d2vd1c1 152966

d2vd1d1 152968

d3ee2a1 158117

d3ee2b1 158119

d1pd211 17713

d1pd221 17714

d2gsqa1 17715

d1gsqa1 17716

d1gwca1 70660

d1gwcb1 70662

d1gwcc1 70664

d1oyja1 87597

d1oyjb1 87599

d1oyjc1 87601

d1oyjd1 87603

d2ljra1 17709

d2ljrb1 17710

d3ljra1 17711

d3ljrb1 17712

d1ljra1 17707

d1ljrb1 17708

d1fw1a1 60051

d1e6ba1 59294

d1g7oa1 60332

d1nhya1 80520

d2fnoa1 133821

d2fnob1 133823

d1z9ha1 124758

d1z9hb1 124760

d1z9hc1 124762

d1z9hd1 124764

d2pbja1 149357

d2pbjb1 149359

d2pbjc1 149361

d2pbjd1 149363

d1okta1 93272

d1oktb1 93274

d1q4ja1 95793

d1q4jb1 95795

d2aawa1 126496

d2aawc1 126498

d1pa3a1 94405

d1pa3b1 94407

d1k0da1 67930

d1k0db1 67932

d1k0dc1 67934

d1k0dd1 67936

d1hqoa1 17749

d1hqob1 17750

d1g6wa1 17751

d1g6wb1 17752

d1g6wc1 17753

d1g6wd1 17754

d1g6ya1 17755

d1g6yb1 17756

d1k0ba1 67914

d1k0bb1 67916

d1k0bc1 67918

d1k0bd1 67920

d1k0ca1 67922

d1k0cb1 67924

d1k0cc1 67926

d1k0cd1 67928

d1k0aa1 67910

d1k0ab1 67912

d1jzra1 67872

d1jzrb1 67874

d1jzrc1 67876

d1jzrd1 67878

d2caqa1 130159

d2c8ua1 130124

d2c8ub1 130126

d2hqta1 147341

d2hqtb_ 147342

d2hqtc_ 147343

d2hqtd_ 147344

d2hqte_ 147345

d2hqtf_ 147346

d2hqtg_ 147347

d2hqth_ 147348

d2hqti_ 147349

d2hqtj_ 147350

d2hqtk_ 147351

d2hqtl_ 147352

d2hqtm_ 147353

d2hqtn_ 147354

d2hqto_ 147355

d2hqtp_ 147356

d2hqtq_ 147357

d2hqtr_ 147358

d2hqts_ 147359

d2hqtt_ 147360

d2hrkb_ 147377

d2hsnb_ 147394

d2hsmb1 147393

d3bula1 155620

d1bmta1 17757

d1bmtb1 17758

d1k7ya1 68272

d1k98a1 68338

d1khda1 77400

d1khdb1 77402

d1khdc1 77404

d1khdd1 77406

d1kgza1 77396

d1kgzb1 77398

d1o17a1 80765

d1o17b1 80767

d1o17c1 80769

d1o17d1 80771

d2gvqa1 135783

d2gvqb1 135785

d2gvqc1 135787

d2gvqd1 135789

d1zyka1 125830

d1zykb1 125832

d1zykc1 125834

d1zykd1 125836

d1zxya1 125803

d1zxyb1 125805

d1zxyc1 125807

d1zxyd1 125809

d1gxba1 83364

d1gxbb1 83366

d1gxbc1 83368

d1gxbd1 83370

d1v8ga1 100505

d1v8gb1 100507

d1brwa1 17764

d1brwb1 17765

d2tpta1 17759

d1otpa1 17760

d1azya1 17761

d1azyb1 17762

d1tpta1 17763

d1uoua1 99706

d2fzta1 134476

d2fztb_ 134477

d2g42a_ 134576

d2g42b_ 134577

d1uura1 100016

d1uusa1 100019

d1bf5a1 17766

d1bg1a1 17767

d1fioa_ 17773

d1s94a_ 98738

d1s94b_ 98739

d1ez3a_ 17768

d1ez3b_ 17769

d1ez3c_ 17770

d3c98b_ 173092

d1br0a_ 17772

d1lvfa_ 74280

d1lvfb_ 74281

d1hs7a_ 61236

d1vcsa1 119985

d3lg7a_ 180271

d3lg7b_ 180272

d3lg7c_ 180273

d1s2xa_ 105229

d3nyla_ 182631

d1tkna_ 107107

d3umha_ 186337

d3umia_ 186338

d3umka_ 186339

d3pmra_ 183850

d3pmrb_ 183851

d3q7la_ 184258

d3q7lb_ 184259

d3qmka_ 184496

d3qmkb_ 184497

d3q7ga_ 184242

d3q7gb_ 184243

d2fupa1 134184

d1t98a2 119197

d1t98b2 119199

d3buxb2 155648

d3buxd2 155651

d3buwb2 155642

d3buwd2 155645

d3bunb2 155627

d1yvha2 124097

d2cbla2 17774

d3bumb2 155624

d1b47a2 17775

d1b47b2 17776

d1b47c2 17777

d3buob2 155630

d3buod2 155633

d1fbva2 17778

d3bi1a1 155295

d3bhxa1 155289

d2or4a1 139258

d3bi0a1 155292

d2pvwa1 139755

d2oota1 139176

d2c6ca1 129989

d2pvva1 139752

d2c6ga1 129992

d2jbja1 138250

d2cija1 130493

d2c6pa1 130001

d2jbka1 138253

d1z8la1 124706

d1z8lb1 124709

d1z8lc1 124712

d1z8ld1 124715

d1de4c1 17779

d1de4f1 17780

d1de4i1 17781

d1cx8a1 17782

d1cx8b1 17783

d1cx8c1 17784

d1cx8d1 17785

d1cx8e1 17786

d1cx8f1 17787

d1cx8g1 17788

d1cx8h1 17789

d2nsua1 138546

d2nsub1 138549

d1wjta_ 114711

d1eo0a_ 17790

d2b4jc1 127835

d2b4jd_ 127836

d1z9ea1 124756

d3hphe_ 177754

d3hphf_ 177755

d3hphg_ 177756

d3hphh_ 177757

d2okua1 148809

d2okub_ 148810

d1f6va_ 17791

d3hqaa_ 177774

d3hqab_ 177775

d1cfaa_ 17793

d1kjsa_ 17792

d1c5aa_ 17794

d1v54h_ 100329

d1v54u_ 100343

d1v55h_ 100357

d1v55u_ 100371

d2occh_ 17796

d2occu_ 17797

d1ocrh_ 17798

d1ocru_ 17799

d1occh_ 17800

d1occu_ 17801

d1oczh_ 17802

d1oczu_ 17803

d1ocoh_ 17804

d1ocou_ 17805

d3ag3h_ 172098

d3ag3u_ 172110

d2dyrh_ 131907

d2dyru_ 131921

d3ag2h_ 172074

d3ag2u_ 172086

d3abmh_ 171976

d3abmu_ 171988

d2eijh_ 132147

d2eiju_ 132161

d2y69h_ 170639

d2y69u_ 170645

d3abkh_ 171928

d3abku_ 171940

d3ag4h_ 172122

d3ag4u_ 172134

d3ablh_ 171952

d3ablu_ 171964

d2eilh_ 132203

d2eilu_ 132217

d3ag1h_ 172050

d3ag1u_ 172062

d3asou_ 193677

d2eikh_ 132175

d2eiku_ 132189

d2dysh_ 131935

d2dysu_ 131949

d2zxwh_ 171578

d2zxwu_ 171590

d2eimh_ 132231

d2eimu_ 132245

d2einh_ 132259

d2einu_ 132273

d1n89a_ 85392

d1l6ha_ 77723

d1tuka_ 119342

d1mida_ 91280

d3gsha_ 176965

d3gshb_ 176966

d1jtba_ 17813

d1lipa_ 17811

d1be2a_ 17812

d1fk5a_ 59866

d1mzma_ 17814

d1fk1a_ 59862

d1fk3a_ 59864

d1fk4a_ 59865

d1fk2a_ 59863

d1fk0a_ 59861

d1mzla_ 17815

d1fk7a_ 59868

d1fk6a_ 59867

d1afha_ 17816

d1rzla_ 17817

d1uvca_ 113445

d1uvcb_ 113446

d1uvba_ 113444

d1uvaa_ 113443

d1bv2a_ 17818

d1bwoa_ 17807

d1bwob_ 17808

d1gh1a_ 17810

d1cz2a_ 17809

d1hypa_ 17806

d2alga_ 162826

d2algb_ 162827

d2b5sa_ 162993

d2b5sb_ 162994

d1hssa_ 17819

d1hssb_ 17820

d1hssc_ 17821

d1hssd_ 17822

d1beaa_ 17826

d1bfaa_ 17827

d1tmqb_ 17824

d1b1ua_ 17823

d1bipa_ 17825

d1psya_ 95081

d1s6da_ 105307

d1pnb.1 17828

d1aiea_ 17829

d1c26a_ 17830

d3saka_ 17871

d3sakb_ 17872

d3sakc_ 17873

d3sakd_ 17874

d1saea_ 17847

d1saeb_ 17848

d1saec_ 17849

d1saed_ 17850

d1saka_ 17831

d1sakb_ 17832

d1sakc_ 17833

d1sakd_ 17834

d1sala_ 17839

d1salb_ 17840

d1salc_ 17841

d1sald_ 17842

d1safa_ 17879

d1safb_ 17880

d1safc_ 17881

d1safd_ 17882

d1olha_ 17883

d1olhb_ 17884

d1olhc_ 17885

d1olhd_ 17886

d1olga_ 17835

d1olgb_ 17836

d1olgc_ 17837

d1olgd_ 17838

d1peta_ 17843

d1petb_ 17844

d1petc_ 17845

d1petd_ 17846

d1pesa_ 17859

d1pesb_ 17860

d1pesc_ 17861

d1pesd_ 17862

d2j0za1 147848

d2j0zb1 147849

d2j0zc1 147850

d2j0zd1 147851

d1hs5a_ 17869

d1hs5b_ 17870

d1a1ua_ 17863

d1a1uc_ 17864

d1adua1 17888

d1adub1 17889

d1anva1 17890

d1adva1 17891

d1advb1 17892

d2wb0x1 169164

d1p71a_ 87840

d1p71b_ 87841

d1p78a_ 87842

d1p78b_ 87843

d1p51a_ 87788

d1p51b_ 87789

d1p51c_ 87790

d1p51d_ 87791

d1huua_ 17897

d1huub_ 17898

d1huuc_ 17899

d1huea_ 17900

d1hueb_ 17901

d1mula_ 91465

d2o97a_ 138949

d2o97b1 145717

d1b8za_ 17902

d1b8zb_ 17903

d1riya_ 111820

d1owfa_ 87487

d1owga_ 87489

d1ihfa_ 17893

d1ouza_ 87449

d1owfb_ 87488

d2ht0b_ 136729

d1owgb_ 87490

d1ihfb_ 17894

d1ouzb_ 87450

d1exea_ 17906

d1exeb_ 17907

d1wtua_ 17904

d1wtub_ 17905

d3rhia_ 184964

d3rhib_ 184965

d3rhic_ 184966

d3rhid_ 184967

d2ht0a_ 136728

d1dp3a_ 59128

d3omya_ 183186

d3omyb_ 183187

d3c4ia_ 173040

d3c4ib_ 173041

d4dkya_ 193265

d1rm6c1 111876

d1rm6f1 111882

d1sb3c1 112056

d1sb3f1 112062

d1dgja1 17909

d1vlba1 108739

d3l4pa1 179942

d1sija1 105581

d1ffva1 17915

d1ffvd1 17916

d1ffua1 17917

d1ffud1 17918

d1n62a1 80090

d1n62d1 80096

d1n60a1 80066

d1n60d1 80072

d1n63a1 80102

d1n63d1 80108

d1n61a1 80078

d1n61d1 80084

d1n5wa1 80045

d1n5wd1 80051

d1t3qa1 106367

d1t3qd1 106373

d1jroa1 67137

d1jroc1 67143

d1jroe1 67149

d1jrog1 67155

d1jrpa1 67161

d1jrpc1 67167

d1jrpe1 67173

d1jrpg1 67179

d1v97a1 108436

d1v97b1 108442

d1vdva1 113614

d1vdvb1 113620

d1fo4a1 17910

d1fo4b1 17911

d3b9ja1 155000

d3b9ji1 155006

d1fiqa1 17912

d1n5xa1 85342

d1n5xb1 85348

d1dj8a_ 17919

d1dj8b_ 17920

d1dj8c_ 17921

d1dj8d_ 17922

d1dj8e_ 17923

d1dj8f_ 17924

d1bg8a_ 17925

d1bg8b_ 17926

d1bg8c_ 17927

d1af7a1 17928

d1bc5a1 17929

d1s5qb_ 105279

d1g1eb_ 17931

d1s5rb_ 105281

d2f05a1 132651

d1e91a_ 17930

d1pd7a_ 94514

d1sv0a_ 106035

d1sv0b_ 106036

d1sv4a_ 106041

d1sv4b_ 106042

d2jv3a_ 166258

d2qara_ 167496

d2qarb_ 167497

d2qard_ 167499

d2qare_ 167500

d2qb0a_ 150324

d2qb0c_ 150325

d2qb1a_ 167503

d2qb1b_ 167504

d1ji7a_ 71682

d1ji7b_ 71683

d1ji7c_ 71684

d1lkya_ 73985

d1lkyb_ 73986

d1lkyc_ 73987

d1lkyd_ 73988

d1lkye_ 73989

d1lkyf_ 73990

d1sxda_ 106079

d1sv0c_ 106037

d1sv0d_ 106038

d1sxea_ 106080

d1rg6a_ 111800

d1dxsa_ 17944

d1coka_ 17945

d1x40a1 121675

d1wwva1 121378

d1b0xa_ 17933

d1sgga_ 17943

d1b4fa_ 17934

d1b4fb_ 17935

d1b4fc_ 17936

d1b4fd_ 17937

d1b4fe_ 17938

d1b4ff_ 17939

d1b4fg_ 17940

d1b4fh_ 17941

d1f0ma_ 17942

d1ucva_ 99191

d1pk3a1 118715

d1pk1b1 118712

d1kw4a_ 73077

d1pk1a1 118711

d1pk1c_ 118713

d1oxja1 87517

d1v38a_ 100280

d1ow5a_ 93630

d1x9xa1 121829

d1x9xb1 121830

d2f3na1 132885

d2f3nb_ 132886

d2f3nc_ 132887

d2f44a_ 132911

d2f44b_ 132912

d2f44c_ 132913

d2d8ca1 131328

d1z1va1 124365

d1uqva_ 99798

d2y9ua_ 196257

d2qkqa_ 167699

d2qkqb_ 167700

d2gyta1 147195

d2dkya1 146539

d2h80a1 147238

d2jw2a1 148224

d1pk3b_ 118716

d1pk3c_ 118717

d1pk1d_ 118714

d3bs5b_ 172803

d3kkaa_ 196506

d3kkab_ 196505

d3kkac_ 179398

d3kkad_ 179399

d3kkae_ 179400

d3bq7a_ 172782

d3bq7b_ 172783

d3bq7c_ 172784

d3bq7d_ 172785

d3bq7e_ 172786

d3bq7f_ 172787

d1cuka2 17946

d1hjpa2 17947

d1d8la1 17948

d1d8lb1 17949

d1c7ya2 17950

d1bdxa2 17951

d1bdxb2 17952

d1bdxc2 17953

d1bdxd2 17954

d1bvsa2 17955

d1bvsb2 17956

d1bvsc2 17957

d1bvsd2 17958

d1bvse2 17959

d1bvsf2 17960

d1bvsg2 17961

d1bvsh2 17962

d1ixra1 76925

d1ixrb2 76928

d1dgsa1 17963

d1dgsb1 17964

d1v9pa1 100544

d1v9pb1 100547

d1kfta_ 77375

d2csba1 130751

d2csba2 130752

d2csba3 130753

d2csba4 130754

d1x2ia1 121641

d1x2ib_ 121642

d2a1jb1 126001

d1z00a1 124294

d2bgwa1 128500

d2bgwb1 128502

d2bhna1 128534

d2bhnb1 128536

d2bhnc1 128538

d2bhnd1 128540

d2a1ja1 126000

d2aq0a1 127136

d2aq0b1 127137

d1z00b1 124295

d3bzka1 155777

d3bzka2 155778

d3bzca1 155752

d3bzca2 155753

d2ocea1 148727

d2ocea2 148728

d2edua1 146805

d2duya1 146589

d1z3eb1 124401

d1lb2b_ 77871

d1lb2e_ 77872

d1cooa_ 17967

d1doqa_ 17968

d3ihqb_ 178327

d3gfkb_ 176587

d3k4ga_ 179067

d3k4gb_ 179068

d3k4gc_ 179069

d3k4gd_ 179070

d3k4ge_ 179071

d3k4gf_ 179072

d3k4gg_ 179073

d3k4gh_ 179074

d3n4mb_ 181899

d3n4mc_ 181900

d1szpa1 106176

d1szpb1 106178

d1szpc1 106180

d1szpd1 106182

d1szpe1 106184

d1szpf1 106186

d1b22a_ 17969

d2i1qa1 136984

d1t4ga1 106418

d2f1ja1 132770

d2b21a1 127687

d1xu4a1 116045

d2f1ia1 132768

d2f1ha1 132766

d1pzna1 95456

d1u9la_ 107751

d1u9lb_ 107752

d1wcla1 120893

d1y88a1 116560

d1ci4a_ 17970

d1ci4b_ 17971

d2bzfa_ 129558

d2ezza_ 17974

d2ezzb_ 17975

d2ezya_ 17972

d2ezyb_ 17973

d1qcka_ 17976

d1qckb_ 17977

d2ezxa_ 17978

d2ezxb_ 17979

d2odga1 139027

d2odgb1 139028

d2fmpa1 133786

d1tv9a1 112675

d2fmsa1 133792

d2isoa1 137606

d2pxia1 149911

d2fmqa1 133789

d2ispa1 137609

d1zjma1 125153

d3c2ma1 155882

d3c2ka1 155876

d1tvaa1 112678

d1bpya1 17980

d9icwa1 17982

d9icka1 17984

d3c2la1 155879

d1zjna1 125156

d9icxa1 17985

d8icoa1 17983

d7icia1 17986

d9icla1 17994

d7icea1 17987

d8icka1 17989

d1mq3a1 79397

d7icna1 17988

d7icka1 17993

d9icoa1 17995

d9icva1 17996

d1zqpa1 17991

d9icma1 18001

d1zqia1 17990

d7icha1 18000

d7icta1 18003

d7icqa1 18002

d8icia1 17998

d8icca1 17997

d8icna1 17999

d9icna1 18004

d7icma1 18009

d9icqa1 18010

d9icsa1 18012

d8icsa1 18008

d8icpa1 18006

d9icua1 18014

d7icpa1 18005

d9icta1 18013

d8icra1 18007

d9icra1 18011

d7icga1 18017

d8icqa1 18015

d7icra1 18016

d9icga1 18028

d8icaa1 18021

d8icxa1 18024

d9icja1 18030

d9icha1 18029

d8icma1 18023

d9icfa1 18027

d1zqka1 18019

d8icza1 18025

d8icba1 18022

d1zqaa1 17981

d9icaa1 18026

d1zqba1 18018

d7icla1 18020

d7icfa1 18031

d1zqma1 18033

d9icca1 18042

d7icsa1 18036

d1zqfa1 18035

d8icga1 18038

d1zqga1 18032

d9icba1 18041

d8icfa1 18037

d1zqna1 18034

d8icla1 18039

d8icua1 18040

d1zqoa1 18043

d9icya1 18050

d8icva1 18047

d8icya1 18048

d9icia1 18049

d8icta1 18046

d1mq2a1 79394

d8icja1 18045

d1bpxa1 18060

d7icoa1 18044

d1zqla1 18053

d1zqca1 18051

d9icpa1 18059

d7icua1 18056

d1zqsa1 18055

d8icea1 18057

d1bpza1 18058

d1zqqa1 18054

d1zqda1 18052

d8icwa1 18061

d8icha1 18062

d7icja1 18063

d1zqta1 18064

d7icva1 17992

d1zqea1 18065

d1zqra1 18066

d9icea1 18067

d1zqja1 18068

d1zqha1 18069

d1huza1 61281

d1huzb1 61283

d1huoa1 61273

d1huob1 61275

d2bpfa1 18070

d1bpda1 18073

d2bpga1 18074

d2bpgb1 18075

d1dk3a_ 18077

d1dk2a_ 18078

d1bnpa_ 18080

d1bnoa_ 18079

d1bpea1 18076

d2bcqa1 128304

d2bcra1 128307

d2pfna1 139672

d1xsna1 122282

d2bcva1 128316

d2pfoa1 139675

d2pfpa1 139678

d3c5ga1 155943

d3c5gb1 155946

d1rzta1 98224

d1rzte1 98227

d1rzti1 98230

d1rztm1 98233

d1xspa1 122285

d2bcsa1 128310

d2bcua1 128313

d1xsla1 122270

d1xsle1 122273

d1xsli1 122276

d1xslm1 122279

d3c5fa1 155937

d3c5fb1 155940

d2gwsa1 135813

d2gwse1 135816

d2gwsi1 135819

d2gwsm1 135822

d2pfqa1 139681

d1nzpa_ 92385

d1jmsa1 66889

d1kdha1 72349

d1keja1 72371

d1bgxt1 18083

d1taqa1 18082

d1taua1 18085

d1rxwa1 98059

d1rxva1 98055

d1rxvb1 98057

d1ul1x1 119690

d1ul1z1 119694

d1a77a1 18090

d1a76a1 18091

d1b43a1 18092

d1b43b1 18093

d1mc8a1 78945

d1mc8b1 78947

d1tfra1 18081

d2ihna1 147679

d1xo1a1 18086

d1xo1b1 18087

d1exna1 18088

d1exnb1 18089

d1ut8a1 99908

d1ut8b1 99910

d1ut5a1 99902

d1ut5b1 99904

d1d8ba_ 18094

d1wuda1 121283

d2e1ea1 131963

d2dgza1 131516

d1wudb_ 121284

d1wudd_ 121285

d2e1fa_ 131964

d1y14a_ 116322

d1y14c_ 116325

d2c35a1 129711

d1go3f_ 65407

d1go3n_ 65409

d1yt3a1 123992

d1yt3a2 123993

d2hbja1 136311

d2hbka1 136313

d2hbla1 136315

d2hbma1 136317

d2cpra1 130705

d1f44a1 64982

d1xo0a1 115674

d1xo0b1 115676

d4crxa1 18095

d4crxb1 18096

d1kbua1 72284

d1kbub1 72286

d1crxa1 18097

d1crxb1 18098

d2hofa1 147313

d2hofb1 147315

d2hoia1 147317

d2hoib1 147319

d2hoig1 147321

d2hoih1 147323

d2crxa1 18099

d2crxb1 18100

d1drga1 64792

d1pvpa1 95177

d1pvpb1 95179

d3crxa1 18101

d3crxb1 18102

d1ma7a1 84921

d1ma7b1 84923

d1pvra1 95185

d1pvrb1 95187

d1xnsa1 115647

d1xnsb1 115649

d1q3ua1 95752

d1q3ub1 95754

d1q3ue1 95756

d1q3uf1 95758

d5crxa1 18103

d5crxb1 18104

d1nzba1 92365

d1nzbb1 92367

d1nzbe1 92369

d1nzbf1 92371

d1pvqa1 95181

d1pvqb1 95183

d1q3va1 95760

d1q3vb1 95762

d1q3ve1 95764

d1q3vf1 95766

d1ouqa1 93557

d1ouqb1 93559

d1ouqe1 93561

d1ouqf1 93563

d1p4ea1 87764

d1p4eb1 87766

d1p4ec1 87768

d1p4ed1 87770

d1floa1 18106

d1flob1 18107

d1floc1 18108

d1flod1 18109

d1m6xa1 78708

d1m6xb1 78710

d1m6xc1 78712

d1m6xd1 78714

d1a0pa1 18105

d1zyma1 18110

d1zymb1 18111

d3ezba1 18120

d3ezaa1 18112

d1ezda1 18119

d1ezba1 18117

d1ezca1 18118

d3ezea1 18121

d1ezaa1 18113

d2ezca1 18115

d2ezba1 18114

d2ezaa1 18116

d1ryka_ 98105

d2fmpa2 133787

d1tv9a2 112676

d2fmsa2 133793

d2i9ga2 137127

d2isoa2 137607

d2pxia2 149912

d2fmqa2 133790

d2ispa2 137610

d1zjma2 125154

d3c2ma2 155883

d3c2ka2 155877

d1tvaa2 112679

d2p66a2 139507

d1bpya3 75819

d9icwa3 76129

d9icka3 76105

d3c2la2 155880

d1zjna2 125157

d9icxa3 76131

d8icoa3 76062

d7icia3 76008

d9icla3 76107

d7icea3 76000

d8icka3 76054

d1mq3a2 79398

d7icna3 76018

d7icka3 76012

d9icoa3 76113

d9icva3 76127

d1zqpa3 75965

d9icma3 76109

d1zqia3 75951

d7icha3 76006

d7icta3 76030

d7icqa3 76024

d8icia3 76050

d8icca3 76040

d8icna3 76060

d9icna3 76111

d7icma3 76016

d9icqa3 76117

d9icsa3 76121

d8icsa3 76070

d8icpa3 76064

d9icua3 76125

d7icpa3 76022

d9icta3 76123

d8icra3 76068

d9icra3 76119

d7icga3 76004

d8icqa3 76066

d7icra3 76026

d9icga3 76097

d8icaa3 76036

d8icxa3 76080

d9icja3 76103

d9icha3 76099

d8icma3 76058

d9icfa3 76095

d1zqka3 75955

d8icza3 76084

d8icba3 76038

d1zqaa3 75935

d9icaa3 76087

d1zqba3 75937

d7icla3 76014

d7icfa3 76002

d1zqma3 75959

d9icca3 76091

d7icsa3 76028

d1zqfa3 75945

d8icga3 76046

d1zqga3 75947

d9icba3 76089

d8icfa3 76044

d1zqna3 75961

d8icla3 76056

d8icua3 76074

d1zqoa3 75963

d9icya3 76133

d8icva3 76076

d8icya3 76082

d9icia3 76101

d8icta3 76072

d1mq2a2 79395

d8icja3 76052

d1bpxa3 75817

d7icoa3 76020

d1zqla3 75957

d1zqca3 75939

d9icpa3 76115

d7icua3 76032

d1zqsa3 75971

d8icea3 76042

d1bpza3 75821

d1zqqa3 75967

d1zqda3 75941

d8icwa3 76078

d8icha3 76048

d7icja3 76010

d1zqta3 75973

d7icva3 76034

d1zqea3 75943

d1zqra3 75969

d9icea3 76093

d1zqja3 75953

d1zqha3 75949

d2vana1 152826

d1zqwa1 75979

d1jn3a1 75864

d1rpla1 75933

d1zqya1 75983

d1bpba1 75811

d1zqua1 75975

d1huza3 75850

d1huzb3 75852

d1huoa3 75846

d1huob3 75848

d1zqxa1 75981

d2bpca1 75989

d1zqza1 75985

d1noma1 75929

d1zqva1 75977

d2bpfa3 75991

d1bpda2 75813

d2bpga3 75993

d2bpgb3 75995

d1bpea2 75815

d2bcqa2 128305

d2bcra2 128308

d2pfna2 139673

d1xsna2 122283

d2bcva2 128317

d2pfoa2 139676

d2pfpa2 139679

d3c5ga2 155944

d3c5gb2 155947

d1rzta2 98225

d1rzte2 98228

d1rzti2 98231

d1rztm2 98234

d1xspa2 122286

d2bcsa2 128311

d2bcua2 128314

d1xsla2 122271

d1xsle2 122274

d1xsli2 122277

d1xslm2 122280

d3c5fa2 155938

d3c5fb2 155941

d2gwsa2 135814

d2gwse2 135817

d2gwsi2 135820

d2gwsm2 135823

d2pfqa2 139682

d1jmsa3 75862

d1kdha3 75882

d1keja3 75884

d2axtu1 144940

d3arcu_ 172319

d4il6u_ 196656

d3bz2u_ 172962

d3bz1u_ 172951

d1m6ya1 78716

d1m6yb1 78718

d1n2xa1 79860

d1n2xb1 79862

d1wg8a1 114605

d1wg8b1 114607

d1q46a1 111594

d1kl9a1 111574

d1q8ka3 111596

d2ahob1 126770

d2fj6a1 133547

d2o6ka1 148632

d2o6kb_ 148633

d3ci0k1 156659

d3ci0k2 156660

d2gola_ 135438

d1hiwa_ 18122

d1hiwb_ 18123

d1hiwc_ 18124

d1hiwq_ 18125

d1hiwr_ 18126

d1hiws_ 18127

d1upha_ 99756

d2jmga1 138357

d2h3qa1 136052

d2h3va1 136057

d2h3ia1 136045

d2h3fa1 136044

d2h3za1 136062

d1l6na1 73624

d1tama_ 18128

d2hmxa_ 18129

d1ecwa_ 18131

d1ed1a_ 18130

d1jvra_ 18132

d2f76x1 133085

d1baxa_ 18133

d1a6sa_ 18134

d1heka_ 65818

d1hekb_ 65819

d1mn8a_ 79318

d1mn8b_ 79319

d1mn8c_ 79320

d1mn8d_ 79321

d1uhua_ 107854

d1qgta_ 18135

d1qgtb_ 18136

d1qgtc_ 18137

d1qgtd_ 18138

d3kxsa_ 179796

d3kxsb_ 179797

d3kxsc_ 179798

d3kxsd_ 179799

d3kxse_ 179800

d3kxsf_ 179801

d1aepa_ 18139

d1ls4a_ 84689

d1eq1a_ 64910

d1l9la_ 77827

d1nkla_ 18140

d3bqqa_ 172790

d3bqqb_ 172791

d3bqqc_ 172792

d3bqqd_ 172793

d4ddja_ 196036

d2doba_ 163647

d2rb3a_ 168037

d2rb3b_ 168038

d2rb3c_ 168039

d2rb3d_ 168040

d2gtga_ 135649

d2r0ra_ 167909

d2r0rb_ 167910

d2qypa_ 151477

d2qypb_ 151478

d2z9aa_ 154235

d2z9ab_ 154236

d2r1qa_ 167924

d1m12a_ 84745

d1sn6a1 118972

d1qdma1 18141

d1qdmb1 18142

d1qdmc1 18143

d1n69a_ 80118

d1n69b_ 80119

d1n69c_ 80120

d1of9a_ 92821

d3bqpa_ 172788

d3bqpb_ 172789

d1o82a_ 92630

d1o82b_ 92631

d1o82c_ 92632

d1o82d_ 92633

d1o83a_ 92634

d1o83b_ 92635

d1o83c_ 92636

d1o83d_ 92637

d1o84a_ 92638

d1o84b_ 92639

d1e68a_ 18144

d1n00a_ 85241

d1aina_ 18145

d1bo9a_ 18146

d1hm6a_ 18147

d1hm6b_ 18148

d1mcxa_ 84933

d1w7ba_ 114317

d1xjla_ 115390

d1xjlb_ 115391

d1axna_ 18149

d1aiia_ 18150

d1i4aa_ 61681

d1anna_ 18151

d1aowa_ 18152

d1yiia_ 162209

d1alaa_ 18153

d1yj0a_ 162212

d1hvda_ 18154

d1anxa_ 18161

d1anxb_ 18162

d1anxc_ 18163

d1hvfa_ 18155

d1hvea_ 18156

d1avra_ 18157

d1avha_ 18159

d1avhb_ 18160

d1sava_ 18158

d1anwa_ 18164

d1anwb_ 18165

d1hvga_ 18166

d1haka_ 18167

d1hakb_ 18168

d2ie7a_ 137293

d2ie6a_ 137292

d1g5na_ 60287

d1a8aa_ 18169

d1n41a_ 79978

d2rana_ 18170

d1n42a_ 79979

d1a8ba_ 18171

d1bcya_ 18172

d1bc1a_ 18173

d2h0ma_ 164889

d1bc3a_ 18175

d1bc0a_ 18174

d1bcwa_ 18176

d2h0la_ 164888

d1bcza_ 18177

d2h0ka_ 164886

d2h0kb_ 164887

d1n44a_ 79980

d1avca1 18178

d1avca2 18179

d1m9ia1 74589

d1m9ia2 74590

d1aeia_ 18180

d1aeib_ 18181

d1aeic_ 18182

d1aeid_ 18183

d1aeie_ 18184

d1aeif_ 18185

d1dm5a_ 18186

d1dm5b_ 18187

d1dm5c_ 18188

d1dm5d_ 18189

d1dm5e_ 18190

d1dm5f_ 18191

d1dk5a_ 18192

d1dk5b_ 18193

d2q4ca_ 139843

d2q4cb_ 139844

d1ycna_ 116612

d1ycnb_ 116613

d3brxa_ 155521

d2zoca_ 171382

d2zocb_ 171383

d2xo2a_ 196259

d2zhja_ 171223

d2zhia_ 171222

d1w3wa_ 161888

d1w45a_ 161889

d1w45b_ 161890

d1tada1 18194

d1tadb1 18195

d1tadc1 18196

d1taga1 18197

d1tnda1 18198

d1tndb1 18199

d1tndc1 18200

d1azta1 18201

d1aztb1 18202

d1azsc1 18203

d1culc1 18204

d1cs4c1 18205

d1cjtc1 18206

d1cjuc1 18207

d1cjkc1 18209

d1cjvc1 18208

d2gvdc1 135773

d1tl7c1 112503

d1u0hc1 112918

d2gvzc1 135799

d3umra1 192178

d3umsa1 192180

d2om2a1 148861

d2om2c1 148863

d2gtpa1 135678

d2gtpb1 135680

d1y3aa1 122582

d1y3ab1 122584

d1y3ac1 122586

d1y3ad1 122588

d2ik8a1 137484

d2ik8c1 137486

d2g83a1 134762

d2g83b1 134764

d1zcba1 124901

d2bcjq1 128291

d1zcaa1 124897

d1zcab1 124899

d2rgna1 152012

d2rgnd1 152015

d1cipa1 18210

d1giaa1 18211

d1as0a1 18212

d1bh2a1 18214

d1bofa1 18217

d1gfia1 18219

d1gdda1 18218

d1gila1 18223

d1gita1 18222

d1as3a1 18224

d1gg2a1 18226

d1gp2a1 18225

d1as2a1 18227

d1agra1 18228

d1agrd1 18229

d1kjya1 72624

d1kjyc1 72626

d1shza1 118964

d1shzd1 118966

d1gota1 18213

d1fqja1 18215

d1fqjd1 18216

d1fqka1 18220

d1fqkc1 18221

d1ej5a_ 18268

d1t84a_ 106649

d1skyb1 18313

d2jdia1 145683

d2jdib1 145686

d2jdic1 145689

d2ck3a1 145033

d2ck3b1 145036

d2ck3c1 145039

d1w0ja1 108989

d1w0jb1 108992

d1w0jc1 108995

d1e79a1 18269

d1e79b1 18270

d1e79c1 18271

d1w0ka1 109008

d1w0kb1 109011

d1w0kc1 109014

d1h8ea1 60738

d1h8eb1 60741

d1h8ec1 60744

d1e1ra1 18275

d1e1rb1 18276

d1e1rc1 18277

d1bmfa1 18281

d1bmfb1 18282

d1bmfc1 18283

d1nbma1 18287

d1nbmb1 18288

d1nbmc1 18289

d1e1qa1 18293

d1e1qb1 18294

d1e1qc1 18295

d1efra1 18299

d1efrb1 18300

d1efrc1 18301

d1h8ha1 60759

d1h8hb1 60762

d1h8hc1 60765

d1ohha1 87015

d1ohhb1 87018

d1ohhc1 87021

d1cowa1 18305

d1cowb1 18306

d1cowc1 18307

d1maba1 18311

d2f43a1 145131

d1fx0a1 60080

d1kmha1 72745

d1skye1 18314

d2jdid1 138263

d2jdie1 138266

d2jdif1 138269

d2ck3d1 130553

d2ck3e1 130556

d2ck3f1 130559

d1w0jd1 108998

d1w0je1 109001

d1w0jf1 109004

d1e79d1 18272

d1e79e1 18273

d1e79f1 18274

d1w0kd1 109017

d1w0ke1 109020

d1w0kf1 109023

d1h8ed1 60747

d1h8ee1 60750

d1h8ef1 60753

d1e1rd1 18278

d1e1re1 18279

d1e1rf1 18280

d1bmfd1 18284

d1bmfe1 18285

d1bmff1 18286

d1nbmd1 18290

d1nbme1 18291

d1nbmf1 18292

d1e1qd1 18296

d1e1qe1 18297

d1e1qf1 18298

d1efrd1 18302

d1efre1 18303

d1efrf1 18304

d1h8hd1 60768

d1h8he1 60771

d1h8hf1 60774

d1ohhd1 87024

d1ohhe1 87027

d1ohhf1 87030

d1cowd1 18308

d1cowe1 18309

d1cowf1 18310

d1mabb1 18312

d2f43b1 132908

d1fx0b1 60083

d1kmhb1 72748

d1fkma1 18315

d1fkma2 18316

d2g77a1 134730

d2g77a2 134731

d1q0qa1 104464

d1q0qb1 104467

d1q0ha1 104440

d1jvsa1 77187

d1jvsb1 77190

d2egha1 132121

d2eghb1 132124

d1t1ra1 106262

d1t1rb1 106266

d1t1sa1 106270

d1t1sb1 106274

d1onoa1 87159

d1onob1 87162

d1k5ha1 68192

d1k5hb1 68195

d1k5hc1 68198

d1q0la1 104455

d1onna1 87153

d1onnb1 87156

d1onpa1 87165

d1onpb1 87168

d1r0ka1 104723

d1r0kb1 104726

d1r0kc1 104729

d1r0kd1 104732

d1r0la1 104735

d1r0lb1 104738

d1r0lc1 104741

d1r0ld1 104744

d2o3la1 148579

d2o3lb_ 148580

d2o4ta1 148590

d2hh6a1 147285

d1abva_ 18317

d2a7ub1 126367

d2oeba1 148749

d4gkfa_ 194109

d4gkfb_ 194108

d1g7da_ 18318

d2c0ga1 129590

d2c0gb1 129592

d1ovna1 93602

d1ovnb1 93604

d2c0fa1 129586

d2c0fb1 129588

d2c1ya1 129647

d2c0ea1 129582

d2c0eb1 129584

d2qtva1 151357

d1m2oa1 78480

d1m2oc1 78486

d1m2va1 78492

d1pd1a1 94507

d1pd0a1 94502

d1pcxa1 94497

d1m2vb1 78497

d1dvka_ 18319

d1dvkb_ 18320

d1u7ka_ 113087

d1u7kb_ 113088

d1u7kc_ 113089

d1u7kd_ 113090

d1u7ke_ 113091

d1u7kf_ 113092

d3bp9a_ 172741

d3bp9b_ 172742

d3bp9c_ 172743

d3bp9d_ 172744

d3bp9e_ 172745

d3bp9f_ 172746

d3bp9g_ 172747

d3bp9h_ 172748

d3bp9i_ 172749

d3bp9j_ 172750

d3bp9k_ 172751

d3bp9l_ 172752

d3bp9m_ 172753

d3bp9n_ 172754

d3bp9o_ 172755

d3bp9p_ 172756

d3bp9q_ 172757

d3bp9r_ 172758

d3bp9s_ 172759

d3bp9t_ 172760

d3bp9u_ 172761

d3bp9v_ 172762

d3bp9x_ 172763

d3bp9y_ 172764

d2eiaa2 18329

d2eiab2 18330

d1eiaa2 18331

d2pwoa_ 167310

d2pwob_ 167311

d2pwoc_ 167312

d2pwod_ 167313

d1m9fc_ 84900

d1m9fd_ 84901

d1m9xc_ 84907

d1m9xd_ 84908

d1m9xg_ 84911

d1m9xh_ 84912

d4e91a_ 197294

d4e91b_ 197293

d1m9ec_ 84896

d1m9ed_ 84897

d1m9yc_ 84915

d1m9yd_ 84916

d1m9yg_ 84919

d1m9yh_ 84920

d1m9dc_ 84892

d1m9dd_ 84893

d4dgac_ 196071

d4dgad_ 196072

d2pwma_ 167302

d2pwmb_ 167303

d2pwmc_ 167304

d2pwmd_ 167305

d2pwme_ 167306

d2pwmf_ 167307

d2pwmg_ 167308

d2pwmh_ 167309

d2gona_ 164784

d2gonb_ 164785

d2gonc_ 164786

d2gond_ 164787

d1m9cc_ 84888

d1m9cd_ 84889

d2golb_ 135439

d2gold_ 135440

d1ak4c_ 18321

d1ak4d_ 18322

d1e6jp2 18323

d1afva_ 18324

d1afvb_ 18325

d2jpra1 148169

d1l6na2 73625

d1gwpa_ 70674

d3dika2 157752

d1qrj.1 18332

d1g03a_ 60162

d1em9a_ 18333

d1em9b_ 18334

d1p7na_ 94308

d1d1da2 18335

d2pxrc_ 149918

d2xdea_ 170044

d2xdeb_ 170045

d4b4na_ 194691

d2x2dd_ 169823

d2x2de_ 169824

d2x82a_ 169932

d2x82b_ 169933

d2x82c_ 169934

d2x82d_ 169935

d2y4za_ 170604

d4htwa_ 193088

d3o0gd_ 182722

d3o0ge_ 182723

d1unld_ 113326

d1unle_ 113327

d1ungd_ 113318

d1unge_ 113319

d1unhd_ 113322

d1unhe_ 113323

d1h4ld_ 70873

d1h4le_ 70874

d3ddqb1 157559

d3ddqb2 157560

d3ddqd1 157561

d3ddqd2 157562

d3bhtb2 155278

d3bhtd2 155280

d3bhvb2 155286

d3bhvd2 155288

d1vina1 18354

d1vina2 18355

d3bhub2 155282

d3bhud2 155284

d2g9xb1 134845

d2g9xb2 134846

d2g9xd1 134848

d2g9xd2 134849

d3dogb1 157815

d3dogb2 157816

d3dogd1 157817

d3dogd2 157818

d3ddpb1 157555

d3ddpb2 157556

d3ddpd1 157557

d3ddpd2 157558

d2cchb1 130237

d2cchb2 130238

d2cchd1 130240

d2cchd2 130241

d1oiub1 103959

d1oiub2 103960

d1oiud1 103962

d1oiud2 103963

d1h1sb1 76496

d1h1sb2 76497

d1h1sd1 76499

d1h1sd2 76500

d1h1rb1 76490

d1h1rb2 76491

d1h1rd1 76493

d1h1rd2 76494

d1h1pb1 76478

d1h1pb2 76479

d1h1pd1 76481

d1h1pd2 76482

d1jsub1 18336

d1jsub2 18337

d1h27b1 76535

d1h27b2 76536

d1h27d1 76538

d1h27d2 76539

d1oi9b1 103947

d1oi9b2 103948

d1oi9d1 103950

d1oi9d2 103951

d1qmzb1 18338

d1qmzb2 18339

d1qmzd1 18340

d1qmzd2 18341

d1p5eb1 87797

d1p5eb2 87798

d1p5ed1 87800

d1p5ed2 87801

d1vywb1 108923

d1vywb2 108924

d1vywd1 108926

d1vywd2 108927

d1h26b1 76529

d1h26b2 76530

d1h26d1 76532

d1h26d2 76533

d1okwb1 93289

d1okwb2 93290

d1okwd1 93292

d1okwd2 93293

d1finb1 18342

d1finb2 18343

d1find1 18344

d1find2 18345

d1okvb1 93283

d1okvb2 93284

d1okvd1 93286

d1okvd2 93287

d1pkdb1 88142

d1pkdb2 88143

d1pkdd1 88145

d1pkdd2 88146

d1ol2b1 93305

d1ol2b2 93306

d1ol2d1 93308

d1ol2d2 93309

d1urcb1 113405

d1urcb2 113406

d1urcd1 113408

d1urcd2 113409

d1ogub1 92943

d1ogub2 92944

d1ogud1 92946

d1ogud2 92947

d1h24b1 76517

d1h24b2 76518

d1h24d1 76520

d1h24d2 76521

d1jstb1 18346

d1jstb2 18347

d1jstd1 18348

d1jstd2 18349

d1e9hb1 64813

d1e9hb2 64814

d1e9hd1 64816

d1e9hd2 64817

d1oiyb1 103965

d1oiyb2 103966

d1oiyd1 103968

d1oiyd2 103969

d1h25b1 76523

d1h25b2 76524

d1h25d1 76526

d1h25d2 76527

d1gy3b1 70729

d1gy3b2 70730

d1gy3d1 70732

d1gy3d2 70733

d1h1qb1 76484

d1h1qb2 76485

d1h1qd1 76487

d1h1qd2 76488

d3eidb1 158160

d3eidb2 158161

d3eidd1 158162

d3eidd2 158163

d2ccib1 130243

d2ccib2 130244

d2ccid1 130246

d2ccid2 130247

d1fvvb1 18350

d1fvvb2 18351

d1fvvd1 18352

d1fvvd2 18353

d3ej1b1 158164

d3ej1b2 158165

d3ej1d1 158166

d3ej1d2 158167

d1ol1b1 93299

d1ol1b2 93300

d1ol1d1 93302

d1ol1d2 93303

d1h28b1 76541

d1h28b2 76542

d1h28d1 76544

d1h28d2 76545

d1kxua1 18358

d1kxua2 18359

d1jkwa1 18356

d1jkwa2 18357

d2i53a1 147505

d2i53a2 147506

d3blhb1 155394

d3blhb2 155395

d3blrb1 155400

d3blrb2 155401

d3blqb1 155397

d3blqb2 155398

d2pk2a1 149586

d2pk2a2 149587

d2pk2b1 149588

d2pk2b2 149589

d2pk2c1 149590

d2pk2c2 149591

d2pk2d1 149592

d2pk2d2 149593

d2ivxa1 147813

d2ivxa2 147814

d2ivxb1 147815

d1w98b1 144548

d1w98b2 144549

d2f2ca2 132806

d2eufa1 132384

d2eufa2 132385

d1bu2a1 18360

d1bu2a2 18361

d1xo2a1 122198

d1xo2a2 122199

d1jowa1 66998

d1jowa2 66999

d1g3nc1 18366

d1g3nc2 18367

d1g3ng1 18368

d1g3ng2 18369

d1f5qb1 18362

d1f5qb2 18363

d1f5qd1 18364

d1f5qd2 18365

d1vola1 18370

d1vola2 18371

d1c9ba1 18372

d1c9ba2 18373

d1c9be1 18374

d1c9be2 18375

d1c9bi1 18376

d1c9bi2 18377

d1c9bm1 18378

d1c9bm2 18379

d1c9bq1 18380

d1c9bq2 18381

d2phga1 139694

d2phga2 139695

d1tfba1 18382

d1tfba2 18383

d1aisb1 18384

d1aisb2 18385

d1d3ub1 18386

d1d3ub2 18387

d2r7ga1 151637

d2r7ga2 151638

d2r7gc1 151639

d2r7gc2 151640

d1guxa_ 18388

d1guxb_ 18389

d1n4ma1 79994

d1n4ma2 79995

d1n4mb1 79996

d1n4mb2 79997

d1ad6a_ 18390

d1o9ka_ 86698

d1o9kb_ 86699

d1o9kc_ 86700

d1o9kd_ 86701

d1o9ke_ 86702

d1o9kf_ 86703

d1o9kg_ 86704

d1o9kh_ 86705

d1gh6b1 65192

d1gh6b2 65193

d1husa_ 18391

d2qang1 150274

d2qalg1 150220

d2qbfg1 150494

d2qbdg1 150440

d2qoyg1 151097

d2qp0g1 151150

d3df1g1 157609

d3df3g1 157663

d2avyg1 144849

d2aw7g1 144892

d1vs5g1 144442

d1vs7g1 144483

d2qb9g1 150334

d2qbbg1 150387

d2i2pg1 145427

d2i2ug1 145469

d2qoug1 150991

d2qowg1 151044

d2qbhg1 150548

d2qbjg1 150602

d2vhog1 153129

d2z4kg1 154058

d2z4mg1 154112

d2vhpg1 153151

d1iqva_ 62661

d1rssa_ 18392

d2uubg1 139939

d2vqeg1 153431

d2vqfg1 153450

d2uuag1 139919

d1j5eg_ 71550

d1fjgg_ 18394

d2uucg1 139959

d2uxcg1 140010

d1xmqg_ 115536

d2uu9g1 139899

d1n32g_ 79876

d1xnqg_ 115610

d1xnrg_ 115632

d1hr0g_ 18395

d1hnzg_ 18396

d2j02g1 137872

d2j00g1 137845

d1xmog_ 115506

d1i94g_ 61998

d2uxdg1 152287

d1hnwg_ 18397

d1hnxg_ 18398

d3d5cg1 157371

d3d5ag1 157341

d2e5lg1 132031

d1n33g_ 79898

d2hhhg1 136483

d2uxbg1 152268

d1n34g_ 79920

d2v48g1 152525

d1i96g_ 62042

d2v46g1 152489

d2f4vg1 132944

d1n36g_ 79943

d1i97g_ 62065

d1i95g_ 62020

d2hgpj1 136427

d2hgij1 136406

d2hgrj1 136448

d2qnhh1 150931

d2ow8h1 139405

d1yl4j1 123603

d1x18f1 121574

d2b9og1 128169

d2b64g1 127939

d2b9mg1 128132

d2dtra2 18399

d1g3wa2 65134

d1g3sa2 65125

d1bi1a2 18402

d1bi0a2 18408

d1fwza2 60078

d1p92a2 104086

d1xcva2 121863

d1g3ta2 65128

d1g3tb2 65131

d1bi3a2 18405

d1bi3b2 18406

d1bi2a2 18403

d1bi2b2 18404

d2tdxa2 18407

d1g3ya2 65137

d1f5ta2 18409

d1f5tb2 18410

d1f5tc2 18411

d1f5td2 18412

d1ddna2 18413

d1ddnb2 18414

d1ddnc2 18415

d1ddnd2 18416

d1dpra2 18400

d1dprb2 18401

d1c0wa2 18417

d1c0wb2 18418

d1c0wc2 18419

d1c0wd2 18420

d2isya2 137613

d2isyb2 137615

d1fx7a2 60089

d1fx7b2 60092

d1fx7c2 60095

d1fx7d2 60098

d2isza2 137617

d2iszb2 137619

d2iszc2 137621

d2iszd2 137623

d2it0a2 137625

d2it0b2 137627

d2it0c2 137629

d2it0d2 137631

d1u8ra2 113169

d1u8rb2 113172

d1u8rc2 113175

d1u8rd2 113178

d1u8rg2 113181

d1u8rh2 113184

d1u8ri2 113187

d1u8rj2 113190

d1b1ba2 18421

d2ev0a2 132414

d2ev0b2 132416

d1on2a2 87104

d1on2b2 87106

d1on1a2 87100

d1on1b2 87102

d2f5da2 132980

d2f5db2 132982

d2f5ea2 132984

d2f5eb2 132986

d2f5fa2 132988

d2hyfa2 136881

d2hyfb2 136883

d2hygd2 136889

d2gf5a1 135075

d1e3ya_ 18426

d1e41a_ 18424

d1fada_ 18428

d1ddfa_ 18423

d1wh4a_ 114632

d1wmga_ 114738

d1wmgb_ 114739

d1wmgc_ 114740

d1wmgd_ 114741

d1wmge_ 114742

d1wmgf_ 114743

d1ngra_ 18422

d1d2za_ 18437

d1d2zc_ 18438

d1ik7a_ 71241

d1ik7b_ 71242

d1ygoa1 123146

d1d2zb_ 18439

d1d2zd_ 18440

d1icha_ 71182

d3ezqa_ 175332

d3ezqc_ 175333

d3ezqe_ 175334

d3ezqg_ 175335

d3ezqi_ 175336

d3ezqk_ 175337

d3ezqm_ 175338

d3ezqo_ 175339

d2a9ia_ 162730

d4f44b_ 193053

d1cy5a_ 18430

d2ygsa_ 18431

d3ygsc_ 18432

d1cwwa_ 18434

d1c15a_ 18433

d2a5yb2 144787

d1dgna_ 18436

d3ygsp_ 18435

d3crda_ 18429

d2p1ha_ 139457

d2gf5a2 135076

d1a1wa_ 18425

d1a1za_ 18427

d1n3ka_ 79965

d1ucpa_ 99186

d1pn5a1 94948

d1hw1a2 18441

d1hw1b2 18442

d1e2xa2 18443

d1h9ga2 60828

d1hw2a2 18444

d1hw2b2 18445

d1h9ta2 60848

d1h9tb2 60850

d2hs5a2 147380

d1ey1a_ 18449

d1eyva_ 18446

d1eyvb_ 18447

d1tzva_ 107526

d1tzwa_ 107527

d1tzta_ 107523

d1tztb_ 107524

d1tzxa_ 107528

d1tzxb_ 107529

d1tzua_ 107525

d3d3ba_ 173658

d3imqa_ 178414

d3imqb_ 178415

d3imqc_ 178416

d3d3ca_ 173659

d3d3cb_ 173660

d3d3cc_ 173661

d1q8ca_ 96202

d1sqga1 105912

d1sqfa1 105910

d1a5ta1 18450

d1jr3e1 63253

d1xxhe1 116171

d1xxhj1 116181

d1xxie1 116191

d1xxij1 116201

d1jr3d1 63251

d1jqjc1 67097

d1jqjd1 67099

d1xxha1 116163

d1xxhf1 116173

d1xxia1 116183

d1xxif1 116193

d1jr3a1 63245

d1jr3b1 63247

d1jr3c1 63249

d1xxhb1 116165

d1xxhc1 116167

d1xxhd1 116169

d1xxhg1 116175

d1xxhh1 116177

d1xxhi1 116179

d1xxib1 116185

d1xxic1 116187

d1xxid1 116189

d1xxig1 116195

d1xxih1 116197

d1xxii1 116199

d2gnoa1 135414

d1iqpa1 62649

d1iqpb1 62651

d1iqpc1 62653

d1iqpd1 62655

d1iqpe1 62657

d1iqpf1 62659

d1sxja1 106081

d1sxjd1 106087

d1sxjc1 106085

d1sxjb1 106083

d1sxje1 106089

d2r9ga1 151768

d2r9gb_ 151769

d2r9gc1 151770

d2r9gd_ 151771

d2r9ge_ 151772

d2r9gf_ 151773

d2r9gg_ 151774

d2r9gh_ 151775

d2r9gi_ 151776

d2r9gj_ 151777

d2r9gk_ 151778

d2r9gl_ 151779

d2r9gm_ 151780

d2r9gn_ 151781

d2r9go_ 151782

d2r9gp_ 151783

d2qw6a1 151401

d3bgea1 155245

d3bgeb_ 155246

d3ctda1 156981

d1b79a_ 18451

d1b79b_ 18452

d1b79c_ 18453

d1b79d_ 18454

d1jwea_ 18455

d1m15a1 78368

d1rl9a1 104979

d3m10a1 180700

d3m10b1 180702

d1p52a1 87792

d1bg0a1 18476

d1sd0a1 105424

d1p50a1 87786

d1qh4a1 18462

d1qh4b1 18463

d1qh4c1 18464

d1qh4d1 18465

d1crka1 18458

d1crkb1 18459

d1crkc1 18460

d1crkd1 18461

d1g0wa1 18475

d1qk1a1 18466

d1qk1b1 18467

d1qk1c1 18468

d1qk1d1 18469

d1qk1e1 18470

d1qk1f1 18471

d1qk1g1 18472

d1qk1h1 18473

d1i0ea1 83655

d1i0eb1 83657

d1i0ec1 83659

d1i0ed1 83661

d1vrpa1 120473

d1u6ra1 119600

d1u6rb1 119602

d2crka1 18474

d1tx9a1 119383

d1al01_ 18477

d1al02_ 18478

d1al03_ 18479

d1al04_ 18480

d1cd31_ 18481

d1cd32_ 18482

d1cd33_ 18483

d1cd34_ 18484

d1llaa1 18485

d1oxya1 18486

d1nola1 18487

d1ll1a1 18488

d1hc1a1 18494

d1hcya1 18495

d1hcyb1 118487

d1hcyc1 118490

d1hcyd1 118493

d1hcye1 118496

d1hcyf1 118499

d1llaa2 18496

d1oxya2 18497

d1nola2 18498

d1ll1a2 18499

d1hc1a2 18505

d1hcya2 18506

d1hcyb2 118488

d1hcyc2 118491

d1hcyd2 118494

d1hcye2 118497

d1hcyf2 118500

d1js8a1 67219

d1js8b1 67221

d1lnla1 84635

d1lnlb1 84637

d1lnlc1 84639

d1bt3a_ 18507

d1bt1a_ 18508

d1bt1b_ 18509

d1buga_ 18510

d1bugb_ 18511

d1bt2a_ 18512

d1bt2b_ 18513

d2p3xa_ 167000

d1by1a_ 18515

d1kz7a1 73333

d1kz7c1 73336

d1kzga1 73355

d1kzgc1 73358

d1lb1a1 73784

d1lb1c1 73787

d1lb1e1 73790

d1lb1g1 73793

d1rj2a1 104954

d1rj2d1 104956

d1rj2g1 104958

d1rj2j1 104960

d1ki1b1 72497

d1ki1d1 72500

d1foea1 18517

d1foec1 18518

d1foee1 18519

d1foeg1 18520

d1xcga1 115120

d1xcge1 115123

d1txda1 107422

d1x86a1 109504

d1x86c1 109507

d1x86e1 109510

d1x86g1 109513

d2dfka1 145077

d1f5xa_ 18516

d1dbha1 18514

d1xdva1 115180

d1xdvb1 115183

d1xd4a1 115149

d1xd4b1 115152

d1ntya1 103873

d2nz8b1 138838

d1boua_ 18521

d1bouc_ 18522

d1b4ua_ 18523

d1b4uc_ 18524

d1hbna1 60898

d1hbnd1 60903

d1mroa1 18525

d1mrod1 18526

d1hbma1 60888

d1hbmd1 60893

d1hboa1 60908

d1hbod1 60913

d1hbua1 60918

d1hbud1 60923

d1e6va1 18527

d1e6vd1 18528

d1e6ya1 18529

d1e6yd1 18530

d1hbnb1 60900

d1hbne1 60905

d1mrob1 18531

d1mroe1 18532

d1hbmb1 60890

d1hbme1 60895

d1hbob1 60910

d1hboe1 60915

d1hbub1 60920

d1hbue1 60925

d1e6vb1 18533

d1e6ve1 18534

d1e6yb1 18535

d1e6ye1 18536

d1bgfa_ 18537

d1dk8a_ 18548

d1emua_ 18549

d1omwa1 87086

d2bcja1 128286

d1ym7a1 123685

d1ym7b1 123688

d1ym7c1 123691

d1ym7d1 123694

d1cmza_ 18547

d1iapa_ 62119

d1htjf_ 61254

d2ik8b1 145528

d2ik8d_ 145529

d3c7lb_ 195303

d3c7kb_ 173069

d3c7kd_ 173070

d2jm5a1 138355

d1agre_ 18538

d1agrh_ 18539

d1ezta_ 18541

d1ezya_ 18540

d1fqia_ 18542

d1fqjb_ 18543

d1fqje_ 18544

d1fqkb_ 18545

d1fqkd_ 18546

d2odeb_ 166648

d2oded_ 166649

d2v4zb_ 168341

d3cx8b_ 173526

d3cx7b_ 173525

d3cx6b_ 173524

d2a72a_ 162700

d2a72b_ 162701

d2bv1a_ 163167

d2bv1b_ 163168

d2oj4a_ 166715

d2af0a_ 162776

d2gtpc_ 161442

d2gtpd_ 161443

d1a9xa1 18550

d1a9xc1 18551

d1a9xe1 18552

d1a9xg1 18553

d1c30a1 18554

d1c30c1 18555

d1c30e1 18556

d1c30g1 18557

d1cs0a1 18558

d1cs0c1 18559

d1cs0e1 18560

d1cs0g1 18561

d1jdbb1 18574

d1jdbe1 18575

d1jdbh1 18576

d1jdbk1 18577

d1t36a1 106301

d1t36c1 106309

d1t36e1 106317

d1t36g1 106325

d1keea1 68492

d1keec1 68500

d1keee1 68508

d1keeg1 68516

d1c3oa1 18562

d1c3oc1 18563

d1c3oe1 18564

d1c3og1 18565

d1m6va1 74536

d1m6vc1 74544

d1m6ve1 74552

d1m6vg1 74560

d1ce8a1 18566

d1ce8c1 18567

d1ce8e1 18568

d1ce8g1 18569

d1bxra1 18570

d1bxrc1 18571

d1bxre1 18572

d1bxrg1 18573

d1apxa_ 18669

d1apxb_ 18670

d1apxc_ 18671

d1apxd_ 18672

d2ghcx_ 164698

d2ggnx_ 164688

d1oafa_ 86734

d1v0hx_ 108205

d2ghdx_ 164699

d1oaga_ 86735

d2ghex_ 164700

d2ghhx_ 135178

d2ghkx_ 135179

d1iyna_ 90722

d3e2oa_ 174569

d2euta_ 132407

d1z53a_ 124449

d4jmwa_ 192957

d1zbya_ 124878

d2rbtx_ 151857

d4jm5a_ 192961

d1zbza_ 124879

d4jm8a_ 192963

d2y5aa_ 170605

d2as4a_ 127234

d2euqa_ 132404

d4jmba_ 192964

d2aqda_ 127178

d3m27a_ 180733

d3m2ia_ 180744

d3m28a_ 180734

d4jm9a_ 192965

d3e2na_ 174568

d2rbvx_ 151859

d4jm6a_ 192958

d2eupa_ 132403

d2eura_ 132405

d2as3a_ 127233

d2as2a_ 127232

d2as6a_ 127241

d2euua_ 132408

d2euoa_ 132402

d2rc0x_ 151864

d2rbwx_ 151860

d2rbyx_ 151862

d2rc2x_ 151866

d2rbxx_ 151861

d2eusa_ 132406

d2as1a_ 127231

d4jmaa_ 192955

d4jmta_ 192959

d1s73a_ 118876

d1jdra_ 62908

d3exba_ 175287

d4jmsa_ 192960

d1mkra_ 84997

d1mk8a_ 84981

d1mkqa_ 84996

d1ml2a_ 85000

d4jmva_ 192966

d4a7ma_ 193405

d1sbma_ 118929

d4jmza_ 192956

d2rbzx_ 151863

d2ycga_ 170750

d1koka_ 77473

d2rbux_ 151858

d1sdqa_ 118951

d2euna_ 132401

d4jn0a_ 192962

d2anza_ 127064

d1kxma_ 73156

d2cypa_ 18600

d1soga_ 105844

d1ryca_ 18601

d1jcia_ 71632

d1kxna_ 73157

d1stqa_ 106008

d2bcna_ 128299

d2bcnc_ 128301

d1ccaa_ 18602

d1s6va_ 98609

d1s6vc_ 98611

d4ccxa_ 18606

d1dj5a_ 18603

d1cmpa_ 18605

d1dj1a_ 18607

d1krja_ 68852

d1ccla_ 18608

d2ccpa_ 18610

d1cmta_ 18609

d1ds4a_ 59129

d1ccca_ 18611

d1ebea_ 59406

d1dsea_ 59130

d1besa_ 18612

d4ccpa_ 18626

d6ccpa_ 18617

d1ccpa_ 18614

d5ccpa_ 18615

d1dcca_ 18613

d7ccpa_ 18624

d3ccpa_ 18616

d1a2fa_ 18619

d1a2ga_ 18620

d1ccka_ 18622

d1dspa_ 59133

d1cpfa_ 18621

d1aa4a_ 18625

d1cpda_ 18618

d1cpea_ 18623

d1ccga_ 18627

d1ccba_ 18631

d1cpga_ 18628

d1beka_ 18632

d2cepa_ 18633

d1cmua_ 18630

d1beqa_ 18629

d1bj9a_ 18636

d1bepa_ 18634

d1bema_ 18635

d3ccxa_ 18637

d1beja_ 18638

d1cyfa_ 18641

d1dsoa_ 59132

d1ccea_ 18640

d1cmqa_ 18639

d1ccja_ 18644

d1aeea_ 18648

d1aeta_ 18643

d2rc1x_ 151865

d1aesa_ 18642

d1ac8a_ 18645

d1aeda_ 18647

d1aema_ 18654

d1aega_ 18650

d1aeba_ 18646

d1aefa_ 18649

d1aeha_ 18651

d1aeja_ 18652

d1aeqa_ 18655

d1aeka_ 18653

d3r99a_ 195664

d1aeva_ 18656

d1ac4a_ 18657

d1aena_ 18658

d1aeoa_ 18659

d1aeua_ 18660

d2pcca_ 18662

d2pccc_ 18663

d4jb4c_ 196865

d1dsga_ 59131

d1ccia_ 18661

d2b11a_ 127654

d2b11c_ 127655

d1u74a_ 107704

d1u74c_ 107706

d2pcba_ 18664

d2pcbc_ 18665

d1u75a_ 107708

d1u75c_ 107710

d2b0za_ 127651

d2b10a_ 127652

d2b10c_ 127653

d2b12a1 127656

d2gb8a1 134909

d2jtia1 148202

d1bvaa_ 18604

d1arua_ 18589

d1arva_ 18590

d1arwa_ 18591

d1hsra_ 18592

d1gzba_ 18593

d1ck6a_ 18594

d1arxa_ 18595

d1arya_ 18596

d1arpa_ 18597

d1c8ia_ 18598

d1gzaa_ 18599

d1llpa_ 18578

d1b80a_ 18579

d1b80b_ 18580

d1b85a_ 18581

d1b85b_ 18582

d1b82a_ 18583

d1b82b_ 18584

d1qpaa_ 18585

d1qpab_ 18586

d1lgaa_ 18587

d1lgab_ 18588

d1mn2a_ 18666

d1mn1a_ 18667

d1mnpa_ 18668

d1lyca_ 74344

d1lycb_ 74345

d1ly9a_ 74342

d1ly9b_ 74343

d1lyka_ 74346

d1lykb_ 74347

d1h3ja_ 83469

d1h3jb_ 83470

d1ly8a_ 74340

d1ly8b_ 74341

d1bgpa_ 18690

d1gwua_ 83351

d7atja_ 18673

d1h5ma_ 70893

d1hcha_ 70965

d1h5aa_ 70882

d2ylja_ 195122

d1h5ga_ 70887

d1h5ca_ 70883

d1h5la_ 70892

d1h5ka_ 70891

d1h5ja_ 70890

d1h5ia_ 70889

d1h5fa_ 70886

d1h5da_ 70884

d1h55a_ 70877

d1h57a_ 70878

d1h5ha_ 70888

d1h5ea_ 70885

d1gwta_ 83350

d1h58a_ 70879

d1kzma_ 77637

d6atja_ 18674

d1gwoa_ 83349

d2atja_ 18675

d2atjb_ 18676

d1gw2a_ 83341

d1gx2a_ 83360

d1gx2b_ 83361

d3atja_ 18677

d3atjb_ 18678

d1atja_ 18679

d1atjb_ 18680

d1atjc_ 18681

d1atjd_ 18682

d1atje_ 18683

d1atjf_ 18684

d4atja_ 81266

d4atjb_ 81267

d1scha_ 18685

d1schb_ 18686

d1fhfa_ 18687

d1fhfb_ 18688

d1fhfc_ 18689

d1pa2a_ 18693

d1qo4a_ 18694

d1qgja_ 18691

d1qgjb_ 18692

d3m2ha_ 180743

d3m23a_ 180730

d3m25a_ 180731

d3m2ca_ 180738

d3m2fa_ 180741

d3m2ga_ 180742

d3m26a_ 180732

d3m2aa_ 180736

d3m2da_ 180739

d3m2ea_ 180740

d3m2ba_ 180737

d3m29a_ 180735

d4a71a_ 194529

d4a6za_ 192501

d2xila_ 170119

d2xj5a_ 170129

d2v2ea_ 168303

d2xj8a_ 170131

d2v23a_ 168296

d4a78a_ 193406

d2x07a_ 169751

d2x08a_ 169752

d3m5qa_ 180871

d3m8ma_ 180961

d1yyda_ 124212

d1yzra_ 124287

d1yzpa_ 124285

d1yyga_ 124217

d1w4wa_ 120640

d1w4ya_ 120641

d4a5ga_ 194373

d4a5gb_ 194374

d2vcna_ 168461

d2vnzx_ 168741

d3zcga_ 194200

d2wd4a_ 169243

d2vnxx_ 168739

d2xifa_ 170113

d2xi6a_ 170110

d2xiha_ 170118

d2vcsa_ 168462

d2xj6a_ 170130

d2vcfx_ 152916

d2cl4x_ 163436

d2y6aa_ 170648

d2y6ba_ 170649

d3zcha_ 194199

d2vo2x_ 168743

d3zcya_ 194198

d1myp.1 18701

d1myp.2 18702

d1dnu.1 64788

d1dnu.2 64789

d1cxp.1 18695

d1cxp.2 18696

d1d5l.1 64774

d1d5l.2 64775

d1d2v.1 18697

d1d2v.2 18698

d1dnw.1 64790

d1dnw.2 64791

d1d7w.1 64776

d1d7w.2 64777

d1mhl.1 18699

d1mhl.2 18700

d1cvua1 18716

d1cvub1 18717

d3pgha1 18726

d3pghb1 18727

d3pghc1 18728

d3pghd1 18729

d4coxa1 18722

d4coxb1 18723

d4coxc1 18724

d4coxd1 18725

d6coxa1 18730

d6coxb1 18731

d1cx2a1 18718

d1cx2b1 18719

d1cx2c1 18720

d1cx2d1 18721

d1pxxa1 95307

d1pxxb1 95309

d1pxxc1 95311

d1pxxd1 95313

d1ddxa1 18732

d1ddxb1 18733

d1ddxc1 18734

d1ddxd1 18735

d5coxa1 18736

d5coxb1 18737

d5coxc1 18738

d5coxd1 18739

d1q4ga1 95789

d1q4gb1 95791

d2ayla1 127559

d2aylb1 127561

d1eqha1 59491

d1eqhb1 59493

d1eqga1 59487

d1eqgb1 59489

d1ht8a1 61248

d1ht8b1 61250

d1ht5a1 61244

d1ht5b1 61246

d1cqea1 18703

d1cqeb1 18704

d2oyup1 149080

d1diya1 18706

d1ptha1 18705

d1pthb1 118582

d1pgea1 18707

d1pgeb1 18708

d1ebva1 18709

d1fe2a1 59778

d1igza1 66138

d2oyep1 149065

d1igxa1 66136

d1u67a1 107697

d1prha1 18710

d1prhb1 18711

d1pgfa1 18714

d1pgfb1 18715

d1pgga1 18712

d1pggb1 18713

d1mwva1 85161

d1mwva2 85162

d1mwvb1 85163

d1mwvb2 85164

d2b2oa1 127712

d2b2oa2 127713

d2b2ob1 127714

d2b2ob2 127715

d2b2ra1 127720

d2b2ra2 127721

d2b2rb1 127722

d2b2rb2 127723

d1x7ua1 114934

d1x7ua2 114935

d1x7ub1 114936

d1x7ub2 114937

d2b2qa1 127716

d2b2qa2 127717

d2b2qb1 127718

d2b2qb2 127719

d2b2sa1 127724

d2b2sa2 127725

d2b2sb1 127726

d2b2sb2 127727

d1u2ka_ 112983

d1u2la_ 112984

d1u2lb_ 112985

d1u2ja_ 112975

d1u2jb_ 112976

d1u2jc_ 112977

d1u2jd_ 112978

d1u2je_ 112979

d1u2jf_ 112980

d1u2jg_ 112981

d1u2jh_ 112982

d1itka1 71417

d1itka2 71418

d1itkb1 71419

d1itkb2 71420

d2ccaa1 130222

d2ccab1 130224

d2ccda1 130228

d2ccdb1 130230

d1sj2a1 105597

d1sj2b1 105599

d1ub2a1 99141

d1ub2a2 99142

d3hdla_ 177408

d3q3ua_ 184198

d1vq8p1 120203

d1vqop1 120377

d1vqpp1 120406

d1yhqp1 123198

d3cc2p1 156185

d1s72p_ 105335

d1vqmp1 120319

d1jj2o_ 63100

d1vqlp1 120290

d1vqkp1 120261

d1vqnp1 120348

d1yijp1 123313

d1yi2p1 123245

d3ccmp1 156345

d1vq7p1 120174

d1vq9p1 120232

d3cc7p1 156233

d1vq5p1 120116

d3ccep1 156273

d3ccup1 156441

d1vq4p1 120087

d1vq6p1 120145

d3ccvp1 156465

d3cpwo1 156915

d1yitp1 123356

d3cd6p1 156489

d3cclp1 156321

d1yjwp1 123480

d1m90q_ 78855

d3cc4p1 156209

d3ccjp1 156297

d2otlp1 139361

d3ccqp1 156369

d3cmap1 156785

d3ccsp1 156417

d2otjp1 139332

d3ccrp1 156393

d1njiq_ 85808

d2qexp1 150704

d1yjnp1 123448

d1kqso_ 68830

d1yj9p1 123409

d1qvgo_ 96407

d1kc8q_ 84372

d1n8rq_ 85444

d1q82q_ 96144

d1qvfo_ 96377

d3cmep1 156822

d1q81q_ 96114

d1k73q_ 84333

d1k9mq_ 72228

d1kd1q_ 72339

d1k8aq_ 72161

d1m1kq_ 74399

d1q86q_ 96182

d2qa4p1 150189

d1q7yq_ 96080

d1ffkm_ 18740

d1aa7a_ 18741

d1aa7b_ 18742

d1ea3a_ 59398

d1ea3b_ 59399

d2z16a_ 170967

d2z16b_ 170968

d3md2a_ 181015

d3md2b_ 181016

d3md2c_ 181017

d3md2d_ 181018

d2abka_ 18743

d1orna_ 87342

d1orpa_ 87343

d1p59a_ 87794

d1rrqa1 97798

d1rrsa1 97800

d1vrla1 120470

d1muna_ 18744

d1kg2a_ 77378

d1kg5a_ 77381

d1muya_ 18745

d1kg6a_ 77382

d1kg7a_ 77383

d1kg4a_ 77380

d1weia_ 109339

d1kg3a_ 77379

d1kqja_ 72879

d1muda_ 18746

d1wega_ 109338

d1wefa_ 109337

d1ngna_ 85697

d1keaa_ 68491

d4e9ea_ 193598

d3ihoa_ 178323

d4dk9a_ 195549

d1mpga1 18747

d1mpgb1 18748

d3cw7a1 157027

d3cw7b1 157029

d3cw7c1 157031

d3cw7d1 157033

d3cwsa1 157052

d3cwsb1 157054

d3cwsc1 157056

d3cwsd1 157058

d1pvsa1 104329

d1pvsb1 104331

d3cwaa1 157037

d3cwab1 157039

d3cwac1 157041

d3cwad1 157043

d3cvsa1 157011

d3cvsb1 157013

d3cvsc1 157015

d3cvsd1 157017

d3cwta1 157060

d3cwtb1 157062

d3cwtc1 157064

d3cwtd1 157066

d3cvta1 157019

d3cvtb1 157021

d3cvtc1 157023

d3cvtd1 157025

d1diza1 18749

d1dizb1 18750

d3cwua1 157068

d3cwub1 157070

d3cwuc1 157072

d3cwud1 157074

d3d4va1 157302

d3d4vb1 157304

d3d4vc1 157306

d3d4vd1 157308

d1m3qa1 91184

d2noha1 138409

d1ko9a1 68717

d1lwya1 78285

d2noba1 138403

d1m3ha1 91176

d1ebma1 18751

d1lwwa1 78283

d2noea1 138405

d1hu0a1 76723

d1n3aa1 85291

d1n39a1 85289

d1lwva1 78281

d2nofa1 138407

d1yqra1 123897

d1yqma1 123893

d2nola1 138411

d2noza1 138414

d1yqla1 123891

d1yqka1 123889

d1fn7a1 59892

d2i5wa1 137069

d1n3ca1 85293

d1nkua_ 85838

d1lmza_ 74037

d1p7ma_ 94307

d2ofka_ 166681

d2ofkb_ 166682

d2ofia_ 166680

d1pu6a_ 95123

d1pu6b_ 95124

d1pu7a_ 95125

d1pu7b_ 95126

d1pu8a_ 95127

d1pu8b_ 95128

d1xqoa_ 115853

d1xqpa_ 115854

d1xg7a_ 115281

d1xg7b_ 115282

d2jg6a_ 166173

d4aiac_ 196017

d4ai4a_ 196042

d4ai5c_ 196041

d1j09a1 77025

d1n75a1 80224

d2cuza1 130822

d1n78a1 80232

d1n78b1 80234

d2dxia1 131874

d2dxib1 131876

d2cv0a1 130824

d2cv0b1 130826

d1n77a1 80228

d1n77b1 80230

d2cv1a1 130828

d2cv1b1 130830

d1glna1 18752

d2cv2a1 130832

d2cv2b1 130834

d1g59a1 60257

d1g59c1 60259

d1irxa1 71378

d1irxb1 71380

d1rlra1 18753

d1r1ra1 18754

d1r1rb1 18755

d1r1rc1 18756

d5r1ra1 18757

d5r1rb1 18758

d5r1rc1 18759

d6r1ra1 18760

d6r1rb1 18761

d6r1rc1 18762

d7r1ra1 18763

d7r1rb1 18764

d7r1rc1 18765

d2r1ra1 18769

d2r1rb1 18770

d2r1rc1 18771

d3r1ra1 18766

d3r1rb1 18767

d3r1rc1 18768

d4r1ra1 18772

d4r1rb1 18773

d4r1rc1 18774

d1peqa1 104131

d1peoa1 104129

d1pema1 104127

d1peua1 104133

d2bq1e1 128953

d2bq1f1 128955

d1dnpa1 18775

d1dnpb1 18776

d1owla1 93647

d1teza1 112409

d1tezb1 112411

d1tezc1 112413

d1tezd1 112415

d1qnfa1 18777

d1owpa1 93655

d1owoa1 93653

d1owna1 93651

d1owma1 93649

d1iqra1 66275

d1iqua1 71280

d1np7a1 80677

d1np7b1 80679

d1u3da1 107638

d1u3ca1 107636

d2pgda1 18778

d1pgpa1 18780

d1pgna1 18781

d1pgoa1 18779

d1pgqa1 18782

d1pgja1 18783

d1pgjb1 18784

d3obba1 182914

d1vpda1 113951

d2cvza1 130890

d2cvzb1 130892

d2cvzc1 130894

d2cvzd1 130896

d1wp4a1 121135

d1wp4b1 121137

d1wp4c1 121139

d1wp4d1 121141

d1np3a1 85946

d1np3b1 85948

d1np3c1 85950

d1np3d1 85952

d1qmga1 18785

d1qmgb1 18786

d1qmgc1 18787

d1qmgd1 18788

d1yvei1 18789

d1yvej1 18790

d1yvek1 18791

d1yvel1 18792

d1wdka1 109250

d1wdka2 109251

d1wdkb1 109254

d1wdkb2 109255

d2d3ta1 131220

d2d3ta2 131221

d2d3tb1 131224

d2d3tb2 131225

d1wdla1 109262

d1wdla2 109263

d1wdlb1 109266

d1wdlb2 109267

d1wdma1 109274

d1wdma2 109275

d1wdmb1 109278

d1wdmb2 109279

d1f0ya1 18793

d1f0yb1 18794

d3hada1 18799

d3hadb1 18800

d2hdha1 18795

d2hdhb1 18796

d1m76a1 91216

d1m76b1 91218

d1f17a1 18797

d1f17b1 18798

d1il0a1 66189

d1il0b1 66191

d1f14a1 18801

d1f14b1 18802

d1f12a1 18803

d1f12b1 18804

d1m75a1 91212

d1m75b1 91214

d1lsja1 91116

d1lsjb1 91118

d1lsoa1 91120

d1lsob1 91122

d3hdha1 18805

d3hdhb1 18806

d3hdhc1 18807

d1mv8a1 85128

d1mv8b1 85131

d1mv8c1 85134

d1mv8d1 85137

d1muua1 85116

d1muub1 85119

d1muuc1 85122

d1muud1 85125

d1mfza1 84941

d1mfzb1 84944

d1mfzc1 84947

d1mfzd1 84950

d1dlja1 18808

d1dlia1 18809

d1bg6a1 18810

d1txga1 112775

d1txgb1 112777

d1evya1 18811

d1n1ea1 85256

d1n1eb1 85258

d1m66a1 78689

d1jdja1 71635

d1n1ga1 91542

d1evza1 18812

d1m67a1 78691

d1ks9a1 68857

d1yona1 123780

d1yjqa1 123459

d2ofpa1 139052

d2ofpb1 139054

d1i36a1 71108

d1i36b1 71110

d1lj8a3 90392

d1m2wa3 90394

d1m2wb3 90396

d2i76a1 147535

d2i76b1 147537

d1yqga1 145920

d2ag8a1 146053

d2ahra1 126775

d2ahrb1 126777

d2ahrc1 126779

d2ahrd1 126781

d2ahre1 126783

d2amfa1 127009

d2amfb1 127011

d2amfc1 127013

d2amfd1 127015

d2amfe1 127017

d2b0ja1 127639

d2g5ca1 134646

d2g5cb1 134648

d2g5cc1 134650

d2g5cd1 134652

d2pv7a1 149887

d2pv7b1 149889

d2f1ka1 132772

d2f1kb1 132774

d2f1kc1 132776

d2f1kd1 132778

d1zkra1 125209

d1zkrb1 125211

d2ejna1 132279

d2ejnb1 132281

d1puoa1 95134

d1puob1 95136

d1zkra2 125210

d1zkrb2 125212

d2ejna2 132280

d2ejnb2 132282

d1puoa2 95135

d1puob2 95137

d1ccda_ 18816

d1utra_ 18817

d1utrb_ 18818

d1utga_ 18813

d2utga_ 18814

d2utgb_ 18815

d1gaia_ 18819

d1gaha_ 18820

d3glya_ 18822

d1doga_ 18823

d1glma_ 18821

d1agma_ 18824

d2fbaa_ 133239

d2f6da_ 133039

d1ayxa_ 18825

d3eqaa_ 175154

d1kwfa_ 73078

d1is9a_ 76777

d1cema_ 18826

d1clca1 18835

d1ut9a1 99912

d1rq5a1 97730

d1v5da_ 113537

d1v5db_ 113538

d1v5ca_ 113536

d1h12a_ 83447

d1h13a_ 83448

d1xwta1 122410

d1h14a_ 83449

d1xw2a1 122389

d1xwqa_ 122405

d2b4fa_ 127828

d2a8za1 126422

d1ks8a_ 77519

d1ksca_ 77520

d1ksda_ 77521

d1g87a1 83275

d1g87b1 83277

d1ga2a1 83281

d1ga2b1 83283

d1k72a1 84309

d1k72b1 84311

d1kfga1 84387

d1kfgb1 84389

d1tf4a1 18827

d1tf4b1 18828

d1js4a1 18829

d1js4b1 18830

d4tf4a1 18831

d4tf4b1 18832

d3tf4a1 18833

d3tf4b1 18834

d1ia6a_ 76738

d1ia7a_ 76739

d1wzza1 121536

d1g9ga_ 83279

d2qnoa_ 150948

d1fcea_ 18836

d1faea_ 18837

d1g9ja_ 83280

d1fbwa_ 18838

d1f9da_ 18839

d1fboa_ 18840

d1f9oa_ 18841

d1l1ya_ 73486

d1l1yb_ 73487

d1l1yc_ 73488

d1l1yd_ 73489

d1l1ye_ 73490

d1l1yf_ 73491

d1l2aa_ 73493

d1l2ab_ 73494

d1l2ac_ 73495

d1l2ad_ 73496

d1l2ae_ 73497

d1l2af_ 73498

d1wu4a1 121272

d3a3va_ 171708

d1wu6a1 121274

d2drra_ 163666

d2droa_ 163664

d2drqa_ 163665

d2drsa_ 163667

d4el8a_ 196674

d1fp3a_ 18842

d1fp3b_ 18843

d2rgka_ 168118

d2rgkb_ 168119

d2rgkc_ 168120

d2rgkd_ 168121

d2rgke_ 168122

d2rgkf_ 168123

d2zbla_ 171130

d2zblb_ 171131

d2zblc_ 171132

d2zbld_ 171133

d2zble_ 171134

d2zblf_ 171135

d2afaa1 126668

d2afab_ 126669

d2afac_ 126670

d2afad_ 126671

d2afae_ 126672

d2afaf_ 126673

d1v7wa1 108411

d1v7va1 108409

d1v7xa1 108413

d1h54a1 60634

d1h54b1 60636

d1lf6a1 77918

d1lf6b1 77920

d1lf9a1 77922

d1lf9b1 77924

d1ulva1 99571

d1ug9a1 99361

d1nc5a_ 85548

d2gh4a_ 164692

d2d8la_ 131332

d2fv1a_ 164470

d2fv1b_ 164471

d2fuza_ 134193

d1vd5a_ 108518

d2fv0a_ 164468

d2fv0b_ 164469

d2ahfa_ 162809

d2ahfb_ 162810

d2d5ja_ 131271

d2d5jb_ 131272

d2ahga_ 162811

d2ahgb_ 162812

d2p0va1 149142

d3qt9a_ 184618

d3qt3a_ 184617

d2nvpa1 148478

d3p2ca_ 183470

d3p2cb_ 183471

d3on6a_ 183188

d3on6b_ 183189

d2jf4a1 148029

d2jg0a_ 148034

d2wyna_ 169699

d2wynb_ 169700

d2wync_ 169701

d2wynd_ 169702

d2jjba_ 166214

d2jjbb_ 166215

d2jjbc_ 166216

d2jjbd_ 166217

d1wu5a_ 121273

d3qrya_ 184599

d3qryb_ 184600

d3qspa_ 184611

d3qspb_ 184612

d3qpfa_ 184553

d3qpfb_ 184554

d1dl2a_ 18844

d1g6ia_ 83272

d2ri9a_ 152057

d2ri9b_ 152058

d1krea_ 68848

d1kreb_ 68849

d1krfa_ 68850

d1krfb_ 68851

d1kkta_ 68687

d1kktb_ 68688

d2ri8a_ 152055

d2ri8b_ 152056

d1x9da1 121810

d1fo3a_ 18845

d1fmia_ 18846

d1fo2a_ 18847

d1nxca_ 92293

d1hcua_ 65796

d1hcub_ 65797

d1hcuc_ 65798

d1hcud_ 65799

d1qaza_ 18848

d1hv6a_ 61294

d4e1ya_ 192355

d4e1yb_ 192356

d4f13a_ 192411

d4f13b_ 192412

d4f10a_ 192409

d4f10b_ 192410

d1hn0a1 83616

d1rwha1 97984

d1rwaa1 97967

d1rw9a1 97964

d1rwfa1 97978

d1rwga1 97981

d1rwca1 97974

d1cb8a1 18849

d1hmua1 61089

d1hm2a1 61083

d1hm3a1 61086

d1hmwa1 61092

d1n7oa1 80259

d1n7na1 80256

d1ojna1 93137

d1n7pa1 80262

d1lxka1 74331

d1w3ya1 109168

d1egua1 18850

d1ojoa1 93140

d1ojma1 93134

d1c82a1 59079

d1ojpa1 93143

d2brpa1 129005

d1n7ra1 80268

d1f9ga1 59738

d1loha1 74147

d1n7qa1 80265

d2brwa1 129022

d2brwb1 129025

d2brvx1 129019

d1f1sa1 64928

d1i8qa1 66090

d1lxma1 78296

d1x1ia1 121584

d1x1ja1 121587

d2e24a1 131976

d2e22a1 131973

d1x1ha1 121581

d1j0ma1 83910

d1j0na1 83913

d1n1ba1 79799

d1n1bb1 79801

d1n20a1 79832

d1n20b1 79834

d1n24a1 79846

d1n24b1 79848

d1n1za1 79828

d1n1zb1 79830

d1n23a1 79842

d1n23b1 79844

d1n22a1 79838

d1n22b1 79840

d1n21a1 79836

d5eaua1 18851

d5easa1 18852

d1hxaa1 83641

d1hxca1 83643

d1hxga1 83645

d5eata1 18853

d1hx9a1 83639

d1w6ka1 114271

d1w6ka2 114272

d1w6ja1 114269

d1w6ja2 114270

d2sqca1 18854

d2sqca2 18855

d2sqcb1 18856

d2sqcb2 18857

d1umpa1 99619

d1umpa2 99620

d1umpb1 99621

d1umpb2 99622

d1umpc1 99623

d1umpc2 99624

d1sqca1 18858

d1sqca2 18859

d1o6ha1 92568

d1o6ha2 92569

d1o6hb1 92570

d1o6hb2 92571

d1o6hc1 92572

d1o6hc2 92573

d1o6ra1 92583

d1o6ra2 92584

d1o6rb1 92585

d1o6rb2 92586

d1o6rc1 92587

d1o6rc2 92588

d1h37a1 90562

d1h37a2 90563

d1h37b1 90564

d1h37b2 90565

d1h37c1 90566

d1h37c2 90567

d1h3ba1 90580

d1h3ba2 90581

d1h3bb1 90582

d1h3bb2 90583

d1h3bc1 90584

d1h3bc2 90585

d1h35a1 90550

d1h35a2 90551

d1h35b1 90552

d1h35b2 90553

d1h35c1 90554

d1h35c2 90555

d1h36a1 90556

d1h36a2 90557

d1h36b1 90558

d1h36b2 90559

d1h36c1 90560

d1h36c2 90561

d1o6qa1 92577

d1o6qa2 92578

d1o6qb1 92579

d1o6qb2 92580

d1o6qc1 92581

d1o6qc2 92582

d3sqca1 18860

d3sqca2 18861

d3sqcb1 18862

d3sqcb2 18863

d3sqcc1 18864

d3sqcc2 18865

d1gsza1 76329

d1gsza2 76330

d1gszb1 76331

d1gszb2 76332

d1gszc1 76333

d1gszc2 76334

d1h39a1 90568

d1h39a2 90569

d1h39b1 90570

d1h39b2 90571

d1h39c1 90572

d1h39c2 90573

d1o79a1 92602

d1o79a2 92603

d1o79b1 92604

d1o79b2 92605

d1o79c1 92606

d1o79c2 92607

d1h3aa1 90574

d1h3aa2 90575

d1h3ab1 90576

d1h3ab2 90577

d1h3ac1 90578

d1h3ac2 90579

d1h3ca1 90586

d1h3ca2 90587

d1h3cb1 90588

d1h3cb2 90589

d1h3cc1 90590

d1h3cc2 90591

d2h6fb1 136186

d2h6hb1 145283

d1tn6b_ 112528

d1ld8b_ 73839

d1s63b_ 105286

d1mzcb_ 85240

d1sa4b_ 105403

d1ld7b_ 73837

d1jcqb_ 66505

d2h6ib1 145284

d3pz4b_ 184090

d1d8db_ 18866

d1jcrb_ 66507

d1kzpb_ 77641

d1kzob_ 77639

d1jcsb_ 66509

d2r2lb_ 151536

d1o1tb_ 86552

d1tn7b_ 112530

d1tn8b_ 112532

d1o5mb_ 92508

d1o1rb_ 86548

d1o1sb_ 86550

d1ft1b_ 18867

d1sa5b_ 105405

d1n4qb_ 91636

d1n4qd_ 91638

d1n4qf_ 91640

d1n4qh_ 91642

d1n4qj_ 91644

d1n4ql_ 91646

d1s64b_ 105288

d1s64d_ 105290

d1s64f_ 105292

d1s64h_ 105294

d1s64j_ 105296

d1s64l_ 105298

d1n4sb_ 91660

d1n4sd_ 91662

d1n4sf_ 91664

d1n4sh_ 91666

d1n4sj_ 91668

d1n4sl_ 91670

d1qbqb_ 18868

d4gtpb_ 194456

d3dpyb1 157826

d1tnyb_ 112570

d1tnyd_ 112572

d1tnyf_ 112574

d1tnyh_ 112576

d1tnyj_ 112578

d1tnyl_ 112580

d1tnob_ 112546

d1tnod_ 112548

d1tnof_ 112550

d1tnoh_ 112552

d1tnoj_ 112554

d1tnol_ 112556

d1tnub_ 112558

d1tnud_ 112560

d1tnuf_ 112562

d1tnuh_ 112564

d1tnuj_ 112566

d1tnul_ 112568

d1n4pb_ 91624

d1n4pd_ 91626

d1n4pf_ 91628

d1n4ph_ 91630

d1n4pj_ 91632

d1n4pl_ 91634

d2bedb_ 128376

d1tnbb_ 112534

d1tnbd_ 112536

d1tnbf_ 112538

d1tnbh_ 112540

d1tnbj_ 112542

d1tnbl_ 112544

d1n4rb_ 91648

d1n4rd_ 91650

d1n4rf_ 91652

d1n4rh_ 91654

d1n4rj_ 91656

d1n4rl_ 91658

d1tnzb_ 112582

d1tnzd_ 112584

d1tnzf_ 112586

d1tnzh_ 112588

d1tnzj_ 112590

d1tnzl_ 112592

d1d8eb_ 18869

d1ni1b_ 91889

d1nl4b_ 80618

d1fppb_ 18870

d1n95b_ 80334

d1ft2b_ 18871

d1x81b_ 114954

d1n9ab_ 80339

d1n94b_ 80332

d3pz1b_ 184087

d3pz3b_ 184089

d3pz2b_ 184088

d3dssb_ 157841

d3hxfb_ 177902

d4gtvb_ 194457

d3hxcb_ 177899

d3hxdb_ 177900

d3dsub_ 157843

d3dstb_ 157842

d3hxeb_ 177901

d3dsvb_ 157844

d3dsxb_ 157846

d3dswb_ 157845

d1dceb_ 18872

d1dced_ 18873

d3c72b_ 155997

d3hxbb_ 177898

d1ltxb_ 84714

d3eu5b_ 175219

d3euvb_ 175223

d2wy8a_ 169688

d2wy7a_ 169687

d1c3da_ 18874

d1ghqa_ 60521

d2goxa_ 161433

d2goxc_ 161434

d3d5ra_ 157403

d3d5rb_ 157404

d3rj3a_ 195832

d3rj3b_ 195830

d3rj3c_ 195831

d3d5sa_ 157407

d3d5sb_ 157408

d2xqwa_ 170300

d2xqwb_ 170301

d2noja_ 148324

d2nojc_ 148326

d2noje_ 148328

d2nojg_ 148330

d1w2sa1 120604

d1qqfa_ 18875

d1qsja_ 18876

d1qsjb_ 18877

d1qsjc_ 18878

d1qsjd_ 18879

d1hzfa_ 76731

d3oxua_ 183387

d3oxub_ 183388

d3oxuc_ 183389

d1r76a_ 104830

d1gxma_ 76371

d1gxmb_ 76372

d1gxna_ 76373

d1gxoa_ 76374

d2g0da_ 147057

d2g02a1 147056

d1a59a_ 18902

d1csha_ 18880

d1csia_ 18881

d1cssa_ 18882

d1amza_ 18884

d1csra_ 18883

d1al6a_ 18885

d1csca_ 18886

d2csca_ 18887

d4csca_ 18888

d3csca_ 18889

d5ctsa_ 18890

d6ctsa_ 18891

d6csca_ 18892

d6cscb_ 18893

d5csca_ 18894

d5cscb_ 18895

d1k3pa_ 77237

d1k3pb_ 77238

d1owba_ 104037

d1owbb_ 104038

d1owca_ 104039

d1owcb_ 104040

d1nxea_ 86377

d1nxeb_ 86378

d1nxga_ 86379

d1nxgb_ 86380

d3enja_ 175100

d2ctsa_ 18896

d1ctsa_ 18897

d4ctsa_ 18898

d4ctsb_ 18899

d1aj8a_ 18900

d1aj8b_ 18901

d1o7xa_ 81171

d1o7xb_ 81172

d1o7xc_ 81173

d1o7xd_ 81174

d1ioma_ 83698

d1ixea_ 83768

d1ixeb_ 83769

d1ixec_ 83770

d1ixed_ 83771

d2h12a_ 164896

d2h12b_ 164897

d2h12c_ 164898

d2h12d_ 164899

d2h12e_ 164900

d2h12f_ 164901

d1vgma_ 161861

d1vgmb_ 161862

d2ifca_ 165532

d2ifcb_ 165533

d2ifcc_ 165534

d2ifcd_ 165535

d2r9ea_ 168025

d2r9eb_ 168026

d2r9ec_ 168027

d2r9ed_ 168028

d2r26a_ 167930

d2r26b_ 167931

d2r26c_ 167932

d2r26d_ 167933

d4e6ya_ 195720

d3tqga_ 185910

d3tqgb_ 185911

d1vgpa_ 161865

d2p2wa_ 166975

d1io7a_ 18971

d1io7b_ 18972

d1f4ta_ 18973

d1f4tb_ 18974

d1io8a_ 62618

d1io8b_ 62619

d1io9a_ 62620

d1io9b_ 62621

d1f4ua_ 18975

d1f4ub_ 18976

d3b4xa_ 172427

d1ue8a_ 107781

d1n40a_ 79977

d3cxza_ 173533

d4ipsa_ 197028

d4g47a_ 194731

d3g5ha_ 176377

d3g5fa_ 176376

d3cxya_ 173532

d2ij5a_ 137451

d2ij5b_ 137452

d2ij5c_ 137453

d2ij5d_ 137454

d2ij5e_ 137455

d2ij5f_ 137456

d3cxva_ 173530

d3cy1a_ 173535

d4icta_ 197027

d1n4ga_ 79991

d2ij7a_ 137457

d2ij7b_ 137458

d2ij7c_ 137459

d2ij7d_ 137460

d2ij7e_ 137461

d2ij7f_ 137462

d3cxxa_ 173531

d3cy0a_ 173534

d1odoa_ 92783

d1gwia_ 76364

d1gwib_ 76365

d1s1fa_ 112008

d1n97a_ 80335

d1n97b_ 80336

d1wiya_ 114684

d1wiyb_ 114685

d1x8va_ 114968

d1h5za_ 90622

d1u13a_ 107578

d1e9xa_ 18969

d1ea1a_ 18970

d1izoa_ 83851

d1izob_ 83852

d1izoc_ 83853

d2ij2a_ 137449

d2ij2b_ 137450

d4hgha_ 197020

d4dtya_ 195178

d4dtyb_ 195177

d1zo4a_ 162554

d1zo4b_ 162555

d3bena_ 155188

d3benb_ 155189

d1jpza_ 67069

d1jpzb_ 67070

d1zo9a_ 125434

d1zo9b_ 125435

d3kx5a_ 179767

d3kx5b_ 192749

d4dubb_ 195175

d3kx3a_ 179763

d3kx3b_ 179764

d2j1ma_ 137953

d2j1mb_ 137954

d1bu7a_ 18937

d1bu7b_ 18938

d4hgga_ 197021

d4hgfa_ 197023

d4dufb_ 195167

d4dufc_ 195168

d1yqpa_ 162277

d1yqpb_ 162278

d4dudb_ 195169

d3kx4a_ 179765

d3kx4b_ 179766

d1yqoa_ 123895

d1yqob_ 123896

d2ij3a_ 165579

d2ij3b_ 165580

d1p0wa_ 93878

d1p0wb_ 93879

d4duaa_ 195171

d4duab_ 195170

d4duca_ 195166

d3dgia_ 173927

d3dgib_ 173928

d4du2a_ 195173

d4du2b_ 195172

d1p0xa_ 93880

d1p0xb_ 93881

d2nnba_ 166310

d2nnbb_ 166311

d1p0va_ 93876

d1p0vb_ 93877

d2hpda_ 18939

d2hpdb_ 18940

d1jmea_ 66885

d1jmeb_ 66886

d2bmha_ 18941

d2bmhb_ 18942

d2j4sa_ 138005

d2j4sb_ 138006

d2x7ya_ 169926

d2x7yb_ 169927

d1bvya_ 18943

d1bvyb_ 18944

d1zoaa_ 162556

d1zoab_ 162557

d3ekfa_ 175015

d3ekfb_ 175016

d1smia_ 105758

d1smib_ 105759

d1faha_ 18945

d1fahb_ 18946

d2x80a_ 169929

d2x80b_ 169930

d3npla_ 182463

d3nplb_ 182464

d2ij4a_ 165581

d2ij4b_ 165582

d3ekba_ 175010

d3ekbb_ 175011

d3ekda_ 175013

d3ekdb_ 175014

d2uwha_ 168201

d2uwhb_ 168202

d2uwhc_ 168203

d2uwhd_ 168204

d2uwhe_ 168205

d2uwhf_ 168206

d1faga_ 18947

d1fagb_ 18948

d1fagc_ 18949

d1fagd_ 18950

d1smja_ 105760

d1smjb_ 105761

d1smjc_ 105762

d1smjd_ 105763

d2zwua_ 171566

d2zwta_ 171565

d1re9a_ 111789

d2zuia_ 171531

d3l63a_ 179996

d2zawa_ 154294

d2zuha_ 171530

d3l61a_ 179994

d2zuja_ 171532

d2zaxa_ 154295

d3fwga_ 176107

d3fwgb_ 176108

d2h7qa_ 136222

d2a1oa_ 162651

d2a1ob_ 162652

d1qmqa_ 18907

d2fe6a_ 133324

d1dz4a_ 18903

d1dz4b_ 18904

d1phca_ 18905

d1phba_ 18906

d2cppa_ 18908

d1phaa_ 18909

d3p6xa_ 183567

d3oiaa_ 183047

d1phda_ 18913

d1phga_ 18910

d1phfa_ 18911

d2fera_ 133350

d1phea_ 18912

d1k2oa_ 68061

d1k2ob_ 68062

d2z97a_ 154233

d3p6ua_ 183564

d3l62a_ 179995

d3ol5a_ 183109

d1yrda_ 123918

d3p6na_ 183557

d3fwfa_ 176105

d3fwfb_ 176106

d2qbma_ 167508

d2qbna_ 167509

d1t87a_ 106653

d1t87b_ 106654

d2feua_ 133352

d2feub_ 133353

d5cp4a_ 18914

d1t85a_ 106650

d1rf9a_ 111795

d1o76a_ 81123

d1o76b_ 81124

d1t88a_ 106655

d1t88b_ 106656

d2qbla_ 167507

d2a1na_ 162649

d2a1nb_ 162650

d1geka_ 18915

d6cp4a_ 18924

d1dz8a_ 18916

d1dz8b_ 18917

d3cppa_ 18920

d1t86a_ 106651

d1t86b_ 106652

d1dz9a_ 18918

d1dz9b_ 18919

d3fwja_ 176111

d2qboa_ 167510

d1cp4a_ 18921

d3p6pa_ 183559

d1akda_ 18923

d7cppa_ 18922

d3p6qa_ 183560

d1dz6a_ 18925

d1dz6b_ 18926

d6cppa_ 18927

d1iwja_ 71489

d4ek1b_ 194903

d4cp4a_ 18928

d3p6ta_ 183563

d1iwia_ 71488

d8cppa_ 18930

d1gema_ 18929

d3p6sa_ 183562

d3p6ma_ 183556

d1iwka_ 71490

d3p6va_ 183565

d3p6oa_ 183558

d2a1ma_ 126003

d2a1mb_ 126004

d1nooa_ 18931

d1geba_ 18932

d2cp4a_ 18933

d1gjma_ 60576

d4g3ra_ 194877

d4g3rb_ 194876

d3p6wa_ 183566

d5cppa_ 18935

d4cppa_ 18934

d3p6ra_ 183561

d1c8ja_ 59085

d1c8jb_ 59086

d1j51a_ 66393

d1j51b_ 66394

d1j51c_ 66395

d1j51d_ 66396

d2h7ra_ 165004

d3cp4a_ 18936

d1lwla_ 78273

d4jwua_ 197413

d3fwia_ 176110

d1mpwa_ 79389

d1mpwb_ 79390

d1p2ya_ 93961

d2h7sa_ 165005

d2h7sc_ 165006

d1p7ra_ 94317

d1z8oa_ 124722

d1z8pa_ 124723

d1z8qa_ 162403

d1jipa_ 66748

d1jioa_ 66747

d1eupa_ 18967

d1oxaa_ 18965

d1jina_ 66746

d1egya_ 18966

d1jfba_ 66624

d1jfca_ 66625

d1f24a_ 18953

d1f26a_ 18951

d1f25a_ 18952

d1geja_ 18954

d1geia_ 18955

d1ehga_ 18956

d1cmna_ 18958

d1ehfa_ 18957

d1cmja_ 18959

d1cl6a_ 18960

d1ehea_ 18961

d1xqda_ 115841

d2roma_ 18962

d1ulwa_ 113296

d1roma_ 18963

d1geda_ 18964

d1cpta_ 18968

d1q5da_ 95891

d1pkfa_ 94829

d1q5ea_ 95892

d1po5a_ 94963

d1suoa_ 106026

d2q6na1 150061

d2q6nb1 150062

d2q6nc1 150063

d2q6nd1 150064

d2q6ne1 150065

d2q6nf1 150066

d2q6ng1 150067

d1nr6a_ 92085

d1n6ba_ 85359

d1dt6a_ 18977

d1pq2a_ 94987

d1pq2b_ 94988

d1r9oa_ 104878

d1og2a_ 86980

d1og2b_ 86981

d1og5a_ 86982

d1og5b_ 86983

d1tqna_ 107238

d1w0fa_ 108987

d2v0ma1 140050

d2v0mb1 140051

d2v0mc1 140052

d2v0md1 140053

d2j0da1 137897

d2j0db1 137898

d1w0ga_ 108988

d1w0ea_ 108986

d1lfka_ 77926

d1lg9a_ 77953

d1lgfa_ 77954

d1ueda_ 99256

d1uedb_ 99257

d3czha1 157149

d3czhb1 157150

d3c6ga1 155975

d3cbda_ 173119

d3cbdb_ 173120

d2zqxa_ 171442

d2zqxb_ 171443

d2zqxc_ 171444

d2zqja_ 171427

d2zqjb_ 171428

d2zqjc_ 171429

d3nxua_ 182615

d3nxub_ 182616

d3ua1a_ 186195

d4i4ha_ 193089

d4gqsb_ 193379

d4k9ua_ 197419

d2bz9a_ 129554

d2bz9b_ 129555

d2w0ba_ 168976

d2ciba_ 146398

d2ci0a_ 146397

d2w09a_ 168974

d2w0aa_ 168975

d2vkua_ 153229

d1yrca_ 123917

d1uyua_ 119770

d1uyub_ 119771

d2frza_ 134001

d2frzb_ 134002

d2gqxa_ 164806

d2gqxb_ 164807

d2gr6a_ 164808

d2gr6b_ 164809

d1t93a_ 161752

d3tnka_ 194803

d1se6a_ 161712

d1se6b_ 161713

d2d09a_ 163527

d2dkka_ 163646

d2d0ea_ 163529

d2nz5a_ 166462

d2nz5b_ 166463

d2nzaa_ 166464

d2nzab_ 166465

d3tzoa_ 186030

d3tzob_ 186031

d3tnkb_ 194802

d3oo3a_ 183197

d3ejbb_ 161662

d3ejbd_ 161663

d3ejbf_ 161664

d3ejbh_ 161665

d3ejdb_ 161666

d3ejdd_ 161667

d3ejdf_ 161668

d3ejdh_ 161669

d3ejeb_ 161670

d3ejed_ 161671

d3ejef_ 161672

d3ejeh_ 161673

d3jusa_ 196472

d3jusb_ 196471

d3l4da_ 179932

d3l4db_ 179933

d3l4dc_ 179934

d3l4dd_ 179935

d3buja_ 195246

d2z36a_ 171018

d2z36b_ 171019

d3oftc_ 196320

d4dxya_ 194815

d3lxia_ 180624

d3lxib_ 180625

d3lxha_ 180622

d3lxhb_ 180623

d3ofua_ 183000

d3ofub_ 183001

d3ofuc_ 183002

d3ofud_ 183003

d3ofue_ 183004

d3ofuf_ 183005

d2rfba_ 168077

d2rfbb_ 168078

d2rfbc_ 168079

d3a4ga_ 171719

d3a51a_ 171742

d3a51b_ 171743

d3a51c_ 171744

d3a51d_ 171745

d3a51e_ 171746

d3a50a_ 171737

d3a50b_ 171738

d3a50c_ 171739

d3a50d_ 171740

d3a50e_ 171741

d3a4za_ 171732

d3a4zb_ 171733

d3a4zc_ 171734

d3a4zd_ 171735

d3a4ze_ 171736

d3vrma_ 196871

d4do1d_ 193595

d3awma_ 172366

d3awpa_ 172367

d3awqa_ 172368

d3vm4a_ 195396

d3vnoa_ 194020

d3vooa_ 194019

d3vtja_ 197280

d3abaa_ 196461

d3e5ja_ 193622

d3abba_ 171915

d3e5la_ 196107

d4fxba_ 193381

d3tywa_ 194967

d3tywb_ 194966

d2zbxa_ 171142

d2zbya_ 171143

d3cv9a_ 173493

d2zbza_ 171144

d3cv8a_ 173492

d3khma_ 196512

d3k1oa_ 196522

d3gw9d_ 196507

d3g1qd_ 196529

d3tika_ 194971

d3tikb_ 194969

d3tikc_ 194970

d3tikd_ 194972

d3b99a_ 194762

d3b99b_ 194763

d1rqta_ 97771

d1rqtb_ 97772

d1rqva1 97777

d1rqvb1 97779

d1rqua1 97773

d1rqub1 97775

d2gyc31 135846

d2gyc51 135848

d2gya31 135834

d2gya51 135836

d1zavu1 145963

d1zavv1 145964

d1zavw1 145965

d1zavx1 145966

d1zavy1 145967

d1zavz1 145968

d1dd3a1 19031

d1dd3b1 19032

d1dd3c_ 19033

d1dd3d_ 19034

d1zaxu1 145977

d1zaxv1 145978

d1zaxw1 145979

d1zaxx1 145980

d1zaxy1 145981

d1zaxz1 145982

d1zawu1 145970

d1zawv1 145971

d1zaww1 145972

d1zawx1 145973

d1zawy1 145974

d1zawz1 145975

d1dd4a1 19035

d1dd4b1 19036

d1dd4c_ 19037

d1dd4d_ 19038

d1yl3i1 123580

d1yl3j1 123582

d1iiea_ 19039

d1iieb_ 19040

d1iiec_ 19041

d1aora1 19042

d1aorb1 19043

d1b25a1 19044

d1b25b1 19045

d1b25c1 19046

d1b25d1 19047

d1b4na1 19048

d1b4nb1 19049

d1b4nc1 19050

d1b4nd1 19051

d1d2ta_ 19052

d1eoia_ 59481

d1eoib_ 59482

d1eoic_ 59483

d1iw8a_ 76866

d1iw8b_ 76867

d1iw8c_ 76868

d1iw8d_ 76869

d1iw8e_ 76870

d1iw8f_ 76871

d1qi9a_ 19053

d1qi9b_ 19054

d1qhba_ 19055

d1qhbb_ 19056

d1qhbc_ 19057

d1qhbd_ 19058

d1qhbe_ 19059

d1qhbf_ 19060

d1up8a_ 99710

d1up8b_ 99711

d1up8c_ 99712

d1up8d_ 99713

d1vnsa_ 19061

d1idqa_ 62300

d1vnia_ 19064

d1vnea_ 19063

d1vnha_ 19062

d1vnca_ 19065

d1vnga_ 19066

d1idua_ 62303

d1vnfa_ 19067

d1wb9a1 120833

d1w7aa1 109218

d1w7ab1 109222

d1e3ma1 19075

d1e3mb1 19076

d1oh6a1 92987

d1oh6b1 92991

d1wbda1 120841

d1wbba1 120837

d1oh7a1 92995

d1oh7b1 92999

d1ng9a1 80480

d1ng9b1 80484

d1oh8a1 93003

d1oh8b1 93007

d1oh5a1 92979

d1oh5b1 92983

d1ewqa1 19069

d1ewqb1 19070

d1fw6a1 19071

d1fw6b1 19072

d1nnea1 85895

d1nneb1 85899

d1ewra1 19073

d1ewrb1 19074

d1f5na1 19077

d1dg3a1 19078

d1bvp11 19079

d1bvp21 19080

d1bvp31 19081

d1bvp41 19082

d1bvp51 19083

d1bvp61 19084

d2btvc1 19086

d2btvd1 19087

d2btve1 19089

d2btvf1 19090

d2btvg1 19092

d2btvh1 19093

d2btvi1 19095

d2btvj1 19096

d2btvp1 19085

d2btvq1 19088

d2btvr1 19091

d2btvs1 19094

d2btvt1 19097

d1qhda1 63324

d1uf2c1 99291

d1uf2d1 99293

d1uf2e1 99295

d1uf2f1 99297

d1uf2g1 99299

d1uf2h1 99301

d1uf2i1 99303

d1uf2j1 99305

d1uf2p1 99307

d1uf2q1 99309

d1uf2r1 99311

d1uf2s1 99313

d1uf2t1 99315

d1xa6a1 115030

d2ngrb_ 19105

d1grnb_ 19106

d1f7ca_ 19107

d1tx4a_ 19098

d1ow3a_ 87485

d1rgpa_ 19099

d1am4a_ 19100

d1am4b_ 19101

d1am4c_ 19102

d1pbwa_ 19103

d1pbwb_ 19104

d1nf1a_ 19110

d1wera_ 19108

d1wq1g_ 19109

d1nvus_ 86276

d2ii0a_ 137425

d1nvvs_ 86279

d1nvws_ 86282

d1xd2c_ 115144

d1bkds_ 19111

d1nvxs_ 86285

d1xdva2 115181

d1xdvb2 115184

d1xd4a2 115150

d1xd4b2 115153

d1jdha_ 66549

d1t08a_ 112190

d1g3ja_ 19114

d1g3jc_ 19115

d1qz7a_ 96618

d1luja_ 78225

d1th1a_ 106905

d1th1b_ 106906

d1jpwa_ 67059

d1jpwb_ 67060

d1jpwc_ 67061

d1m1ea_ 78399

d1i7wa_ 61909

d1i7wc_ 61911

d4ev9a_ 196944

d3bcta_ 19112

d3ouxa_ 183309

d1i7xa_ 61913

d1i7xc_ 61915

d2bcta_ 19113

d3ouwa_ 183308

d1jppa_ 67043

d1jppb_ 67044

d1wa5c_ 114433

d1z3ha1 124404

d1z3hb1 124405

d1q1sc_ 95606

d3l3qa_ 179913

d1y2ac_ 162146

d1pjmb_ 94764

d1pjnb_ 94765

d1iala_ 19116

d1q1tc_ 95607

d1iq1c_ 66260

d1ejli_ 19117

d1ejyi_ 19118

d2bkub1 128712

d1qgra_ 19119

d2p8qa_ 149314

d1ibrb_ 19120

d1ibrd_ 19121

d1qgka_ 19122

d1f59a_ 19123

d1f59b_ 19124

d1o6pa_ 86636

d1o6pb_ 86637

d1o6oa_ 92574

d1o6ob_ 92575

d1o6oc_ 92576

d1m5ns_ 78653

d2q5da1 150048

d2q5db1 150049

d1gcja_ 19125

d1gcjb_ 19126

d1ukla_ 99493

d1uklb_ 99494

d1wa5b_ 114432

d1ee4a_ 19128

d1ee4b_ 19129

d1bk5a_ 19130

d1bk5b_ 19131

d1ee5a_ 19132

d1un0a_ 99641

d1un0b_ 99642

d1bk6a_ 19133

d1bk6b_ 19134

d1qbkb_ 19127

d3ea5b_ 158074

d3ea5d_ 158075

d3nd2a_ 182155

d2c1ta_ 129642

d2c1tb_ 129643

d3tx7a_ 185995

d2z5ka_ 171064

d3tpma_ 185902

d1v18a_ 119816

d2c1ma_ 163227

d4i5la_ 197094

d1b3ua_ 19135

d1b3ub_ 19136

d2ie3a_ 137290

d3k7va_ 179170

d3dw8a_ 157908

d3dw8d_ 157910

d2ie4a_ 137291

d3k7wa_ 179172

d2pkga1 139708

d2pkgb1 139709

d2nppa1 138444

d2nppd1 138446

d2nyma1 138821

d2nymd1 138824

d2nyla1 138815

d2nyld1 138818

d3fgaa_ 175767

d1u6gc_ 113066

d1b89a_ 19138

d1utca1 99914

d1utcb1 99916

d1c9la1 19139

d1c9lb1 19140

d1bpoa1 19143

d1bpob1 19144

d1bpoc1 19145

d1c9ia1 19141

d1c9ib1 19142

d1lrva_ 19146

d1e8ya1 19152

d1e8za1 19153

d1he8a1 19154

d1e7ua1 19147

d1e8xa1 19148

d1e7va1 19149

d1e90a1 19150

d1e8wa1 19151

d3b7sa1 154940

d3b7rl1 154937

d3choa1 156641

d2vj8a1 153183

d2r59a1 151583

d3b7ux1 154946

d1hs6a1 61233

d1h19a1 70847

d1gw6a1 83342

d3chqa1 156647

d1sqma1 105941

d3chpa1 156644

d3b7ta1 154943

d3chra1 156650

d3chsa1 156653

d1m8za_ 78834

d1ib2a_ 62141

d1m8wa_ 78828

d1m8wb_ 78829

d3q0na_ 184124

d3q0nb_ 184125

d1m8xa_ 78830

d1m8xb_ 78831

d3q0la_ 184120

d3q0lb_ 184121

d3bsxa_ 155529

d3bsxb_ 155530

d3q0pa_ 184128

d3q0pb_ 184129

d1m8ya_ 78832

d1m8yb_ 78833

d3q0oa_ 184126

d3q0ob_ 184127

d3q0ma_ 184122

d3q0mb_ 184123

d3bsba_ 155522

d3bsbb_ 155523

d3q0qa_ 184130

d3q0ra_ 184131

d3q0sa_ 184132

d2yjya_ 170828

d2yjyb_ 170829

d3gvoa_ 177041

d3gvta_ 177044

d3gvtb_ 177045

d1ho8a_ 61107

d2vgla_ 161577

d2vglb_ 153035

d2jkrb_ 166247

d2jkre_ 166248

d1oxja2 87518

d1h6ka1 60675

d1h6ka2 60676

d1h6ka3 60677

d1h6kb1 60678

d1h6kb2 60679

d1h6kb3 60680

d1h6kc1 60681

d1h6kc2 60682

d1h6kc3 60683

d1h2vc1 76592

d1h2vc2 76593

d1h2vc3 76594

d1h2tc1 76580

d1h2tc2 76581

d1h2tc3 76582

d1n52a1 79999

d1n52a2 80000

d1n52a3 80001

d1h2ua1 76584

d1h2ua2 76585

d1h2ua3 76586

d1h2ub1 76587

d1h2ub2 76588

d1h2ub3 76589

d1n54a1 80003

d1n54a2 80004

d1n54a3 80005

d1ug3a1 119671

d1ug3a2 119672

d1hu3a_ 19137

d2nsza1 138560

d2iona1 137575

d2iosa_ 137576

d2iola1 137573

d2iolb_ 137574

d1uw4b_ 100072

d1uw4d_ 100074

d1paqa_ 94413

d1upka_ 99758

d1upla_ 99759

d1uplb_ 99760

d3gnia_ 176776

d2wtka_ 169618

d2wtkd_ 169619

d3zhpa_ 196874

d1oyza_ 93775

d1te4a_ 106794

d1t06a_ 106194

d1t06b_ 106195

d2b6ca1 127983

d2b6cb_ 127984

d3zbob_ 197128

d1rz4a2 105132

d1w9ca_ 114410

d1w9cb_ 114411

d2jaka1 138240

d2nppb1 138445

d2nppe1 138447

d2nymb1 138822

d2nyme1 138825

d2nylb1 138816

d2nyle1 138819

d1xqra1 122241

d1xqsa1 122243

d1xqsb1 122244

d1z3xa1 124416

d1z3ya1 124418

d1y6ia1 122660

d2bnxa1 128861

d1z2cb1 124377

d1z2cd1 124379

d2bapa1 128242

d2bapb1 128243

d3eg5b_ 161659

d3eg5d_ 161660

d1xm9a1 122142

d2i9ca1 147566

d3bvsa_ 172871

d3jxya_ 178918

d3jx7a_ 178899

d3jy1a_ 178920

d3jxza_ 178919

d2bpta_ 128951

d3k49a_ 179062

d3k49c_ 179063

d3k49e_ 179064

d2bkud_ 128713

d3juia_ 178836

d1re0b_ 97318

d1ku1a_ 72996

d1ku1b_ 72997

d1bc9a_ 19180

d2r09a1 151492

d2r09b1 151494

d2r0da1 151496

d2r0db1 151498

d1r8se_ 97248

d1r8me_ 97242

d1s9de_ 98751

d1pbva_ 19179

d1r8qe_ 97245

d1r8qf_ 97246

d1xsza1 116005

d1xszb1 116007

d1xt0b_ 116009

d4a4pa_ 194436

d4a4pb_ 194435

d3ltla_ 180557

d3ltlb_ 180558

d3l8na_ 196460

d1lsha1 74242

d1qsaa1 19182

d1qtea1 19183

d1slya1 19184

d2h6fa1 136185

d2h6ha1 136188

d1tn6a_ 112527

d1ld8a_ 73838

d1s63a_ 105285

d2h6ga1 136187

d1mzca_ 85239

d1sa4a_ 105402

d1ld7a_ 73836

d1jcqa_ 66504

d2f0ya1 132681

d2h6ia1 136189

d1d8da_ 19185

d1jcra_ 66506

d1kzpa_ 77640

d1kzoa_ 77638

d1jcsa_ 66508

d2r2la_ 151535

d1o1ta_ 86551

d1tn7a_ 112529

d1tn8a_ 112531

d1o5ma_ 92507

d1o1ra_ 86547

d1o1sa_ 86549

d1ft1a_ 19186

d1sa5a_ 105404

d1n4qa_ 91635

d1n4qc_ 91637

d1n4qe_ 91639

d1n4qg_ 91641

d1n4qi_ 91643

d1n4qk_ 91645

d1s64a_ 105287

d1s64c_ 105289

d1s64e_ 105291

d1s64g_ 105293

d1s64i_ 105295

d1s64k_ 105297

d1n4sa_ 91659

d1n4sc_ 91661

d1n4se_ 91663

d1n4sg_ 91665

d1n4si_ 91667

d1n4sk_ 91669

d1qbqa_ 19187

d3dpya1 157825

d1tnya_ 112569

d1tnyc_ 112571

d1tnye_ 112573

d1tnyg_ 112575

d1tnyi_ 112577

d1tnyk_ 112579

d1tnoa_ 112545

d1tnoc_ 112547

d1tnoe_ 112549

d1tnog_ 112551

d1tnoi_ 112553

d1tnok_ 112555

d1tnua_ 112557

d1tnuc_ 112559

d1tnue_ 112561

d1tnug_ 112563

d1tnui_ 112565

d1tnuk_ 112567

d1n4pa_ 91623

d1n4pc_ 91625

d1n4pe_ 91627

d1n4pg_ 91629

d1n4pi_ 91631

d1n4pk_ 91633

d2beda_ 128375

d1tnba_ 112533

d1tnbc_ 112535

d1tnbe_ 112537

d1tnbg_ 112539

d1tnbi_ 112541

d1tnbk_ 112543

d1n4ra_ 91647

d1n4rc_ 91649

d1n4re_ 91651

d1n4rg_ 91653

d1n4ri_ 91655

d1n4rk_ 91657

d1tnza_ 112581

d1tnzc_ 112583

d1tnze_ 112585

d1tnzg_ 112587

d1tnzi_ 112589

d1tnzk_ 112591

d1d8ea_ 19188

d1ni1a_ 91888

d1nl4a_ 80617

d1fppa_ 19189

d1n95a_ 80333

d1ft2a_ 19190

d1x81a_ 114953

d1n9aa_ 80338

d1n94a_ 80331

d1dcea1 19191

d1dcec1 19192

d1ltxa1 84711

d2o8pa1 148679

d3efza1 158144

d1o9da_ 86689

d1o9ca_ 86688

d1o9ea_ 86690

d1o9fa_ 86691

d3m50a_ 180856

d1a4oa_ 19194

d1a4ob_ 19195

d1a4oc_ 19196

d1a4od_ 19197

d1a37a_ 19198

d1a37b_ 19199

d1a38a_ 19200

d1a38b_ 19201

d2o02a_ 148541

d2o02b_ 148542

d1qjba_ 19202

d1qjbb_ 19203

d1qjaa_ 19204

d1qjab_ 19205

d3cu8a_ 173464

d3cu8b_ 173465

d3nkxa_ 182351

d3nkxb_ 182352

d3rdha_ 184886

d3rdhb_ 184887

d3rdhc_ 184888

d3rdhd_ 184889

d1ib1a_ 62133

d1ib1b_ 62134

d1ib1c_ 62135

d1ib1d_ 62136

d2v7da_ 152717

d2v7db_ 152718

d2v7dc_ 152719

d2v7dd_ 152720

d3iqua_ 178564

d3iqja_ 178557

d3mhra_ 181230

d3iqva_ 178565

d3lw1a_ 180598

d3p1na_ 183462

d3p1sa_ 183467

d3p1ra_ 183466

d3p1qa_ 183465

d2br9a_ 163150

d3uala_ 186197

d3p1oa_ 183463

d3p1pa_ 183464

d3o8ia_ 182876

d4e2ea_ 195789

d4dnkb_ 196005

d2c1na_ 129640

d2c1nb_ 129641

d2bq0a_ 163144

d2bq0b_ 163145

d1ywta_ 162319

d1ywtb_ 162320

d2c74a_ 163269

d2c74b_ 163270

d2b05a_ 162955

d2b05b_ 162956

d2b05c_ 162957

d2b05d_ 162958

d2b05e_ 162959

d2b05f_ 162960

d2c1ja_ 129638

d2c1jb_ 129639

d2c23a_ 163231

d1yz5a_ 162341

d1yz5b_ 162342

d3axyc_ 172373

d3axyd_ 172374

d3axyi_ 172375

d3axyj_ 172376

d3e6yb_ 196599

d2o98a_ 138950

d2o98b_ 138951

d1ihga1 62380

d1iipa1 62451

d1kt0a1 77525

d1kt1a1 77528

d1p5qa1 104068

d1p5qb1 104070

d1p5qc1 104072

d1qz2a1 104663

d1qz2b1 104665

d1qz2c1 104667

d1elwa_ 19207

d1elwb_ 19208

d1elra_ 19209

d3eska_ 175194

d1iyga_ 76947

d1xnfa_ 115580

d1xnfb_ 115581

d2pqra_ 161508

d2pqrb_ 149793

d2pqna_ 149792

d1y8ma1 122757

d1nzna_ 92382

d1pc2a_ 94427

d1zu2a1 125662

d1hh8a_ 61042

d1e96b_ 19211

d1w3ba_ 109149

d1w3bb_ 109150

d1fcha_ 19212

d1fchb_ 19213

d1hxia_ 61366

d3cv0a_ 173488

d3cvna_ 173506

d3cvpa_ 173507

d3cvla_ 173503

d1tjca_ 112440

d1tjcb_ 112441

d1a17a_ 19206

d1wao11 120813

d1wao21 120815

d1wao31 120817

d1wao41 120819

d2buga1 129203

d2fbna1 133254

d1ya0a1 122785

d2c2la1 129672

d2c2lb1 129674

d2c2lc1 129676

d2c2ld1 129678

d1qqea_ 19210

d2q7fa1 150085

d1wm5a_ 121028

d2v5fa_ 168346

d2vyia1 153706

d3r9ab_ 184856

d3r9ad_ 184858

d1ya0b_ 122786

d2j9qa_ 138152

d2j9qb_ 161474

d2c0ma_ 129596

d2c0mb_ 129597

d2c0mc_ 129598

d2c0mf_ 129599

d2vgxa1 153046

d1hz4a_ 65960

d2ff4a2 133368

d2ff4b2 133371

d2feza2 133358

d2awia2 127436

d2awib2 127438

d2awic2 127440

d2awid2 127442

d2awie2 127444

d2awif2 127446

d2awig2 127448

d2awih2 127450

d2awii2 127452

d2awij2 127454

d2awik2 127456

d2awil2 127458

d2aw6a2 127409

d2aw6b2 127411

d2grla2 135561

d2grlb2 135563

d2grlc2 135565

d2grld2 135567

d2axza2 127537

d2axzb2 127539

d2axzc2 127541

d2axzd2 127543

d2grma2 147162

d2grmb2 147164

d2axva2 127525

d2axvb2 127527

d2axvc2 127529

d2axvd2 127531

d2i6ha1 147528

d3ckca_ 156736

d3ckcb_ 156737

d3ck8a_ 156730

d3ck8b_ 156731

d3ck7a1 156726

d3ck7b_ 156727

d3ck7c_ 156728

d3ck7d_ 156729

d3ck9a_ 156732

d3ck9b_ 156733

d3ckba_ 156734

d3ckbb_ 156735

d3ejna1 158180

d3cgha1 156618

d2onda1 148899

d2ondb_ 148900

d2ooea1 148923

d2hr2a1 147369

d2hr2b_ 147370

d2hr2c_ 147371

d2hr2d_ 147372

d2hr2e_ 147373

d2hr2f_ 147374

d3gzsa_ 177116

d3gzsb_ 177117

d4f53b_ 194987

d3sgha_ 185372

d3sghb_ 185373

d1inza_ 62610

d1eyha_ 19214

d1h0aa_ 76434

d1edua_ 19215

d1ujka_ 99457

d1ujkb_ 99458

d1jwga_ 71911

d1jwgb_ 71912

d1jwfa_ 71910

d1py1a_ 95315

d1py1b_ 95316

d1py1c_ 95317

d1py1d_ 95318

d1ujja_ 99455

d1ujjb_ 99456

d1mhqa_ 84973

d1mhqb_ 84974

d1juqa_ 67322

d1juqb_ 67323

d1juqc_ 67324

d1juqd_ 67325

d1lf8a_ 73879

d1lf8b_ 73880

d1lf8c_ 73881

d1lf8d_ 73882

d1jpla_ 67027

d1jplb_ 67028

d1jplc_ 67029

d1jpld_ 67030

d1dvpa1 19216

d1elka_ 19217

d1elkb_ 19218

d3g2sa_ 176298

d3g2sb_ 176299

d3g2ta_ 176300

d3g2tb_ 176301

d3g2va_ 176304

d3g2vb_ 176305

d3g2ua_ 176302

d3g2ub_ 176303

d3g2wa_ 176306

d3g2wb_ 176307

d1hx8a2 90363

d1hx8b2 90365

d1hf8a2 90355

d1hg5a2 90361

d1hg2a2 90359

d1hfaa2 90357

d1szaa_ 106142

d1szab_ 106143

d1szac_ 106144

d1sz9a_ 106139

d1sz9b_ 106140

d1sz9c_ 106141

d2bf0x_ 128402

d3ldza_ 180200

d3ldzb_ 180201

d3ldzc_ 180202

d3ldzd_ 180203

d3ag3e_ 172095

d3ag3r_ 172107

d2dyre_ 131904

d2dyrr_ 131918

d1v54e_ 100326

d1v54r_ 100340

d3ag2e_ 172071

d3ag2r_ 172083

d3abme_ 171973

d3abmr_ 171985

d1v55e_ 100354

d1v55r_ 100368

d2eije_ 132144

d2eijr_ 132158

d3abke_ 171925

d3abkr_ 171937

d3ag4e_ 172119

d3ag4r_ 172131

d3able_ 171949

d3ablr_ 171961

d2eile_ 132200

d2eilr_ 132214

d3ag1e_ 172047

d3ag1r_ 172059

d3asor_ 196284

d2eike_ 132172

d2eikr_ 132186

d2dyse_ 131932

d2dysr_ 131946

d2occe_ 19224

d2occr_ 19225

d1ocre_ 19226

d1ocrr_ 19227

d2zxwe_ 171575

d2zxwr_ 171587

d2eime_ 132228

d2eimr_ 132242

d2eine_ 132256

d2einr_ 132270

d1occe_ 19228

d1occr_ 19229

d1ocze_ 19230

d1oczr_ 19231

d1ocoe_ 19232

d1ocor_ 19233

d2grrb_ 147168

d2grob_ 147165

d2grqb_ 147167

d2grnb1 145220

d2grpb_ 147166

d2iy0c1 145552

d1z5sc1 144737

d2io2c1 145535

d2io3c1 145537

d1kpsb_ 68787

d1kpsd_ 68789

d3uipc_ 186332

d3uioc_ 186330

d3uinc_ 186328

d1k8kg_ 68313

d1u2vg_ 112996

d1tyqg_ 112849

d2p9ig_ 139589

d3uleg_ 193999

d2p9kg_ 139597

d2p9lg_ 139605

d2p9sg_ 139629

d2p9ug_ 139637

d3dxkg_ 174330

d2p9ng_ 139613

d3dxmg_ 174334

d2p9pg_ 139621

d3ukug_ 194000

d3rseg_ 185145

d4fhrb_ 194792

d1qc7a_ 18456

d1qc7b_ 18457

d1lkvx_ 73980

d3ajca_ 172208

d3usya_ 186403

d3usyb_ 186404

d1l5ja1 73592

d1l5jb1 73595

d1j1ja_ 90762

d1j1jb_ 90763

d1j1jc_ 90764

d1j1jd_ 90765

d1keya_ 72389

d1keyb_ 72390

d1keyc_ 72391

d1keyd_ 72392

d3qb5a_ 184314

d3qb5b_ 184315

d3qb5c_ 184316

d1ldja2 73848

d1u6ga2 113063

d1ldka_ 73851

d2hyec2 145415

d2pfva_ 167170

d2b7ma1 128046

d2d2sa1 131188

d2b1ea_ 162964

d2wzka_ 196510

d1klxa_ 77440

d1ouva_ 93570

d1rw2a_ 97961

d1q2za_ 95656

d1wy6a_ 121432

d1n8va_ 85457

d1n8vb_ 85458

d1kx9a_ 77602

d1kx9b_ 77603

d1n8ua_ 85456

d1kx8a_ 77601

d1k19a_ 77226

d1n4ka1 79992

d2a9ua1 126450

d2a9ub_ 126451

d2ajaa1 126856

d1yvra1 124116

d1yvpa1 124108

d2i91a1 137105

d2i91b1 137107

d2ouxa1 149031

d2yvxa1 153781

d2yvxb1 153784

d2yvxc1 153787

d2yvxd1 153790

d3bnea1 155436

d3bnba1 155430

d1f8na1 59703

d1ygea1 19234

d3bnda1 155434

d3bnca1 155432

d1fgta1 59830

d1fgra1 59828

d1fgoa1 59824

d1fgqa1 59826

d1fgma1 65009

d1y4ka1 122622

d2sbla1 118670

d2sblb1 19235

d1rrha1 111922

d1n8qa1 85422

d1ik3a1 66172

d1jnqa1 84183

d1hu9a1 83631

d1no3a1 85916

d1rova1 97674

d1rrla1 111926

d1rrlb1 111928

d1lnha1 19237

d2p0ma1 149136

d2p0mb1 149138

d1loxa1 19238

d1c1ka_ 19239

d1ui5a2 107857

d1ui5b2 107859

d1ui6a2 107861

d1ui6b2 107863

d1t56a2 106429

d1u9na2 113247

d1u9oa2 113249

d2fx0a2 134270

d3loca2 180466

d3locb2 180468

d3locc2 180470

d3locd2 180472

d1rkta2 97624

d1rktb2 97626

d1vi0a2 100721

d1vi0b2 100723

d1jt6a2 67248

d1jt6b2 67250

d1jt6d2 67252

d1jt6e2 67254

d1rkwa2 104968

d1rkwb2 104970

d1rkwd2 104972

d1rkwe2 104974

d2hq5a2 147330

d2hq5b2 147332

d2hq5d2 147334

d2hq5e2 147336

d3btla2 155583

d3btlb2 155585

d3btld2 155587

d3btle2 155589

d3btia2 155567

d3btib2 155569

d3btid2 155571

d3btie2 155573

d2dtza2 146579

d2dtzb2 146581

d2dtzd2 146583

d2dtze2 146585

d3bt9a2 155551

d3bt9b2 155553

d3bt9d2 155555

d3bt9e2 155557

d1jusa2 67327

d1jusb2 67329

d1jusd2 67331

d1juse2 67333

d1jtxa2 67285

d1jtxb2 67287

d1jtxd2 67289

d1jtxe2 67291

d2g0ea2 147059

d2g0eb2 147061

d2g0ed2 147063

d2g0ee2 147065

d3btja2 155575

d3btjb2 155577

d3btjd2 155579

d3btje2 155581

d1rpwa2 105037

d1rpwb2 105039

d1rpwc2 105041

d1rpwd2 105043

d1qvta2 104603

d1qvtb2 104605

d1qvtd2 104607

d1qvte2 104609

d1qvua2 104611

d1qvub2 104613

d1qvud2 104615

d1qvue2 104617

d3btca2 155559

d3btcb2 155561

d3btcd2 155563

d3btce2 155565

d1jt0a2 71851

d1jt0b2 71853

d1jt0c2 71855

d1jt0d2 71857

d2gbya2 147103

d2gbyb2 147105

d2gbyd2 147107

d2gbye2 147109

d1juma2 67305

d1jumb2 67307

d1jumd2 67309

d1jume2 67311

d1jupa2 67315

d1jupb2 67317

d1jupd2 67319

d1jupe2 67321

d1jtya2 67293

d1jtyb2 67295

d1jtyd2 67297

d1jtye2 67299

d2gena2 135063

d2fd5a2 133292

d2gfna2 135102

d2gfnb2 135104

d2d6ya2 131314

d2d6yb2 131316

d2oi8a2 148779

d2g3ba2 134559

d2g3bb2 134561

d2g7sa2 134738

d2g7ga2 134733

d2hkua2 147303

d2i10a2 136961

d2np3a2 138421

d2np3b2 138423

d3c07a2 155808

d2hyja2 147457

d2id3a2 137262

d2id3b2 137264

d2g7la2 134735

d1sgma2 105538

d1sgmb2 105540

d1t33a2 106296

d1t33b2 106298

d2vkva2 153231

d2vkea2 153222

d2o7oa2 138931

d2fj1a2 133542

d2tcta2 19240

d1bjza2 19242

d2x9da2 169969

d2trta2 19241

d1orka2 19244

d1a6ia2 19243

d1bj0a2 19245

d1bjya2 19246

d1bjyb2 19247

d1qpia2 19249

d2iu5a2 147802

d2fq4a2 133938

d1zk8a2 125178

d2o7ta2 148671

d2zoya2 171392

d2zoyb2 171394

d2zoza2 154741

d1z0xa2 124335

d1z0xb2 124337

d2fbqa2 133257

d2np5a2 138425

d2np5b2 138427

d2np5c2 138429

d2np5d2 138431

d2id6a2 147625

d2ieka2 147648

d1z77a2 124589

d1zkga2 125195

d1zkgb2 125197

d1t7ra_ 106635

d1t7fa_ 106625

d1t7ma_ 106626

d1t7ta_ 106638

d1t79a_ 106619

d1t74a_ 106613

d1t76a_ 106618

d1t73a_ 106612

d2pnua_ 149706

d2ax6a_ 127479

d2am9a_ 126999

d3b66a_ 154877

d2amba_ 127001

d2q7ka_ 167462

d2ax9a_ 127481

d2ax8a_ 162943

d1t65a_ 119159

d2q7ia_ 150086

d3rlla_ 185021

d2q7ja_ 150087

d2axaa_ 127482

d2q7la_ 167463

d2pita_ 149517

d1xowa_ 115717

d2ax7a_ 127480

d3b5ra_ 154851

d2ao6a_ 127068

d3b65a_ 154876

d2amaa_ 127000

d3rlja_ 185020

d2pipl_ 149514

d1gs4a_ 76328

d2oz7a_ 139441

d3b67a_ 154878

d3b68a_ 154879

d2piva_ 149519

d1t63a_ 119158

d2pira_ 149516

d2pioa_ 149513

d2hvca_ 147418

d1xq3a_ 115820

d2piua_ 149518

d2yhda_ 170786

d4hlwa_ 194075

d1t5za_ 119157

d2piqa_ 149515

d1e3ga_ 59192

d2z4ja_ 154050

d2pkla_ 149604

d2pixa_ 149521

d2piwa_ 149520

d3l3xa_ 179923

d3l3za_ 179924

d2qpya_ 151217

d3g0wa_ 176241

d2ihqa_ 137423

d1i37a_ 61583

d1i38a_ 61584

d1xnna_ 122197

d2nw4a1 138709

d1osha_ 93496

d3flia_ 175890

d3l1ba_ 179853

d3p89a_ 193652

d3ruua_ 185192

d3hc5a_ 177363

d3p88a_ 196194

d1osva_ 93500

d1osvb_ 93501

d1ot7a_ 93505

d1ot7b_ 93506

d1r1kd_ 96819

d1r20d_ 96845

d2qzoa_ 167892

d2qzob_ 167893

d1xpca_ 115738

d2qxsa_ 167872

d2qxsb_ 167873

d2qsea_ 167792

d2qseb_ 167793

d2ioga_ 147744

d1xp6a_ 115734

d2b1za_ 162969

d2b1zb_ 162970

d2p15a_ 166933

d2p15b_ 166934

d2b1va_ 162967

d2b1vb_ 162968

d2yjab_ 191839

d1xp1a_ 115721

d1xp9a_ 115737

d1l2ia_ 73503

d1l2ib_ 73504

d2poga_ 149721

d2pogb_ 161507

d1yima_ 162210

d2qa8a_ 167490

d2qa8b_ 167491

d3os8a_ 183253

d3os8b_ 183254

d3os8c_ 183255

d3os8d_ 183256

d2ayra1 127569

d1x7ra_ 162039

d2r6ya_ 151623

d2r6yb_ 151624

d2r6wa_ 151621

d2r6wb_ 151622

d2qaba_ 167492

d2qabb_ 167493

d2ouza_ 139378

d3erta_ 19292

d3erda_ 19293

d3erdb_ 19294

d2qr9a_ 167777

d2qr9b_ 167778

d1sj0a_ 98893

d3uuca_ 194773

d3uucb_ 194775

d3uucc_ 194772

d3uucd_ 194774

d2faia_ 164311

d2faib_ 164312

d2qgta_ 167573

d2qgtb_ 167574

d2q70a_ 150072

d2q70b_ 161524

d1yina_ 162211

d1zkya_ 162506

d1zkyb_ 162507

d2b23a_ 162971

d2b23b_ 162972

d3os9a_ 183257

d3os9b_ 183258

d3os9c_ 183259

d3os9d_ 183260

d1qkta_ 19295

d3dt3a_ 157848

d3dt3b_ 157849

d3osaa_ 183261

d3osab_ 183262

d3osac_ 183263

d3osad_ 183264

d2g5oa_ 164583

d2g5ob_ 164584

d1uoma_ 88497

d1gwqa_ 76366

d1gwqb_ 76367

d1gwra_ 76368

d1gwrb_ 76369

d2qgwa_ 167585

d2qgwb_ 167586

d2qe4a_ 167557

d2qe4b_ 167558

d2qa6a_ 167488

d2qa6b_ 167489

d1pcga_ 94432

d1pcgb_ 94433

d2qxma_ 167870

d2qxmb_ 167871

d2qh6a_ 167591

d2qh6b_ 167592

d2g44a_ 164578

d2g44b_ 164579

d1a52a_ 19296

d1a52b_ 19297

d1errb_ 19299

d2ioka_ 147747

d2iokb_ 147748

d1r5ka_ 111706

d1r5kb_ 111707

d1r5kc_ 111708

d2q6ja_ 167457

d2q6jb_ 167458

d1x7ea_ 162037

d1x7eb_ 162038

d2i0ja_ 165352

d2i0jb_ 165353

d2i0jc_ 165354

d2i0jd_ 165355

d1erea_ 19300

d1ereb_ 19301

d1erec_ 19302

d1ered_ 19303

d1eree_ 19304

d1eref_ 19305

d1g50a_ 65149

d1g50b_ 65150

d1g50c_ 65151

d1qkua_ 19306

d1qkub_ 19307

d1qkuc_ 19308

d3olla_ 183140

d3ollb_ 183141

d1qkma_ 19309

d3omqa_ 183183

d3omqb_ 183184

d2nv7a_ 148458

d2nv7b_ 148459

d2yjdb_ 196256

d3ompa_ 183181

d3ompb_ 183182

d1yyea1 124213

d1yyeb1 124214

d3olsa_ 183145

d3olsb_ 183146

d1x76a_ 121777

d1x76b_ 121778

d3omoa_ 183179

d3omob_ 183180

d1x7ja_ 121788

d1x7jb_ 121789

d1u3ra_ 119522

d1u3rb_ 119523

d1zafa_ 162431

d1zafb_ 162432

d2z4ba_ 154048

d2z4bb_ 154049

d1x7ba_ 121784

d1x7bb_ 121785

d1x78a_ 121779

d1x78b_ 121780

d2giua_ 135252

d1u3sa_ 119524

d1u3sb_ 119525

d1u9ea_ 119635

d1u9eb_ 119636

d1u3qa_ 119518

d1u3qb_ 119519

d1u3qc_ 119520

d1u3qd_ 119521

d2fsza_ 164457

d2fszb_ 164458

d2i0ga_ 136950

d2i0gb_ 136951

d1yy4a1 124205

d1yy4b1 124206

d2qtua_ 151355

d2qtub_ 151356

d1l2ja_ 73505

d1l2jb_ 73506

d1ndea_ 80415

d1qkna_ 19310

d1hj1a_ 65843

d3e7ca_ 174713

d3e7cb_ 174714

d1nhza_ 85727

d3clda_ 173313

d3cldb_ 173314

d3bqda_ 155498

d1m2za_ 84768

d1m2zd_ 84769

d1p93a_ 87986

d1p93b_ 87987

d1p93c_ 87988

d1p93d_ 87989

d1pzla_ 104393

d1m7wa_ 84869

d1m7wb_ 84870

d1m7wc_ 84871

d1m7wd_ 84872

d1lv2a_ 78230

d1pdua_ 88034

d1pdub_ 88035

d2gpua_ 135493

d2gpoa_ 135484

d1s9qa_ 105388

d1s9qb_ 105389

d2ewpa_ 132488

d2ewpb_ 132489

d2ewpc_ 132490

d2ewpd_ 132491

d2ewpe_ 132492

d1s9pa_ 105384

d1s9pb_ 105385

d1s9pc_ 105386

d1s9pd_ 105387

d2gp7a_ 135452

d2gp7b_ 135453

d2gp7c_ 135454

d2gp7d_ 135455

d1tfca_ 106850

d1tfcb_ 106851

d2gppa_ 135485

d2gppb_ 135486

d2gpva_ 135494

d2gpvb_ 135495

d2gpvc_ 135496

d2gpvd_ 161435

d2gpve_ 161436

d2gpvf_ 161437

d1kv6a_ 77546

d1kv6b_ 77547

d1vjba_ 108629

d1vjbb_ 108630

d1xvpb_ 116091

d1xvpd_ 116093

d1xv9b_ 116081

d1xv9d_ 116083

d1xnxa_ 115667

d1xnxb_ 115668

d1xlse_ 115451

d1xlsf_ 115452

d1xlsg_ 115453

d1xlsh_ 115454

d3v3ea_ 194612

d2qw4a1 151397

d1pk5a_ 88139

d1pk5b_ 88140

d1zh7a_ 162474

d1zh7b_ 162475

d1ovla_ 87455

d1ovlb_ 87456

d1ovlc_ 87457

d1ovld_ 87458

d1ovle_ 87459

d1ovlf_ 87460

d1n83a_ 80276

d1s0xa_ 98313

d1nq7a_ 92047

d1k4wa_ 72068

d1n4ha_ 91618

d1uhlb_ 107850

d2aclb1 144801

d2acld_ 144802

d2aclf_ 144803

d2aclh_ 144804

d1pq9a_ 95004

d1pq9b_ 95005

d1pq9c_ 95006

d1pq9d_ 95007

d1upva_ 113398

d1pq6a_ 94998

d1pq6b_ 94999

d1pq6c_ 95000

d1pq6d_ 95001

d1pqca_ 95009

d1pqcb_ 95010

d1pqcc_ 95011

d1pqcd_ 95012

d1upwa_ 113399

d1p8da_ 87941

d1p8db_ 87942

d3vi8a_ 194751

d2p54a_ 139497

d3kdua_ 179296

d3kdub_ 179297

d2znna_ 171377

d3g8ia_ 176419

d1i7ga_ 71121

d4bcra_ 197142

d4bcrb_ 197141

d2rewa_ 151991

d2npaa_ 166324

d2npac_ 166325

d3feia_ 175727

d3et1b_ 193734

d1k7la_ 68268

d1k7lc_ 68269

d1k7le_ 68270

d1k7lg_ 68271

d3kdta_ 179294

d3kdtb_ 179295

d1kkqa_ 68679

d1kkqb_ 68680

d1kkqc_ 68681

d1kkqd_ 68682

d3u9qa_ 186194

d3b1ma_ 191859

d3v9ta_ 186470

d3v9va_ 186471

d3et3a_ 196608

d3lmpa_ 180397

d2xkwb_ 191836

d3kmga_ 179448

d3kmgd_ 179449

d3b0qb_ 196255

d4em9a_ 193928

d2q5sa_ 150053

d2q5sb_ 150054

d3feja_ 175728

d3noaa_ 182438

d3noab_ 182439

d3v9ya_ 186472

d3vsoa_ 192976

d3vsob_ 192977

d2hfpa_ 136388

d2gtka_ 135654

d2q59a_ 150043

d2q59b_ 150044

d2q61a_ 150055

d2q61b_ 150056

d2zvta_ 171552

d2zvtb_ 191692

d3b0rb_ 196253

d3ty0a_ 185999

d3ty0b_ 186000

d3r5na_ 191960

d3sz1a_ 192093

d3sz1b_ 192094

d3vjha_ 192205

d3vjhb_ 192206

d3pbaa_ 183629

d3pbab_ 183630

d2prga_ 19311

d2prgb_ 19312

d3g9ea_ 176442

d1fm9d_ 19313

d3an4b_ 191847

d2q5pa_ 150051

d2q5pb_ 150052

d3po9a_ 183908

d3po9b_ 183909

d3an3b_ 191846

d2i4ja_ 137049

d2i4jb_ 137050

d1fm6d_ 19316

d1fm6x_ 19317

d3vspa_ 192978

d3vspb_ 192975

d1prga_ 19314

d1prgb_ 19315

d2q8sa_ 150131

d2q8sb_ 150132

d1rdtd_ 111788

d3ia6a_ 178202

d3ia6b_ 178203

d2poba_ 149717

d2pobb_ 149718

d2p4ya_ 149221

d2p4yb_ 149222

d2q6sa_ 150070

d2q6sb_ 150071

d3hoda_ 177729

d3hodb_ 177730

d3et0b_ 196609

d2i4za_ 137053

d2i4zb_ 137054

d2i4pa_ 137051

d2i4pb_ 137052

d3oswa_ 183272

d3oswb_ 183273

d1k74d_ 68252

d3qt0a_ 191953

d3osia_ 183267

d3osib_ 183268

d4emab_ 193929

d3r8ia_ 184847

d3r8ib_ 184848

d1i7ia_ 71125

d1i7ib_ 71126

d2g0ha_ 134493

d2g0hb_ 134494

d2zk2a_ 171269

d2zk2b_ 171270

d3r8aa_ 195834

d3r8ab_ 195833

d2znoa_ 171378

d2znob_ 171379

d1knua_ 77457

d1knub_ 77458

d1zeoa_ 124992

d1zeob_ 124993

d3vjia_ 192207

d3vjib_ 192208

d2g0ga_ 134491

d2g0gb_ 134492

d3h0ad_ 177126

d2zk0a_ 171265

d2zk0b_ 171266

d3k8sa_ 179198

d3k8sb_ 179199

d3d6da_ 173713

d3d6db_ 173714

d4e4qa_ 192841

d4e4qb_ 192598

d2zk6a_ 171277

d2zk6b_ 171278

d2zk5a_ 171275

d2zk5b_ 171276

d3b3ka_ 172412

d3b3kb_ 172413

d3prga_ 19318

d2q6ra_ 150068

d2q6rb_ 150069

d4e4ka_ 192599

d4e4kb_ 192600

d2zk4a_ 171273

d2zk4b_ 171274

d2zk3a_ 171271

d2zk3b_ 171272

d1nyxa_ 86431

d1nyxb_ 86432

d3ho0a_ 177723

d3ho0b_ 177724

d1wm0x_ 109404

d2zk1a_ 171267

d2zk1b_ 171268

d4prga_ 19319

d4prgb_ 19320

d4prgc_ 19321

d4prgd_ 19322

d2qmva1 150898

d3tkma_ 194999

d2b50a_ 127872

d2b50b_ 127873

d3gz9a_ 177105

d2awha_ 127433

d2awhb_ 127434

d3peqa_ 183687

d3peqb_ 183688

d3et2a_ 175209

d3et2b_ 175210

d3sp9b_ 196308

d2gwxa_ 19323

d2gwxb_ 19324

d1y0sa_ 122512

d1y0sb_ 122513

d2znqa_ 171380

d2znqb_ 171381

d3gwxa_ 19325

d3gwxb_ 19326

d2q5ga_ 150050

d2q5gb_ 161523

d3dy6a_ 157935

d3dy6b_ 157936

d1gwxa_ 19327

d1gwxb_ 19328

d1nrla_ 92091

d1nrlb_ 92092

d1m13a_ 84746

d1ilga_ 62544

d1skxa1 118970

d1ilha_ 62545

d2o9ia_ 166590

d2o9ib_ 166591

d1sqna_ 105944

d1sqnb_ 105945

d1sr7a_ 105957

d1sr7b_ 105958

d1a28a_ 19290

d1a28b_ 19291

d1zuca_ 125666

d1zucb_ 125667

d3kbaa_ 179231

d3kbab_ 179232

d2ovha_ 139380

d3hq5a_ 177772

d3hq5b_ 177773

d3d90a_ 173760

d3d90b_ 173761

d2ovma_ 139381

d1e3ka_ 59193

d1e3kb_ 59194

d1dkfb_ 19281

d1xapa_ 115038

d1xdkb_ 115165

d1xdkf_ 115167

d1fcya_ 19282

d1fd0a_ 76164

d1fcza_ 19283

d1fcxa_ 19284

d1exaa_ 19285

d1exxa_ 19286

d2lbda_ 19287

d3lbda_ 19288

d4lbda_ 19289

d2p1ta_ 149165

d1mzna_ 79704

d1mznc_ 79705

d1mzne_ 79706

d1mzng_ 79707

d1mvca_ 79499

d1mv9a_ 79498

d3e94a_ 174766

d3fuga_ 176063

d3oapa_ 182906

d3pcua_ 183653

d3fc6a_ 175671

d3fc6c_ 175672

d2p1ua_ 149166

d2p1va_ 149167

d3kwya_ 179762

d1g5ya_ 60294

d1g5yb_ 60295

d1g5yc_ 60296

d1g5yd_ 60297

d1fm9a_ 19274

d3ozja_ 183419

d3ozjc_ 183420

d1fm6a_ 19277

d1fm6u_ 19278

d1rdta_ 111787

d1fbya_ 19275

d1fbyb_ 19276

d3fala_ 175615

d3falc_ 175616

d1k74a_ 68251

d1xvpa_ 116090

d1xvpc_ 116092

d1g1ua_ 60204

d1g1ub_ 60205

d1g1uc_ 60206

d1g1ud_ 60207

d1xv9a_ 116080

d1xv9c_ 116082

d3h0aa_ 177125

d2acla_ 126559

d2aclc_ 126560

d2acle_ 126561

d2aclg_ 126562

d2zy0a_ 171603

d2zy0c_ 171604

d3uvvb_ 195566

d1lbda_ 19279

d3r5mc_ 196054

d3nsqa_ 182524

d3nsqb_ 182525

d3nspa_ 182522

d3nspb_ 182523

d1xlsa_ 115447

d1xlsb_ 115448

d1xlsc_ 115449

d1xlsd_ 115450

d3r29a_ 184779

d3r29b_ 184780

d3a9ea_ 171885

d1dkfa_ 19280

d1xdka_ 115164

d1xdke_ 115166

d1h9ua_ 70923

d1h9ub_ 70924

d1h9uc_ 70925

d1h9ud_ 70926

d1uhla_ 107849

d3d24a1 157216

d3d24c1 157217

d2pjla_ 167198

d2pjlb_ 167199

d1xb7a_ 109537

d3ilza_ 178392

d3jzba_ 178928

d1nava_ 85500

d3gwsx_ 177060

d1n46a_ 85315

d1n46b_ 85316

d1nq0a_ 86005

d2pinb_ 195024

d1xzxx_ 116274

d1naxa_ 85501

d3jzca_ 178929

d1nq2a_ 86007

d3imya_ 178429

d1q4xa_ 95833

d1nq1a_ 86006

d1y0xx_ 116316

d1nuoa_ 86198

d1bsxa_ 19330

d1bsxb_ 19331

d1hg4a_ 19333

d1hg4b_ 19334

d1hg4c_ 19335

d1hg4d_ 19336

d1hg4e_ 19337

d1hg4f_ 19338

d1g2na_ 60227

d3ixpa_ 178680

d1r1ka_ 96818

d1r20a_ 96844

d3b0ta_ 196254

d3az3a_ 172392

d1ie9a_ 62318

d3az1a_ 172390

d1ie8a_ 62317

d3az2a_ 172391

d1db1a_ 19329

d4g2ia_ 193772

d3cs6a_ 156955

d3m7ra_ 180913

d3kpza_ 179545

d3cs4a_ 156954

d1s19a_ 98339

d1s0za_ 98326

d3ax8a_ 172369

d3a2ja_ 171660

d2o4ja1 138908

d2zmia_ 154691

d1rkga_ 97611

d2o4ra1 138913

d1rjka_ 97571

d2zl9a_ 154626

d3afra_ 172042

d2zlaa_ 154627

d2zlca_ 154629

d2zxna_ 171567

d1rkha_ 97612

d2zmha_ 154690

d1rk3a_ 97591

d2zmja_ 154692

d3a2ha_ 171659

d3ry9a_ 185201

d3ry9b_ 185202

d1xiua_ 162079

d1xiub_ 162080

d3eybd_ 175304

d3uvva_ 195568

d3ixpd_ 178681

d3zqta_ 186517

d3vhva_ 186511

d3a40x_ 171712

d2e2ra_ 146654

d3zr7a_ 186527

d3zr7b_ 186528

d3a3zx_ 171711

d2zkca1 171281

d3plza_ 183842

d3plzb_ 183843

d2hb7a_ 136307

d2w8ya_ 169112

d2w8yb_ 169113

d3zrba_ 186531

d3zrbb_ 186532

d2zbsa_ 154323

d2h79a_ 165003

d1txia_ 119387

d3a78a_ 171844

d1z95a_ 162417

d2hama_ 136290

d2hasa_ 136294

d3l03a_ 179833

d3l03b_ 179834

d2hara_ 136293

d1yuca_ 162303

d1yucb_ 162304

d2hb8a_ 136308

d3g8oa_ 176423

d3g8ob_ 176424

d4apub_ 195553

d2aa6b_ 193194

d3zraa_ 186529

d3zrab_ 186530

d2zasa_ 154285

d2aa2a_ 193193

d1zgya_ 125064

d3olfa_ 183134

d3olfc_ 183135

d2fvja_ 134203

d3omka_ 183173

d3omkc_ 183174

d4a4wa_ 192809

d4a4wb_ 192491

d3beja_ 155186

d3bejb_ 155187

d3q95a_ 184282

d3q95b_ 184283

d4a4va_ 192492

d4a4vb_ 192810

d2yfea_ 191837

d2yfeb_ 191838

d3q97a_ 184284

d3q97b_ 184285

d3k6pa_ 179135

d2vsra_ 153529

d2vsrb_ 153530

d2a3ia_ 162659

d3k22a_ 178972

d3k22b_ 178973

d3fs1a_ 176008

d3okia_ 183075

d3okic_ 183076

d1xqca_ 122233

d1xqcb_ 122234

d1xqcc_ 122235

d1xqcd_ 122236

d3adwa_ 172021

d3adwb_ 172022

d3adxa_ 172023

d3adxb_ 172024

d3fxva_ 176130

d3omma_ 183175

d3ommc_ 183176

d1y9ra_ 162170

d1y9rb_ 162171

d2hwqa_ 147427

d2hwqb_ 161457

d2f4ba_ 132918

d2f4bb_ 132919

d2j4aa_ 194388

d3fura_ 176066

d3d57a_ 173689

d3d57b_ 192713

d2jj3a_ 148127

d2jj3b_ 148128

d2ylpa_ 170856

d3l0ea_ 179839

d3oofa_ 183203

d3oofc_ 183204

d3adsa_ 172013

d3adsb_ 172014

d2h77a_ 165002

d2vv1a_ 153607

d2vv1b_ 161587

d3vhwa_ 193906

d3adva_ 172019

d3advb_ 172020

d2abia_ 162747

d2abib_ 162748

d2abic_ 162749

d2ylqa_ 170857

d2atha_ 127291

d2athb_ 127292

d2xyja_ 170482

d2xyjb_ 170483

d2vv4a_ 153612

d2vv4b_ 161588

d3ooka_ 183205

d3ookc_ 183206

d2yata_ 170710

d3cs8a_ 156958

d3gn8a_ 176774

d3gn8b_ 176775

d2vsta_ 153531

d2vstb_ 153532

d2bawa_ 128248

d2bawb_ 128249

d3gbka_ 176499

d3gbkb_ 176500

d3hzfa_ 177962

d2oaxa_ 166608

d2oaxb_ 166609

d2oaxc_ 166610

d2oaxd_ 166611

d2oaxe_ 166612

d2oaxf_ 166613

d2hwra_ 147428

d2hwrb_ 161458

d3hm1a_ 177686

d3hm1b_ 177687

d3kfca_ 179327

d3kfcb_ 179328

d3kfcc_ 179329

d3kfcd_ 179330

d2vv0a_ 153605

d2vv0b_ 153606

d1ya3a_ 162174

d1ya3b_ 162175

d1ya3c_ 162176

d3okha_ 183074

d2gl8a_ 164756

d2gl8b_ 164757

d2gl8c_ 164758

d2gl8d_ 164759

d3cwda_ 157049

d3cwdb_ 157050

d3d5fa_ 157397

d3d5fb_ 157398

d2yloa_ 170855

d3dcta_ 157538

d1yoka_ 162275

d2xyxa_ 170490

d2xyxb_ 170491

d1zdua_ 162451

d3dcua_ 157539

d3adua_ 172017

d3adub_ 172018

d3adta_ 172015

d3adtb_ 172016

d2vv2a_ 153608

d2vv2b_ 153609

d2om9a_ 139142

d2om9b_ 139143

d2om9c_ 139144

d2om9d_ 139145

d2vv3a_ 153610

d2vv3b_ 153611

d4is8a_ 197320

d2j14a_ 137935

d2j14b_ 137936

d3cdsa_ 173164

d3cdsb_ 173165

d3h52a_ 177189

d3h52b_ 177190

d3h52c_ 177191

d3h52d_ 177192

d1xj7a_ 122031

d3cdpa_ 173160

d3cdpb_ 173161

d3mnpa_ 181456

d3mnoa_ 181455

d3mnea_ 181445

d2j7ya_ 138130

d2zfxa_ 171200

d2j7xa_ 138129

d2r40a_ 151571

d2nxxa_ 166448

d2nxxb_ 166449

d2nxxc_ 166450

d2nxxd_ 166451

d2nxxe_ 166452

d2nxxf_ 166453

d2nxxg_ 166454

d2nxxh_ 166455

d2xhsa_ 170097

d3l0la_ 179842

d3l0lb_ 179843

d3b0wb_ 193702

d1olpa1 93320

d1olpb1 93322

d1olpc1 93324

d1olpd1 93326

d1gyga1 70753

d1gygb1 70755

d1ca1a1 19340

d1qmda1 19341

d1qmdb1 19342

d1khoa1 72489

d1khob1 72491

d1qm6a1 19343

d1qm6b1 19344

d1ah7a_ 19339

d2huca_ 165275

d1p6da_ 94167

d1p5xa_ 94151

d2ffza_ 164343

d2fgna_ 164355

d1p6ea_ 94168

d1ak0a_ 19345

d3snga_ 195005

d4dj4a_ 194387

d4dj4b_ 194385

d3w52a_ 196873

d1n5ua1 85339

d1n5ua2 85340

d1n5ua3 85341

d2bxka1 129431

d2bxka2 129432

d1ha2a1 60866

d1ha2a2 60867

d1ha2a3 60868

d1gnia1 65397

d1gnia2 65398

d1gnia3 65399

d1h9za1 60863

d1h9za2 60864

d1h9za3 60865

d1hk4a1 83533

d1hk4a2 83534

d1hk4a3 83535

d2bxia1 129429

d2bxia2 129430

d2bxaa2 129402

d2bxab2 129404

d2vuea2 153545

d2vueb2 153547

d2bxqa1 129443

d2bxqa2 129444

d1gnja1 65400

d1gnja2 65401

d1gnja3 65402

d1bm0a1 19348

d1bm0a2 19349

d1bm0a3 19350

d1bm0b1 19351

d1bm0b2 19352

d1bm0b3 19353

d1e7aa1 19360

d1e7aa2 19361

d1e7aa3 19362

d1e7ab1 19363

d1e7ab2 19364

d1e7ab3 19365

d1hk1a1 83524

d1hk1a2 83525

d1hk1a3 83526

d1ao6a1 19354

d1ao6a2 19355

d1ao6a3 19356

d1ao6b1 19357

d1ao6b2 19358

d1ao6b3 19359

d1e7ha1 19366

d1e7ha2 19367

d1e7ha3 19368

d1hk5a1 83536

d1hk5a2 83537

d1hk5a3 83538

d1hk2a1 83527

d1hk2a2 83528

d1hk2a3 83529

d1bj5a1 19369

d1bj5a2 19370

d1bj5a3 19371

d1e7ca1 19378

d1e7ca2 19379

d1e7ca3 19380

d1e7fa1 19375

d1e7fa2 19376

d1e7fa3 19377

d1e7ea1 19372

d1e7ea2 19373

d1e7ea3 19374

d2i2za2 137020

d1hk3a1 83530

d1hk3a2 83531

d1hk3a3 83532

d2vdba1 152970

d2vdba2 152971

d1e7ga1 19381

d1e7ga2 19382

d1e7ga3 19383

d2i30a2 137022

d1e7ia1 19384

d1e7ia2 19385

d1e7ia3 19386

d2bxga2 129426

d2bxgb2 129428

d2bx8a2 129398

d2bx8b2 129400

d1tf0a1 106822

d1tf0a2 106823

d1tf0a3 106824

d1e7ba1 19390

d1e7ba2 19391

d1e7ba3 19392

d1e7bb1 19393

d1e7bb2 19394

d1e7bb3 19395

d2bxea2 129418

d2bxeb2 129420

d2bxfa2 129422

d2bxfb2 129424

d1bkea1 19387

d1bkea2 19388

d1bkea3 19389

d1e78a1 59336

d1e78a2 59337

d1e78a3 59338

d1e78b1 59339

d1e78b2 59340

d1e78b3 59341

d2bxda1 129413

d2bxda2 129414

d2bxdb1 129415

d2bxdb2 129416

d2bxba1 129405

d2bxba2 129406

d2bxbb1 129407

d2bxbb2 129408

d1o9xa1 86714

d1o9xa2 86715

d1o9xa3 86716

d2bxca1 129409

d2bxca2 129410

d2bxcb1 129411

d2bxcb2 129412

d1uora1 19396

d1uora2 19397

d1uora3 19398

d1ysxa1 123985

d2esgc1 132323

d2esgc2 132324

d1kxpd1 73160

d1kxpd2 73161

d1kxpd3 73162

d1kw2a1 73071

d1kw2a2 73072

d1kw2a3 73073

d1kw2b1 73074

d1kw2b2 73075

d1kw2b3 73076

d1ma9a1 78887

d1ma9a2 78888

d1ma9a3 78889

d1j78a1 66397

d1j78a2 66398

d1j78a3 66399

d1j78b1 66400

d1j78b2 66401

d1j78b3 66402

d1j7ea1 66403

d1j7ea2 66404

d1j7ea3 66405

d1j7eb1 66406

d1j7eb2 66407

d1j7eb3 66408

d1lota1 74161

d1lota2 74162

d1lota3 74163

d1q5na_ 95901

d1re5a_ 104911

d1re5b_ 104912

d1re5c_ 104913

d1re5d_ 104914

d1f1oa_ 19433

d1dofa_ 19429

d1dofb_ 19430

d1dofc_ 19431

d1dofd_ 19432

d1c3ca_ 19425

d1c3cb_ 19426

d1c3ua_ 19427

d1c3ub_ 19428

d1tjva_ 107054

d1tjvb_ 107055

d1tjvc_ 107056

d1tjvd_ 107057

d1tjwa_ 107058

d1tjwb_ 107059

d1tjwc_ 107060

d1tjwd_ 107061

d1k7wa_ 72130

d1k7wb_ 72131

d1k7wc_ 72132

d1k7wd_ 72133

d1tjua_ 107050

d1tjub_ 107051

d1tjuc_ 107052

d1tjud_ 107053

d1hy0a_ 61392

d1hy0b_ 61393

d1u16a_ 112952

d1auwa_ 19416

d1auwb_ 19417

d1auwc_ 19418

d1auwd_ 19419

d1hy1a_ 61394

d1hy1b_ 61395

d1hy1c_ 61396

d1hy1d_ 61397

d1dcna_ 19420

d1dcnb_ 19421

d1dcnc_ 19422

d1dcnd_ 19423

d1u15a_ 112948

d1u15b_ 112949

d1u15c_ 112950

d1u15d_ 112951

d1tj7a_ 112438

d1tj7b_ 112439

d1k62a_ 68213

d1k62b_ 68214

d1aosa_ 19414

d1aosb_ 19415

d1i0aa_ 61479

d1i0ab_ 61480

d1i0ac_ 61481

d1i0ad_ 61482

d1yfma_ 19413

d1fura_ 19403

d1furb_ 19404

d1fuoa_ 19411

d1fuob_ 19412

d1fuqa_ 19405

d1fuqb_ 19406

d2fusa_ 19409

d2fusb_ 19410

d1fupa_ 19407

d1fupb_ 19408

d1kq7a_ 72866

d1kq7b_ 72867

d1vdka_ 100561

d1vdkb_ 100562

d3r6qa_ 184817

d3r6qb_ 184818

d3r6qc_ 184819

d3r6qd_ 184820

d3r6qe_ 184821

d3r6qf_ 184822

d3r6qg_ 184823

d3r6qh_ 184824

d3r6va_ 184827

d3r6vb_ 184828

d3r6vc_ 184829

d3r6vd_ 184830

d3r6ve_ 184831

d3r6vf_ 184832

d3r6vg_ 184833

d3r6vh_ 184834

d1j3ua_ 84078

d1j3ub_ 84079

d1jswa_ 19399

d1jswb_ 19400

d1jswc_ 19401

d1jswd_ 19402

d1xwoa_ 162113

d1xwob_ 162114

d1xwoc_ 162115

d1xwod_ 162116

d2pfma_ 167167

d2pfmb_ 167168

d3tv2a_ 185964

d1yfea_ 123061

d2x75a_ 169915

d1gkma_ 70228

d1gkja_ 70227

d1gk2a_ 65229

d1gk2b_ 65230

d1gk2c_ 65231

d1gk2d_ 65232

d1eb4a_ 64894

d1b8fa_ 19434

d1gk3a_ 65233

d1t6ja_ 112259

d1t6jb_ 112260

d1t6pa_ 112263

d1t6pb_ 112264

d1t6pc_ 112265

d1t6pd_ 112266

d1t6pe_ 112267

d1t6pf_ 112268

d1t6pg_ 112269

d1t6ph_ 112270

d1w27a_ 114096

d1w27b_ 114097

d4eeia_ 195579

d4eeib_ 195578

d3c8ta_ 173090

d3nz4a_ 182649

d3nz4b_ 182650

d2yiia_ 170805

d2yiib_ 170806

d2yiic_ 170807

d2yiid_ 170808

d2e9fa_ 163912

d2e9fb_ 163913

d2e9fc_ 163914

d2e9fd_ 163915

d1ubya_ 19435

d1fpsa_ 19436

d1ubva_ 19437

d1ubwa_ 19438

d1ubxa_ 19439

d1rqja_ 97744

d1rqjb_ 97745

d1rqia_ 97742

d1rqib_ 97743

d1rtra_ 97820

d1rtrb_ 97821

d2q80a1 150112

d2q80b_ 150113

d2q80c_ 150114

d2q80d_ 150115

d2q80e_ 150116

d2q80f_ 150117

d1v4ea_ 100296

d1v4eb_ 100297

d1v4ka_ 100302

d1v4ia_ 100299

d1vg3a_ 108601

d1v4ha_ 100298

d1v4ja_ 100300

d1v4jb_ 100301

d1vg2a_ 108600

d1vg6a_ 108604

d1vg7a_ 108605

d1vg4a_ 108602

d1vg4b_ 108603

d3b7la_ 172465

d3n45f_ 181884

d2f94f_ 164288

d3s4ja_ 185269

d2f9kf_ 164292

d3ryea_ 185204

d1zw5a_ 162613

d3n1vf_ 181819

d3n5hf_ 181909

d2f8cf_ 164276

d3n5jf_ 181914

d2f92f_ 164287

d3n46f_ 181885

d2f7mf_ 164274

d4ga3a_ 197013

d3n49f_ 181886

d3n1wf_ 181820

d2f89f_ 164275

d3n6kf_ 181939

d2f8zf_ 164286

d3n3lf_ 181876

d2azla_ 162950

d1wl2a_ 161962

d3p8ra_ 183590

d1ezfa_ 19440

d1ezfb_ 19441

d1ezfc_ 19442

d3vjca_ 195594

d3vjcb_ 195593

d3vjcc_ 195592

d3v66a_ 194203

d3asxa_ 172324

d3q2za_ 184176

d3q30a_ 184177

d1n1ba2 79800

d1n1bb2 79802

d1n20a2 79833

d1n20b2 79835

d1n24a2 79847

d1n24b2 79849

d1n1za2 79829

d1n1zb2 79831

d1n23a2 79843

d1n23b2 79845

d1n22a2 79839

d1n22b2 79841

d1n21a2 79837

d5eaua2 19443

d5easa2 19444

d1hxaa2 83642

d1hxca2 83644

d1hxga2 83646

d5eata2 19445

d1hx9a2 83640

d1di1a_ 19446

d1di1b_ 19447

d1dgpa_ 19448

d1dgpb_ 19449

d1ps1a_ 19450

d1ps1b_ 19451

d1hm7a_ 76719

d1hm7b_ 76720

d1hm4a_ 76715

d1hm4b_ 76716

d3bnya_ 172724

d3bnyb_ 172725

d3bnyc_ 172726

d3bnyd_ 172727

d3bnxa_ 172720

d3bnxb_ 172721

d3bnxc_ 172722

d3bnxd_ 172723

d2oa6a_ 166599

d2oa6b_ 166600

d2oa6c_ 166601

d2oa6d_ 166602

d2e4oa_ 163820

d2e4ob_ 163821

d2e4oc_ 163822

d2e4od_ 163823

d3ckea_ 173281

d3ckeb_ 173282

d3ckec_ 173283

d3cked_ 173284

d1kiya_ 72555

d1kiyb_ 72556

d1jfaa_ 66622

d1jfab_ 66623

d1jfga_ 66636

d1jfgb_ 66637

d1kiza_ 72557

d1kizb_ 72558

d3oacd_ 196411

d3m0gb_ 196463

d3lvsa_ 196800

d4dwba_ 196824

d4e1ea_ 196825

d1kp8a1 84414

d1kp8b1 84417

d1kp8c1 84420

d1kp8d1 84423

d1kp8e1 84426

d1kp8f1 84429

d1kp8g1 84432

d1kp8h1 84435

d1kp8i1 84438

d1kp8j1 84441

d1kp8k1 84444

d1kp8l1 84447

d1kp8m1 84450

d1kp8n1 84453

d1oela1 19452

d1oelb1 19453

d1oelc5 19454

d1oeld1 19455

d1oele1 19456

d1oelf1 19457

d1oelg1 19458

d1mnfa1 91321

d1mnfb1 91324

d1mnfc1 91327

d1mnfd1 91330

d1mnfe1 91333

d1mnff1 91336

d1mnfg1 91339

d1mnfh1 91342

d1mnfi1 91345

d1mnfj1 91348

d1mnfk1 91351

d1mnfl1 91354

d1mnfm1 91357

d1mnfn1 91360

d1pcqa1 94444

d1pcqb1 94447

d1pcqc1 94450

d1pcqd1 94453

d1pcqe1 94456

d1pcqf1 94459

d1pcqg1 94462

d1pcqh1 94465

d1pcqi1 94468

d1pcqj1 94471

d1pcqk1 94474

d1pcql1 94477

d1pcqm1 94480

d1pcqn1 94483

d1aona1 19473

d1aonb1 19474

d1aonc1 19475

d1aond1 19476

d1aone1 19477

d1aonf1 19478

d1aong1 19479

d1aonh1 19480

d1aoni1 19481

d1aonj1 19482

d1aonk1 19483

d1aonl1 19484

d1aonm1 19485

d1aonn1 19486

d1pf9a1 94606

d1pf9b1 94609

d1pf9c1 94612

d1pf9d1 94615

d1pf9e1 94618

d1pf9f1 94621

d1pf9g1 94624

d1pf9h1 94627

d1pf9i1 94630

d1pf9j1 94633

d1pf9k1 94636

d1pf9l1 94639

d1pf9m1 94642

d1pf9n1 94645

d1grla1 19487

d1grlb1 118456

d1grlc1 118459

d1grld1 118462

d1grle1 118465

d1grlf1 118468

d1grlg1 118471

d1kpo11 90976

d1kpo21 90979

d1kpoo1 90982

d1kpop1 90985

d1kpoq1 90988

d1kpor1 90991

d1kpos1 90994

d1kpot1 90997

d1kpou1 91000

d1kpov1 91003

d1kpow1 91006

d1kpox1 91009

d1kpoy1 91012

d1kpoz1 91015

d1j4za1 90838

d1j4zb1 90841

d1j4zc1 90844

d1j4zd1 90847

d1j4ze1 90850

d1j4zf1 90853

d1j4zg1 90856

d1j4zh1 90859

d1j4zi1 90862

d1j4zj1 90865

d1j4zk1 90868

d1j4zl1 90871

d1j4zm1 90874

d1j4zn1 90877

d1sjpa1 112092

d1sjpb1 112095

d1ioka1 66224

d1iokb1 66227

d1iokc1 66230

d1iokd1 66233

d1ioke1 66236

d1iokf1 66239

d1iokg1 66242

d1we3a1 109288

d1we3b1 109291

d1we3c1 109294

d1we3d1 109297

d1we3e1 109300

d1we3f1 109303

d1we3g1 109306

d1we3h1 109309

d1we3i1 109312

d1we3j1 109315

d1we3k1 109318

d1we3l1 109321

d1we3m1 109324

d1we3n1 109327

d1wf4a1 109340

d1wf4b1 109343

d1wf4c1 109346

d1wf4d1 109349

d1wf4e1 109352

d1wf4f1 109355

d1wf4g1 109358

d1wf4h1 109361

d1wf4i1 109364

d1wf4j1 109367

d1wf4k1 109370

d1wf4l1 109373

d1wf4m1 109376

d1wf4n1 109379

d1q3qa1 95704

d1q3qb1 95707

d1q3qc1 95710

d1q3qd1 95713

d1q2va1 95643

d1q2vb1 95646

d1q2vc1 95649

d1q2vd1 95652

d1q3ra1 95716

d1q3rb1 95719

d1q3rc1 95722

d1q3rd1 95725

d1q3sa1 95728

d1q3sb1 95731

d1q3sc1 95734

d1q3sd1 95737

d1q3se1 95740

d1q3sf1 95743

d1q3sg1 95746

d1q3sh1 95749

d1a6da1 19488

d1a6ea1 19490

d1a6db1 19489

d1a6eb1 19491

d1ecma_ 19492

d1ecmb_ 19493

d2d8da1 131329

d2d8db_ 131330

d2d8ea_ 131331

d1ybza1 122901

d2h9da1 136250

d2h9db_ 136251

d2h9dc_ 136252

d2h9dd_ 136253

d2h9ca_ 136248

d2h9cb_ 136249

d3reta_ 184927

d3retb_ 184928

d3rema_ 184906

d3remb_ 184907

d3hgwa_ 177559

d3hgwb_ 177560

d3hgwc_ 177561

d3hgwd_ 177562

d3hgxa_ 177563

d3hgxb_ 177564

d5csma_ 19494

d1csma_ 19495

d1csmb_ 19496

d4csma_ 19498

d4csmb_ 19499

d2csma_ 19497

d3csma_ 19500

d3csmb_ 19501

d2gtvx1 135704

d2fp1a_ 133891

d2fp1b_ 133892

d2fp2a_ 133893

d2fp2b_ 133894

d2f6la1 133049

d2f6lb_ 133050

d2ao2a_ 127065

d2ao2b_ 127066

d2ao2c_ 127067

d1pprm1 19502

d1pprm2 19503

d1pprn1 19504

d1pprn2 19505

d1ppro1 19506

d1ppro2 19507

d1wova1 121124

d1wzda_ 121493

d1wzdb_ 121494

d1iw0a_ 83720

d1iw0b_ 83721

d1iw0c_ 83722

d1iw1a_ 83723

d1iw1b_ 83724

d1iw1c_ 83725

d3i8ra_ 178146

d3i8rb_ 178147

d3i8rc_ 178148

d2z68a_ 154174

d2z68b_ 154175

d3mooa_ 181465

d3moob_ 181466

d1wnwa_ 114765

d1wnwb_ 114766

d1wnwc_ 114767

d1wnxa_ 114768

d1wnxb_ 114769

d1v8xa_ 108432

d1v8xb_ 108433

d1v8xc_ 108434

d1wzga_ 121501

d1wzgb_ 121502

d1wnva_ 114762

d1wnvb_ 114763

d1wnvc_ 114764

d1wzfa_ 121499

d1wzfb_ 121500

d1n45a_ 79981

d1n45b_ 79982

d3czya_ 157159

d3czyb_ 157160

d1ozwa_ 93858

d1ozwb_ 93859

d1ozla_ 93848

d1ozlb_ 93849

d1oyla_ 93732

d1oylb_ 93733

d1ozra_ 93855

d1ozrb_ 93856

d1xjza1 122049

d1xjzb_ 122050

d1xk3a_ 115400

d1xk3b_ 115401

d1ozea_ 93819

d1ozeb_ 93820

d3k4fa_ 179065

d3k4fb_ 179066

d1ni6a_ 85734

d1ni6b_ 85735

d1ni6c_ 85736

d1ni6d_ 85737

d1twna_ 119366

d1twnb_ 119367

d1xk0a1 122051

d1xk0b_ 122052

d1twra_ 119369

d1twrb_ 119370

d1xk2a_ 115398

d1xk2b_ 115399

d1s13a_ 105152

d1s13b_ 105153

d3hoka_ 177735

d3hokb_ 177736

d1s8ca_ 105364

d1s8cb_ 105365

d1s8cc_ 105366

d1s8cd_ 105367

d1xk1a1 122053

d1xk1b_ 122054

d1t5pa_ 106463

d1t5pb_ 106464

d1n3ua_ 79973

d1n3ub_ 79974

d1oyka_ 93730

d1oykb_ 93731

d3tgma_ 185824

d3tgmb_ 185825

d1j02a_ 90732

d1ix4a_ 90715

d1vgia_ 108622

d1ivja_ 76844

d1ulxa_ 107941

d1ix3a_ 90714

d1ubba_ 99160

d1dvga_ 19510

d1dvgb_ 19511

d1j2ca_ 90780

d1dvea_ 19512

d1irma_ 71347

d1irmb_ 71348

d1irmc_ 71349

d1we1a_ 114542

d1we1b_ 114543

d1we1c_ 114544

d1we1d_ 114545

d1wovb_ 121125

d1woxa_ 121128

d1woxb_ 121129

d1wowa_ 121126

d1wowb_ 121127

d1j77a_ 62674

d1p3ua_ 94069

d1p3ta_ 94068

d1p3va_ 94070

d1sk7a_ 105671

d2a2ma1 126039

d1wwma1 121363

d1udda_ 107776

d1uddb_ 107777

d1uddc_ 107778

d1uddd_ 107779

d1rtwa_ 97823

d1rtwb_ 97824

d1rtwc_ 97825

d1rtwd_ 97826

d2a6ba1 126235

d2f2ga_ 132816

d2f2gb_ 132817

d2gm7a1 135364

d1to9a_ 107172

d1to9b_ 107173

d1yaka1 122833

d1yakb1 122834

d1yakc1 122835

d1yakd1 122836

d1yafa1 122814

d1yafb1 122815

d1yafc1 122816

d1yafd1 122817

d1tyha_ 107458

d1tyhb_ 107459

d1tyhd_ 107460

d1tyhe_ 107461

d2q4xa_ 139880

d2q4xb_ 139881

d1wwmb_ 121364

d3hlxa_ 177684

d3hlxd_ 177685

d3hnha_ 177705

d3hmla_ 177696

d3hmlb_ 177697

d1otva_ 93526

d1otvb_ 93527

d1otwa_ 93528

d1otwb_ 93529

d1rcwa_ 97299

d1rcwb_ 97300

d1rcwc_ 97301

d3ibxd_ 178229

d2rd3a_ 168050

d2rd3d_ 168051

d2gm8a_ 135368

d2gm8b_ 135369

d2gm8c_ 135370

d2gm8d_ 135371

d2gm7b_ 135365

d2gm7c_ 135366

d2gm7d_ 135367

d3mvua_ 181610

d4fn6b_ 194219

d4fn6c_ 194220

d3no6a_ 182434

d3no6b_ 182435

d3no6c_ 182436

d3no6d_ 182437

d1z72a_ 124583

d1z72b_ 124584

d1poca_ 19513

d1g4ia_ 60241

d1vl9a_ 113665

d2baxa_ 128250

d2bcha_ 163040

d1unea_ 19583

d1vkqa_ 108688

d4bp2a_ 19584

d1bp2a_ 19585

d2b96a_ 163013

d1mkta_ 19587

d2zp4a_ 171397

d1bpqa_ 19588

d1o3wa_ 86616

d2bppa_ 19589

d2zp3a_ 171396

d1mkua_ 19590

d1gh4a_ 60516

d2zp5a_ 171398

d1mkva_ 19592

d1c74a_ 19593

d1ceha_ 19591

d2bd1a_ 163044

d2bd1b_ 163045

d1fdka_ 19596

d1mksa_ 19594

d1irba_ 19586

d1kvya_ 19595

d1kvxa_ 19597

d1kvwa_ 19598

d3bp2a_ 19599

d1o2ea_ 92406

d2bp2a_ 19600

d1bvma_ 19601

d3eloa_ 175064

d1le6a_ 73862

d1le6b_ 73863

d1le6c_ 73864

d1le7a_ 73865

d1le7b_ 73866

d3u8ia_ 194471

d3u8ib_ 194470

d1n28a_ 91550

d1n28b_ 91551

d3u8da_ 192821

d3u8db_ 192515

d1kvoa_ 19564

d1kvob_ 19565

d1kvoc_ 19566

d1kvod_ 19567

d1kvoe_ 19568

d1kvof_ 19569

d1kqua_ 91023

d3u8ha_ 192820

d3u8hb_ 192514

d1poda_ 19570

d3u8ba_ 192516

d1poea_ 19571

d1poeb_ 19572

d1bbca_ 19573

d1j1aa_ 83961

d1j1ab_ 83962

d1db4a_ 19574

d1aypa_ 19575

d1aypb_ 19576

d1aypc_ 19577

d1aypd_ 19578

d1aype_ 19579

d1aypf_ 19580

d1db5a_ 19581

d1dcya_ 19582

d1n29a_ 91552

d1hn4a_ 65893

d1hn4b_ 65894

d1l8sa_ 77811

d1l8sb_ 77812

d2b00a_ 127620

d1fxfa_ 60102

d1fxfb_ 60103

d1fx9a_ 60100

d1fx9b_ 60101

d1y6oa_ 162160

d1y6ob_ 162161

d2phia_ 19602

d2phib_ 19603

d2b01a_ 127621

d1y6pa_ 162162

d1y6pb_ 162163

d2azza_ 127619

d2b03a_ 127622

d3p2pa_ 19607

d3p2pb_ 19608

d2b04a_ 127623

d4p2pa_ 19604

d5p2pa_ 19605

d5p2pb_ 19606

d1p2pa_ 19609

d1pisa_ 19610

d1pira_ 19611

d1sfwa_ 19612

d1sfva_ 19613

d1sz8a_ 99044

d1mh8a_ 84963

d1mh7a_ 84962

d1s6ba_ 98592

d1xxwa_ 122431

d1mh2a_ 84960

d2rd4a_ 168052

d1s6bb_ 98593

d1xxwb_ 122432

d1mh2b_ 84961

d2rd4b_ 168053

d3jqla_ 178697

d3njua_ 182336

d1yxla_ 124191

d3osha_ 183266

d1ln8a_ 84634

d3jtia_ 178824

d1oxra_ 93714

d1mf4a_ 91261

d3jq5a_ 178683

d3gcia_ 176518

d3q4ya_ 184213

d1td7a_ 106771

d1zm6a1 125335

d1t37a_ 99116

d2q2ja_ 167397

d2q2jb_ 167398

d3cyla_ 173545

d3cylb_ 173546

d1qlla_ 19556

d1qllb_ 19557

d1s8ia_ 98735

d1s8ha_ 98734

d1s8ga_ 98733

d1goda_ 19617

d1vapa_ 19548

d1vapb_ 19549

d1ppaa_ 19550

d1psja_ 19526

d1m8ra_ 78812

d1bk9a_ 19527

d1m8sa_ 78813

d1jiaa_ 19528

d1jiab_ 19529

d1b4wa_ 19536

d1b4wb_ 19537

d1b4wc_ 19538

d1b4wd_ 19539

d1bjja_ 19530

d1bjjb_ 19531

d1bjjc_ 19532

d1bjjd_ 19533

d1bjje_ 19534

d1bjjf_ 19535

d1c1ja_ 70061

d1c1jb_ 70062

d1c1jc_ 70063

d1c1jd_ 70064

d1a2aa_ 19540

d1a2ab_ 19541

d1a2ac_ 19542

d1a2ad_ 19543

d1a2ae_ 19544

d1a2af_ 19545

d1a2ag_ 19546

d1a2ah_ 19547

d1mc2a_ 74622

d1mg6a_ 79091

d1a3da_ 19517

d1psha_ 19518

d1pshb_ 19519

d1pshc_ 19520

d1a3fa_ 19521

d1a3fb_ 19522

d1a3fc_ 19523

d1owsa_ 87491

d1owsb_ 87492

d1u4ja_ 107673

d1u4jb_ 107674

d1g0za_ 83263

d1g0zb_ 83264

d1fe5a_ 19563

d1g2xa_ 83265

d1g2xb_ 83266

d1g2xc_ 83267

d1dpya_ 19562

d1tc8a_ 106758

d1po8a_ 94966

d3i03a_ 177983

d1umvx_ 99630

d3i3ia_ 178049

d3cxia_ 173528

d3cxib_ 173529

d1u73a_ 113075

d1u73b_ 113076

d3hzda_ 177960

d3hzdb_ 177961

d3hzwa_ 177972

d3hzwb_ 177973

d3i3ha_ 178047

d3i3hb_ 178048

d1gp7a_ 76251

d1gp7b_ 76252

d1gp7c_ 76253

d1m8ta_ 91227

d1m8tb_ 91228

d1m8tc_ 91229

d1m8td_ 91230

d1m8te_ 91231

d1m8tf_ 91232

d2nota_ 19554

d2notb_ 19555

d1ae7a_ 19553

d4e4ca_ 192365

d1buna_ 19614

d1pa0a_ 104097

d1pa0b_ 104098

d3mlma_ 181389

d3mlmb_ 181390

d1pc9a_ 104107

d1pc9b_ 104108

d1vipa_ 19558

d1jltb_ 66868

d1aokb_ 19561

d1jlta_ 66867

d1vpia_ 19559

d1aoka_ 19560

d1q5ta_ 95932

d1q5tb_ 95933

d1rgba1 118769

d1rgbb1 118770

d1rgbk1 118771

d1rgbl1 118772

d1oz6a_ 93805

d1ijla_ 66163

d1ijlb_ 66164

d1oqsb_ 93428

d1oqsd_ 93430

d1oqsf_ 93432

d1oqsh_ 93434

d1oqsa_ 93427

d1oqsc_ 93429

d1oqse_ 93431

d1oqsg_ 93433

d1p7oa_ 104076

d1p7ob_ 104077

d1p7oc_ 104078

d1p7od_ 104079

d1p7oe_ 104080

d1p7of_ 104081

d1ozya_ 104047

d1ozyb_ 104048

d1gmza_ 65356

d1gmzb_ 65357

d1tk4a_ 107073

d1tj9a_ 107015

d1skga_ 98901

d1sxka_ 99027

d1sqza_ 98975

d3g8fa_ 176416

d1tg1a_ 106888

d1th6a_ 106907

d1tjka_ 107026

d1sv3a_ 99015

d3fo7a_ 175927

d1tg4a_ 106889

d1fv0a_ 70147

d1fv0b_ 70148

d1tdva_ 106786

d1q7aa_ 96025

d1oxla_ 93702

d1oxlb_ 93703

d1kpma_ 72844

d1kpmb_ 72845

d1jq9a_ 77149

d1jq9b_ 77150

d1q6va_ 95999

d1tgma_ 106900

d1jq8a_ 77147

d1jq8b_ 77148

d4hmba_ 192530

d1fb2a_ 59758

d1fb2b_ 59759

d2gnsa1 135416

d1tp2a_ 107181

d1tp2b_ 107182

d1oyfa_ 87595

d1oyfb_ 87596

d1cl5a_ 19551

d1cl5b_ 19552

d1sv9a_ 99021

d4eixa_ 192378

d3cbia1 156155

d3cbib1 156156

d3cbic1 156157

d3cbid1 156158

d1poaa_ 19514

d1poba_ 19515

d1pobb_ 19516

d1y4la_ 122624

d1y4lb_ 122625

d1clpa_ 19615

d1clpb_ 19616

d1pp2l_ 19525

d1pp2r_ 19524

d2aoza_ 162848

d4dcfa_ 195185

d4dcfb_ 195183

d4dcfc_ 195181

d4dcfd_ 195184

d1xxsa_ 162122

d1xxsb_ 162123

d3t0ra_ 194872

d3t0rb_ 194871

d3t0rc_ 194870

d3t0rd_ 194873

d3qnla_ 191943

d3qnlb_ 191944

d2ok9a_ 166726

d2ok9b_ 166727

d2osna_ 139300

d2osha_ 166838

d3r0ld_ 184756

d2qoga_ 167741

d2qogb_ 167742

d2qogc_ 167743

d2qogd_ 167744

d3fg5a_ 175766

d3h1xa_ 177152

d2h4ca_ 164936

d2h4cb_ 164937

d2h4cc_ 164938

d2h4cd_ 164939

d2h4ce_ 164940

d2h4cf_ 164941

d2h4cg_ 164942

d2h4ch_ 164943

d2g58a_ 134645

d1zwpa_ 125749

d2oyfa_ 139432

d2arma_ 127208

d2pyca_ 139780

d2q1pa_ 139787

d2qvda_ 151387

d2otfa_ 139312

d2pvta_ 167288

d1zyxa_ 125870

d1zr8a_ 125528

d2pb8a_ 139646

d2qu9a_ 151367

d2qhwa_ 150795

d2olia_ 139137

d2dpza_ 131618

d2pwsa_ 139765

d2quea_ 151368

d2pmja_ 139741

d1y38a_ 122580

d1y38b_ 122581

d2zbha_ 154321

d2o1na_ 138874

d2b17a_ 127661

d2fnxa_ 133833

d2ouba_ 139377

d2otha_ 139313

d4hg9b_ 194479

d4hg9c_ 194478

d2wq5a_ 169556

d1zlba_ 125248

d3iq3a_ 178519

d3iq3b_ 178520

d1zl7a_ 125237

d1z76a_ 162394

d1z76b_ 162395

d2h8ia_ 165008

d2h8ib_ 165009

d3jr8a_ 178718

d3jr8b_ 178719

d2oqda_ 166824

d2oqdb_ 166825

d1pwoa_ 161674

d1pwob_ 161675

d1pwoc_ 161676

d1pwod_ 161677

d1yxha_ 162322

d1y75a_ 162166

d1y75b_ 162167

d2azya_ 127618

d3l30a_ 179891

d3fvja_ 176080

d4dbka_ 186746

d4g5ia_ 192529

d3hswa_ 177821

d3o4ma_ 182790

d3qlma_ 184475

d3fvia_ 176076

d3fvib_ 176077

d3fvic_ 176078

d3fvid_ 176079

d3v9ma_ 193998

d3v9mb_ 193997

d2qhea_ 167597

d2qhda_ 167595

d2qhdb_ 167596

d3bjwa_ 172669

d3bjwb_ 172670

d3bjwc_ 172671

d3bjwd_ 172672

d3bjwe_ 172673

d3bjwf_ 172674

d3bjwg_ 172675

d3bjwh_ 172676

d3ux7c_ 195495

d3g8ha_ 176418

d3g8ga_ 176417

d3diha_ 173980

d2i0ua_ 165364

d2i0ue_ 165365

d4h0qa_ 194682

d2ph4a_ 167187

d2ph4b_ 167188

d1lwba_ 84728

d1faza_ 59757

d1kp4a_ 77479

d1it5a_ 76782

d1it4a_ 76781

d3vbzb_ 194895

d3vc0a_ 194894

d2aiba_ 126824

d2aibb_ 126825

d2a8fa_ 126398

d2a8fb_ 126399

d1ljpa_ 73942

d1ljpb_ 73943

d1lria_ 74223

d1bxma_ 19618

d1beoa_ 19619

d1bega_ 19620

d1g8qa_ 19621

d1g8qb_ 19622

d1iv5a_ 76840

d1iv5b_ 76841

d3mw6a_ 181629

d3mw6c_ 181630

d3mw6d_ 181631

d3mw6e_ 181632

d3mw6f_ 181633

d1dvoa_ 19623

d1vq821 144417

d1vqo21 144429

d1vqp21 144431

d1yhq21 144636

d3cc221 156170

d1s722_ 111599

d1vqm21 144425

d1jj21_ 63082

d1vql21 144423

d1vqk21 144421

d1vqn21 144427

d1yij21 144642

d1yi221 144638

d3ccm21 156333

d1vq721 144415

d1vq921 144419

d3cc721 156221

d1vq521 144411

d3cce21 156261

d3ccu21 156429

d1vq421 144409

d1vq621 144413

d3ccv21 156453

d3cpw11 156904

d1yit21 144644

d3cd621 156477

d3ccl21 156309

d1yjw21 144652

d1m903_ 78837

d3cc421 156197

d3ccj21 156285

d2otl21 145729

d3ccq21 156357

d3cma21 156773

d3ccs21 156405

d2otj21 145727

d3ccr21 156381

d1nji3_ 85790

d2qex21 150689

d1yjn21 144650

d1kqs1_ 68812

d1yj921 144646

d1qvg1_ 96389

d1kc83_ 84354

d1n8r3_ 85426

d1q823_ 96126

d1qvf1_ 96359

d3cme21 156809

d1q813_ 96096

d1k733_ 84315

d1k9m3_ 72210

d1kd13_ 72321

d1k8a3_ 72143

d1m1k3_ 74381

d1q863_ 96164

d2qa421 150174

d1q7y3_ 96062

d1ffky_ 19624

d1w6sb_ 114285

d1w6sd_ 114287

d1h4ib_ 60587

d1h4id_ 60589

d1h4jb_ 60591

d1h4jd_ 60593

d1h4jf_ 60595

d1h4jh_ 60597

d2ad7b_ 126573

d2ad7d_ 126575

d2ad6b_ 126569

d2ad6d_ 126571

d2ad8b_ 126577

d2ad8d_ 126579

d1g72b_ 19627

d1g72d_ 19628

d4aahb_ 19625

d4aahd_ 19626

d1lrwb_ 91112

d1lrwd_ 91114

d2d0vb_ 163538

d2d0ve_ 163540

d2d0vj_ 163542

d3v5wg_ 194615

d1tbge_ 19630

d1tbgf_ 19631

d1tbgg_ 19632

d1tbgh_ 19633

d2trcg_ 19634

d1gg2g_ 19636

d1gp2g_ 19635

d1omwg_ 87090

d3pvug_ 184020

d3pvwg_ 184022

d3cikg_ 173249

d3pscg_ 183953

d3krwg_ 179631

d1xhmb_ 122006

d1b9yb_ 19637

d1a0rg_ 19639

d1b9xb_ 19638

d2bcjg1 128290

d3ah8g_ 172178

d1gotg_ 19629

d1hfes_ 19640

d1hfet_ 19641

d1ef1c_ 19642

d1ef1d_ 19643

d1dpjb_ 19645

d1g0vb_ 60190

d1dp5b_ 19646

d1e79i_ 19647

d1h8ei_ 60758

d2jdii_ 161477

d2ck3i_ 161403

d2v7qi_ 152735

d4asui_ 195069

d1pbyc_ 94423

d1jjuc_ 66780

d1jmxg_ 66907

d1jmzg_ 66914

d4ihje_ 194008

d3ryce_ 185203

d3ryie_ 185208

d3ryfe_ 185205

d3ryhe_ 185207

d3ut5e_ 194854

d1sa0e_ 98777

d3e22e1 157987

d1z2be1 124375

d3du7e1 157864

d1sa1e_ 98786

d1rp3b_ 97681

d1rp3d_ 97685

d1rp3f_ 97689

d1rp3h_ 97693

d1sc5b_ 98801

d2f2ac_ 132804

d2g5hc_ 134661

d2dqnc_ 131642

d2df4c1 131448

d2g5ic1 134665

d3ip4c_ 178498

d1wmib1 121046

d1wmid_ 121048

d2fo1d1 145175

d2es4d1 145122

d1h29a_ 76546

d1h29b_ 76547

d1h29c_ 76548

d1h29d_ 76549

d1gwsa_ 76370

d1czja_ 19663

d3caoa_ 19661

d3cara_ 19662

d1w7oa_ 114318

d1up9a_ 113390

d1upda_ 113391

d3cyra_ 19648

d2cy3a_ 19649

d1i77a_ 61878

d1gmba_ 76233

d1gm4a_ 76232

d1aqea_ 19650

d1wada_ 19658

d1qn0a_ 19659

d1qn1a_ 19660

d1gyoa_ 70763

d1gyob_ 70764

d1j0pa_ 90743

d2ewka_ 164205

d2yyxa_ 170938

d2ewia_ 164204

d2ewua_ 164206

d1j0oa_ 90742

d2yywa_ 170937

d1wr5a_ 114835

d2yxca_ 170904

d2z47a_ 171048

d2z47b_ 171049

d2ctha_ 19652

d2cthb_ 19653

d2ffna_ 133390

d2cdva_ 19651

d2cyma_ 19654

d1mdva_ 19655

d1mdvb_ 19656

d1a2ia_ 19657

d2bpna1 128950

d1it1a_ 71406

d1hh5a_ 61041

d1kwja_ 68877

d1l3oa_ 73551

d1ehja_ 19667

d1newa_ 19664

d1f22a_ 19665

d1lm2a_ 74026

d1rwja_ 105114

d1os6a_ 93476

d1ofwa_ 92850

d1ofwb_ 92851

d19hca_ 19668

d19hcb_ 19669

d1ofya_ 92852

d1ofyb_ 92853

d1duwa_ 59138

d3bxua_ 172924

d3bxub_ 172925

d3h4na_ 177177

d3h4nb_ 177178

d3h34a_ 177167

d4hdla_ 194395

d4hb8a_ 194400

d3sj4x_ 195007

d4haja_ 194402

d4hbfa_ 194401

d3sj1x_ 195006

d4hb6a_ 194399

d4hc3a_ 194398

d3sj0x_ 195008

d3selx_ 195042

d3h33a_ 177166

d2wjnc_ 169383

d2i5nc_ 137063

d2wjmc_ 169380

d1dxrc_ 19670

d1vrnc_ 161867

d6prcc_ 19671

d2jblc_ 165986

d1prcc_ 19674

d5prcc_ 19673

d3prcc_ 19672

d2prcc_ 19675

d3g7fc_ 176407

d7prcc_ 19677

d1r2cc_ 96859

d3d38c1 157273

d1eysc_ 19678

d1oaha_ 86736

d1oahb_ 86737

d2rf7a_ 168071

d2rf7b_ 168072

d2rf7c_ 168073

d2rf7d_ 168074

d1gu6a_ 70575

d1gu6c_ 70576

d1gu6e_ 70577

d1gu6g_ 70578

d1qdba_ 19688

d1qdbb_ 19689

d1qdbc_ 19690

d1fs8a_ 19692

d1fs7a_ 19691

d1fs9a_ 19693

d1ft5a_ 19681

d1ft6a_ 19682

d1bvba_ 19683

d1h21a_ 76510

d1h21b_ 76511

d1h21c_ 76512

d1h21d_ 76513

d1y0pa1 122507

d1q9ia1 96273

d2b7ra1 128049

d1kssa1 72928

d1m64a1 78683

d1m64b1 78686

d1e39a1 19694

d1qjda1 19695

d1ksua1 72931

d1ksub1 72934

d2b7sa1 128052

d1jrya1 67199

d1jryb1 67202

d1lj1a1 78023

d1lj1b1 78026

d1jrza1 67205

d1jrzb1 67208

d1jrxa1 67193

d1jrxb1 67196

d1p2ea1 93926

d1p2ha1 93930

d1qo8a1 19696

d1qo8d1 19697

d1m1qa_ 74419

d1m1ra_ 74420

d1m1pa_ 74413

d1m1pb_ 74414

d1m1pc_ 74415

d1m1pd_ 74416

d1m1pe_ 74417

d1m1pf_ 74418

d1d4da1 19702

d1d4ca1 19698

d1d4cb1 19699

d1d4cc1 19700

d1d4cd1 19701

d1d4ea1 19703

d1fgja_ 19679

d1fgjb_ 19680

d1jnia_ 71761

d1ogyb_ 92954

d1ogyd_ 92957

d1ogyf_ 92960

d1ogyh_ 92963

d1ogyj_ 92966

d1ogyl_ 92969

d1ogyn_ 92972

d1ogyp_ 92975

d1sp3a_ 105866

d2j7aa_ 165927

d2j7ab_ 165928

d2j7ad_ 165929

d2j7ae_ 165930

d2j7ag_ 165931

d2j7ah_ 165932

d2j7aj_ 165933

d2j7ak_ 165934

d2j7am_ 165935

d2j7an_ 165936

d2j7ap_ 165937

d2j7aq_ 165938

d3l1ta_ 179858

d3l1tb_ 179859

d3l1tc_ 179860

d3l1td_ 179861

d2rdza_ 151967

d2rdzb_ 151968

d2rdzc_ 151969

d2rdzd_ 151970

d3tora_ 185897

d3torb_ 185898

d3torc_ 185899

d3tord_ 185900

d3ubra_ 195502

d3ubrb_ 195501

d2e80a_ 132092

d2e81a_ 132093

d3bnja_ 172719

d3bnga_ 172717

d3bnha_ 172718

d3bnfa_ 155438

d2b59b1 127880

d1dava_ 59114

d1daqa_ 59113

d2cclb1 145025

d1ohzb_ 93044

d2ccld_ 145026

d2y3nb_ 170562

d2y3nd_ 170564

d2vn6b_ 161586

d2vn5b_ 161584

d2vn5d_ 161585

d3ul4b_ 194258

d2odgc1 139029

d2odci1 139026

d1jeia_ 62918

d1gjja1 83291

d1gjja2 83292

d1h9ea_ 60825

d1h9fa_ 60826

d1jeqa1 64748

d1jjra_ 66772

d1zrja1 125548

d1kcfa1 68434

d1kcfb1 68436

d2do1a1 131595

d1v66a_ 113543

d1y02a1 116278

d1h1js_ 70850

d1a62a1 64708

d1a8va1 64712

d1a8vb1 64714

d2a8va1 64752

d2a8vb1 64754

d2a8vc1 64756

d1xpua1 115790

d1xpub1 115793

d1xpuc1 115796

d1xpud1 115799

d1xpue1 115802

d1xpuf1 115805

d1pvoa1 88316

d1pvoc1 88321

d1pvod1 88324

d1pvoe1 88327

d1pvof1 88330

d1xpra1 115772

d1xprb1 115775

d1xprc1 115778

d1xprd1 115781

d1xpre1 115784

d1xprf1 115787

d1pv4a1 88295

d1pv4c1 88300

d1pv4d1 88303

d1pv4e1 88306

d1pv4f1 88309

d1xpoa1 122212

d1xpob1 122215

d1xpoc1 122218

d1xpod1 122221

d1xpoe1 122224

d1xpof1 122227

d1a63a1 64710

d2hjqa1 147296

d2outa1 149027

d1e7la1 64728

d1e7lb1 64730

d1en7a1 64733

d1en7b1 64735

d1e7da1 64724

d1e7db1 64726

d2qnfa1 150923

d2qnfb1 150925

d2qnca1 150919

d2qncb1 150921

d1wija_ 114674

d2dk4a1 146533

d1ijya_ 62517

d1ijyb_ 62518

d1ijxa_ 62511

d1ijxb_ 62512

d1ijxc_ 62513

d1ijxd_ 62514

d1ijxe_ 62515

d1ijxf_ 62516

d1h99a1 60814

d1h99a2 60815

d1tlva1 119302

d1tlva2 119303

d1ynjk1 123741

d1ynnk1 123746

d1i6ve_ 61858

d3dxje1 157929

d3dxjo1 157932

d1smye_ 105780

d1smyo_ 105790

d1iw7e_ 71476

d1iw7o_ 71484

d1zyre1 125855

d1zyro1 125865

d2cw0e1 130904

d2cw0o1 130914

d2a6he_ 126266

d2a6ho_ 126276

d2be5e_ 128357

d2be5o_ 128367

d2a68e_ 126197

d2a68o_ 126207

d2a69e_ 126217

d2a69o_ 126227

d3eqle_ 175157

d3eqlo_ 175159

d2a6ee_ 126246

d2a6eo_ 126256

d4g7ho_ 194416

d2o5ie_ 148602

d2o5io_ 161489

d1twff_ 112731

d3cqzf1 156931

d1i50f_ 61760

d1k83f_ 68281

d1i3qf_ 61613

d1twcf_ 112717

d1twaf_ 112702

d1twhf_ 112757

d1i6hf_ 61837

d1twgf_ 112744

d2r7zf1 151661

d2r92f1 151748

d2nvyf1 138686

d2e2if1 132000

d2nvtf1 138653

d2e2jf1 132013

d2vumf1 153551

d2r93f1 151757

d2ja7f1 138197

d2ja7r1 138213

d2nvxf1 138673

d2b63f1 127924

d2ja5f1 138165

d2ja8f1 138229

d2yu9f1 140070

d2ja6f1 138181

d2e2hf1 131987

d2nvzf1 138699

d2b8kf1 128083

d1qkla_ 39754

d1i2ta_ 61582

d1ifwa_ 71206

d1jh4a_ 84171

d1jgna_ 84170

d1g9la_ 60399

d1nmra_ 91992

d3ntwa_ 182537

d3ntwc_ 182538

d1gyza_ 70793

d2zjrn1 154567

d3cf5n1 156554

d2zjqn1 154538

d2zjpn1 154506

d3dlln1 157791

d1xbpo1 145880

d2qamq1 150257

d2qaoq1 150310

d2qbeq1 150477

d2qbgq1 150531

d2i2tq1 145456

d2i2vq1 145498

d2qozq1 151133

d2qp1q1 151186

d3df4q1 157700

d3df2q1 157646

d2qbaq1 150370

d2qbcq1 150423

d2qoxq1 151080

d2qovq1 151027

d1vs8q1 144512

d2qbiq1 150585

d2qbkq1 150639

d2awbq1 144921

d2aw4q1 144880

d1vs6q1 144471

d2vhmq1 153080

d2vhnq1 153112

d2z4lq1 154095

d2z4nq1 154149

d2rdoq1 151957

d2gyco1 145272

d2j28q1 145637

d2gyao1 145250

d3bbxq1 155114

d2j03u1 145604

d2j01u1 145577

d3d5bu1 157363

d3d5du1 157393

d2v47u1 152517

d2v49u1 152552

d2hgqt1 145354

d2hgjt1 145322

d1vspo1 145797

d2hgut1 145386

d1vsao1 144526

d1yl311 144690

d2b66u1 144960

d2b9pu1 144982

d2b9nu1 144973

d1g8ea_ 60346

d1g8eb_ 60347

d2avua1 127383

d2avub1 127384

d2avuc1 127385

d2avud1 127386

d4es4b_ 193467

d3bqoa_ 155503

d1h6oa_ 60689

d1h6pa_ 60690

d1h6pb_ 60691

d3bu8a1 155595

d3bu8b1 155596

d3buaa_ 155597

d3buab_ 155598

d3buac_ 155599

d3buad_ 155600

d1k1fa_ 68007

d1k1fb_ 68008

d1k1fc_ 68009

d1k1fd_ 68010

d1k1fe_ 68011

d1k1ff_ 68012

d1k1fg_ 68013

d1k1fh_ 68014

d1k8ke_ 68311

d1u2ve_ 112994

d1tyqe_ 112847

d2p9ie_ 139587

d2p9ke_ 139595

d2p9le_ 139603

d2p9se_ 139627

d2p9ue_ 139635

d3dxke_ 174328

d2p9ne_ 139611

d3dxme_ 174332

d2p9pe_ 139619

d3ukue_ 193252

d3rsee_ 185143

d1u61a_ 107696

d2nuga1 148452

d2nugb1 148454

d2ez6a1 132616

d2ez6b1 132618

d1rc7a1 97289

d1yz9a1 124271

d1yz9b1 124273

d1jfza_ 66655

d1jfzb_ 66656

d1jfzc_ 66657

d1jfzd_ 66658

d1i4sa_ 66026

d1i4sb_ 66027

d1rc5a_ 97283

d1rc5b_ 97284

d1rc5c_ 97285

d1rc5d_ 97286

d2nufa1 148448

d2nufb1 148450

d1yyka1 124218

d1yykb1 124220

d1yyoa1 124244

d1yyob1 124246

d2nuea1 148444

d2nueb1 148446

d1yywa1 124256

d1yywb1 124258

d1yywc1 124260

d1yywd1 124262

d1o0wa1 80751

d1o0wb1 80753

d1ztda1 125639

d1ztdb_ 125640

d1tlha_ 112506

d1tkva_ 107118

d1tkvb_ 107119

d1tl6a1 119298

d1jr5a_ 67113

d1jr5b_ 67114

d1gpja1 65451

d1knca_ 68703

d1kncb_ 68704

d1kncc_ 68705

d1gu9a_ 65540

d1gu9b_ 65541

d1gu9c_ 65542

d1gu9d_ 65543

d1gu9e_ 65544

d1gu9f_ 65545

d1gu9g_ 65546

d1gu9h_ 65547

d1gu9i_ 65548

d1gu9j_ 65549

d1gu9k_ 65550

d1gu9l_ 65551

d1lw1a_ 74290

d1lw1b_ 74291

d1lw1c_ 74292

d1me5a_ 79024

d1me5b_ 79025

d1me5c_ 79026

d2q0ta1 149990

d2q0tb_ 149991

d2q0tc_ 149992

d2af7a1 126658

d2af7b_ 126659

d2af7c_ 126660

d2af7d_ 126661

d2af7e_ 126662

d2af7f_ 126663

d2af7g_ 126664

d2af7h_ 126665

d2af7i_ 126666

d1vkea_ 108650

d1vkeb_ 108651

d1vkec_ 108652

d1vked_ 108653

d1vkee_ 108654

d1vkef_ 108655

d1p8ca_ 94354

d1p8cb_ 94355

d1p8cc_ 94356

d1p8cd_ 94357

d1p8ce_ 94358

d1p8cf_ 94359

d2gmya1 135398

d2gmyb_ 135399

d2gmyc_ 135400

d2gmyd_ 135401

d2gmye_ 135402

d2gmyf_ 135403

d2o4da1 138907

d2ijca1 137465

d2ijcb_ 137466

d2ijcc_ 137467

d2ijcd_ 137468

d2ijce_ 137469

d2ijcf_ 137470

d2ijcg_ 137471

d2ijch_ 137472

d2ijci_ 137473

d2oyoa1 149068

d2oyob_ 149069

d2prra1 149803

d2prrb_ 149804

d2prrc_ 149805

d2prrd_ 149806

d2prre_ 149807

d2prrf_ 149808

d2prrg_ 149809

d2prrh_ 149810

d2prri_ 149811

d2prrj_ 149812

d2prrk_ 149813

d2prrl_ 149814

d2pfxa1 149452

d2pfxb_ 149453

d3c1la_ 172986

d3c1lb_ 172987

d3c1lc_ 172988

d3c1ld_ 172989

d3c1le_ 172990

d3c1lf_ 172991

d3c1lg_ 172992

d3c1lh_ 172993

d3c1li_ 172994

d3c1lj_ 172995

d3c1lk_ 172996

d3c1ll_ 172997

d2ouwa1 149029

d2ouwb_ 149030

d2cwqa1 130931

d2c52b1 145022

d1kbha_ 68382

d1kbhb_ 68383

d1jjsa_ 66773

d2c52a1 129876

d1zoqc_ 125453

d1zoqd_ 125454

d1l8wa_ 73704

d1l8wb_ 73705

d1l8wc_ 73706

d1l8wd_ 73707

d1ov9a_ 93591

d1ov9b_ 93592

d1ni8a_ 80535

d1ni8b_ 80536

d1hnra_ 17896

d1lr1a_ 78154

d1lr1b_ 78155

d1hnsa_ 17895

d2qanm1 150280

d2qalm1 150226

d2qbfm1 150500

d2qbdm1 150446

d2qoym1 151103

d2qp0m1 151156

d3df1m1 157615

d3df3m1 157669

d2avym1 144854

d2aw7m1 144897

d1vs5m1 144447

d1vs7m1 144488

d2qb9m1 150340

d2qbbm1 150393

d2i2pm1 145432

d2i2um1 145474

d2qoum1 150997

d2qowm1 151050

d2qbhm1 150554

d2qbjm1 150608

d2vhom1 153135

d2z4km1 154064

d2z4mm1 154118

d2vhpm1 153157

d2gy9m1 145235

d2gybm1 145257

d2uubm1 139944

d2vqem1 153437

d2vqfm1 153456

d2uuam1 139924

d1j5em_ 71556

d1fjgm_ 16293

d2uucm1 139964

d2uxcm1 140016

d1xmqm_ 115542

d2uu9m1 139904

d1n32m_ 79882

d1xnqm_ 115616

d1xnrm_ 115638

d1hr0m_ 16294

d1hnzm_ 16295

d2j02m1 137878

d2j00m1 137851

d1xmom_ 115512

d1i94m_ 62004

d2uxdm1 152293

d1hnwm_ 16296

d1hnxm_ 16297

d2e5lm1 132037

d1n33m_ 79904

d2hhhm1 136489

d2uxbm1 152274

d1n34m_ 79926

d2v48m1 152531

d1i96m_ 62048

d2v46m1 152495

d2f4vm1 132950

d1n36m_ 79949

d1i97m_ 62071

d1i95m_ 62026

d2hgpp1 136432

d2hgip1 136411

d2hgrp1 136453

d2qnhn1 150937

d2ow8n1 139411

d2r1gi1 151524

d1yl4p1 123609

d2b9om1 128175

d2b64m1 127945

d2b9mm1 128138

d1r2za1 96892

d1l1za1 75911

d1l1ta1 75908

d1l2da1 75920

d1l2ca1 75917

d1r2ya1 96889

d2f5qa1 133004

d1l2ba1 75914

d2f5sa1 133007

d1k82a1 75866

d1k82b1 75869

d1k82c1 75872

d1k82d1 75875

d1tdza1 106787

d1xc8a1 121852

d1pjja1 104164

d1pjia1 104161

d1pm5a1 104190

d1nnja1 80669

d1kfva1 75886

d1kfvb1 75889

d1ee8a1 75823

d1ee8b1 75826

d1k3xa1 77242

d2ea0a1 146754

d1k3wa1 77239

d1q3ba1 104518

d1q3ca1 104521

d1q39a1 104515

d2oq4a1 148978

d2oq4b1 148981

d1tdha1 106772

d2hkja1 136553

d1z5ba1 124464

d1z5bb1 124467

d1z59a1 124455

d1mu5a1 79472

d1z5ca1 124470

d1z5cb1 124473

d1z5aa1 124458

d1z5ab1 124461

d1mx0a1 79618

d1mx0b1 79621

d1mx0c1 79624

d1mx0d1 79627

d1mx0e1 79630

d1mx0f1 79633

d1nexa1 80441

d1nexc1 80445

d1fs1b1 19252

d1fs1d1 19253

d2ovra1 139386

d2ovqa1 139384

d1fqvb1 19262

d1fqvd1 19263

d1fqvf1 19264

d1fqvh1 19265

d1fqvj1 19266

d1fqvl1 19267

d1fqvn1 19268

d1fqvp1 19269

d1fs2b1 19272

d1fs2d1 19273

d2ovpa1 139382

d1p22b1 87717

d1ldkd1 73855

d1nexb1 80443

d1nexd1 80447

d2ovrb1 145735

d2ovqb1 145733

d2ovpb1 145731

d1p22a1 87715

d1fs1a1 19250

d1fs1c1 19251

d2astb1 127273

d2assb1 127270

d1fqva1 19254

d1fqvc1 19255

d1fqve1 19256

d1fqvg1 19257

d1fqvi1 19258

d1fqvk1 19259

d1fqvm1 19260

d1fqvo1 19261

d1fs2a1 19270

d1fs2c1 19271

d1ldke1 73857

d1a6qa1 75801

d2cqna1 130726

d1uzca_ 100222

d2b7ea1 146091

d2dofa1 146551

d2doda1 146549

d2doea1 146550

d1s7za_ 98715

d1q1va_ 104479

d1uj8a1 119683

d2q66a1 150057

d2hhpa1 136503

d3c66a1 155969

d3c66b1 155972

d2o1pa1 138875

d2o1pb1 138878

d1fa0a3 75837

d1fa0b3 75839

d1q79a1 104548

d1f5aa3 75835

d1q78a1 104545

d1px5a1 95277

d1px5b1 95279

d1r89a1 97221

d1r8ca1 97230

d1ueta1 99272

d1r8ba1 97227

d1ueua1 99275

d1r8aa1 97224

d1tfwa1 106862

d1tfwb1 106865

d1tfwc1 106868

d1tfwd1 106871

d2draa1 131666

d2dvia1 131790

d2dr8a1 131660

d2zh6a1 154448

d1ueva1 99278

d2zh3a1 154439

d2zh2a1 154436

d2zh4a1 154442

d2zh5a1 154445

d2dr9a1 131663

d2zh8a1 154454

d2dr7a1 131657

d2drba1 131669

d2zh1a1 154433

d2dr5a1 131654

d2zh9a1 154457

d2zhba1 154463

d2zh7a1 154451

d2zhaa1 154460

d1tfya1 106874

d1tfyb1 106877

d1tfyc1 106880

d1tfyd1 106883

d1sz1a1 106133

d1sz1b1 106136

d2b4va1 127864

d2pbea1 149355

d1m1eb_ 78400

d1t08b_ 112191

d1lujb_ 78226

d1tf5a1 106834

d1m6na1 78697

d1tf2a1 106830

d1m74a1 78720

d1nkta1 85830

d1nktb1 85834

d1nl3a1 85839

d1nl3b1 85843

d1j0ta_ 77051

d1koya_ 77478

d1iyra_ 76973

d1j2na_ 84019

d1j2ma_ 84018

d2rlta1 152157

d1k5oa_ 77269

d2h2ja1 136008

d2h2jb1 136010

d2h2jc1 136012

d1p0ya1 87654

d1p0yb1 87656

d1p0yc1 87658

d2h21a1 135976

d2h21b1 135978

d2h21c1 135980

d1ozva1 87632

d1ozvb1 87634

d1ozvc1 87636

d2h23a1 135982

d2h23b1 135984

d2h23c1 135986

d1mlva1 79283

d1mlvb1 79285

d1mlvc1 79287

d2h2ea1 135998

d2h2eb1 136000

d2h2ec1 136002

d2jola1 148159

d2joka1 148158

d1r6ea_ 104821

d1r9ka_ 104869

d1gzsb_ 76425

d1gzsd_ 76427

d1mi1a1 79137

d1mi1b1 79139

d1t77a1 112290

d1t77b1 112292

d1t77c1 112294

d1t77d1 112296

d1miua1 79153

d1mjea1 79193

d1iyjb1 76948

d1iyjd1 76953

d1miua2 79154

d1mjea2 79194

d1iyjb2 76949

d1iyjd2 76954

d1tf5a2 106835

d1m6na2 78698

d1tf2a2 106831

d1m74a2 78721

d1nkta2 85831

d1nktb2 85835

d1nl3a2 85840

d1nl3b2 85844

d1vfga1 108570

d1vfgb1 108572

d1miwa1 79162

d1miwb1 79164

d1miva1 79158

d1mivb1 79160

d1miya1 79168

d1miyb1 79170

d1ou5a1 87445

d1ou5b1 87447

d1khya_ 77410

d1khyb_ 77411

d1khyc_ 77412

d1khyd_ 77413

d1qvra1 96432

d1qvrb1 96435

d1qvrc1 96438

d1k6ka_ 77274

d1mbxa_ 78927

d1mbxb_ 78928

d1mbua_ 78921

d1mbub_ 78922

d1mg9b_ 79093

d1r6bx1 104818

d1lzwb_ 78313

d1ksfx1 77522

d1mbva_ 78925

d1r6cx_ 118735

d1r6oa_ 118736

d1r6ob_ 118737

d1r6qa_ 118740

d1r6qb_ 118741

d1m98a1 78866

d1m98b1 78868

d1tj1a1 112434

d1tj2a1 112436

d1tiwa1 112430

d2fzna1 134460

d1tj0a1 112432

d1k87a1 77286

d2fzma1 134458

d1ku2a2 83097

d1ku2b2 83099

d1rp3a3 97680

d1rp3c3 97684

d1rp3e3 97688

d1rp3g3 97692

d1sc5a3 98800

d1h3la_ 76639

d1h3lb_ 76640

d1siga_ 19068

d1smyf3 105783

d1smyp3 105793

d2a6hf3 126269

d2a6hp3 126279

d2be5f3 128360

d2be5p3 128370

d2a68f3 126200

d2a68p3 126210

d2a69f3 126220

d2a69p3 126230

d1iw7f3 83088

d1iw7p3 83091

d2a6ef3 126249

d2a6ep3 126259

d1zyrf3 125858

d1zyrp3 125868

d2cw0f3 130907

d2cw0p3 130917

d1or7a2 87333

d1or7b2 87335

d1ng7a_ 85671

d1ng7b_ 85672

d1no1a_ 85913

d1no1b_ 85914

d1no1c_ 85915

d1or7c_ 87336

d1or7f_ 87337

d1ny9a_ 86401

d1ng6a_ 85670

d2g5hb1 134659

d2df4b1 131446

d2g5ib1 134663

d2d6fc1 131307

d2d6fd1 131310

d1zq1c1 125493

d1zq1d1 125496

d1nt2b_ 86150

d2ozbb1 145742

d2ozbe_ 145743

d2idga1 147627

d2idgb_ 147628

d2idgc_ 147629

d1s9ua_ 105390

d2o9xa1 148689

d1n1ca_ 85254

d1n1cb_ 85255

d2xola_ 170261

d2xolb_ 170262

d2yjma_ 196018

d3efpa_ 174916

d3efpb_ 174917

d3u41c_ 193260

d3cw0a_ 173508

d3cw0b_ 173509

d3cw0c_ 173510

d3cw0d_ 173511

d1n81a_ 85388

d1r5qa_ 97103

d1r8ja1 104847

d1r8jb1 104849

d1v2za_ 108300

d1q6ba_ 95968

d1q6bb_ 95969

d1q6aa_ 95966

d1q6ab_ 95967

d1sv1a_ 106039

d1sv1b_ 106040

d1suya_ 106033

d1suyb_ 106034

d1w53a_ 109175

d1ofcx3 92828

d1mp1a_ 91382

d1x3wb1 121672

d1qzea3 96631

d1oqya3 93441

d1tp4a_ 107183

d1pvea_ 104322

d1x3zb_ 121674

d2f4mb_ 132931

d2f4ob_ 132932

d1r6ra_ 97158

d1r6rb_ 97159

d1sfka_ 105488

d1sfkb_ 105489

d1sfkc_ 105490

d1sfkd_ 105491

d1sfke_ 105492

d1sfkf_ 105493

d1sfkg_ 105494

d1sfkh_ 105495

d1o5ha_ 92497

d1o5hb_ 92498

d1s0pa_ 98300

d1s0pb_ 98301

d1tjfa_ 119288

d1tjfb_ 119289

d1uptb_ 99768

d1uptd_ 99770

d1uptf_ 99772

d1upth_ 99774

d1r4ae_ 96990

d1r4af_ 96991

d1r4ag_ 96992

d1r4ah_ 96993

d1vf6a_ 100600

d1vf6b_ 100601

d1y76a1 122683

d1y76c1 122685

d1y74a1 122679

d1y74c1 122681

d1zl8a1 125238

d1y76b1 122684

d1y76d1 122686

d1vf6c_ 100602

d1vf6d_ 100603

d1y74b1 122680

d1y74d1 122682

d1zl8b1 125239

d1rsob_ 97812

d1rsod_ 97814

d1rsoa_ 97811

d1rsoc_ 97813

d1tlqa_ 107134

d1y9ia_ 116586

d1y9ib_ 116587

d1y9ic_ 116588

d1y9id_ 116589

d1rfza_ 97411

d1rfzb_ 97412

d1rfzc_ 97413

d1rfzd_ 97414

d1q5za_ 95954

d1sdia_ 105437

d1qz4a_ 96613

d1izma_ 90725

d1nxha_ 92300

d1nxhb_ 92301

d2icwg_ 137251

d2icwh_ 137252

d1r5id_ 97091

d1r5ih_ 97096

d2ojed1 139107

d2ojeh1 139112

d3kpha_ 179524

d3kphb_ 179525

d1yfsa1 123088

d1yfsb1 123090

d1riqa1 97516

d1yfra1 123084

d1yfrb1 123086

d1yfta1 123092

d1ygba1 123140

d1w2ya_ 109140

d1w2yb_ 109141

d2cica_ 130487

d1ogla_ 92922

d1ogka_ 92918

d1ogkb_ 92919

d1ogkd_ 92920

d1ogke_ 92921

d1vmga_ 113676

d2gtaa1 135630

d2gtab1 135631

d2gtac1 135632

d2gtad1 135633

d2oiea1 139092

d2oieb1 139093

d2oiec1 139094

d2oied1 139095

d2a3qa1 126092

d2a3qb1 126093

d2oiga1 139096

d2oigb1 139097

d2oigc1 139098

d2oigd1 139099

d2p06a1 139452

d2p06b1 139453

d1yvwa1 124122

d2a7wa1 126369

d3c90a_ 156060

d3c90b_ 156061

d3c90c_ 156062

d3c90x_ 156063

d1y6xa1 122676

d1yxba1 124175

d1yxbb_ 124176

d1yxbc_ 124177

d1yxbd_ 124178

d1yxbe_ 124179

d1yxbf_ 124180

d1yxbg_ 124181

d1yxbh_ 124182

d3obca_ 182916

d3obcb_ 182917

d2yaya_ 170711

d2yb0a_ 170716

d2yb0b_ 170717

d2yb0d_ 170718

d2yb0e_ 170719

d2cjea_ 163419

d2yaza_ 170712

d2yazb_ 170713

d2yazd_ 170714

d2yaze_ 170715

d2q73d_ 194680

d2q5za_ 167433

d2q5zb_ 167434

d1us7b_ 99858

d1n93x_ 91707

d1pp1x_ 94969

d1ux5a_ 100144

d1ux4a_ 100142

d1ux4b_ 100143

d1y64b1 122659

d1v9da_ 100535

d1v9db_ 100536

d1v9dc_ 100537

d1v9dd_ 100538

d1un8a1 99661

d1un8b1 99664

d1un9a1 99667

d1un9b1 99670

d3cr3a1 156937

d3cr3b_ 156938

d3pnlb_ 183875

d2btda_ 163160

d1t5ja_ 99125

d3o5ta_ 182815

d3g9da_ 176440

d3g9db_ 176441

d2woec_ 196537

d2wodb_ 196536

d1rf8b_ 97371

d2para_ 167099

d2parb_ 167100

d2paqa_ 139643

d2paqb_ 139644

d2paua_ 167101

d2paub_ 167102

d1ynba1 123718

d1ynbb_ 123719

d1ynbc_ 123720

d1yoya1 123795

d2heka1 136354

d2gz4a1 147196

d2gz4b_ 147197

d2gz4c_ 147198

d2gz4d_ 147199

d2o6ia1 138920

d1xx7a_ 116143

d1xx7b_ 116144

d1xx7c_ 116145

d1xx7d_ 116146

d1xx7e_ 116147

d1xx7f_ 116148

d2pq7a1 149786

d1vj7a1 100801

d1vj7b1 100803

d3dtoa1 157850

d3dtob1 157851

d3dtoc1 157852

d3dtod1 157853

d3djba1 157756

d3djbb_ 157757

d3b57a1 154835

d2pjqa1 149569

d2pjqb_ 149570

d2pjqc_ 149571

d2pjqd_ 149572

d2qgsa1 150782

d2qgsb_ 150783

d2hekb_ 136355

d2cqza_ 163462

d2cqzb_ 163463

d2cqzc_ 163464

d2cqzd_ 163465

d2cqze_ 163466

d2cqzf_ 163467

d1taza_ 106734

d3frga_ 176001

d3d3pa_ 173662

d1f0ja_ 19346

d1f0jb_ 19347

d3gwta_ 177061

d1xm6a_ 115471

d1xm6b_ 115472

d1rora_ 111901

d1rorb_ 111902

d1ro6a_ 111897

d1ro6b_ 111898

d1xlza_ 115459

d1xlzb_ 115460

d1ro9a_ 111899

d1ro9b_ 111900

d1xn0a_ 115571

d1xn0b_ 115572

d1xmua_ 115562

d1xmub_ 115563

d1xosa_ 115712

d1tb5a_ 106735

d1tb5b_ 106736

d1xlxa_ 115455

d1xlxb_ 115456

d1xm4a_ 115465

d1xm4b_ 115466

d3o56a_ 196277

d1xota_ 115713

d1xotb_ 115714

d1xmya_ 115569

d1xmyb_ 115570

d1y2ka_ 122571

d1y2kb_ 122572

d1y2ba_ 122556

d1y2bb_ 122557

d1xora_ 115710

d1xorb_ 115711

d1xoma_ 115704

d1xomb_ 115705

d1tbba_ 106741

d1tbbb_ 106742

d1tb7a_ 106739

d1tb7b_ 106740

d2pw3a_ 167297

d2pw3b_ 167298

d1y2da_ 122560

d1y2db_ 122561

d1y2ca_ 122558

d1y2cb_ 122559

d2qyna_ 151475

d2qynb_ 151476

d1xona_ 115706

d1xonb_ 115707

d1xoqa_ 115708

d1xoqb_ 115709

d1y2ea_ 122562

d1y2eb_ 122563

d3k4sa_ 179079

d1oyna_ 87609

d1oynb_ 87610

d1oync_ 87611

d1oynd_ 87612

d1zkna_ 125201

d1zknb_ 125202

d1zknc_ 125203

d1zknd_ 125204

d1q9ma_ 96288

d1q9mb_ 96289

d1q9mc_ 96290

d1q9md_ 96291

d1ptwa_ 95106

d1ptwb_ 95107

d1ptwc_ 95108

d1ptwd_ 95109

d3iaka_ 178215

d1mkda_ 84982

d1mkdb_ 84983

d1mkdc_ 84984

d1mkdd_ 84985

d1mkde_ 84986

d1mkdf_ 84987

d1mkdg_ 84988

d1mkdh_ 84989

d1mkdi_ 84990

d1mkdj_ 84991

d1mkdk_ 84992

d1mkdl_ 84993

d1so2a_ 98936

d1so2b_ 98937

d1so2c_ 98938

d1so2d_ 98939

d1soja_ 98942

d1sojb_ 98943

d1sojc_ 98944

d1sojd_ 98945

d1soje_ 98946

d1sojf_ 98947

d1sojg_ 98948

d1sojh_ 98949

d1soji_ 98950

d1sojj_ 98951

d1sojk_ 98952

d1sojl_ 98953

d1tbfa_ 106743

d1xoza_ 115719

d2chma1 130472

d1xp0a_ 115720

d2h44a_ 136069

d3tgga_ 185823

d3tgea_ 185822

d2h40a_ 136063

d1t9sa_ 106726

d1t9sb_ 106727

d3bjca1 155329

d1t9ra_ 106725

d3b2ra_ 154768

d3b2rb_ 154769

d1rkpa_ 97620

d2h42a_ 136064

d2h42b_ 136065

d2h42c_ 136066

d1udta_ 99221

d1uhoa_ 107851

d1udua_ 99222

d1udub_ 99223

d3dy8a_ 157937

d3dy8b_ 157938

d3dyna_ 157942

d3dynb_ 157943

d3dysa_ 157946

d3dysb_ 157947

d3jswa_ 178795

d3jswb_ 178796

d2hd1a_ 136338

d2hd1b_ 136339

d4g2ja_ 197157

d4g2jb_ 197159

d4e90a_ 193959

d4e90b_ 193958

d3dyqa_ 157944

d3dyqb_ 157945

d3dyla_ 157940

d3dylb_ 157941

d3k3ha_ 179040

d3k3hb_ 179041

d3n3za_ 181882

d3n3zb_ 181883

d3jsia_ 178785

d3jsib_ 178786

d4gh6a_ 194598

d4gh6b_ 194597

d3k3ea_ 179038

d3k3eb_ 179039

d3hdza_ 177417

d3hc8a_ 177364

d3siea_ 193656

d2qyka_ 167884

d2qykb_ 167885

d3i8vb_ 196548

d2yy2a_ 153829

d2yy2b_ 153830

d3tvxa_ 185967

d3tvxb_ 185968

d3jwqd_ 178877

d3jwrb_ 196525

d1vqra_ 114015

d1vqrb_ 114016

d1vqrc_ 114017

d1vqrd_ 114018

d2ibna1 137195

d2ibnb_ 137196

d2huoa1 136778

d1u6za1 119608

d2floa1 133733

d2flob1 133736

d2floc1 133739

d2flod1 133742

d3ecma_ 174830

d3ecna_ 174831

d3ecnb_ 174832

d3m1ta_ 180721

d1v65a_ 108393

d1rxqa_ 105118

d1rxqb_ 105119

d1rxqc_ 105120

d1rxqd_ 105121

d2p1aa1 149159

d2p1ab_ 149160

d2f22a1 132795

d2f22b_ 132796

d2ou6a1 149020

d2hkva1 147306

d2oqma1 148992

d2oqmb_ 148993

d2oqmc_ 148994

d2oqmd_ 148995

d2nsfa1 148386

d2nsga1 148388

d1ojha_ 103976

d1ojhb_ 103977

d1ojhc_ 103978

d1ojhd_ 103979

d1ojhe_ 103980

d1ojhf_ 103981

d1ojhg_ 103982

d1ojhh_ 103983

d1ojhi_ 103984

d1ojhj_ 103985

d1ojhk_ 103986

d1ojhl_ 103987

d1sj7a1 105603

d1sj7b1 105604

d1sj7c1 105605

d1sj8a1 105606

d1r0da_ 104715

d1r0db_ 104716

d1r0dd_ 104717

d1r0de_ 104718

d1r0df_ 104719

d1r0dg_ 104720

d1r0dh_ 104721

d1r0di_ 104722

d1sj8a2 105607

d1u89a1 119625

d1x4pa1 145853

d2dt6a1 146575

d2dt7b1 146576

d1x4oa1 145852

d1ug0a_ 107822

d1puza_ 104320

d1seda_ 105449

d1sedb_ 105450

d1sedc_ 105451

d1vk5a_ 108635

d1uuja_ 108046

d1uujb_ 108047

d1uujc_ 108048

d1uujd_ 108049

d1txua_ 107439

d2ot3a_ 139311

d1w26a1 109085

d1w26b1 109088

d2vrha1 153506

d1t11a1 106236

d1t11b1 106239

d1m5ya1 78664

d1m5yb1 78667

d1m5yc1 78670

d1m5yd1 78673

d2pv3a1 149882

d2pv3b1 149884

d1tfja_ 161770

d4gh0a_ 196772

d1swxa_ 106075

d4gixa_ 196776

d2euka_ 132391

d1sx6a_ 106078

d2evda_ 132426

d4ghpa_ 196775

d2evla_ 132434

d4gjqa_ 197310

d2evsa_ 132442

d2evse_ 161416

d2euma_ 132400

d1wbea_ 161938

d2bv7a_ 163171

d2evta_ 164201

d1tm9a_ 107150

d1szha_ 106157

d1szhb_ 106158

d1rp4a_ 105027

d1rq1a_ 105047

d3m31a_ 180774

d1q8da_ 104554

d1xn8a_ 115577

d1u84a_ 113114

d1xoua_ 115715

d1xoub_ 115716

d1xlya_ 115457

d1xlyb_ 115458

d1wpba_ 114783

d1wpbb_ 114784

d1wpbc_ 114785

d1wpbd_ 114786

d1wpbe_ 114787

d1wpbf_ 114788

d1wpbg_ 114789

d1wpbh_ 114790

d1wpbi_ 114791

d1wpbj_ 114792

d1wpbk_ 114793

d1wpbl_ 114794

d1wpbm_ 114795

d1wpbn_ 114796

d1wpbo_ 114797

d1wpbp_ 114798

d1td6a_ 112390

d1x9na1 115002

d1t3wa_ 112233

d1t3wb_ 112234

d2haja1 136288

d2idob_ 137285

d2idod_ 137287

d1se7a_ 112074

d2ae9a1 126616

d2axds1 127483

d1du2a_ 15701

d1urua_ 99835

d2elba1 146895

d2d4ca1 131246

d1x04a1 121542

d1x03a1 121541

d1zwwa1 125750

d1zwwb_ 125751

d2c08a1 129578

d2d4cb_ 131247

d2d4cc_ 131248

d2d4cd_ 131249

d2z0va_ 170961

d2z0vb_ 170962

d1i4da_ 61682

d1i4db_ 61683

d1i4ta_ 61729

d1i4tb_ 61730

d1i4la_ 61718

d1i4lb_ 61719

d1i49a_ 61679

d1i49b_ 61680

d2ykta_ 170840

d1y2oa1 122573

d1y2ob_ 122574

d1wdza1 120928

d2efka1 146815

d2efla1 146816

d3soga_ 185471

d1sg7a1 118961

d1x3aa1 121659

d2diia1 131530

d3d8aa_ 157448

d3d8ab_ 157449

d3d8ac_ 157450

d3d8ad_ 157451

d3d8ae_ 157452

d3d8af_ 157453

d3d8ag_ 157454

d3d8ah_ 157455

d2g7oa1 134736

d2g9ea1 134799

d2qklb1 150847

d2a6ta1 126309

d1xl3c1 122104

d1z21a1 124367

d1xkpa1 122086

d1xl3a1 122102

d1xl3b_ 122103

d2au5a1 127322

d1wu9a1 121279

d1wu9b_ 121280

d1yiba1 123278

d2hkqa_ 136557

d1txqb1 119389

d2hl5a_ 136559

d2hl5b_ 136560

d1yiga1 123291

d1yigb_ 123292

d3tq7a_ 185903

d3gjoa_ 176709

d3gjob_ 176710

d3gjoc_ 176711

d3gjod_ 176712

d3tq7b_ 185904

d2choa1 130479

d2chob1 130482

d2vvna1 153634

d2vvnb1 153637

d2chna1 130473

d2chnb1 130476

d2j47a1 137991

d2jiwa1 148101

d2jiwb1 148104

d2vvsa1 153655

d2j4ga1 147876

d2j4gb1 147879

d2cbia1 130179

d2cbib1 130182

d2j62a1 147889

d2j62b1 147892

d2cbja1 130185

d2cbjb1 130188

d2ijqa1 137481

d2cwya1 130960

d2ijqb_ 161463

d2cxda_ 130994

d2cxdb_ 130995

d2gnxa1 147142

d2es9a1 132318

d2fi0a1 133507

d2euca1 132382

d2eucb_ 132383

d2j0oa1 137901

d2j0ob1 137902

d2j0na1 137899

d2j0nb1 137900

d2jaaa1 138238

d2jaab1 138239

d2j9ta1 138153

d3r9va_ 184866

d3r9vb_ 184867

d2c5ra1 129944

d1zaea1 124822

d1zaeb1 124823

d2c5rb_ 129945

d2c5rc_ 129946

d2c5rd_ 129947

d2c5re_ 129948

d2c5rf_ 129949

d2bnka_ 128833

d2bnkb_ 128834

d1ykhb1 123519

d1ykeb1 123514

d1yked1 123516

d1ykha1 123518

d1ykea1 123513

d1ykec1 123515

d1yoza1 123796

d1yozb_ 123797

d2fefa2 133344

d2fefb2 133346

d2fefc2 133348

d2jeka1 138286

d2cxfa1 131004

d2cxla1 131011

d2dwka_ 163729

d2fm9a1 133770

d2fm8c1 133769

d2apla1 127132

d1z67a1 124507

d2gtta1 135682

d2gttb1 135683

d2gttc1 135684

d2gttd1 135685

d2gtte1 135686

d2gttf1 135687

d2gttg1 135688

d2gtth1 135689

d2gtti1 135690

d2gttj1 135691

d2gttk1 135692

d2gttl1 135693

d2gttm1 135694

d2gttn1 135695

d2gtto1 135696

d2gttp1 135697

d2gttq1 135698

d2gttr1 135699

d2gtts1 135700

d2gttt1 135701

d2gttu1 135702

d2gttv1 135703

d2qvja_ 167834

d2qvjb_ 167835

d2qvjc_ 167836

d2qvjd_ 167837

d2qvje_ 167838

d2gica1 135227

d2gicb_ 135228

d2gicc_ 135229

d2gicd_ 135230

d2gice_ 135231

d3hhwk_ 177587

d3hhwl_ 177588

d3hhwm_ 177589

d3hhwn_ 177590

d3hhwo_ 177591

d1z3xa2 124417

d1z3ya2 124419

d1y6ia2 122661

d1zt2b1 125625

d1zt2d1 125626

d2ex3b1 132500

d2ex3d1 132503

d2ex3f1 132506

d2ex3h1 132509

d2ex3j1 132512

d2ex3l1 132515

d2c6ja1 129996

d1zroa1 125551

d1zrob1 125553

d1zrla1 125549

d1zrla2 125550

d3rrca_ 185128

d3rrcb_ 185129

d2f6sa1 133058

d2g03a1 134490

d3zlma_ 197452

d3s6aa_ 185293

d2nw8a1 138714

d2nw8b1 138715

d2nw9a1 138716

d2nw9b1 138717

d1yw0a1 124126

d2nw7a1 138710

d2nw7b1 138711

d2nw7c1 138712

d2nw7d1 138713

d1yw0b_ 124127

d1yw0c_ 124128

d1yw0d_ 124129

d1zeea1 124981

d1zeeb_ 124982

d2d0ta1 131093

d2d0ua1 131095

d2d0ub1 131096

d2d0tb_ 131094

d2nwba_ 166380

d2nwbb_ 166381

d2csba5 130755

d2ixoa1 137779

d2ixna1 137777

d2ixpa1 137781

d2g62a1 134685

d2ixma1 137776

d2hv6a1 136784

d2ixob_ 137780

d2ixnb_ 137778

d2ixpb_ 137782

d2ixpc_ 137783

d2ixpd_ 137784

d2di4a1 131522

d2ce7a1 130312

d2ce7e1 130320

d2ceaa1 130332

d2ceae1 130340

d2bw3a1 129317

d2bw3b1 129319

d2c9wa1 145023

d2izva1 145555

d2nn4a1 148291

d2nn4b_ 148292

d2nn4c_ 148293

d2db7a1 146480

d2db7b_ 146481

d2l7ha_ 166263

d2l7hb_ 166264

d2y0ta_ 170514

d2y0tb_ 170515

d2y20a_ 170517

d2y20b_ 170518

d2y20c_ 170519

d2y20d_ 170520

d2y20e_ 170521

d2y20f_ 170522

d2y0qa_ 170510

d2y0qb_ 170511

d2y0qc_ 170512

d2y0qd_ 170513

d2y21a_ 170523

d2y21b_ 170524

d2y21c_ 170525

d2y21d_ 170526

d2y21e_ 170527

d2y21f_ 170528

d2y21g_ 170529

d2y21h_ 170530

d2y21i_ 170531

d2y21j_ 170532

d2y21k_ 170533

d2y21l_ 170534

d2i5ua1 147519

d2oy9a1 149063

d2oy9b_ 149064

d2pp4a1 149727

d2h7ba1 147237

d2p6va1 149278

d2e9xa1 146739

d2e9xe_ 146746

d2ehob1 146845

d2ehof1 146851

d2ehoj1 146857

d2e9xb1 146740

d2e9xf1 146747

d2q9qa1 150156

d2q9qe1 150162

d2ehoc1 146846

d2ehog1 146852

d2ehok1 146858

d2e9xc1 146742

d2e9xg1 146749

d2q9qd1 150160

d2q9qh1 150166

d2ehod1 146848

d2ehoh1 146854

d2ehol1 146860

d2e9xd1 146744

d2e9xh1 146751

d2q9qb1 150158

d2q9qf1 150164

d2ehoa1 146844

d2ehoe1 146850

d2ehoi1 146856

d2pyqa1 149953

d2pyqd_ 149956

d2peoa1 149441

d2peia1 149405

d2peii_ 149413

d2pema1 149429

d2pemb1 149430

d2pemc1 149431

d2pemd1 149432

d2peme1 149433

d2pemf1 149434

d2peka1 149423

d2pekb1 149424

d2pekc1 149425

d2pekd1 149426

d2peke1 149427

d2pekf1 149428

d2peja1 149417

d2pejb1 149418

d2pejc1 149419

d2pejd1 149420

d2peje1 149421

d2pejf1 149422

d2py8a1 149935

d3q20a_ 184162

d3q20b_ 184163

d2piha1 149511

d2iaza1 147593

d2iazb_ 147594

d2iazc_ 147595

d2iazd_ 147596

d2oeea1 148750

d2oeeb_ 148751

d2oeqa1 148754

d2oeqb_ 148755

d2oeqc_ 148756

d2oeqd_ 148757

d2gqba1 147155

d1uz3a1 145786

d1uz3b1 145787

d2fmme1 145174

d1utua1 145784

d2otaa1 149010

d2otab_ 149011

d2jr2a1 148182

d2jr2b1 148183

d2jpqa1 148167

d2jpqb1 148168

d2juza1 148219

d2juzb1 148220

d2qtia_ 151349

d2juwa1 148217

d2juwb1 148218

d2jrxa1 148187

d2jrxb1 148188

d3brja1 155511

d3brjb_ 155512

d3brjc_ 155513

d3brjd_ 155514

d3c8ga1 156033

d3c8gb1 156034

d3c8gd1 156036

d2pv4a1 149886

d2ou3a1 149018

d2ou3b_ 149019

d2h5na1 147227

d2h5nb_ 161449

d2h5nc_ 161450

d2h5nd_ 161451

d2o8ia1 148676

d2q37a1 150028

d2o70a1 148645

d2o70b_ 148646

d2o70c_ 148647

d2o70d_ 148648

d2o70e_ 148649

d2o70f_ 148650

d2o73a_ 148651

d2o73b_ 148652

d2o73c_ 148653

d2o73d_ 148654

d2o73e_ 148655

d2o73f_ 148656

d2o74a_ 148657

d2o74b_ 148658

d2o74c_ 148659

d2o74d_ 148660

d2o74e_ 148661

d2o74f_ 148662

d2q0zx1 149993

d2p6ra2 149271

d2p6ua2 149275

d2p0ta1 149141

d2i15a1 147485

d2i15b_ 147486

d2i15c_ 147487

d2oufa1 149021

d2bo3a1 146162

d3mlia_ 181385

d3mlib_ 181386

d3mlic_ 181387

d3mlid_ 181388

d2o35a1 148566

d3bzka3 155779

d3bzca3 155754

d2ocea3 148729

d2o8sa1 148680

d2o8sb1 148681

d2ebfx1 146766

d2ebhx1 146769

d2ec5a1 146778

d2ec5b1 146781

d2giya1 135254

d2giyb_ 135255

d2gj7e1 135268

d2gj7f1 135269

d2aw2a1 144863

d2aw2x_ 144864

d1l6za1 77770

d1rjca1 144370

d1xfpa_ 109587

d1zvha1 144772

d1kxqe_ 73171

d1kxqf_ 73172

d1kxqg_ 73173

d1kxqh_ 73174

d1kxvc_ 73190

d1kxvd_ 73191

d1zvya1 144780

d1mvfa_ 85141

d1mvfb_ 85142

d1ri8a1 144369

d1op9a_ 93398

d1jtpa_ 67278

d1jtpb_ 67279

d1jtta_ 67282

d1zv5a1 144771

d1kxtb_ 73179

d1kxtd_ 73182

d1kxtf_ 73185

d1f2xk_ 20511

d1f2xl_ 20512

d1jtoa_ 67274

d1jtob_ 67275

d1mela_ 20505

d1melb_ 20506

d1zmya1 144746

d1bzqk_ 20507

d1bzql_ 20508

d1bzqm_ 20509

d1bzqn_ 20510

d1g6vk_ 20513

d3bn9d1 155426

d3bn9f1 155428

d1sjva_ 105662

d1hcva_ 20514

d1i3ua_ 61636

d1i3va_ 61637

d1i3vb_ 61638

d1qd0a_ 20515

d1za3b1 124789

d1za3h1 124791

d1g9ea_ 76216

d1ieha_ 71199

d1hnfa1 19741

d1qa9a_ 19742

d1qa9c_ 19743

d1cdba_ 19744

d1gyaa_ 19745

d1hnga1 19752

d1hngb1 19753

d1cdca_ 19746

d1cdcb_ 19747

d1a6pa_ 19748

d1a6pb_ 19749

d1a64a_ 19750

d1a64b_ 19751

d1a7ba_ 19754

d1a7bb_ 19755

d1a7bc_ 19756

d1a7bd_ 19757

d1ccza1 19758

d1qa9b_ 19759

d1qa9d_ 19760

d1ci5a1 19761

d1yjdc1 144649

d2nxyb1 138745

d2ny2b1 138770

d2ny4b1 138782

d2ny3b1 138776

d2ny1b1 138764

d2nxzb1 138751

d1cdya1 19719

d2ny0b1 138758

d3cd4a1 19720

d2ny5c1 138788

d1g9mc1 19721

d1rzjc1 98198

d1cdha1 19722

d1cdua1 19723

d1gc1c1 19724

d2ny6b1 138794

d1cdja1 19725

d1rzkc1 98205

d1g9nc1 19726

d2qadb1 150203

d2qadf1 150207

d1cdia1 19727

d2b4cc1 127820

d1wioa1 19728

d1wioa2 19729

d1wiob1 19730

d1wiob2 19731

d1jl4d1 63161

d1wipa1 19732

d1wipa2 19733

d1wipb1 19734

d1wipb2 19735

d1wiqa1 19736

d1wiqa2 19737

d1wiqb1 19738

d1wiqb2 19739

d1cida1 19740

d1akjd_ 19712

d1akje_ 19713

d1cd8a_ 19714

d3qzwg_ 184744

d3qzwh_ 184745

d3qzwi_ 184746

d3qzwj_ 184747

d1nezg_ 85592

d1nezh_ 85593

d2atpa_ 127301

d2atpc_ 127302

d3b9ka_ 172509

d3b9ke_ 172510

d1bqhg_ 19715

d1bqhh_ 19716

d1bqhi_ 19717

d1bqhk_ 19718

d2arjq_ 127200

d2arjr_ 127201

d2atpb1 144837

d2atpd_ 144838

d3b9kb_ 192714

d3b9kf_ 172511

d1dr9a1 19762

d1i8la1 61970

d1i8lb1 61972

d1ncna_ 85553

d1ncnb_ 85554

d1i85a_ 61946

d1i85b_ 61947

d1eaja_ 59401

d1eajb_ 59402

d1f5wa_ 19705

d1f5wb_ 19706

d1kacb_ 19707

d2w9la_ 169120

d2w9lb_ 169121

d2w9lg_ 169126

d2w9lj_ 169129

d2w9lk_ 169130

d2w9lo_ 169134

d2w9lp_ 169135

d2w9lt_ 169139

d2w9lv_ 169140

d2w9lx_ 169141

d2w9ly_ 169142

d2w9lz_ 169143

d1p6ab_ 94162

d1p69b_ 94160

d1rsfa_ 97809

d1jmaa_ 63177

d1l2ga_ 91048

d1l2gb_ 91049

d1l2gc_ 91050

d1l2gd_ 91051

d1nfdf1 20187

d1nfdh1 20189

d1ol0a_ 93296

d1ol0b_ 93297

d1jpth1 67051

d1l7ih1 73656

d1fgvh_ 19853

d1jpsh1 67045

d1t3fb1 112230

d1op3h1 87208

d1op3m1 87214

d1tjgh1 112446

d1ohqa_ 93027

d1ohqb_ 93028

d1tjhh1 112450

d2b1hh1 127668

d1gpoh1 20225

d1gpoi1 20227

d1tjih1 112454

d2f5bh1 88506

d1u8ib1 113134

d1u8hb1 113130

d2f5ah1 88502

d2pr4h1 139748

d1fvcb_ 19863

d1fvcd_ 19865

d1u95b1 113230

d1jv5b_ 67345

d2b0sh1 127646

d1u8kb1 113142

d1u8jb1 113138

d1om3h1 87059

d1om3k1 87061

d1u92b1 113222

d1u91b1 113218

d1u8qb1 113166

d2b1ah1 127662

d1u8mb1 113150

d1fvdb1 19867

d1fvdd1 19869

d1ad0b1 20125

d1ad0d1 20127

d2p8lb1 139526

d1u8nb1 113154

d2h9gb1 136257

d2h9gh1 136259

d1u93b1 113226

d1pg7h1 94674

d1pg7i1 94676

d1u8lb1 113146

d2pw1b1 139758

d1fveb1 19871

d1fved1 19873

d2nyyd1 138831

d1tzhb1 107479

d1tzhh1 107481

d2pw2b1 139760

d2fjfb1 133579

d2fjfd1 133581

d2fjff1 133583

d2fjfh1 133585

d2fjfi1 133587

d2fjfk1 133589

d2fjfn1 133591

d2fjfp1 133593

d2fjfr1 133595

d2fjft1 133597

d2fjfv1 133599

d2fjfx1 133601

d2agjh1 144806

d1n8zb1 80320

d2p8mb1 139528

d2fjgb1 133603

d2fjgh1 133605

d2p8pb1 139530

d1mhph1 84967

d1mhpx_ 84971

d1b2wh1 20307

d2fgwh1 19855

d1ad9b1 20215

d1ad9h1 20213

d1u8ob1 113158

d1bvkb_ 20279

d1bvke_ 20281

d1tzib1 107489

d1it9h1 76783

d1u8pb1 113162

d1t04b1 112184

d1t04d1 112188

d1bvla_ 20282

d1bvlc_ 20284

d1i9rh1 71151

d1i9rk1 71153

d1i9rx1 71159

d2nz9d1 138842

d2nz9f1 138846

d1op5h1 87216

d1op5m1 87222

d1vhpa_ 20504

d1s78d1 98626

d1s78f1 98630

d1uj3b1 107890

d1rjlb1 111823

d1rzfh1 98150

d2fx7h1 134276

d1n0xh1 91528

d1n0xk1 91530

d2nxyd1 138749

d2ny2d1 138774

d1rz7h1 98133

d1rzga1 98154

d1rzgc1 98158

d2ny4d1 138786

d1iqdb1 62646

d2ny3d1 138780

d1vgeh1 20095

d1rhhb1 97475

d1rhhd1 97479

d2ny1d1 138768

d2nxzd1 138755

d2fx9h1 134296

d2fx9i1 134298

d1tzgh1 119396

d1tzgi1 119398

d2ny0d1 138762

d1yymh1 124233

d1yymr1 124240

d2i5yh1 137072

d2i5yr1 137079

d2fx8h1 134280

d2fx8i1 134282

d2fx8j1 134284

d2fx8k1 134286

d2dd8h1 145073

d2ny7h1 138800

d1rz8b1 98139

d1rz8d1 98143

d2g75a1 147086

d2g75c1 147090

d2i60h1 137082

d2i60r1 137089

d2ny5h1 138790

d1g9mh1 20263

d1rzjh1 98201

d1gc1h1 20265

d1yylh1 124223

d1yylr1 124230

d2ny6d1 138798

d1hzhh1 61437

d1hzhk1 61441

d1rzkh1 98208

d1g9nh1 20267

d2qadd1 150205

d2qadh1 150209

d1rzib1 98168

d1rzid1 98172

d1rzif1 98176

d1rzih1 98180

d1rzij1 98184

d1rzil1 98188

d1rzin1 98192

d1rzip1 98196

d7fabh1 19777

d1dn0b1 19831

d1dn0d1 19833

d1qlrb1 19835

d1qlrd1 19837

d2j6eh1 145665

d2j6ei1 145667

d1mcoh1 20564

d8fabb1 19773

d8fabd1 19775

d2fb4h1 19847

d1aqkh1 20193

d2qqnh1 151224

d1w72h1 114299

d1w72i1 114301

d1nl0h1 91945

d3c08h1 155809

d1zlvh1 125262

d1zlvm1 125264

d1zlsh1 125255

d1y0lb1 122488

d1y0ld1 122492

d1y0lf1 122496

d1y0lh1 122498

d1dqlh_ 19829

d1igmh_ 19827

d3cfjb1 156567

d3cfjd1 156571

d3cfjf1 156575

d3cfjh1 156577

d3cfkb1 156583

d3cfkd1 156587

d3cfkf1 156591

d3cfkh1 156595

d3cfki1 156597

d3cfkk1 156601

d3cfkn1 156607

d3cfkp1 156611

d2ghwb2 145199

d2ghwd2 145201

d3dvgb1 157890

d1dfbh1 19811

d3b2uc1 154774

d3b2uf1 154778

d3b2uh1 154780

d3b2uj1 154784

d3b2un1 154788

d3b2uq1 154792

d3b2ut1 154796

d3b2uw1 154800

d1deeb1 20443

d1deed1 20445

d1deef1 20447

d3dvnb1 157896

d3dvnh1 157898

d1hezb1 60985

d1hezd1 60989

d1u6ah1 119564

d2jb5h1 147955

d1y18b1 122523

d1y18d1 122527

d1y18f1 122531

d1y18h1 122533

d3bkyh1 155372

d2oqjb1 148984

d2oqje1 148986

d2oqjh1 148988

d2oqjk1 148990

d1zlwh1 125266

d1zlwm1 125268

d2jb6b1 147957

d2jb6h1 147959

d1zluh1 125258

d1zlum1 125260

d1adqh1 20273

d2ig2h1 19849

d3c09c1 155813

d3c09h1 155817

d3b2vh1 154802

d3c2ah1 155872

d3c2ai1 155874

d1q1jh1 95581

d1q1ji1 95583

d2b4ch1 127822

d1dlfh_ 20383

d3t65b1 185656

d3sy0b1 185583

d2dlfh_ 20385

d3t4yb1 185646

d1qygh1 96589

d1q72h1 96014

d1q9wb1 96322

d1q9wd1 96326

d3t77b1 185669

d1pz5b1 95403

d1q9ob1 96294

d1q9od1 96298

d1riuh1 97518

d1kelh1 20113

d1sbsh1 20367

d1rfdh1 97372

d1flrh1 19905

d1rivh1 97522

d1sm3h1 20369

d1q9kb1 96278

d1kemh1 20115

d1cloh1 20123

d2ck0h1 100853

d1fl5b1 65017

d1fl5h1 65019

d1axth1 20305

d1t66d1 106547

d1t66h1 106549

d2bmkb1 144992

d2bmkh1 144994

d1q9lb1 96282

d1q9ld1 96286

d1m7db1 84863

d1ck0h1 90422

d1nbvh1 19899

d1m7ib1 84867

d1wcbb1 144554

d1wcbh1 144556

d1mamh1 19887

d1wc7b1 144550

d1wc7h1 144552

d1fl6b1 65025

d1fl6h1 65027

d1v7mh1 100450

d1v7mi1 100452

d1cbvh1 19901

d3ck0h1 100865

d1m71b1 84853

d1iaii1 20075

d1mcph1 19859

d2mcph1 19861

d2uudh1 152181

d2uudj1 152182

d4fabh1 19907

d1v7nh1 100460

d1v7ni1 100462

d1v7nj1 100464

d1v7nk1 100466

d1aifb1 20077

d1aifh1 20079

d1jguh1 66688

d1jgvh1 66692

d1lo0h1 74115

d1lo0y1 74121

d1lo2h1 74123

d1lo2y1 74129

d1l7th1 77799

d1ub5a1 99143

d1ub5h1 99147

d1lo3h1 74131

d1lo3y1 74137

d1i9jh1 71144

d1ub6a1 99151

d1ub6h1 99155

d1fl3a1 20486

d1fl3h1 20484

d1i9ih1 71140

d1lo4h1 74139

d1kfah1 77361

d1kfai1 77363

d1vpoh1 113970

d1mqkh_ 85053

d1i8kb_ 71131

d2hffb1 136379

d2hffh1 136381

d1dsfh_ 20247

d1qkzh1 20137

d1hilb1 19787

d1hild1 19789

d1bm3h1 20067

d2fbjh1 19851

d1opgh1 20069

d1h0db1 83427

d1cfvh_ 20161

d1bfvh_ 20163

d1i8ib_ 71129

d2hrph1 20171

d2hrpn1 20173

d1f4xh1 20451

d1ikfh1 20033

d1f4wh1 20453

d1indh1 19839

d2bfvh_ 20165

d2j4wh1 145646

d1bz7b1 20403

d2h1ph1 20191

d1ucbh1 20101

d1ejoh1 20429

d2hfgh1 136383

d1mf2h1 20175

d1mf2n1 20177

d1igch1 20047

d1ifhh1 19791

d1frgh1 19925

d1ar1c_ 20207

d1clyh1 20103

d1clzh1 20105

d1ineh1 19841

d1igfh1 19813

d1igfj1 19815

d1blnb1 20331

d1blnd1 20333

d1fh5h1 20465

d1igtb1 19765

d1igtd1 19767

d1h3ph1 76648

d1himl1 19792

d1himm1 19794

d1otsc1 87417

d1otse1 87421

d2igfh1 19817

d1f4yh1 20455

d1qleh_ 20209

d2j5lc1 145663

d1hinh1 19797

d1knob1 20009

d1knod1 20011

d1knof1 20013

d1ottc1 87427

d1otte1 87431

d1r24b1 20405

d1r24d1 20407

d1qfwi_ 20437

d1otuc1 87437

d1otue1 87441

d1uz8b1 108168

d1uz8h1 108170

d1uz6f1 108152

d1uz6h1 108154

d1uz6p1 108160

d1uz6w1 108164

d2gfbb1 19993

d2gfbd1 19995

d2gfbf1 19997

d2gfbh1 19999

d2gfbj1 20001

d2gfbl1 20003

d2gfbn1 20005

d2gfbp1 20007

d1wejh1 20361

d1cr9h1 20431

d1a3rh1 19781

d1dvfd_ 20107

d12e8h1 20269

d12e8p1 20271

d35c8h1 20297

d1il1a1 62539

d1gafh1 20083

d2rcsh1 20087

d1dzba1 20488

d1dzbb1 20490

d1aj7h1 20085

d25c8h1 20299

d1qblh1 20363

d1qbmh1 20365

d1qoka1 20492

d1k6qh1 72092

d15c8h1 20301

d1hklh1 20089

d1fgnh1 20217

d1cu4h1 20433

d2bdnh1 144985

d2ap2b_ 20179

d2ap2d_ 20181

d1ap2b_ 20183

d1ap2d_ 20185

d1moea2 85031

d1moeb2 85033

d2gsib1 145221

d2gsid1 145223

d2gsif1 145225

d2gsih1 145227

d1bbdh1 19783

d1jnlh1 66956

d1a5fh1 20303

d1jnnh1 66960

d1ahwb1 20219

d1ahwe1 20221

d2hh0h1 136462

d1mjuh1 91302

d1mexh1 91257

d1j05b_ 90736

d1j05h_ 90737

d1rurh1 97879

d1c5ch1 20475

d1mfah1 19889

d1um5h1 113302

d1mj8h1 91290

d1q0xh1 95517

d1p7kb1 94289

d1p7kh1 94291

d1mvub_ 91475

d1f3dh1 20462

d1f3dk1 20463

d1n7ml1 80254

d1um4h1 113298

d1e6oh1 20495

d1a6wh_ 20253

d1xiwd_ 115370

d1xiwh_ 115374

d1ruqh1 97875

d1r3jb1 96930

d1nqba1 20042

d1nqbc1 20044

d2fath1 147015

d1yejh1 20141

d1a3lh1 20241

d1q0yh1 95521

d1jfqh1 66646

d1um6h1 113306

d1yeih1 20143

d1h8na2 60784

d1mjjb1 91296

d1mjjh1 91298

d1yech1 20145

d1mfbh1 19891

d1p2cb1 93917

d1p2ce1 93922

d1kn4h1 72765

d1kn2h1 72760

d1mfch1 19895

d1mfeh1 19893

d1e4wh1 59232

d1mieh1 91281

d1k4ca1 68126

d2fd6h1 145155

d1yegh1 20155

d1yefh1 20153

d1a6uh_ 20255

d1mfdh1 19897

d1c5bh1 20477

d6fabh1 19909

d1d5ih1 20195

d1e4xh1 59236

d1e4xi1 59238

d1yekh1 20147

d1i8mb1 66084

d1i8mh1 66086

d1d6vh1 20197

d1mh5b1 91268

d1mh5h1 91270

d1mlbb1 20051

d1s5hb1 105269

d1yeeh1 20157

d1mj7h1 91286

d1k4da1 68131

d1emth1 20483

d1mreh1 20019

d1i3gh_ 66018

d1kegh1 77345

d1mrdh1 20017

d2cgrh1 19919

d1a0qh1 20287

d1nmbh_ 20063

d1mrfh1 20021

d1r3ih1 96924

d3fctb1 20375

d3fctd1 20377

d1mrch1 20023

d1r3lb1 96940

d1iqwh1 66277

d1h8sa2 60790

d1h8sb2 60792

d1yehh1 20159

d1a6vh_ 20257

d1a6vi_ 20259

d1a6vj_ 20261

d2pcpb1 20371

d2pcpd1 20373

d2mpah1 20357

d1rihh1 97509

d1eapb1 20015

d1ehlh1 20503

d1a6tb1 20249

d1a6td1 20251

d1ngph1 20091

d1cicb1 19965

d1igjb1 19819

d1igjd1 19821

d2jelh1 19979

d1mnuh1 20359

d1ktrh_ 72992

d1plgh1 20081

d1pg7x1 94684

d1pg7z1 94688

d1a14h_ 20061

d1h8oa2 60786

d1h8ob2 60788

d1mpah1 20135

d1forh1 19989

d1igih1 19823

d1ngqh1 20093

d1nj9b1 91899

d1nj9h1 91901

d1nmcb_ 20058

d1nmch_ 20057

d1jhlh_ 19967

d1mlcb1 20053

d1mlcd1 20055

d1mimh1 20129

d3bt2h1 155543

d1bqlh1 19971

d1lmka1 20034

d1lmkc1 20036

d1lmke1 20038

d1lmkg1 20040

d1rmfh1 20029

d1eo8h1 20295

d1qfuh1 20293

d2f19h1 19843

d1pskh1 20139

d1faih1 19845

d1e6jh1 20497

d1axsb1 20201

d1axsh1 20199

d1jnhb1 66941

d1jnhd1 66945

d1jnhf1 66949

d1jnhh1 66953

d1uwgh1 100115

d1uwgy1 100121

d1kb5h1 20229

d1f11b1 20457

d1f11d1 20459

d2iffh1 19973

d1uweh1 100103

d1uwev1 100109

d1uwey1 100113

d1jn6b1 66925

d1r3kb1 96935

d1bbjb1 118420

d1bbjh1 19785

d1egjh1 20501

d1fbih1 20025

d1fbiq1 20027

d1bogb1 20335

d1nmah_ 20065

d1ae6h1 20211

d1yedb1 20151

d1yedh1 20149

d1d5bb1 20205

d1d5bh1 20203

d1hi6b1 20337

d1cfqb1 20339

d1cgsh1 19921

d1cfsb1 20341

d1za6b1 124806

d1za6d1 124809

d1za6f1 124812

d1za6h1 124815

d1cftb1 20345

d1yuhb1 20099

d1yuhh1 20097

d1hh6b1 20343

d1fpth1 20031

d1hh9b1 20347

d1cfnb1 20349

d1igyb1 19769

d1igyd1 19771

d1fskc1 20467

d1fskf1 20469

d1fski1 20471

d1fskl1 20473

d1pkqb1 88150

d1pkqg1 88155

d1afvh1 20131

d1afvk1 20133

d1figh1 19923

d1qfwh_ 20435

d1qnzh_ 20449

d1rvfh_ 19991

d2dtgc1 145097

d1ngzb1 80502

d1ngxb1 85704

d1ngxh1 85706

d1sy6h1 106113

d1ngyb1 80498

d1b4jh1 20309

d1ngwb1 80490

d1ngwh1 80492

d1ct8b1 20409

d1ct8d1 20411

d1nlbh1 80621

d1n4xh_ 85324

d1n4xi_ 85325

d1mhhb1 79121

d1mhhd1 79125

d1jglh1 66679

d1teth1 19903

d1c1eh1 20239

d1n64h1 80114

d1jhkh1 66715

d1bj1h1 20323

d1bj1k1 20325

d1i7zb1 61921

d1i7zd1 61925

d1a4jb1 20233

d1a4jh1 20231

d1ncbh1 19981

d1cz8h1 20327

d1cz8y1 20329

d1ncah1 19983

d1a4kb1 20237

d1a4kh1 20235

d1ncdh1 19985

d1ncch1 19987

d1jp5a2 67002

d1jp5b2 67004

d1fj1b1 20119

d1fj1d1 20121

d1ghfh1 20109

d1dbjh1 19801

d1dbbh1 19799

d1dbah1 19803

d1cl7h1 20419

d1dbmh1 19805

d1iaih1 20073

d2dblh1 19809

d1dbkh1 19807

d1ob1b1 86750

d1ob1e1 86756

d1tqbb1 107213

d1tqbc1 107215

d1tpxb1 107190

d1tpxc1 107192

d1tqcb1 107218

d1tqcc1 107220

d1a2yb_ 19927

d1vfab_ 19929

d1g7jb_ 19933

d1vfbb_ 19931

d1g7ib_ 19935

d1kiqb_ 19939

d1g7mb_ 19937

d1fnsh1 20289

d1a7nh_ 19941

d1g7hb_ 19949

d1a7oh_ 19943

d1a7rh_ 19945

d1kirb_ 19947

d1dvfb_ 19953

d1a7ph_ 19951

d1a7qh_ 19955

d1g7lb_ 19959

d1kipb_ 19957

d1fe8h1 59786

d1fe8i1 59788

d1fe8j1 59790

d1nc2b1 91783

d1nc2d1 91787

d1oakh1 20291

d43c9b_ 20387

d43c9d_ 20389

d43c9f_ 20391

d43c9h_ 20393

d1nc4b1 91791

d1nc4d1 91795

d43cab_ 20395

d43cad_ 20397

d43caf_ 20399

d43cah_ 20401

d1gigh1 19911

d1fdlh1 19961

d1p4bh_ 94098

d1cicd1 19963

d1dl7h_ 20499

d1ibgh1 20049

d1nldh1 20111

d1p4ih_ 94104

d2visb1 19913

d2vitb1 19915

d2virb1 19917

d1t4kb1 112237

d1t4kd1 112241

d1etzb1 20481

d1etzh1 20479

d1ggbh1 19875

d1ggch1 19881

d1ggih1 19877

d1ggij1 19879

d1jrhh1 20223

d1r0ah1 104679

d2hmid1 20275

d1n6qh1 80207

d1t03h1 99056

d1n5yh1 80060

d1hysd1 61422

d1j5oh1 71571

d1ncwh1 85555

d1rukh1 97859

d1ruph1 97871

d1rumh1 97867

d1ruah1 97855

d1kcvh1 72315

d1rulh1 97863

d1f58h1 20351

d1orsb1 87351

d1f8th1 59705

d3cx5j_ 157090

d3cx5u_ 157106

d1ay1h1 20243

d1kcuh1 72311

d1ru9h1 97851

d1kb9j_ 77325

d1f90h1 59709

d2ibzx_ 137229

d1bgxh1 20245

d1ezvx_ 59554

d1cf8h1 20379

d1kcsh1 72307

d1kc5h1 72294

d3cxhj_ 157123

d3cxhu_ 157139

d1c12b1 20381

d1p84j_ 87864

d1nd0b1 85561

d1nd0d1 85565

d1nd0f1 85569

d1nd0h1 85573

d3f58h1 20353

d2f58h1 20355

d1bafh1 19779

d1hq4b1 83623

d1hq4d1 83627

d1kcrh1 72303

d1nsnh1 20071

d1kyoj_ 73262

d1kyou_ 73277

d32c2b1 20439

d1orqb1 87346

d1kenh1 72379

d1kent1 72383

d2a0ld1 125957

d2a0lf1 125959

d1s5ih1 105272

d1uach_ 99132

d3a6ch_ 171774

d2dqdh1 145081

d1j1xh_ 77063

d1j1ph_ 77060

d3a67h_ 171770

d1j1oh_ 77057

d3a6bh_ 171772

d2dqch1 145080

d2dqjh_ 131635

d1dqqb1 20421

d1dqqd1 20423

d1nbyb1 85538

d2dqeh1 145082

d1ndgb1 85578

d1nbzb1 85543

d1ua6h_ 99129

d1osph1 20117

d1dqjb1 20425

d1ic7h_ 62258

d2dqih_ 131633

d1ndmb1 85583

d1ic5h_ 62254

d1dqdh1 20441

d2dqgh1 145085

d1dqmh1 20427

d2dqhh1 145086

d1ic4h_ 62251

d1c08b_ 19975

d2dqfb1 145083

d2dqfe_ 145084

d1nakh1 91753

d1naki1 91755

d3hfmh1 19977

d1ai1h1 19883

d1acyh1 19885

d1oaqh_ 92707

d1lk3h1 73952

d1lk3i1 73954

d1oauh_ 92717

d1oaui_ 92718

d1oauj_ 92719

d1oauk_ 92720

d1ce1h1 20319

d1oarh_ 92709

d1oari_ 92710

d1oarj_ 92711

d1oark_ 92712

d1bfob1 20311

d1bfod1 20313

d1bfof1 20315

d1bfoh1 20317

d1c5db1 20415

d1c5dh1 20413

d2bjmh_ 128624

d1ocwh_ 92779

d1oaxh_ 92725

d1oaxj_ 92727

d1oayh_ 92733

d1oayj_ 92735

d2arjb1 144831

d2arjh1 144833

d1fn4b1 59886

d1fn4d1 59890

d1beyh1 20321

d1f3rb1 20416

d1oazh_ 92743

d1oazj_ 92744

d2r0lh1 151502

d2r0kh1 151500

d2jixd1 148111

d2jixf1 148115

d2jixh1 148117

d1jptl1 67053

d2imna_ 19856

d1l7il1 73658

d1fgvl_ 19852

d1jpsl1 67047

d1t3fa1 112228

d1op3k1 87210

d1op3l1 87212

d1tjgl1 112448

d1tjhl1 112452

d1gpol1 20224

d1gpom1 20226

d1tjil1 112456

d2f5bl1 88508

d1u8ia1 113132

d1ivla_ 20516

d1ivlb_ 20517

d1u8ha1 113128

d2f5al1 88504

d1fvca_ 19862

d1fvcc_ 19864

d1u95a1 113228

d1jv5a_ 67344

d1u8ka1 113140

d1u8ja1 113136

d1om3l1 87063

d1om3m1 87065

d1u92a1 113220

d1u91a1 113216

d1u8qa1 113164

d1u8ma1 113148

d1fvda1 19866

d1fvdc1 19868

d1ad0a1 20124

d1ad0c1 20126

d1u8na1 113152

d1u93a1 113224

d1pg7l1 94678

d1pg7m1 94680

d1u8la1 113144

d1fvea1 19870

d1fvec1 19872

d1tzha1 107477

d1tzhl1 107483

d1n8za1 80318

d1mhpl1 84969

d1mhpy_ 84972

d1b2wl1 20306

d2fgwl1 19854

d1ad9a1 20214

d1ad9l1 20212

d1u8oa1 113156

d1bvka_ 20278

d1bvkd_ 20280

d1tzia1 107487

d1it9l1 76785

d1u8pa1 113160

d1t04a1 112182

d1t04c1 112186

d1bvlb_ 20283

d1bvld_ 20285

d1i9rl1 71155

d1i9rm1 71157

d1i9ry1 71161

d1op5k1 87218

d1op5l1 87220

d1s78c1 98624

d1s78e1 98628

d1s3kh1 105237

d1uj3a1 107888

d1rjla1 111821

d2r9hd1 151784

d2r9hf1 151786

d1bwwa_ 20518

d1bwwb_ 20519

d1wtla_ 20530

d1wtlb_ 20531

d1b0wa_ 20532

d1b0wb_ 20533

d1b0wc_ 20534

d1rz7l1 98135

d1rzgb1 98156

d1rzgd1 98160

d1vgel1 20094

d1qp1a_ 20535

d1qp1b_ 20536

d1qp1c_ 20537

d1brea_ 20538

d1breb_ 20539

d1brec_ 20540

d1bred_ 20541

d1bree_ 20542

d1bref_ 20543

d1reia_ 20520

d1reib_ 20521

d1dqll_ 19828

d1igml_ 19826

d1f6ll_ 76159

d1dfbl1 19810

d1b6da1 20603

d1b6db1 20604

d1deea1 20442

d1deec1 20444

d1deee1 20446

d1heza1 60983

d1hezc1 60987

d1rzia1 98166

d1rzic1 98170

d1rzie1 98174

d1rzig1 98178

d1rzii1 98182

d1rzik1 98186

d1rzim1 98190

d1rzio1 98194

d1ar2a_ 20522

d1eeqa_ 20544

d1eeqb_ 20545

d1eeua_ 20546

d1eeub_ 20547

d1efqa_ 20548

d1qaca_ 20549

d1qacb_ 20550

d1ek3a_ 59434

d1ek3b_ 59435

d3lvea_ 20552

d1lvea_ 20553

d5lvea_ 20551

d4lvea_ 20554

d4lveb_ 20555

d2lvea_ 20556

d1n0xl1 91532

d1n0xm1 91534

d2brrl1 129011

d2brrx1 129013

d1iqda1 62644

d1dn0a1 19830

d1dn0c1 19832

d2ny7l1 138802

d2ghwb1 145198

d1hzhl1 61445

d1hzhm1 61447

d1qlra1 19834

d1qlrc1 19836

d2fx7l1 134278

d1rhha1 97473

d1rhhc1 97477

d2fx9l1 134300

d2fx9m1 134302

d1tzgl1 119400

d1tzgm1 119402

d1yyml1 124235

d1yymq1 124238

d2i5yl1 137074

d2i5yq1 137077

d2fx8l1 134288

d2fx8m1 134290

d2fx8n1 134292

d2fx8o1 134294

d1rz8a1 98137

d1rz8c1 98141

d1y0la1 122486

d1y0lc1 122490

d1y0le1 122494

d1y0ll1 122500

d2i60l1 137084

d2i60q1 137087

d3cfja1 156565

d3cfjc1 156569

d3cfje1 156573

d3cfjl1 156579

d1g9ml1 20262

d3cfka1 156581

d3cfkc1 156585

d3cfke1 156589

d3cfkg1 156593

d3cfkj1 156599

d3cfkl1 156603

d3cfkm1 156605

d3cfko1 156609

d1rzjl1 98203

d3dvga1 157888

d1gc1l1 20264

d1yyll1 124225

d1yylq1 124228

d3dvna1 157894

d3dvnl1 157900

d2agjl1 126727

d1u6al1 119566

d2qscl1 151316

d1y18a1 122521

d1y18c1 122525

d1y18e1 122529

d1y18l1 122535

d1rzkl1 98210

d1g9nl1 20266

d2qr0a1 151234

d2qr0e1 151240

d2qr0g1 151244

d2qr0k1 151250

d2qr0m1 151254

d2qr0q1 151260

d2qr0s1 151264

d2qr0w1 151270

d2b4cl1 127824

d1mjul1 91304

d1ncwl1 85557

d1rukl1 97861

d1rupl1 97873

d1dlfl_ 20382

d1ruml1 97869

d2dlfl_ 20384

d1rurl1 97881

d1mj8l1 91292

d1qygl1 96591

d1q72l1 96016

d1n4xl_ 85326

d1n4xm_ 85327

d1rual1 97857

d2ok0l1 145722

d1jgul1 66690

d1f3dj1 20461

d1f3dl1 20460

d1pz5a1 95401

d1jgvl1 66694

d1wz1l_ 121477

d1ruql1 97877

d1riul1 97520

d1nqba2 20043

d1nqbc2 20045

d1kell1 20112

d1cr9l1 20430

d1yejl1 20140

d1a3ll1 20240

d1rull1 97865

d1yeil1 20142

d1mjja1 91294

d1mjjl1 91300

d1yecl1 20144

d1rfdl1 97374

d1dsfl_ 20246

d1kn4l1 72767

d1kn2l1 72762

d1miel1 91283

d1flrl1 19904

d1qkzl1 20136

d1rivl1 97524

d1f8tl1 59707

d1yegl1 20154

d1yefl1 20152

d1lo0l1 74117

d1lo0x1 74119

d1lo2l1 74125

d1lo2x1 74127

d1tetl1 19902

d1cfvl_ 20160

d1l7tl1 77801

d1yekl1 20146

d1bfvl_ 20162

d1ub5b1 99145

d1ub5l1 99149

d1keml1 20114

d1mh5a1 91266

d1mh5l1 91272

d1fl5a1 65015

d1fl5l1 65021

d1yeel1 20156

d1mj7l1 91288

d1c1el1 20238

d1mrel1 20018

d1kegl1 77347

d1mrdl1 20016

d1axtl1 20304

d2cgrl1 19918

d1t66c1 106545

d1t66l1 106551

d1mrfl1 20020

d1ru9l1 97853

d1lo3l1 74133

d1lo3x1 74135

d1m7da1 84861

d1mrcl1 20022

d1i9jl1 71146

d1f90l1 59711

d2bfvl_ 20164

d1yehl1 20158

d1a4ja1 20232

d1a4jl1 20230

d2pcpa1 20370

d2pcpc1 20372

d1ub6b1 99153

d1ub6l1 99157

d2h1pl1 20190

d2mpal1 20356

d1ehll1 20502

d1ucbl1 20100

d1igja1 19818

d1igjc1 19820

d2jell1 19978

d1mnul1 20358

d1ktrl_ 72993

d1plgl1 20080

d1mpal1 20134

d1igil1 19822

d1nbvl1 19898

d1a4ka1 20236

d1a4kl1 20234

d1m7ia1 84865

d1cu4l1 20432

d1fl3b1 20487

d1fl3l1 20485

d1i9il1 71142

d1nd0a1 85559

d1nd0c1 85563

d1nd0e1 85567

d1nd0g1 85571

d2ojzl1 145718

d2ojzm1 145720

d1lmka2 20035

d1lmkc2 20037

d1lmke2 20039

d1lmkg2 20041

d1rmfl1 20028

d1clyl1 20102

d1clzl1 20104

d1qful1 20292

d1igfl1 19812

d1igfm1 19814

d1jp5a1 67001

d1jp5b1 67003

d1blna1 20330

d1blnc1 20332

d1lo4l1 74141

d1fl6a1 65023

d1fl6l1 65029

d1nldl1 20110

d1cbvl1 19900

d1dbjl1 19800

d1dbbl1 19798

d1dbal1 19802

d1cl7l1 20418

d1dbml1 19804

d1m71a1 84851

d1kfal1 77365

d1kfam1 77367

d2igfl1 19816

d1nakl1 91757

d1nakm1 91759

d1ae6l1 20210

d1yeda1 20150

d1yedl1 20148

d2dbll1 19808

d1cgsl1 19920

d1dbkl1 19806

d1fptl1 20030

d4fabl1 19906

d1maka_ 19825

d1maja_ 19824

d1uz8a1 108166

d1uz8l1 108172

d1s3kl1 105239

d1uz6e1 108150

d1uz6l1 108156

d1uz6m1 108158

d1uz6v1 108162

d1vpol1 113972

d1s5il1 105274

d3t65a1 185654

d3sy0a1 185581

d1nlbl1 80623

d3t4ya1 185644

d1mvua_ 91474

d1q9wa1 96320

d1q9wc1 96324

d2imma_ 19857

d3t77a1 185667

d1q9oa1 96292

d1q9oc1 96296

d1a3rl1 19780

d1sbsl1 20366

d1hila1 19786

d1hilc1 19788

d1mhha1 79119

d1mhhc1 79123

d1q9ka1 96276

d1il1b1 62541

d43c9a_ 20386

d43c9c_ 20388

d43c9e_ 20390

d43c9g_ 20392

d1n64l1 80116

d43caa_ 20394

d43cac_ 20396

d43cae_ 20398

d43cag_ 20400

d1q9la1 96280

d1q9lc1 96284

d2ap2a_ 20178

d2ap2c_ 20180

d1ap2a_ 20182

d1ap2c_ 20184

d1ifhl1 19790

d1frgl1 19924

d1h3pl1 76650

d1bbdl1 19782

d1himh1 19793

d1himj1 19795

d1a5fl1 20302

d1mcpl1 19858

d2mcpl1 19860

d1hinl1 19796

d1pkqa1 88148

d1pkqf1 88153

d2dtgb1 145095

d1t4ka1 112235

d1t4kc1 112239

d1j05a_ 90735

d1j05l_ 90738

d1f58l1 20350

d1h0da1 83425

d2hrpl1 20170

d2hrpm1 20172

d1i7za1 61919

d1i7zc1 61923

d1iqwl1 66279

d1ejol1 20428

d1mf2l1 20174

d1mf2m1 20176

d2gcya1 134994

d2nyyc1 138829

d1moea1 85030

d1moeb1 85032

d2aabl1 144795

d3f58l1 20352

d1ggbl1 19874

d1ibgl1 20048

d2f58l1 20354

d1ggcl1 19880

d1ggil1 19876

d1ggim1 19878

d2gsia1 135579

d2gsic1 135581

d2gsie1 135583

d2gsig1 135585

d1f11a1 20456

d1f11c1 20458

d1ai1l1 19882

d1nsnl1 20070

d1egjl1 20500

d1acyl1 19884

d32c2a1 20438

d1afvl1 20130

d1afvm1 20132

d2nz9c1 138840

d2nz9e1 138844

d1qfwl_ 20434

d1qnzl_ 20448

d2r69l1 151602

d1orsa1 87349

d35c8l1 20296

d25c8l1 20298

d1cf8l1 20378

d15c8l1 20300

d1hq4a1 83621

d1hq4c1 83625

d1iaim1 20074

d1ob1a1 86748

d1ob1d1 86754

d1orqa1 87344

d1figl1 19922

d1aifa1 20076

d1aifl1 20078

d1kenl1 72381

d1kenu1 72385

d2a0lc1 125956

d2a0le1 125958

d1yqvl1 123900

d1ay1l1 20242

d1dqdl1 20440

d2bmka1 128798

d2bmkl1 128800

d2b2xl1 127750

d2b2xm1 127752

d1qoka2 20493

d2z92b1 154226

d1bgxl1 20244

d2j4wl1 138007

d1forl1 19988

d1wcba1 120882

d1wcbl1 120884

d1miml1 20128

d1bqll1 19970

d2z91b1 154220

d2z91d1 154223

d1eo8l1 20294

d1wc7a1 120878

d1wc7l1 120880

d1pskl1 20138

d2oslb1 139296

d2osll1 139298

d1bafl1 19778

d1v7ml1 100454

d1v7mm1 100456

d2iffl1 19972

d2z93d1 154230

d1otsd1 87419

d1otsf1 87423

d2j5lb1 138045

d2fedd1 133333

d2fedf1 133335

d2ht4d1 136738

d2ht4f1 136740

d1v7nl1 100468

d1v7nm1 100470

d1v7nn1 100472

d1v7no1 100474

d2feel1 133339

d2feeo1 133341

d2exwd1 132568

d2exwf1 132570

d2htkd1 136745

d2htkf1 136747

d2htld1 136749

d2htlf1 136751

d2h2pd1 136018

d2h2pf1 136020

d2ht3d1 136734

d2ht3f1 136736

d1ottd1 87429

d1ottf1 87433

d2ht2d1 136730

d2ht2f1 136732

d2hlfd1 136566

d2hlff1 136568

d2h2sd1 136024

d2h2sf1 136026

d2ez0d1 132606

d2ez0f1 132608

d1z3gl1 144733

d1z3gm1 144735

d2fecl1 133329

d2feco1 133331

d1otud1 87439

d1otuf1 87443

d2exyd1 132574

d2exyf1 132576

d1rvfl_ 19990

d1sy6l1 106115

d1clol1 20122

d1a6ta1 20248

d1a6tc1 20250

d1e6ol1 20494

d2fatl1 147017

d2fd6l1 145157

d2fbjl1 19850

d3bt2l1 155545

d1e6jl1 20496

d1mqkl_ 85054

d1mexl1 91259

d1a2ya_ 19926

d1c5cl1 20474

d1um5l1 113304

d1uacl_ 99133

d1vfaa_ 19928

d1p7ka1 94287

d1p7kl1 94293

d1g7ja_ 19932

d3a6cl_ 171775

d1vfba_ 19930

d2dqdl_ 131621

d1i8ka_ 71130

d1j1xl_ 77064

d1j1pl_ 77061

d3a67l_ 171771

d1j1ol_ 77058

d3a6bl_ 171773

d2dqcl_ 131619

d2dqjl_ 131636

d1kcvl1 72317

d1g7ia_ 19934

d1dqqa1 20420

d1dqqc1 20422

d1nbya1 85536

d1n7mh1 80252

d1kiqa_ 19938

d2adfl1 126586

d1um4l1 113300

d1wejl1 20360

d2dqel_ 131623

d1g7ma_ 19936

d1fnsl1 20288

d1a7nl_ 19940

d1xiwc_ 115369

d1xiwg_ 115373

d1ndga1 85576

d1r3ja1 96928

d1g7ha_ 19948

d1a7ol_ 19942

d1a7rl_ 19944

d1nbza1 85541

d1kira_ 19946

d1jfql1 66648

d1um6l1 113308

d1dvfa_ 19952

d1dvfc_ 20106

d1a7pl_ 19950

d1a7ql_ 19954

d1h8na1 60783

d1ua6l_ 99130

d1p2ca1 93915

d1p2cd1 93920

d1g7la_ 19958

d1e4wl1 59234

d1kipa_ 19956

d12e8l1 20268

d12e8m1 20270

d1k4cb1 68128

d1ospl1 20116

d1dqja1 20424

d1ic7l_ 62259

d1bm3l1 20066

d2dqil1 145087

d1c5bl1 20476

d6fabl1 19908

d1jgll1 66681

d1opgl1 20068

d3cx5k_ 157091

d3cx5v_ 157107

d1fe8l1 59792

d1fe8m1 59794

d1fe8n1 59796

d1d5il1 20194

d1e4xl1 59240

d1e4xm1 59242

d1i8ma1 66082

d1i8ml1 66088

d1d6vl1 20196

d1gafl1 20082

d2rcsl1 20086

d1ndma1 85581

d1dzba2 20489

d1dzbb2 20491

d1aj7l1 20084

d1i8ia_ 71128

d1mlba1 20050

d1s5ha1 105267

d1ic5l_ 62255

d1oakl1 20290

d1kcul1 72313

d1k4db1 68133

d1emtl1 20482

d1ct8a1 20408

d1ct8c1 20410

d1qbll1 20362

d1i3gl_ 66019

d2dqgl_ 131629

d1dqml1 20426

d1a0ql1 20286

d1nmbl_ 20062

d2dqhl_ 131631

d1ikfl1 20032

d1r3il1 96926

d3fcta1 20374

d3fctc1 20376

d1jhkl1 66717

d1bj1j1 20324

d1bj1l1 20322

d1kb9k_ 77326

d1qbml1 20364

d1r3la1 96938

d1ic4l_ 62252

d1h8sa1 60789

d1h8sb1 60791

d2ibzy_ 137230

d1ezvy_ 59555

d1fdll1 19960

d1bz7a1 20402

d1ncbl1 19980

d1rihl1 97511

d1cz8l1 20326

d1cz8x1 20328

d1c08a_ 19974

d1eapa1 20014

d1cica1 19964

d1cicc1 19962

d3cmol1 156837

d3cmox1 156839

d1k6ql1 72094

d1hkll1 20088

d1a14l_ 20060

d1kcsl1 72309

d1h8oa1 60785

d1h8ob1 60787

d1ncal1 19982

d1fgnl1 20216

d1kc5l1 72296

d1nmcc_ 20059

d1nmcl_ 20056

d3cxhk_ 157124

d3cxhv_ 157140

d1c12a1 20380

d2j88l1 138137

d1jhll_ 19966

d1mlca1 20052

d1mlcc1 20054

d1p84k_ 87865

d1maml1 19886

d1igcl1 20046

d2dqfa_ 131625

d2dqfd_ 131627

d1ar1d_ 20206

d1ncdl1 19984

d1nccl1 19986

d2f19l1 19842

d1fail1 19844

d1fj1a1 20118

d1fj1c1 20120

d1fh5l1 20464

d1axsa1 20200

d1axsl1 20198

d1igta1 19764

d1igtc1 19766

d1kcrl1 72305

d1uwgl1 100117

d1uwgx1 100119

d1kb5l1 20228

d1ghfl1 20108

d1uwel1 100105

d1uweu1 100107

d1uwex1 100111

d1r3ka1 96933

d1bbja1 118418

d1bbjl1 19784

d1jnll1 66958

d1fbil1 20024

d1fbip1 20026

d1jrhl1 20222

d1boga1 20334

d1nmal_ 20064

d1iail1 20072

d1kyok_ 73263

d1kyov_ 73278

d1d5ba1 20204

d1d5bl1 20202

d1hi6a1 20336

d1r0al1 104681

d1cfqa1 20338

d1jnnl1 66962

d1cfsa1 20340

d1cfta1 20344

d1qlel_ 20208

d1hh6a1 20342

d1hh9a1 20346

d1ahwa1 20218

d1ahwd1 20220

d1knoa1 20008

d1knoc1 20010

d1knoe1 20012

d1cfna1 20348

d1igya1 19768

d1igyc1 19770

d1fskb1 20466

d1fske1 20468

d1fskh1 20470

d1fskk1 20472

d2hmic1 20274

d1n6ql1 80209

d1t03l1 99058

d1n5yl1 80062

d3hfml1 19976

d1r24a1 20404

d1r24c1 20406

d1hysc1 61420

d1qfwm_ 20436

d1j5ol1 71573

d2dtgd1 145099

d1ngza1 80500

d1ngxa1 85702

d1ngxl1 85708

d1ngya1 80496

d2hh0l1 136464

d2gfba1 19992

d2gfbc1 19994

d2gfbe1 19996

d2gfbg1 19998

d2gfbi1 20000

d2gfbk1 20002

d2gfbm1 20004

d2gfbo1 20006

d1b4jl1 20308

d1ngwa1 80488

d1ngwl1 80494

d1lk3l1 73956

d1lk3m1 73958

d1ce1l1 20318

d1bfoa1 20310

d1bfoc1 20312

d1bfoe1 20314

d1bfog1 20316

d1c5da1 20414

d1c5dl1 20412

d2arja1 144829

d2arjl1 144835

d1za3a1 124787

d1za3l1 124793

d1fn4a1 59884

d1fn4c1 59888

d1beyl1 20320

d1f3rb2 20417

d2v7na1 152724

d2v7nc1 152726

d2v7ne1 152728

d2v7ng1 152730

d2hwzl1 147429

d1nfde1 20186

d1nfdg1 20188

d1pewa_ 104135

d1pewb_ 104136

d1cd0a_ 20559

d1cd0b_ 20560

d1pw3a_ 104335

d1pw3b_ 104336

d2cd0a_ 20561

d2cd0b_ 20562

d2rhea_ 20523

d1rzfl1 98152

d2fb4l1 19846

d1nl0l1 91947

d1bjma1 20524

d1bjmb1 20525

d3bjla1 20526

d3bjlb1 20527

d4bjla1 20528

d4bjlb1 20529

d1q1jl1 95585

d1q1jm1 95587

d2ig2l1 19848

d2j6el1 145669

d2j6em1 145671

d1aqkl1 20192

d3dgga1 157714

d3dggc1 157717

d7fabl1 19776

d1lgva1 84608

d1lgvb1 84610

d1jvka1 71897

d1jvkb1 71899

d1lhza1 84612

d1lhzb1 84614

d1dcla1 20565

d1dclb1 20566

d2mcg11 20567

d2mcg21 20568

d3mcg11 20569

d3mcg21 20570

d1mcda1 20571

d1mcdb1 20572

d1mcka1 20573

d1mckb1 20574

d1mcba1 20575

d1mcbb1 20576

d1mcqa1 20577

d1mcqb1 20578

d1mcfa1 20579

d1mcfb1 20580

d1mcia1 20581

d1mcib1 20582

d1mcca1 20583

d1mccb1 20584

d1mcsa1 20585

d1mcsb1 20586

d1mcla1 20589

d1mclb1 20590

d1mcea1 20587

d1mceb1 20588

d1mcna1 20591

d1mcnb1 20592

d1mcra1 20593

d1mcrb1 20594

d1mcja1 20595

d1mcjb1 20596

d1a8jh1 20598

d1a8jl1 20597

d1mcha1 20599

d1mchb1 20600

d1mcwm1 20601

d1mcww1 20602

d1mcol1 20563

d8faba1 19772

d8fabc1 19774

d1w72l1 114303

d1w72m1 114305

d2dd8l1 145075

d1lila1 20557

d1lilb1 20558

d1adql1 20272

d1zvoa1 125710

d1zvob1 125712

d1mfal1 19888

d1q0xl1 95519

d1a6wl_ 20252

d1q0yl1 95523

d1mfbl1 19890

d1mfcl1 19894

d1mfel1 19892

d1sm3l1 20368

d1a6ul_ 20254

d1mfdl1 19896

d1nc2a1 91781

d1nc2c1 91785

d1f4xl1 20450

d1nc4a1 91789

d1nc4c1 91793

d1f4wl1 20452

d1indl1 19838

d1gigl1 19910

d1a6vl_ 20256

d1a6vm_ 20258

d1a6vn_ 20260

d1p4bl_ 94099

d1ngpl1 20090

d1pg7w1 94682

d1pg7y1 94686

d1ngql1 20092

d1etza1 20480

d1etzl1 20478

d1dl7l_ 20498

d1nj9a1 91897

d1nj9l1 91903

d1inel1 19840

d1p4il_ 94105

d1jnha1 66939

d1jnhc1 66943

d1jnhe1 66947

d1jnhg1 66951

d1jn6a1 66923

d2visa1 19912

d2vita1 19914

d2vira1 19916

d1f4yl1 20454

d1yuha1 20098

d1yuhl1 20096

d3c6la1 155977

d3c6le1 155983

d1oaql_ 92708

d1oaul_ 92721

d1oaum_ 92722

d1oaun_ 92723

d1oauo_ 92724

d1oarl_ 92713

d1oarm_ 92714

d1oarn_ 92715

d1oaro_ 92716

d2bjml_ 128625

d1ocwl_ 92780

d1oaxl_ 92729

d1oaxm_ 92730

d1oaxn_ 92731

d1oaxo_ 92732

d1oayl_ 92737

d1oaym_ 92738

d1oayn_ 92739

d1oayo_ 92740

d1oazl_ 92745

d1oazn_ 92746

d1i8lc_ 61974

d1i8ld_ 61975

d1i85c_ 61948

d1i85d_ 61949

d1ah1a_ 20652

d1dqta_ 20653

d1dqtb_ 20654

d1dqtc_ 20655

d1dqtd_ 20656

d1nbqa1 85532

d1nbqb1 85534

d1f97a1 19763

d2crya1 146423

d1neua_ 19704

d1pkoa_ 88147

d3cspa_ 173438

d1py9a_ 95329

d1pkqe_ 88152

d1pkqj_ 88157

d2g5ra_ 134674

d1nkoa_ 85829

d1o7sa_ 86644

d2hrla_ 136689

d2df3a_ 131444

d1o7va_ 86655

d1qfoa_ 19708

d1qfob_ 19709

d1qfoc_ 19710

d1od9a_ 86839

d1qfpa_ 19711

d1urla_ 113412

d1od7a_ 86837

d1odaa_ 92781

d1hkfa_ 83553

d1sq2n_ 105881

d1t6vn_ 106598

d1t6vo_ 106599

d2i26n1 145417

d1vera_ 108550

d1vesa_ 108551

d1vesb_ 108552

d1xeda_ 115229

d1xedb_ 115230

d1xedc_ 115231

d1xedd_ 115232

d1xede_ 115233

d1xedf_ 115234

d3bp6a_ 172739

d3bp5a_ 155462

d1npua_ 92038

d3sbwb_ 196327

d3bikb_ 155309

d3bikc_ 155310

d1ogad1 86990

d1kgcd1 77384

d2bnqd1 144996

d2f53d1 145138

d2bnrd1 145000

d1j8hd1 71606

d1mi5d1 79145

d2esvd1 145124

d1fytd1 20621

d2f54d1 145142

d2f54k1 145146

d2ak4d1 144808

d2ak4i1 144812

d2ak4n1 144816

d2ak4t1 144820

d1bd2d1 20622

d1qrnd1 20624

d1qsed1 20625

d1qsfd1 20626

d1ymmd1 144702

d1ao7d_ 20623

d1ogae1 86992

d2bnub1 146159

d1kgce1 77386

d2bnqe1 144998

d2f53e1 145140

d2bnre1 145002

d2vlme1 153293

d2vlje1 153280

d2vlre1 153302

d2vlrj1 153307

d1j8he1 71608

d2ntsp1 148392

d2ij0c1 145525

d2ij0e_ 145526

d2gj6e1 135262

d2vlke1 153285

d1mi5e1 79147

d2esve1 145126

d1fyte1 20644

d2f54e1 145144

d2f54l1 145148

d2ak4e1 144810

d2ak4j1 144814

d2ak4p1 144818

d2ak4u1 144822

d1bd2e1 20645

d1qrne1 20647

d2axha1 127490

d2axhb1 127492

d1qsee1 20648

d1qsfe1 20649

d1zglp1 125047

d1zglr1 125049

d1zglt1 125051

d1zglv1 125053

d2cdea1 145027

d2cdeb1 130264

d2cded1 130266

d2cdef1 130268

d1ymme1 123705

d1ktke1 72984

d1ktkf1 72986

d2rcja1 151890

d2rcjb1 151892

d2rcje1 151894

d2rcjf1 151896

d2rcji1 151898

d2rcjj1 151900

d2rcjm1 151902

d2rcjn1 151904

d2rcjq1 151906

d2rcjr1 151908

d1ao7e1 20646

d1tvda_ 20650

d1tvdb_ 20651

d1hxmb1 61369

d1hxmd1 61373

d1hxmf1 61377

d1hxmh1 61381

d1ypze1 144708

d1ypzg1 144712

d1hxma1 61367

d1hxmc1 61371

d1hxme1 61375

d1hxmg1 61379

d2qr0b1 151236

d2qr0f1 151242

d2qr0h1 151246

d2qr0l1 151252

d2qr0n1 151256

d2qr0r1 151262

d2qr0t1 151266

d2qr0x1 151272

d1ypzf1 144710

d1ypzh1 144714

d1h5ba_ 60638

d1h5bb_ 60639

d1h5bc_ 60640

d1h5bd_ 60641

d1lp9e1 91086

d1lp9l1 91093

d1ac6a_ 20606

d1ac6b_ 20607

d2uwee1 152209

d2uwel1 152216

d1u3ha1 144383

d1u3he_ 144385

d1i9ea_ 66099

d1b88a_ 20608

d1b88b_ 20609

d1tcra1 20605

d2jcce1 147966

d2jccl1 147973

d1fo0a_ 20610

d1kj2a_ 72559

d1kj2d_ 72561

d1kb5a_ 20611

d2ol3a1 139130

d2j8ue1 147920

d2j8ul1 147927

d1nama_ 85488

d1mwaa1 79558

d1mwac1 79562

d2ckba1 20614

d2ckbc1 20615

d1nfda1 20612

d1nfdc1 20613

d1g6ra1 20616

d1g6rc1 20617

d1d9ka_ 20618

d1d9ke_ 20619

d1bwma1 20620

d2aq2a1 144824

d1beca1 20627

d2q86b1 150120

d2q86d1 150122

d1lp9f1 91088

d1lp9m1 91095

d2uwef1 152211

d2uwem1 152218

d1l0ya1 73440

d1l0yc1 73444

d2gj6d1 135260

d1sbba1 20628

d1sbbc1 20629

d1u3hb1 144384

d1u3hf_ 144386

d2icwj1 145521

d1tcrb1 20630

d2jccf1 147968

d2jccm1 147975

d2aq3a1 144825

d1fo0b_ 20631

d1kj2b_ 72560

d1kj2e_ 72562

d1kb5b_ 20632

d1l0xa1 73432

d1l0xc1 73436

d2ol3b_ 139131

d2j8uf1 147922

d2j8um1 147929

d1namb_ 85489

d1mwab1 79560

d1mwad1 79564

d2ckbb1 20637

d2ckbd1 20638

d1jcka1 20633

d1jckc1 20634

d1nfdb1 20635

d1nfdd1 20636

d1g6rb1 20639

d1g6rd1 20640

d1d9kb_ 20641

d1d9kf_ 20642

d1bwma2 20643

d1smoa_ 105764

d1smob_ 105765

d1q8ma_ 96215

d1q8mb_ 96216

d1q8mc_ 96217

d1q8md_ 96218

d1u9ka_ 113244

d1u9kb_ 113245

d1rhfa1 97469

d1rhfb1 97471

d2p45b_ 161499

d2p49b_ 149207

d3g9ab_ 176435

d2x6ma_ 169907

d2p43b_ 149199

d1yc7a_ 162178

d1yc7b_ 162179

d2p42b_ 149195

d2p42d_ 149197

d3qskb_ 184610

d2p44b_ 161498

d2p4ab_ 191679

d2p4ad_ 161504

d3ebaa_ 174815

d3eaka_ 174807

d3eakb_ 174808

d3k1kc_ 178953

d3k1kd_ 178954

d2x89a_ 169936

d2x89b_ 169937

d2x89c_ 169938

d2p48b_ 161503

d2p46b_ 161500

d2p46d_ 161501

d2p47b_ 161502

d3ogoe_ 183015

d3ogof_ 183016

d3ogog_ 183017

d3ogoh_ 183018

d1yzzb_ 193841

d3dwta_ 192719

d3dwtb_ 192720

d3dwtc_ 192721

d3dwtd_ 174313

d3dwte_ 192716

d3dwtf_ 192717

d3dwtg_ 192718

d3dwth_ 192722

d4kkna_ 197338

d2q20a_ 167393

d2q20b_ 167394

d2j12b_ 137933

d1t2ja_ 161745

d2wbwb_ 169196

d3cdca_ 173145

d3cdcb_ 173146

d3cdfa_ 173149

d3cdfb_ 173150

d3cdfc_ 173151

d3cdfd_ 173152

d3cdfe_ 173153

d3cdff_ 173154

d3dvia_ 174282

d3u79b_ 194995

d3u79c_ 194994

d3b9vd_ 172512

d3b5ga_ 172430

d3b5gb_ 172431

d3dvfa_ 196580

d3oska_ 183269

d3oskb_ 183270

d3lrgb_ 196375

d2eiza_ 161412

d3u7aa_ 194996

d2hp4a_ 165191

d2hp4b_ 165192

d2eksa_ 161415

d3bx7c_ 172894

d2j1ka_ 137941

d2j1kb_ 137942

d2j1kg_ 137943

d2j1kj_ 137944

d2j1kk_ 137945

d2j1ko_ 137946

d2j1kp_ 137947

d2j1kt_ 137948

d2j1kv_ 137949

d2j1kx_ 137950

d2j1ky_ 137951

d2j1kz_ 137952

d3rrqa_ 195364

d2w0ld_ 168984

d3bdxa_ 172565

d3bdxb_ 172566

d3bdxc_ 172567

d2q1ea_ 167389

d2q1eb_ 167390

d2q1ec_ 167391

d2q1ed_ 167392

d3p9wb_ 193748

d3p9wd_ 197349

d3p9wf_ 197427

d2yssa_ 161589

d3cdya_ 173170

d3cdyb_ 173171

d3lrho_ 180527

d2w0ka_ 168982

d2w0kb_ 168983

d2x44d_ 169850

d3upcg_ 195082

d3upch_ 195085

d3upci_ 195083

d3upcj_ 195084

d2x1qa_ 169796

d2x1qb_ 169797

d2x1pd_ 169795

d4b50a_ 196696

d2x1oa_ 169793

d2x1ob_ 169794

d2xa3a_ 169970

d4hemf_ 196676

d3qxta_ 184683

d3qxtb_ 184684

d1u0qa_ 161796

d1u0qb_ 161797

d3qxuc_ 196334

d4hepg_ 196678

d1shma_ 161716

d1shmb_ 161717

d1shmd_ 161718

d1shme_ 161719

d4eizd_ 196882

d4idla_ 194007

d3k7ua_ 179169

d1sjxa_ 161728

d4eigb_ 196883

d4aq1b_ 195192

d4aq1d_ 195193

d2wzpd_ 169729

d2wzpe_ 169730

d2wzpf_ 169731

d2wzpj_ 169732

d2wzpk_ 169733

d2wzpl_ 169734

d4iosd_ 196685

d4iose_ 196686

d4iosg_ 196684

d3k80a_ 179178

d3k80b_ 179179

d3ezjb_ 175323

d3ezjd_ 175324

d3ezjf_ 175325

d3ezjh_ 175326

d3qxve_ 196292

d4fhbd_ 196936

d3rjqb_ 195449

d2bsed_ 163157

d2bsee_ 163158

d2bsef_ 163159

d2q3aa_ 167404

d2q3ab_ 167405

d3rnqa_ 185069

d3rnka_ 185063

d1wz1h_ 121476

d3mc0a_ 180999

d3mc0c_ 181000

d2aq1a_ 161385

d2aq1c_ 161386

d2aq1e_ 161387

d2aq1g_ 161388

d2bvea_ 129256

d2bveb_ 129257

d3byya_ 172940

d2pxya_ 161514

d2pxyb_ 161515

d2oi9b_ 166701

d2oi9c_ 166702

d3ehbc_ 158152

d3ehbd_ 158153

d3hb3c_ 177355

d3hb3d_ 177356

d3byta_ 172935

d3bytc_ 172936

d3byte_ 172937

d3bytg_ 172938

d2e7la_ 163893

d2e7lb_ 163894

d2e7lc_ 163895

d2e7ld_ 163896

d2z35a_ 171016

d2z35b_ 171017

d2icwl_ 145522

d3owea_ 192770

d3owec_ 192766

d3owee_ 192771

d3oweg_ 192768

d3owei_ 192767

d3owek_ 183332

d3owem_ 192769

d3oweo_ 183333

d2cjuh_ 163422

d2aq3c_ 144826

d2aq3e_ 144827

d2aq3g_ 144828

d3bzda_ 172964

d2z31a_ 161590

d2z31b_ 161591

d3e3qa_ 174624

d3e3qc_ 193621

d3e3qe_ 174625

d3e3qf_ 174626

d3e3qj_ 174627

d3e3qn_ 174628

d3e3qs_ 174629

d3e3qw_ 174630

d2apba_ 162849

d2apxa_ 162856

d2apfa_ 162850

d2apva_ 162854

d2apwa_ 162855

d2apta_ 162852

d2aptb_ 162853

d3r8bb_ 192777

d3r8bd_ 184844

d3r8bf_ 192776

d3r8bh_ 192780

d3r8bj_ 192782

d3r8bl_ 192778

d3r8bn_ 192779

d3r8bp_ 192781

d2i25n_ 193885

d2i25o_ 161459

d2i27n_ 147490

d2i27o_ 147491

d2i26o_ 145418

d2ywza_ 170898

d2coqa_ 163461

d2z8vd_ 171107

d2z8wc_ 171108

d2z8wd_ 171109

d2ywya_ 170894

d2ywyb_ 170895

d2ywyc_ 170896

d2ywyd_ 170897

d2xt1b_ 170378

d2xxcb_ 170464

d2xxmb_ 170472

d4jvpa_ 196921

d2xv6b_ 170417

d2xv6d_ 192801

d2xfxb_ 170087

d3l9rb_ 180123

d3l9rd_ 180124

d3l9rf_ 180125

d3l9rh_ 180126

d1bmga_ 20667

d4f7eb_ 192542

d3pwve_ 191927

d4f7cb_ 192541

d4f7cd_ 192831

d1k5nb_ 77268

d3mreb_ 181499

d3czfb_ 173563

d2yxfa_ 153823

d3ib4a_ 178220

d3ln4b_ 180407

d3d25b_ 173631

d2a83b_ 126388

d3mrgb_ 181501

d3bwab_ 155685

d1i4fb_ 61689

d4fxla_ 193588

d1x7qb_ 121792

d1ogtb_ 92941

d2bvpb_ 129293

d3mrkb_ 181505

d1uxsb_ 113452

d3mrbb_ 181496

d1xh3b_ 115289

d1ogab_ 86989

d3qdaa_ 184339

d2hn7b_ 136631

d2hjlb_ 136546

d1zhlb_ 125104

d2gtwb_ 135707

d2gtwe_ 135710

d3pwlb_ 184036

d3pwle_ 184037

d2fyyb_ 134415

d3pwnb_ 184040

d3pwne_ 184041

d3dx7b_ 174315

d3gsob_ 176969

d3myza_ 181720

d3myzb_ 181721

d3fqnb_ 175982

d1uxwb_ 113455

d2v2xb_ 152435

d2v2xe_ 152438

d3mrrb_ 181512

d2vllb_ 153289

d2vlle_ 153292

d3kpmb_ 179529

d3bgmb_ 155249

d3fqrb_ 175984

d2v2wb_ 152429

d2v2we_ 152432

d3fqxb_ 175990

d3klab_ 179425

d3klae_ 179426

d2bvob_ 129290

d1m6ob_ 91191

d2gtzb_ 135717

d2gtze_ 135720

d3bh8b_ 155258

d1zhkb_ 125101

d3o3db_ 182771

d3o3de_ 182772

d3pwjb_ 184034

d3pwje_ 184035

d2gitb_ 135248

d2gite_ 135251

d3utqb_ 186406

d3kyob_ 179809

d3kyod_ 179810

d2f8oa_ 164279

d2f8ob_ 164280

d2gt9b_ 135626

d2gt9e_ 135629

d3oxsb_ 183385

d1syvb_ 112168

d3bh9b_ 155261

d3l3ib_ 179909

d3oxrb_ 183384

d3bp7b_ 172740

d2nw3b_ 138708

d3fqub_ 175987

d1tvbb_ 119350

d1tvbe_ 119353

d2d4fa_ 131250

d3d18b_ 173612

d3o3ab_ 182767

d3o3ae_ 182768

d3fqtb_ 175986

d3bw9b_ 155682

d3dx6b_ 174314

d3mrdb_ 181498

d2cikb_ 130498

d1xr9b_ 122252

d2z9ta_ 171118

d3b6sb_ 154911

d1ldsa_ 73858

d1zsdb_ 125603

d3ekca_ 175012

d3l3db_ 179906

d3bxnb_ 172923

d3bp4b_ 172738

d1n2rb_ 91563

d3bo8b_ 172729

d1jgdb_ 84169

d3ft2b_ 176051

d1jf1b_ 62929

d3gswb_ 176974

d3mrcb_ 181497

d2bnqb_ 128851

d3lkob_ 180350

d3lkqb_ 180352

d3t8xb_ 185697

d3t8xd_ 185698

d3o3eb_ 182773

d3o3ee_ 182774

d1duzb_ 20671

d1duze_ 20673

d3fqwb_ 175989

d3gsub_ 176972

d3b3ib_ 154808

d2axfb_ 127486

d2p5eb_ 149239

d3dhma_ 173951

d2h26b_ 135992

d2hjkb_ 136543

d3ft4b_ 176053

d3c9nb_ 156131

d3mrjb_ 181504

d1tvhb_ 119356

d1tvhe_ 119359

d3lkpb_ 180351

d3vh8b_ 186497

d3vh8e_ 186498

d1t1zb_ 119123

d3lv3b_ 180580

d3kppb_ 179532

d2x70b_ 169911

d2x70e_ 169912

d3ln5b_ 180408

d3h7bb_ 177302

d3h7be_ 177303

d3o3bb_ 182769

d3o3be_ 182770

d3kpqb_ 179533

d2x4qb_ 169869

d2x4qe_ 169870

d3ft3b_ 176052

d3rewb_ 184929

d3rewe_ 184930

d1i7ub_ 66064

d1i7ue_ 66067

d3myjb_ 181698

d3myje_ 181699

d3dhja_ 173950

d3gsvb_ 176973

d1t1yb_ 119120

d2h6pb_ 136203

d2guob_ 135740

d2guoe_ 135743

d3hcvb_ 177397

d2av1b_ 127351

d2av1e_ 127354

d3mrmb_ 181507

d3kpnb_ 179530

d3h9hb_ 177333

d3h9he_ 177334

d2f53b1 145137

d3givb_ 176672

d3give_ 176673

d3kplb_ 179528

d3gsrb_ 176971

d3gjfb_ 176703

d3gjfe_ 176704

d3hpjb_ 177760

d3hpje_ 177761

d2x4ub_ 169877

d2x4ue_ 169878

d2fz3b_ 134418

d3lksb_ 180354

d1vgkb_ 120061

d1w0wb_ 120565

d3mr9b_ 181495

d2av7b_ 127361

d2av7e_ 127364

d2bnrb_ 128854

d1agdb_ 20742

d3mrpb_ 181510

d1hsbb_ 20737

d2axgb_ 127489

d2p5wb_ 149262

d3na4a_ 182113

d1m05b_ 91158

d1m05d_ 91161

d1jgeb_ 77114

d3dx8b_ 174316

d1a1nb_ 20756

d1t21b_ 119129

d1w72b_ 114295

d1w72e_ 114298

d1lp9b_ 91085

d1lp9i_ 91092

d3qfdb_ 184368

d3qfde_ 184369

d1agcb_ 20744

d1t1xb_ 119117

d3kwwb_ 179761

d2bvqb_ 129296

d1t1wb_ 119114

d3l3gb_ 179907

d3gsxb_ 176975

d1t22b_ 119132

d1ydpb_ 123002

d2bssb_ 129111

d3ixab_ 178662

d3ixae_ 178663

d1of2b_ 92810

d3lknb_ 180349

d2x4rb_ 169871

d2x4re_ 169872

d3dtxb_ 174225

d1s8db_ 118888

d3lkrb_ 180353

d3mgtb_ 181205

d3mgte_ 181206

d3mgth_ 181207

d3mgtk_ 181208

d1agfb_ 20746

d3gsqb_ 176970

d3dbxb_ 173808

d1t20b_ 119126

d3ox8b_ 183366

d3ox8e_ 183367

d1hsab_ 20733

d1hsae_ 20735

d1e27b_ 20764

d1w0vb_ 120562

d2x4ob_ 169865

d2x4oe_ 169866

d1qewb_ 96343

d1jhtb_ 63065

d1im3b_ 62567

d1im3f_ 62571

d1im3j_ 62575

d1im3n_ 62579

d1duyb_ 20675

d1duye_ 20677

d1a1ob_ 20752

d3mrqb_ 181511

d3mv7b_ 181589

d2bstb_ 129114

d2x4pb_ 169867

d2x4pe_ 169868

d1ageb_ 20750

d2clrb_ 20679

d2clre_ 20681

d1xr8b_ 122249

d1agbb_ 20748

d2pyeb_ 149941

d3mgob_ 181189

d3mgoe_ 181190

d3mgoh_ 181191

d3mgok_ 181192

d3i6gb_ 178110

d3i6ge_ 178111

d3bhbb_ 155264

d3mrfb_ 181500

d1s9wb_ 105393

d3mv8b_ 181590

d2x4tb_ 169875

d2x4te_ 169876

d1qvob_ 104598

d1qvoe_ 104601

d3mrib_ 181503

d3kpob_ 179531

d1gzqb_ 70820

d1onqb_ 93367

d1onqd_ 93370

d1tmcb_ 20738

d1a1mb_ 20754

d2x4nb_ 169863

d2x4ne_ 169864

d1eeyb_ 83172

d1eeye_ 83175

d2bsrb_ 129108

d1i1yb_ 20683

d1i1ye_ 20685

d3mrlb_ 181506

d3qfjb_ 184370

d1a9eb_ 20758

d2vljb_ 153279

d3hlab_ 20687

d3d2ub_ 157255

d3d2uf_ 157258

d2x4sb_ 169873

d2x4se_ 169874

d2vlrb_ 153301

d2vlrg_ 153306

d3mrob_ 181509

d3i6le_ 178116

d3kynb_ 179808

d1s9yb_ 105399

d2hlab_ 20740

d1sysb_ 112165

d2uweb_ 152208

d2uwei_ 152215

d3l3kb_ 179911

d1i7rb_ 66052

d1i7re_ 66055

d2bckb_ 128295

d2bcke_ 128298

d2rfxb_ 152004

d3mrhb_ 181502

d3mrnb_ 181508

d1cg9b_ 90421

d1akjb_ 20689

d3lowa_ 180479

d3lowb_ 180480

d3o4lb_ 182789

d3qdjb_ 184342

d3l3jb_ 179910

d3utsb_ 186407

d3utsg_ 186408

d1qr1b_ 20713

d1qr1e_ 20715

d2gj6b_ 135259

d1q94b_ 104561

d1q94e_ 104564

d3tlrb_ 194724

d3tlrc_ 194723

d1eezb_ 83178

d1eeze_ 83181

d1qlfb_ 20818

d4en3d_ 194418

d3hujb_ 177845

d3hujd_ 177846

d2vlkb_ 153284

d1mi5b_ 79144

d1s9xb_ 105396

d3qeqb_ 184362

d3kpsb_ 179536

d2c7ub_ 130085

d2c7ue_ 130088

d2dypb_ 131898

d3kprb_ 179534

d3kprg_ 179535

d3uttb_ 186409

d3uttg_ 186410

d2esvb_ 132350

d1hhkb_ 20691

d1hhke_ 20693

d2f54b_ 132970

d2f54g_ 132973

d1qqdb_ 20770

d3i6kb_ 178114

d3i6kf_ 178115

d2ak4b_ 126902

d2ak4g_ 126905

d2ak4l_ 126908

d2ak4r_ 126911

d2ciib_ 130492

d2jccb_ 147965

d2jcci_ 147972

d1bd2b_ 20695

d3bvnb_ 172869

d3bvne_ 172870

d3bzeb_ 155759

d3bzed_ 155762

d3bzef_ 155765

d3bzeh_ 155768

d3qzwb_ 184742

d3qzwe_ 184743

d3d39b_ 173657

d1b0gb_ 20701

d1b0ge_ 20703

d3pwpb_ 184042

d3qdgb_ 184341

d3jtth_ 178826

d1mheb_ 20772

d1mhed_ 20774

d3u0pb_ 195503

d3u0pf_ 195504

d3h9sb_ 177337

d1im9b_ 62584

d1im9f_ 62589

d1qrnb_ 20699

d3qdmb_ 184343

d3tm6a_ 194722

d1xz0b_ 122463

d1xz0d_ 122466

d1e28b_ 20766

d1hhib_ 20709

d1hhie_ 20711

d1zvsb_ 125725

d1zvse_ 125726

d1ce6b_ 20820

d1i7tb_ 66058

d1i7te_ 66061

d1exub_ 20865

d3ffcb_ 175750

d3ffcg_ 175751

d1gzpb_ 70817

d1hhjb_ 20705

d1hhje_ 20707

d2f74b_ 133081

d2f74e_ 133084

d1de4b_ 20776

d1de4e_ 20778

d1de4h_ 20780

d3m17b_ 180710

d3m17d_ 180711

d3m17f_ 180712

d3m17h_ 180713

d3haeb_ 177339

d3haee_ 177340

d3haek_ 177341

d3haeq_ 177342

d3ciqa_ 173252

d3ciqb_ 173253

d3ciqc_ 173254

d3ciqd_ 173255

d3ciqe_ 173256

d3ciqf_ 173257

d3ciqg_ 173258

d3ciqh_ 173259

d3ciqi_ 173260

d3ciqj_ 173261

d3ciqk_ 173262

d3ciql_ 173263

d3mzta_ 181733

d3mztb_ 181734

d3mztc_ 181735

d3mztd_ 181736

d3mzte_ 181737

d3mztf_ 181738

d1a6zb_ 20782

d1a6zd_ 20784

d3d3vb_ 173665

d1hhgb_ 20717

d1hhge_ 20719

d2j8ub_ 147919

d2j8ui_ 147926

d3mv9b_ 181591

d2nx5b_ 138729

d2nx5g_ 138732

d2nx5l_ 138735

d2nx5r_ 138738

d1kprb_ 77482

d1kprd_ 77485

d1r3hb_ 96913

d1r3hd_ 96916

d1r3hf_ 96919

d1r3hh_ 96922

d1qseb_ 20723

d1qsfb_ 20725

d1b0rb_ 20721

d3gsnl_ 176968

d1i1fb_ 20729

d1i1fe_ 20731

d1efxb_ 20768

d2po6b1 149712

d2po6f1 149715

d1a9bb_ 20760

d1a9be_ 20762

d1ktlb_ 77537

d1ktld_ 77540

d1zt4b1 125627

d1zt4d1 125628

d1hhhb_ 20727

d2d31b1 131192

d2d31e1 131193

d1c16b_ 20857

d1c16d_ 20859

d1c16f_ 20861

d1c16h_ 20863

d3cdgb1 156514

d3cdgd1 156517

d1p7qb_ 94314

d1zs8b1 125595

d1zs8d1 125596

d1zs8f1 125597

d1zs8h1 125598

d1zs8j1 125599

d1uqsb_ 99793

d1py4a_ 95323

d1py4b_ 95324

d1py4c_ 95325

d1py4d_ 95326

d1hlam_ 20669

d1ypzb1 123839

d1ypzd1 123842

d1jnja_ 66955

d3ciib1 156672

d3ciie1 156675

d1ao7b_ 20697

d1lk2b_ 91058

d1kpub_ 84458

d1g7pb_ 70155

d3gmob_ 176752

d3g08b_ 176233

d1g7qb_ 70158

d1fzkb_ 60151

d3ecbb_ 174824

d3gmlb_ 176749

d3gmpb_ 176753

d1kpvb_ 84461

d3t1fb_ 185608

d3gmnb_ 176751

d1fzob_ 60157

d3gmmb_ 176750

d1fzmb_ 60154

d3gmqb_ 176754

d2zsvb_ 171487

d2zsvd_ 171488

d3gmrb_ 176755

d3ilpb_ 178382

d1legb_ 73871

d1t0nb_ 112207

d1t0ne_ 112210

d1fzjb_ 60148

d2fikb_ 133526

d1wbxb_ 120868

d3pwub_ 191926

d2akrb_ 126933

d2akrd_ 126936

d3p9mb_ 183608

d3p9me_ 183609

d2clvb_ 130592

d2clvp_ 130595

d3p9lb_ 183606

d3p9le_ 183607

d2q7yb_ 150108

d2q7yd_ 150111

d1s7qb_ 98657

d2clzb_ 130605

d2clzp_ 130608

d1rjyb_ 111838

d1rjye_ 111841

d4hv8b_ 192942

d4hv8d_ 192943

d1s7sb_ 98666

d1u58b_ 119531

d4huub_ 192940

d4huue_ 192941

d1jufb_ 71883

d3cc5b_ 173130

d3cc5e_ 173131

d1rk1b_ 111853

d1wbzb_ 120874

d1wbzd_ 120877

d1t0mb_ 112201

d1t0me_ 112204

d2vaab_ 20786

d1lekb_ 73874

d3fonb_ 175933

d3fond_ 175934

d3e6hb_ 174692

d1mhcb_ 20832

d1mhce_ 20834

d2vabb_ 20788

d3ilqd_ 178383

d2zokb_ 154724

d2zokd_ 154727

d2zokf_ 154730

d2zokh_ 154733

d3quyb_ 184636

d3pabb_ 183615

d3pabe_ 183616

d1vadb_ 20790

d4huxb_ 192937

d1qo3b_ 20842

d1nezb_ 85591

d1jpfb_ 67018

d1yn6b_ 123714

d3fomb_ 175932

d1inqb_ 71249

d1vacb_ 20792

d3rugd_ 191987

d1yn7b_ 123717

d3quzb_ 184637

d1s7ub_ 98675

d1s7ue_ 98678

d1s7uh_ 98681

d1s7uk_ 98684

d1kbgl_ 20794

d1s7tb_ 98669

d1s7te_ 98672

d3p4ob_ 183503

d3p4oe_ 183504

d1jpgb_ 67021

d1kj3l_ 72573

d1kj3m_ 72574

d1pqzb_ 104299

d1oszb_ 20796

d1s7vb_ 98687

d1s7ve_ 98690

d1k8db_ 68294

d1s7wb_ 98693

d1s7we_ 98696

d1s7wh_ 98699

d1s7wk_ 98702

d1s7xb_ 98705

d1s7xe_ 98708

d1s7xh_ 98711

d1s7xk_ 98714

d3p4mb_ 183499

d3p4me_ 183500

d1z5lb_ 124482

d1z5ld_ 124485

d3ch1b_ 173237

d3ch1e_ 173238

d3ch1h_ 173239

d3ch1k_ 173240

d1wbyb_ 120871

d3au1b_ 172341

d2fo4b_ 133871

d1hocb_ 20816

d3e6fb_ 174687

d1rk0b_ 111850

d1zt1b_ 125623

d3qi9b_ 191941

d4huvb_ 192938

d4huve_ 192939

d1nani_ 85495

d1nanp_ 85498

d3ma7b_ 180985

d3ma7d_ 180986

d3folb_ 175931

d3arbb_ 172307

d2fwob_ 134252

d2gazb_ 134898

d3o8xb_ 182881

d3cplb_ 173391

d3cpld_ 173392

d3ta3b_ 185738

d3o9wb_ 182900

d1rjzb_ 111844

d1rjze_ 111847

d3p4nb_ 183501

d3p4ne_ 183502

d3rtqb_ 191983

d3tvmb_ 185965

d3tvmf_ 185966

d3l3hb_ 179908

d1ld9b_ 20836

d1ld9e_ 20838

d1cd1b_ 21580

d1cd1d_ 21582

d1zhbb_ 125083

d1zhbe_ 125086

d1zhbh_ 125089

d1zhbk_ 125092

d1fo0l_ 20798

d3rzcb_ 185226

d2ve6b_ 161573

d2ve6e_ 161574

d2ve6h_ 161575

d2ve6k_ 161576

d1kj2l_ 72567

d1kj2m_ 72568

d2zswb_ 171489

d2zswd_ 171490

d2zswf_ 171491

d2zswh_ 171492

d3buyb_ 155655

d3c8kb_ 156051

d3cchb_ 173135

d3cche_ 173136

d3cchh_ 173137

d3cchk_ 173138

d3areb_ 172321

d3quxb_ 184635

d1ffpb_ 76179

d1ffpe_ 76182

d3he7b_ 177419

d3dmmb_ 174058

d1fg2b_ 20822

d1fg2e_ 20824

d1fg2h_ 20826

d1fg2k_ 20828

d1ffob_ 76173

d1ffoe_ 76176

d1zhnb_ 125107

d3ftgb_ 176059

d3scmb_ 185358

d2zolb_ 154736

d2zold_ 154739

d3cvhb_ 157004

d3cvhn_ 157008

d1bqhb_ 20800

d1bqhe_ 20802

d3arfb_ 172322

d1biib_ 20844

d2mhab_ 20804

d2mhad_ 20806

d2ol3l_ 139134

d1ffnb_ 76167

d1ffne_ 76170

d3rgvd_ 184963

d1n59b_ 80010

d1n59d_ 80013

d3he6b_ 177418

d1n5ab_ 80016

d1n5ae_ 80019

d1n5ah_ 80022

d1n5ak_ 80025

d1s7rb_ 98660

d1s7re_ 98663

d1bz9b_ 20830

d1naml_ 85492

d1mwal_ 79570

d1mwam_ 79571

d1n3nb_ 85305

d1n3nd_ 85308

d1n3nf_ 85311

d1n3nh_ 85314

d3sdab_ 185359

d1zt7b1 125629

d1zt7d1 125630

d1ddhb_ 20846

d1ldpl_ 20840

d1p4lb_ 94108

d2ckbl_ 20808

d2ckbm_ 20810

d1g6rl_ 20812

d1g6rm_ 20814

d1p1zb_ 93905

d1kjvb_ 77423

d3frub_ 20658

d3frud_ 20660

d3fruf_ 20662

d1kjmb_ 77418

d1ed3b_ 20848

d1ed3e_ 20850

d1i1ab_ 20664

d1frtb_ 20666

d3qq4b_ 184575

d3qq3b_ 184573

d3qq3e_ 184574

d2h26a1 135990

d1onqa1 93365

d1onqc1 93368

d1xz0a1 122461

d1xz0c1 122464

d1gzqa1 70818

d1gzpa1 70815

d1uqsa1 99791

d2po6a1 149710

d2po6e1 149713

d1zt4a1 144763

d1zt4c1 144765

d2fika1 133524

d2akra1 126931

d2akrc1 126934

d2q7ya1 150106

d2q7yc1 150109

d2gaza1 134896

d1cd1a1 21579

d1cd1c1 21581

d1zhna1 125105

d1hyrc1 61415

d1b3ja1 20855

d1je6a1 71638

d1r3ha1 96911

d1r3hc1 96914

d1r3he1 96917

d1r3hg1 96920

d1c16a1 20856

d1c16c1 20858

d1c16e1 20860

d1c16g1 20862

d1ypza1 123837

d1ypzc1 123840

d1k5na1 77266

d2a83a1 126386

d1i4fa1 61687

d1x7qa1 121790

d1ogta1 92939

d2bvpa1 129291

d1uxsa1 113450

d1xh3a1 115287

d1ogaa1 86987

d2hn7a1 136629

d2hjla1 136544

d1zhla1 125102

d2gtwa1 135705

d2gtwd1 135708

d2fyya1 134413

d1uxwa1 113453

d2v2xa1 152433

d2v2xd1 152436

d2vlla1 153287

d2vlld1 153290

d3bgma1 155247

d2v2wa1 152427

d2v2wd1 152430

d2bvoa1 129288

d1m6oa1 91189

d2gtza1 135715

d2gtzd1 135718

d3bh8a1 155256

d1zhka1 125099

d2gita1 135246

d2gitd1 135249

d2gt9a1 135624

d2gt9d1 135627

d1syva1 112166

d3bh9a1 155259

d2nw3a1 138706

d1tvba1 119348

d1tvbd1 119351

d2cika1 130496

d1xr9a1 122250

d3b6sa1 154909

d1zsda1 125601

d1n2ra1 91561

d1jgda1 84167

d1jf1a1 62927

d2bnqa1 128849

d1duza1 20670

d1duzd1 20672

d3b3ia1 154806

d2axfa1 127484

d2p5ea1 149237

d2hjka1 136541

d3c9na1 156129

d1tvha1 119354

d1tvhd1 119357

d1t1za1 119121

d1i7ua1 66062

d1i7ud1 66065

d1t1ya1 119118

d2h6pa1 136201

d2guoa1 135738

d2guod1 135741

d2av1a1 127349

d2av1d1 127352

d2f53a1 132966

d2fz3a1 134416

d1w0wa1 120563

d2av7a1 127359

d2av7d1 127362

d2bnra1 128852

d1agda1 20741

d1hsba1 20736

d2axga1 127487

d2p5wa1 149260

d1m05a1 91156

d1m05c1 91159

d1jgea1 77112

d1a1na1 20755

d1t21a1 119127

d1w72a1 114293

d1w72d1 114296

d1lp9a1 91083

d1lp9h1 91090

d1agca1 20743

d1t1xa1 119115

d2bvqa1 129294

d1t1wa1 119112

d1t22a1 119130

d1ydpa1 144633

d2bssa1 129109

d1of2a1 92808

d1s8da1 118886

d1agfa1 20745

d1t20a1 119124

d1hsaa1 20732

d1hsad1 20734

d1e27a1 20763

d1w0va1 120560

d1qewa1 96341

d1jhta1 63063

d1im3a1 62565

d1im3e1 62569

d1im3i1 62573

d1im3m1 62577

d1duya1 20674

d1duyd1 20676

d1a1oa1 20751

d2bsta1 129112

d1agea1 20749

d2clra1 20678

d2clrd1 20680

d1xr8a1 122247

d1agba1 20747

d2pyea1 149939

d3bhba1 155262

d1s9wa1 105391

d1qvoa1 104596

d1qvod1 104599

d1a1ma1 20753

d1eeya1 83170

d1eeyd1 83173

d2bsra1 129106

d1i1ya1 20682

d1i1yd1 20684

d1a9ea1 20757

d2vlja1 153277

d3hlaa1 20686

d2vlra1 153299

d2vlrf1 153304

d1s9ya1 105397

d2hlaa1 20739

d1sysa1 112163

d2uwea1 152206

d2uweh1 152213

d1i7ra1 66050

d1i7rd1 66053

d3bzfa1 155769

d3bzfc1 155772

d2bcka1 128293

d2bckd1 128296

d2rfxa1 152002

d1cg9a1 90419

d1akja1 20688

d1qr1a1 20712

d1qr1d1 20714

d2gj6a1 135257

d1q94a1 104559

d1q94d1 104562

d1eeza1 83176

d1eezd1 83179

d2vlka1 153282

d1mi5a1 79142

d1s9xa1 105394

d2c7ua1 130083

d2c7ud1 130086

d2dypa1 145106

d2esva1 132348

d1hhka1 20690

d1hhkd1 20692

d2f54a1 132968

d2f54f1 132971

d1qqda1 20769

d2ak4a1 126900

d2ak4f1 126903

d2ak4k1 126906

d2ak4q1 126909

d2jcca1 147963

d2jcch1 147970

d1bd2a1 20694

d3bzea1 155757

d3bzec1 155760

d3bzee1 155763

d3bzeg1 155766

d1b0ga1 20700

d1b0gd1 20702

d1mhea1 20771

d1mhec1 20773

d1im9a1 62582

d1im9e1 62587

d1qrna1 20698

d1e28a1 20765

d1hhia1 20708

d1hhid1 20710

d1i7ta1 66056

d1i7td1 66059

d1hhja1 20704

d1hhjd1 20706

d1hhga1 20716

d1hhgd1 20718

d2j8ua1 147917

d2j8uh1 147924

d2nx5a1 138727

d2nx5f1 138730

d2nx5k1 138733

d2nx5q1 138736

d1kpra1 77480

d1kprc1 77483

d1qsea1 20722

d1qsfa1 20724

d1b0ra1 20720

d1i1fa1 20728

d1i1fd1 20730

d1efxa1 20767

d1a9ba1 20759

d1a9bd1 20761

d1ktla1 77535

d1ktlc1 77538

d1hhha1 20726

d2d31a1 145067

d2d31d1 145069

d3cdga1 156512

d3cdgc1 156515

d1p7qa1 94312

d1hlaa1 20668

d3ciia1 156670

d3ciid1 156673

d1ao7a1 20696

d1lk2a1 91056

d1kpua1 84456

d1g7pa1 70153

d1g7qa1 70156

d1fzka1 60149

d1kpva1 84459

d1fzoa1 60155

d1fzma1 60152

d1lega1 73869

d1t0na1 112205

d1t0nd1 112208

d1fzja1 60146

d1wbxa1 120866

d2clva1 130590

d2clvh1 130593

d1s7qa1 98655

d2clza1 130603

d2clzh1 130606

d1rjya1 111836

d1rjyd1 111839

d1s7sa1 98664

d1vgka1 144407

d1jufa1 71881

d1rk1a1 111851

d1wbza1 120872

d1wbzc1 120875

d1t0ma1 112199

d1t0md1 112202

d2vaaa1 20785

d1leka1 73872

d1mhca1 20831

d1mhcd1 20833

d2vaba1 20787

d2zoka1 154722

d2zokc1 154725

d2zoke1 154728

d2zokg1 154731

d1vada1 20789

d1qo3a1 20841

d1neza1 85589

d1jpfa1 67016

d1yn6a1 123712

d1inqa1 71247

d1vaca1 20791

d1yn7a1 123715

d1s7ua1 98673

d1s7ud1 98676

d1s7ug1 98679

d1s7uj1 98682

d1kbgh1 20793

d1s7ta1 98667

d1s7td1 98670

d1jpga1 67019

d1kj3h1 72569

d1kj3i1 72571

d1osza1 20795

d1s7va1 98685

d1s7vd1 98688

d1k8da1 68292

d1s7wa1 98691

d1s7wd1 98694

d1s7wg1 98697

d1s7wj1 98700

d1s7xa1 98703

d1s7xd1 98706

d1s7xg1 98709

d1s7xj1 98712

d1wbya1 120869

d2fo4a1 133869

d1hoca1 20815

d1rk0a1 111848

d1zt1a1 144761

d1nanh1 85493

d1nanl1 85496

d1qlfa1 20817

d2fwoa1 145177

d2ciia1 130490

d1rjza1 111842

d1rjzd1 111845

d1ld9a1 20835

d1ld9d1 20837

d1zhba1 125081

d1zhbd1 125084

d1zhbg1 125087

d1zhbj1 125090

d1fo0h1 20797

d2ve6a1 153000

d2ve6d1 153002

d2ve6g1 153004

d2ve6j1 153006

d1kj2h1 72563

d1kj2i1 72565

d3c8ka1 156049

d1ffpa1 76177

d1ffpd1 76180

d1ce6a1 20819

d1fg2a1 20821

d1fg2d1 20823

d1fg2g1 20825

d1fg2j1 20827

d1ffoa1 76171

d1ffod1 76174

d2f74a1 133079

d2f74d1 133082

d2zola1 154734

d2zolc1 154737

d3cvha1 157002

d3cvhm1 157006

d1bqha1 20799

d1bqhd1 20801

d1biia1 20843

d2mhaa1 20803

d2mhac1 20805

d2ol3h1 139132

d1ffna1 76165

d1ffnd1 76168

d1n59a1 80008

d1n59c1 80011

d1n5aa1 80014

d1n5ad1 80017

d1n5ag1 80020

d1n5aj1 80023

d1s7ra1 98658

d1s7rd1 98661

d1bz9a1 20829

d1namh1 85490

d1mwah1 79566

d1mwai1 79568

d1n3na1 85303

d1n3nc1 85306

d1n3ne1 85309

d1n3ng1 85312

d1zt7a1 144767

d1zt7c1 144769

d1ddha1 20845

d1ldph1 20839

d1p4la1 94106

d2ckbh1 20807

d2ckbi1 20809

d1zs8a1 144749

d1zs8c1 144751

d1zs8e1 144753

d1zs8g1 144755

d1zs8i1 144757

d1g6rh1 20811

d1g6ri1 20813

d1p1za1 93903

d1kjva1 77421

d1kjma1 77416

d1ed3a1 20847

d1ed3d1 20849

d1zvsa1 144773

d1zvsd1 144775

d1hdma1 21583

d1uvqa1 100062

d1s9va1 98760

d1s9vd1 98764

d1jk8a1 63145

d1klua1 72724

d1fv1a1 21605

d1fv1d1 21607

d1d5za1 21619

d1pywa1 95377

d1d5ma1 21617

d1sjha1 105648

d1jwua1 84248

d1sjea1 105640

d1d6ea1 21621

d1t5xa1 106489

d1klga1 72714

d1j8ha1 71602

d1aqda1 21585

d1aqdd1 21587

d1aqdg1 21589

d1aqdj1 21591

d1t5wa1 106481

d1t5wd1 106485

d1jwsa1 84242

d1hxya1 61386

d1jwma1 84236

d2seba1 21593

d1fyta1 21595

d1d5xa1 21623

d1kg0a1 72441

d1r5ia1 97087

d1r5ie1 97092

d1dlha1 21597

d1dlhd1 21599

d1bx2a1 21609

d1bx2d1 21611

d1a6aa1 21615

d1seba1 21601

d1sebe1 21603

d1h15a1 76454

d1h15d1 76458

d1hqra1 21613

d1lo5a1 78116

d1k8ia1 68301

d1iaka1 21625

d2g9ha1 134802

d2p24a1 149169

d2ipka1 137577

d1muja1 79488

d2pxyc1 149922

d1k2da1 84291

d1es0a1 21643

d1u3hc1 119510

d1u3hg1 119514

d2icwa1 137243

d2icwd1 137247

d1lnua1 74097

d1lnuc1 74101

d1lnue1 74105

d1lnug1 74109

d2z31c1 153951

d2ojea1 139103

d2ojee1 139108

d2iada1 21639

d2fsea1 134017

d2fsec1 134021

d1iaoa1 21641

d1f3ja1 21645

d1f3jd1 21647

d3c60c1 155961

d3c60g1 155965

d1zgla1 125031

d1zgld1 125035

d1zglg1 125039

d1zglj1 125043

d3c6lc1 155979

d3c6lg1 155985

d1d9kc1 21627

d1d9kg1 21629

d1ymma1 123701

d1jl4a1 63157

d1fnga1 59904

d1fngc1 59908

d1fnea1 59896

d1fnec1 59900

d1ieaa1 21631

d1ieac1 21633

d1ktda1 72964

d1ktdc1 72968

d1i3ra1 61620

d1i3rc1 61624

d1i3re1 61628

d1i3rg1 61632

d1r5va1 97116

d1r5vc1 97120

d1kt2a1 72951

d1kt2c1 72955

d1ieba1 21635

d1iebc1 21637

d1r5wa1 97124

d1r5wc1 97128

d1hdmb1 21584

d1uvqb1 100064

d1s9vb1 98762

d1s9ve1 98766

d1jk8b1 63147

d1klub1 72726

d1fv1b1 21606

d1fv1e1 21608

d1d5zb1 21620

d1pywb1 95379

d1d5mb1 21618

d2g9hb1 134804

d2nnab1 148315

d2ipkb1 137579

d1sjhb1 105650

d1jwub1 84250

d1sjeb1 105642

d1d6eb1 21622

d1t5xb1 106491

d1klgb1 72716

d1j8hb1 71604

d1aqdb1 21586

d1aqde1 21588

d1aqdh1 21590

d1aqdk1 21592

d1t5wb1 106483

d1t5we1 106487

d1jwsb1 84244

d1hxyb1 61388

d1jwmb1 84238

d2sebb1 21594

d2icwb1 137245

d2icwe1 137249

d1fytb1 21596

d1d5xb1 21624

d1kg0b1 72443

d1r5ib1 97089

d1r5if1 97094

d2iamb1 137160

d2ianb1 137164

d2iang1 137168

d2ianl1 137172

d2ianq1 137176

d1dlhb1 21598

d1dlhe1 21600

d1bx2b1 21610

d1bx2e1 21612

d2ojeb1 139105

d2ojef1 139110

d1a6ab1 21616

d1sebb1 21602

d1sebf1 21604

d2fseb1 134019

d2fsed1 134023

d1h15b1 76456

d1h15e1 76460

d1hqrb1 21614

d1lo5b1 78118

d1zglb1 125033

d1zgle1 125037

d1zglh1 125041

d1zglk1 125045

d1ymmb1 123703

d1k8ib1 68303

d1iakb1 21626

d1mujb1 79490

d2pxyd1 149924

d1k2db1 84293

d1es0b1 21644

d1lnub1 74099

d1lnud1 74103

d1lnuf1 74107

d1lnuh1 74111

d3c5zd1 155955

d3c5zh1 155959

d2z31d1 153953

d2iadb1 21640

d1iaob1 21642

d1f3jb1 21646

d1f3je1 21648

d3c60d1 155963

d3c60h1 155967

d3c6ld1 155981

d3c6lh1 155987

d1d9kd1 21628

d1d9kh1 21630

d1jl4b1 63159

d1fngb1 59906

d1fngd1 59910

d1fneb1 59898

d1fned1 59902

d1ieab1 21632

d1iead1 21634

d1ktdb1 72966

d1ktdd1 72970

d1i3rb1 61622

d1i3rd1 61626

d1i3rf1 61630

d1i3rh1 61634

d1r5vb1 97118

d1r5vd1 97122

d1kt2b1 72953

d1kt2d1 72957

d1iebb1 21636

d1iebd1 21638

d1r5wb1 97126

d1r5wd1 97130

d1exua1 20864

d3frua1 20657

d3fruc1 20659

d3frue1 20661

d1i1aa1 20663

d1frta1 20665

d1de4a1 20775

d1de4d1 20777

d1de4g1 20779

d1a6za1 20781

d1a6zc1 20783

d2fbjh2 20955

d1mcph2 20959

d2mcph2 20961

d1ow0a1 87477

d1ow0b1 87479

d2qeja1 150680

d2qejb1 150682

d1ow0a2 87478

d1ow0b2 87480

d2qeja2 150681

d2qejb2 150683

d1o0va1 80745

d1o0vb1 80748

d1g84a_ 60345

d1fp5a1 21530

d1o0va2 80746

d1o0vb2 80749

d1f6ab1 21532

d1f6ad1 21534

d1fp5a2 21531

d1o0va3 80747

d1o0vb3 80750

d1f6ab2 21533

d1f6ad2 21535

d1nfdf2 21203

d1nfdh2 21205

d1c5ch2 21439

d1um5h2 113303

d1rzfh2 98151

d2fx7h2 134277

d8fabb2 20883

d8fabd2 20885

d1jpth2 67052

d1l7ih2 73657

d1jpsh2 67046

d1n7ml2 80255

d1um4h2 113299

d1n0xh2 91529

d1n0xk2 91531

d1t3fb2 112231

d1op3h2 87209

d1op3m2 87215

d2nxyd2 138750

d2fb4h2 20951

d7fabh2 20887

d1aqkh2 21209

d1um6h2 113307

d2ny2d2 138775

d2hffb2 136380

d2hffh2 136382

d1tjgh2 112447

d1rz7h2 98134

d1rzga2 98155

d1rzgc2 98159

d2ny4d2 138787

d1iqdb2 62647

d2ny3d2 138781

d2qqnh2 151225

d1vgeh2 21127

d1ce1h2 21311

d1w72h2 114300

d1w72i2 114302

d1rhhb2 97476

d1rhhd2 97480

d1tjhh2 112451

d2b1hh2 127669

d1tjih2 112455

d1c5bh2 21441

d2ny1d2 138769

d2f5bh2 88507

d2nxzd2 138756

d1u8ib2 113135

d1d5ih2 21211

d1d6vh2 21213

d1u8hb2 113131

d1gafh2 21115

d2f5ah2 88503

d2rcsh2 21119

d2pr4h2 139749

d1aj7h2 21117

d2fx9h2 134297

d2fx9i2 134299

d1tzgh2 119397

d1tzgi2 119399

d1u95b2 113231

d2ny0d2 138763

d3dggb1 157716

d3dggd1 157719

d1fl5b2 65018

d1fl5h2 65020

d1yymh2 124234

d1yymr2 124241

d2i5yh2 137073

d2i5yr2 137080

d1nl0h2 91946

d2fx8h2 134281

d2fx8i2 134283

d2fx8j2 134285

d2fx8k2 134287

d3c08h2 155810

d2b0sh2 127647

d1u8kb2 113143

d3bn9d2 155427

d3bn9f2 155429

d3c2ah2 155873

d3c2ai2 155875

d1u8jb2 113139

d2dd8h2 145074

d1om3h2 87060

d1om3k2 87062

d2ny7h2 138801

d1u92b2 113223

d3fctb2 21367

d3fctd2 21369

d1u91b2 113219

d1u8qb2 113167

d1bj1h2 21315

d1bj1k2 21317

d1zlvh2 125263

d1zlvm2 125265

d2b1ah2 127663

d1zlsh2 125256

d1u8mb2 113151

d1i7zb2 61922

d1i7zd2 61926

d1fvdb2 20963

d1fvdd2 20965

d1ad0b2 21155

d1ad0d2 21157

d2p8lb2 139527

d1rz8b2 98140

d1rz8d2 98144

d1a4jb2 21245

d1a4jh2 21243

d1u8nb2 113155

d1bz7b2 21375

d1y0lb2 122489

d1y0ld2 122493

d1y0lf2 122497

d1y0lh2 122499

d2g75a2 147087

d2g75c2 147091

d1cz8h2 21319

d1cz8y2 21321

d2i60h2 137083

d2i60r2 137090

d2ny5h2 138791

d2h9gb2 136258

d2h9gh2 136260

d1ucbh2 21133

d3difb1 157749

d3difd1 157750

d3cfjb2 156568

d3cfjd2 156572

d3cfjf2 156576

d3cfjh2 156578

d1u93b2 113227

d2hfgh2 136384

d1pg7h2 94675

d1pg7i2 94677

d1hklh2 21121

d1u8lb2 113147

d1a4kb2 21249

d1a4kh2 21247

d1g9mh2 21263

d2pw1b2 139759

d3cfkb2 156584

d3cfkd2 156588

d3cfkf2 156592

d3cfkh2 156596

d3cfki2 156598

d3cfkk2 156602

d3cfkn2 156608

d3cfkp2 156612

d1fveb2 20967

d1fved2 20969

d2nyyd2 138832

d1rzjh2 98202

d1mimh2 21159

d3dvgb2 157891

d1dfbh2 20921

d1tzhb2 107480

d1tzhh2 107482

d1gc1h2 21265

d2pw2b2 139761

d1yylh2 124224

d1yylr2 124231

d2fjfb2 133580

d2fjfd2 133582

d2fjff2 133584

d2fjfh2 133586

d2fjfi2 133588

d2fjfk2 133590

d2fjfn2 133592

d2fjfp2 133594

d2fjfr2 133596

d2fjft2 133598

d2fjfv2 133600

d2fjfx2 133602

d3b2uc2 154775

d3b2uf2 154779

d3b2uh2 154781

d3b2uj2 154785

d3b2un2 154789

d3b2uq2 154793

d3b2ut2 154797

d3b2uw2 154801

d1q1jh2 95582

d1q1ji2 95584

d1clyh2 21135

d3dvnb2 157897

d3dvnh2 157899

d1n8zb2 80321

d2p8mb2 139529

d1u6ah2 119565

d1axsb2 21217

d1axsh2 21215

d2ny6d2 138799

d2jb5h2 147956

d1fl6b2 65026

d1fl6h2 65028

d2fjgb2 133604

d2fjgh2 133606

d2p8pb2 139531

d1mhph2 84968

d1b2wh2 21299

d2fgwh2 20957

d1ad9b2 21227

d1ad9h2 21225

d1uwgh2 100116

d1uwgy2 100122

d1y18b2 122524

d1y18d2 122528

d1y18f2 122532

d1y18h2 122534

d3bkyh2 155373

d1u8ob2 113159

d2oqjb2 148985

d2oqje2 148987

d2oqjh2 148989

d2oqjk2 148991

d1hzhh2 61438

d1hzhk2 61442

d1uweh2 100104

d1uwev2 100110

d1uwey2 100114

d1zlwh2 125267

d1zlwm2 125269

d2jb6b2 147958

d2jb6h2 147960

d1bbjb2 118421

d1bbjh2 20895

d1zluh2 125259

d1zlum2 125261

d1tzib2 107490

d1it9h2 76784

d1u8pb2 113163

d1t04b2 112185

d1t04d2 112189

d1d5bb2 21221

d1d5bh2 21219

d2ig2h2 20953

d1rzkh2 98209

d1g9nh2 21267

d1za6b2 124807

d1za6d2 124810

d1za6f2 124813

d1za6h2 124816

d2qadd2 150206

d2qadh2 150210

d3c09c2 155814

d3c09h2 155818

d1pkqb2 88151

d1pkqg2 88156

d1za3b2 124790

d1za3h2 124792

d1rzib2 98169

d1rzid2 98173

d1rzif2 98177

d1rzih2 98181

d1rzij2 98185

d1rzil2 98189

d1rzin2 98193

d1rzip2 98197

d1i9rh2 71152

d1i9rk2 71154

d1i9rx2 71160

d2nz9d2 138843

d2nz9f2 138847

d1beyh2 21313

d1op5h2 87217

d1op5m2 87223

d1mcoh2 21469

d2qr0b2 151237

d2qr0f2 151243

d2qr0h2 151247

d2qr0l2 151253

d2qr0n2 151257

d2qr0r2 151263

d2qr0t2 151267

d2qr0x2 151273

d2b4ch2 127823

d3b2vh2 154803

d1s78d2 98627

d1s78f2 98631

d2rcja2 151891

d2rcjb2 151893

d2rcje2 151895

d2rcjf2 151897

d2rcji2 151899

d2rcjj2 151901

d2rcjm2 151903

d2rcjn2 151905

d2rcjq2 151907

d2rcjr2 151909

d2rcju1 151910

d1ngzb2 80503

d1ngxb2 85705

d1ngxh2 85707

d1s3kh2 105238

d1uj3b2 107891

d1ngyb2 80499

d2hh0h2 136463

d1b4jh2 21301

d1ngwb2 80491

d1ngwh2 80493

d1mjuh2 91303

d1mexh2 91258

d1ncwh2 85556

d1rukh2 97860

d1ruph2 97872

d1rumh2 97868

d3t65b2 185657

d3sy0b2 185584

d1rurh2 97880

d3bkjh1 155358

d1nlbh2 80622

d3bkmh1 155359

d3baeh1 155029

d1mj8h2 91291

d1q0xh2 95518

d1yqvh1 144718

d3t4yb2 185647

d2dquh1 146558

d1qygh2 96590

d1p7kb2 94290

d1p7kh2 94292

d1q72h2 96015

d2dqth1 146557

d1q9wb2 96323

d1q9wd2 96327

d1ruah2 97856

d2ok0h1 139122

d1kcvh2 72316

d1jguh2 66689

d3t77b2 185670

d1t2qh1 119134

d1dqqb2 21391

d1dqqd2 21393

d1nbyb2 85539

d1f3dh2 21426

d1f3dk2 21427

d1jgvh2 66693

d3cvih1 157010

d1q9ob2 96295

d1q9od2 96299

d2adfh1 126585

d1wejh2 21353

d1e6oh2 21453

d1fnsh2 21281

d3bkch1 155344

d1ruqh2 97876

d1ndgb2 85579

d1riuh2 97519

d1r3jb2 96931

d2a77h1 126328

d2brrh1 129010

d2brry1 129015

d2fath2 147016

d1kelh2 21143

d1cr9h2 21401

d1nbzb2 85544

d1yejh2 21171

d1a3lh2 21253

d1a3rh2 20891

d1q0yh2 95522

d1jfqh2 66647

d1rulh2 97864

d1yeih2 21173

d1f58h2 21343

d1mjjb2 91297

d1mjjh2 91299

d1sbsh2 21359

d2qhrh1 150794

d1yech2 21175

d1uywh1 119772

d1uywm1 119773

d1rfdh2 97373

d1mfbh2 20985

d1p2cb2 93918

d1p2ce2 93923

d1orsb2 87352

d1kn4h2 72766

d1kn2h2 72761

d1mfch2 20989

d1mfeh2 20987

d1e4wh2 59233

d2d03h1 131060

d1mieh2 91282

d1flrh2 20999

d1qkzh2 21167

d1hilb2 20897

d1hild2 20899

d12e8h2 21269

d12e8p2 21271

d2q76b1 150074

d2q76d1 150075

d1rivh2 97523

d1f8th2 59706

d1k4ca2 68127

d3clfh1 156753

d1osph2 21147

d1dqjb2 21395

d2fd6h2 145156

d1mhhb2 79122

d1mhhd2 79126

d1yegh2 21185

d1bm3h2 21099

d1yefh2 21183

d2g60h1 134684

d1sm3h2 21361

d1q9kb2 96279

d1gpoh2 21237

d1gpoi2 21239

d1mfdh2 20991

d35c8h2 21289

d1lo0h2 74116

d1lo0y2 74122

d6fabh2 21003

d1lo2h2 74124

d1lo2y2 74130

d1jglh2 66680

d1opgh2 21101

d2vdrh1 152999

d1h0db2 83428

d2aeph1 126639

d1il1a2 62540

d1teth2 20997

d1fe8h2 59787

d1fe8i2 59789

d1fe8j2 59791

d1l7th2 77800

d1e4xh2 59237

d1e4xi2 59239

d1yekh2 21177

d1i8mb2 66085

d1i8mh2 66087

d1nc2b2 91784

d1nc2d2 91788

d1ub5a2 99144

d1ub5h2 99148

d2vdoh1 152996

d1ndmb2 85584

d2z4qb1 154161

d1kemh2 21145

d2vdqh1 152998

d1cloh2 21153

d2vl5a1 153264

d2vl5c1 153265

d1mh5b2 91269

d1mh5h2 91271

d1mlbb2 21093

d2hrph2 21195

d2hrpn2 21197

d1s5hb2 105270

d1ay1h2 21255

d2ck0h2 100854

d1dqdh2 21407

d1yeeh2 21187

d1mj7h2 91287

d1oakh2 21283

d25c8h2 21291

d1c1eh2 21251

d1kcuh2 72312

d1f4xh2 21415

d1k4da2 68132

d1emth2 21447

d1ct8b2 21381

d1ct8d2 21383

d1nc4b2 91792

d1nc4d2 91796

d1qblh2 21355

d1mreh2 21073

d2vdlh1 152993

d2dtmh1 146577

d1kegh2 77346

d1mrdh2 21071

d1n64h2 80115

d1axth2 21297

d2cgrh2 21013

d2ai0h1 126809

d2ai0i1 126810

d2ai0j1 126811

d2ai0k1 126812

d1dqmh2 21397

d1a0qh2 21279

d1t66d2 106548

d1t66h2 106550

d1mrfh2 21075

d1uwxh1 119767

d1uwxm1 119768

d1ikfh2 21087

d2q8bh1 150125

d1ru9h2 97852

d1lo3h2 74132

d1lo3y2 74138

d1r3ih2 96925

d1q9lb2 96283

d1q9ld2 96287

d1jhkh2 66716

d1f4wh2 21417

d1mrch2 21077

d1indh2 20943

d2vdph1 152997

d1gigh2 21005

d1i9jh2 71145

d1qbmh2 21357

d1r3lb2 96941

d2vdkh1 152992

d1f90h2 59710

d2q8ah1 150124

d1iqwh2 66278

d2z92a1 154225

d1yehh2 21189

d1bgxh2 21257

d1fdlh2 21021

d2j4wh2 145647

d2pcpb2 21363

d2pcpd2 21365

d1ub6a2 99152

d1ub6h2 99156

d1ck0h2 90423

d2ddqh1 131397

d2h1ph2 21207

d1ncbh2 21037

d2mpah2 21349

d1rihh2 97510

d1eapb2 21069

d1ehlh2 21459

d2vdnh1 152995

d1a6tb2 21259

d1a6td2 21261

d1cf8h2 21371

d1ngph2 21123

d2vdmh1 152994

d1cicb2 21025

d1cicd2 21023

d2adgb1 126588

d2orbh1 149001

d2orbi1 149002

d1igjb2 20929

d1igjd2 20931

d1ejoh2 21399

d2jelh2 21035

d1mnuh2 21351

d2uylb1 152317

d2uyln1 152318

d2uylw1 152319

d2uyly1 152320

d3cmoh1 156836

d3cmoy1 156841

d1k6qh2 72093

d1plgh2 21113

d2r0wh1 151509

d15c8h2 21293

d1pg7x2 94685

d1pg7z2 94689

d1kcsh2 72308

d1mpah2 21165

d1forh2 21045

d1igih2 20933

d1ncah2 21039

d2oz4h1 149102

d2a1wh1 126020

d2a1wi1 126021

d1nbvh2 20993

d1ngqh2 21125

d1etzb2 21445

d1etzh2 21443

d1fgnh2 21229

d1kc5h2 72295

d3cleh1 156752

d1cu4h2 21403

d1nj9b2 91900

d1nj9h2 91902

d2bdnh2 144986

d1mf2h2 21199

d1mf2n2 21201

d1c12b2 21373

d1fl3a2 21450

d1fl3h2 21448

d1ztxh1 125659

d2j88h1 138136

d1i9ih2 71141

d1mlcb2 21095

d1mlcd2 21097

d1nd0b2 85562

d1nd0d2 85566

d1nd0f2 85570

d1nd0h2 85574

d3bt2h2 155544

d2ojzh1 139120

d2ojzi1 139121

d1mamh2 20983

d1bqlh2 21029

d2aabh1 126470

d2vc2h1 152914

d1igch2 21089

d2z91a1 154219

d2z91c1 154222

d1ifhh2 20901

d1frgh2 21019

d1ncdh2 21041

d1rmfh2 21083

d1ncch2 21043

d3f58h2 21345

d2a6ib1 126280

d1eo8h2 21287

d1wc7b2 144551

d1qfuh2 21285

d1ggbh2 20971

d1ineh2 20945

d1ibgh2 21091

d2f19h2 20947

d1pskh2 21169

d1faih2 20949

d2f58h2 21347

d1igfh2 20923

d1igfj2 20925

d1fj1b2 21149

d1fj1d2 21151

d1blnb2 21323

d1blnd2 21325

d1fh5h2 21429

d1lo4h2 74140

d1e6jh2 21455

d1bafh2 20889

d2adib1 126589

d1ggch2 20977

d1hq4b2 83624

d1hq4d2 83628

d1ggih2 20973

d1ggij2 20975

d1v7mh2 100451

d1v7mi2 100453

d1xgyh1 121985

d1xgyi1 121986

d1igtb2 20867

d1igtd2 20871

d1nldh2 21141

d2a6jb1 126281

d2a6jh1 126282

d1cbvh2 20995

d1kcrh2 72304

d1h3ph2 76649

d1jnhb2 66942

d1jnhd2 66946

d1jnhf2 66950

d1jnhh2 66954

d1kb5h2 21241

d1ghfh2 21139

d1dbjh2 20911

d2gsib2 145222

d2gsid2 145224

d2gsif2 145226

d2gsih2 145228

d2adjb1 126590

d1f11b2 21421

d1f11d2 21423

d1bbdh2 20893

d2iffh2 21031

d1dbbh2 20909

d3ck0h2 100866

d1himl2 20902

d1himm2 20904

d3cvhh1 157005

d3cvhq1 157009

d2nr6d1 148357

d2nr6f1 148358

d1dbah2 20913

d1jn6b2 66926

d2z93a1 154228

d2z93c1 154229

d2or9h1 148999

d2or9i1 149000

d1cl7.1 21389

d1r3kb2 96936

d1otsc2 87418

d1otse2 87422

d1dbmh2 20915

d1jnlh2 66957

d1a5fh2 21295

d1ai1h2 20979

d2visb2 21007

d1kfah2 77362

d1kfai2 77364

d1nsnh2 21103

d1egjh2 21457

d1fbih2 21079

d1fbiq2 21081

d2igfh2 20927

d2vitb2 21009

d1jrhh2 21235

d1bogb2 21327

d1nakh2 91754

d1naki2 91756

d1ae6h2 21223

d1yedb2 21181

d1yedh2 21179

d1iaih2 21105

d1iaii2 21107

d2virb2 21011

d1hi6b2 21329

d1r0ah2 104680

d1acyh2 20981

d1cfqb2 21331

d2dblh2 20919

d1jnnh2 66961

d2a6db1 126238

d2a6dh1 126239

d1cgsh2 21015

d1dbkh2 20917

d1yjdh1 123419

d1cfsb2 21333

d1cftb2 21337

d1f4yh2 21419

d1yuhb2 21131

d1yuhh2 21129

d2j5lc2 145664

d1hh6b2 21335

d1fpth2 21085

d1hinh2 20907

d1hh9b2 21339

d1ahwb2 21231

d1ahwe2 21233

d1knob2 21063

d1knod2 21065

d1knof2 21067

d32c2b2 21405

d2zjsh1 154578

d1cfnb2 21341

d1igyb2 20875

d1igyd2 20879

d1fskc2 21431

d1fskf2 21433

d1fski2 21435

d1fskl2 21437

d1ob1b2 86751

d1ob1e2 86757

d4fabh2 21001

d2v7hb1 152722

d2v7hh1 152723

d1orqb2 87347

d2hmid2 21275

d2a6kb1 126283

d2a6kh1 126284

d1afvh2 21161

d1afvk2 21163

d3bqud1 155504

d1v7nh2 100461

d1v7ni2 100463

d1v7nj2 100465

d1v7nk2 100467

d1yntb1 123755

d1yntd1 123756

d1n6qh2 80208

d1t03h2 99057

d1figh2 21017

d1aifb2 21109

d1aifh2 21111

d1n5yh2 80061

d1ottc2 87428

d1otte2 87432

d3hfmh2 21033

d1hysd2 61423

d1z3gh1 124402

d1z3gi1 124403

d1otuc2 87438

d1otue2 87442

d1j5oh2 71572

d1kenh2 72380

d1kent2 72384

d2dtga1 131712

d2dtgc2 145098

d1sy6h2 106114

d1vpoh2 113971

d1rjlb2 111824

d1tqbb2 107214

d1tqbc2 107216

d1tpxb2 107191

d1tpxc2 107193

d1s5ih2 105273

d1t4kb2 112238

d1t4kd2 112242

d2gfbb2 21047

d2gfbd2 21049

d2gfbf2 21051

d2gfbh2 21053

d2gfbj2 21055

d2gfbl2 21057

d2gfbn2 21059

d2gfbp2 21061

d1tqcb2 107219

d1tqcc2 107221

d1pz5b2 95404

d1m7db2 84864

d1m7ib2 84868

d1clzh2 21137

d1m71b2 84854

d1r24b2 21377

d1r24d2 21379

d1uz8b2 108169

d1uz8h2 108171

d1uz6f2 108153

d1uz6h2 108155

d1uz6p2 108161

d1uz6w2 108165

d1lk3h2 73953

d1lk3i2 73955

d1bfob2 21303

d1bfod2 21305

d1bfof2 21307

d1bfoh2 21309

d1c5db2 21387

d1c5dh2 21385

d2arjb2 144832

d2arjh2 144834

d1fn4b2 59887

d1fn4d2 59891

d2r0lh2 151503

d2r0kh2 151501

d2jixd2 148112

d2jixf2 148116

d2jixh2 148118

d1l6xa1 73642

d3dj9a1 157755

d1oqoa1 87313

d1oqob1 87315

d3c2sa1 155885

d1dn2a1 21512

d1dn2b1 21514

d2ql1a1 150859

d1h3ua1 76657

d1h3ub1 76659

d1oqxa1 87325

d1oqxb1 87327

d1h3ta1 76653

d1h3tb1 76655

d1hzhh3 61439

d1hzhk3 61443

d1h3xa1 90602

d1h3xb1 90604

d1adqa1 21518

d1fc1a1 21520

d1fc1b1 21522

d1fc2d1 21516

d1t83a1 106643

d1t83b1 106645

d2j6ea1 138080

d2j6eb1 138082

d1h3wm1 76665

d1h3va1 76661

d1h3vb1 76663

d1fcca1 21524

d1fccb1 118451

d1t89a1 106657

d1t89b1 106659

d1mcoh3 21470

d2gj7a1 135264

d2gj7b1 135266

d1e4ka1 21526

d1e4kb1 21528

d1h3ya1 76667

d1h3yb1 76669

d1igyb3 20876

d1igyd3 20880

d1igtb3 20868

d1igtd3 20872

d1i1ca1 21536

d1i1cb1 21538

d1i1ac1 21540

d1i1ad1 21542

d1frtc1 21544

d1pfca_ 21546

d1l6xa2 73643

d1oqoa2 87314

d1oqob2 87316

d3c2sa2 155886

d1dn2a2 21513

d1dn2b2 21515

d2ql1a2 150860

d1h3ua2 76658

d1h3ub2 76660

d1oqxa2 87326

d1oqxb2 87328

d1h3ta2 76654

d1h3tb2 76656

d1hzhh4 61440

d1hzhk4 61444

d1h3xa2 90603

d1h3xb2 90605

d1adqa2 21519

d1fc1a2 21521

d1fc1b2 21523

d1fc2d2 21517

d1za6b3 124808

d1za6d3 124811

d1za6f3 124814

d1za6h3 124817

d1t83a2 106644

d1t83b2 106646

d2j6ea2 138081

d2j6eb2 138083

d1h3wm2 76666

d1h3va2 76662

d1h3vb2 76664

d1fcca2 21525

d1fccb2 118452

d1t89a2 106658

d1t89b2 106660

d1mcoh4 21471

d2gj7a2 135265

d2gj7b2 135267

d1e4ka2 21527

d1e4kb2 21529

d1h3ya2 76668

d1h3yb2 76670

d1cqka_ 21547

d1cqkb_ 21548

d1igyb4 20877

d1igyd4 20881

d1igtb4 20869

d1igtd4 20873

d1i1ca2 21537

d1i1cb2 21539

d1i1ac2 21541

d1i1ad2 21543

d1frtc2 21545

d1dn0b2 20935

d1dn0d2 20937

d1deeb2 21409

d1deed2 21411

d1deef2 21413

d1hezb2 60986

d1hezd2 60990

d2agjh2 144807

d1qlrb2 20939

d1qlrd2 20941

d1adqh2 21273

d2j6eh2 145666

d2j6ei2 145668

d1c5cl2 21438

d1um5l2 113305

d2fx7l2 134279

d1jptl2 67054

d1l7il2 73659

d1jpsl2 67048

d1n7mh2 80253

d1um4l2 113301

d1n0xl2 91533

d1n0xm2 91535

d1t3fa2 112229

d1op3k2 87211

d1op3l2 87213

d1um6l2 113309

d1tjgl2 112449

d1rz7l2 98136

d1rzgb2 98157

d1rzgd2 98161

d1iqda2 62645

d1vgel2 21126

d1ce1l2 21310

d2hwzl2 147430

d1rhha2 97474

d1rhhc2 97478

d1tjhl2 112453

d1tjil2 112457

d1c5bl2 21440

d2f5bl2 88509

d1u8ia2 113133

d1d5il2 21210

d1d6vl2 21212

d1u8ha2 113129

d1gafl2 21114

d2f5al2 88505

d2rcsl2 21118

d1aj7l2 21116

d2fx9l2 134301

d2fx9m2 134303

d1tzgl2 119401

d1tzgm2 119403

d1u95a2 113229

d3dgga2 157715

d3dggc2 157718

d1fl5a2 65016

d1fl5l2 65022

d1yyml2 124236

d1yymq2 124239

d2i5yl2 137075

d2i5yq2 137078

d2fx8l2 134289

d2fx8m2 134291

d2fx8n2 134293

d2fx8o2 134295

d1dn0a2 20934

d1dn0c2 20936

d1u8ka2 113141

d1u8ja2 113137

d1om3l2 87064

d1om3m2 87066

d2ny7l2 138803

d1u92a2 113221

d3fcta2 21366

d3fctc2 21368

d1u91a2 113217

d1u8qa2 113165

d1bj1j2 21316

d1bj1l2 21314

d1u8ma2 113149

d1i7za2 61920

d1i7zc2 61924

d1fvda2 20962

d1fvdc2 20964

d1ad0a2 21154

d1ad0c2 21156

d1rz8a2 98138

d1rz8c2 98142

d1a4ja2 21244

d1a4jl2 21242

d1u8na2 113153

d1bz7a2 21374

d1y0la2 122487

d1y0lc2 122491

d1y0le2 122495

d1y0ll2 122501

d1cz8l2 21318

d1cz8x2 21320

d2i60l2 137085

d2i60q2 137088

d1ucbl2 21132

d3cfja2 156566

d3cfjc2 156570

d3cfje2 156574

d3cfjl2 156580

d1u93a2 113225

d1pg7l2 94679

d1pg7m2 94681

d1hkll2 21120

d1u8la2 113145

d1a4ka2 21248

d1a4kl2 21246

d1g9ml2 21262

d3cfka2 156582

d3cfkc2 156586

d3cfke2 156590

d3cfkg2 156594

d3cfkj2 156600

d3cfkl2 156604

d3cfkm2 156606

d3cfko2 156610

d1fvea2 20966

d1fvec2 20968

d2nyyc2 138830

d1rzjl2 98204

d1miml2 21158

d3dvga2 157889

d1dfbl2 20920

d1tzha2 107478

d1tzhl2 107484

d1gc1l2 21264

d1yyll2 124226

d1yylq2 124229

d1b6da2 21510

d1b6db2 21511

d1deea2 21408

d1deec2 21410

d1deee2 21412

d1clyl2 21134

d3dvna2 157895

d3dvnl2 157901

d1heza2 60984

d1hezc2 60988

d2agjl2 126728

d1n8za2 80319

d1u6al2 119567

d2qscl2 151317

d1axsa2 21216

d1axsl2 21214

d1fl6a2 65024

d1fl6l2 65030

d1mhpl2 84970

d1b2wl2 21298

d2fgwl2 20956

d1ad9a2 21226

d1ad9l2 21224

d1uwgl2 100118

d1uwgx2 100120

d1y18a2 122522

d1y18c2 122526

d1y18e2 122530

d1y18l2 122536

d1u8oa2 113157

d1hzhl2 61446

d1hzhm2 61448

d1uwel2 100106

d1uweu2 100108

d1uwex2 100112

d1qlra2 20938

d1qlrc2 20940

d1bbja2 118419

d1bbjl2 20894

d1tzia2 107488

d1it9l2 76786

d1u8pa2 113161

d1t04a2 112183

d1t04c2 112187

d1d5ba2 21220

d1d5bl2 21218

d1rzkl2 98211

d1g9nl2 21266

d1pkqa2 88149

d1pkqf2 88154

d1rzia2 98167

d1rzic2 98171

d1rzie2 98175

d1rzig2 98179

d1rzii2 98183

d1rzik2 98187

d1rzim2 98191

d1rzio2 98195

d2fedd2 133334

d2fedf2 133336

d1i9rl2 71156

d1i9rm2 71158

d1i9ry2 71162

d2feel2 133340

d2feeo2 133342

d1beyl2 21312

d1op5k2 87219

d1op5l2 87221

d2qr0a2 151235

d2qr0e2 151241

d2qr0g2 151245

d2qr0k2 151251

d2qr0m2 151255

d2qr0q2 151261

d2qr0s2 151265

d2qr0w2 151271

d2fecl2 133330

d2feco2 133332

d2b4cl2 127825

d1s78c2 98625

d1s78e2 98629

d1ngza2 80501

d1ngxa2 85703

d1ngxl2 85709

d1s3kl2 105240

d1uj3a2 107889

d1ngya2 80497

d2hh0l2 136465

d1b4jl2 21300

d1ngwa2 80489

d1ngwl2 80495

d1mjul2 91305

d1mexl2 91260

d1ncwl2 85558

d1rukl2 97862

d1rupl2 97874

d1ruml2 97870

d3t65a2 185655

d3sy0a2 185582

d1rurl2 97882

d1nlbl2 80624

d1mj8l2 91293

d1yqvl2 123901

d3t4ya2 185645

d1qygl2 96592

d1p7ka2 94288

d1p7kl2 94294

d1q72l2 96017

d1q9wa2 96321

d1q9wc2 96325

d1rual2 97858

d1kcvl2 72318

d1jgul2 66691

d3t77a2 185668

d1dqqa2 21390

d1dqqc2 21392

d1nbya2 85537

d1f3dj2 21425

d1f3dl2 21424
[truncated: 1,984,586 more chars]
